# Supplementary material for: The sponge effect and carbon emission mitigation potentials of the global cement cycle
Source: Nat Commun. 2020 Jul 29;11:3777. doi: 10.1038/s41467-020-17583-w (PMC7392754; doi:10.1038/s41467-020-17583-w)
Supplement: Supplementary file 1 — Supplementary Information [file 41467_2020_17583_MOESM1_ESM.pdf]

Supplementary Information

The sponge effect and carbon emission mitigation potentials of the global cement cycle

Cao et al.

Contents

Supplementary Figures 8
Supplementary Figure 1 8
Supplementary Figure 2 9
Supplementary Figure 3 10
Supplementary Figure 4 11
Supplementary Figure 5 12
Supplementary Figure 6 13
Supplementary Figure 7 14
Supplementary Figure 8 15
Supplementary Figure 9 16
Supplementary Figure 10 17
Supplementary Figrue 11 18
Supplementary Figure 12 19
Supplementary Figure 13 20
Supplementary Figure 14 22
Supplementary Figure 15 24
Supplementary Figrue 16 26
Supplementary Figure 17 28
Supplementary Figure 18 30
Supplementary Figure 19 32
Supplementary Figure 20 34
Supplementary Figure 21 36
Supplementary Figure 22 38
Supplementary Figure 23 40
Supplementary Figure 24 41
Supplementary Figure 25 42
Supplementary Figure 26 43
Supplementary Figure 27 44
Supplementary Figure 28 45
Supplementary Figure 29 46
Supplementary Figure 30 47

Supplementary Figure 31 48
Supplementary Figure 32 49
Supplementary Figure 33 50
Supplementary Figure 34 51
Supplementary Figure 35 52
Supplementary Figure 36 53
Supplementary Figure 37 54
Supplementary Figure 38 55
Supplementary Figure 39 56
Supplementary Figure 40 57
Supplementary Figure 41 58
Supplementary Figure 42 59
Supplementary Figure 43 60
Supplementary Figure 44 61
Supplementary Figure 45 62
Supplementary Figure 46 63
Supplementary Figure 47 64
Supplementary Figure 48 65
Supplementary Figure 49 66
Supplementary Figrue 50 67
Supplementary Figure 51 68
Supplementary Figure 52 69
Supplementary Figure 53 70
Supplementary Figure 54 71
Supplementary Figure 55 72
Supplementary Figure 56 73
Supplementary Figure 57 74
Supplementary Figure 58 75
Supplementary Figure 59 76
Supplementary Figure 60 77
Supplementary Figure 61 78
Supplementary Figure 62 79
Supplementary Figure 63 80

Supplementary Figure 64 81
Supplementary Figure 65 82
Supplementary Figure 66 83
Supplementary Figure 67 84
Supplementary Figure 68 85
Supplementary Figure 69 86
Supplementary Figure 70 87
Supplementary Figure 71 88
Supplementary Figure 72 89
Supplementary Figure 73 90
Supplementary Figure 74 91
Supplementary Figure 75 92
Supplementary Figure 76 93
Supplementary Figure 77 94
Supplementary Figure 78 95
Supplementary Figure 79 96
Supplementary Figure 80 97
Supplementary Figure 81 98
Supplementary Figure 82 99
Supplementary Figure 83 100
Supplementary Figure 84 101
Supplementary Figure 85 102
Supplementary Figure 86 103
Supplementary Figure 87 104
Supplementary Figure 88 105
Supplementary Figure 89 106
Supplementary Figure 90 107
Supplementary Figure 91 108
Supplementary Figure 92 109
Supplementary Figure 93 110
Supplementary Figure 94 111
Supplementary Figure 95 112
Supplementary Figure 96 113

Supplementary Figrue 97 115
Supplementary Figure 98 116
Supplementary Figure 99 117
Supplementary Figure 100 119
Supplementary Figure 101 120
Supplementary Figure 102 121
Supplementary Figure 103 122
Supplementary Figure 104 123
Supplementary Figure 105 124
Supplementary Tables 125
Supplementary Table 1 125
Supplementary Table 2 126
Supplementary Table 3 131
Supplementary Table 4 132
Supplementary Table 5 136
Supplementary Table 6 137
Supplementary Table 7 139
Supplementary Table 8 140
Supplementary Table 9 141
Supplementary Table 10 142
Supplementary Table 11 143
Supplementary Table 12 145
Supplementary Table 13 146
Supplementary Table 14 147
Supplementary Table 15 148
Supplementary Table 16 149
Supplementary Note 1. Geographic coverage and aggregation 150
Supplementary Note 2. Dynamic material flow analysis model 150
Supplementary Note 2.1. Top-down stock-flow estimation approach (Retrospective cycle: 1930-2014) 150
Supplementary Note 2.2. Stock-driven approach (Prospective cycle: 2015-2100) 151
Supplementary Note 2.3. Scenario indicators by regions and by end-use sectors 152
Supplementary Note 2.4. Cement inflows (sectoral and total) 152

Supplementary Note 3. Cement technology roadmap 154
Supplementary Note 3.1. Thermal efficiency..155
Supplementary Note 3.2. Electric efficiency 155
Supplementary Note 3.3. Alternative fuel 155
Supplementary Note 3.4. Clinker substitution 156
Supplementary Note 3.5. Carbon capture and storage (CCS) 156
Supplementary Note 4. Cement carbonation model 157
Supplementary Note 4.1. Uptake by cement kiln dust...157
Supplementary Note 4.2. Uptake by construction wastes 157
Supplementary Note 4.3. Uptake by concrete..158
Supplementary Note 4.3.1. Concrete in use stage 158
Supplementary Note 4.3.2. Concrete in demolition and secondary use stages 159
Supplementary Note 4.4. Uptake by mortar 161
Supplementary Note 4.4.1. Mortar for rendering and plastering in use stage 161
Supplementary Note 4.4.2. Mortar for masonry with rendering on both sides in use stage........ 161
Supplementary Note 4.4.3. Mortar for masonry with rendering on only one side in use stage... 162
Supplementary Note 4.4.4. Mortar for masonry without rendering in use stage........................ 163
Supplementary Note 4.4.5. Mortar for repairing and maintenance in use stage 163
Supplementary Note 4.4.6. Mortar in demolition and secondary use stages 164
Supplementary Note 4.5. Data compilation and uncertainties..164
Supplementary Note 4.5.1. CaO content in clinker.. 164
Supplementary Note 4.5.2. Proportion of CaO converted to $CaCO_3$... 164
Supplementary Note 4.5.3. Market shares of cement used for concrete 164
Supplementary Note 4.5.4. Market shares of concrete strength classes 164
Supplementary Note 4.5.5. Cement content of concrete 164
Supplementary Note 4.5.6. Carbonation rates of different strength classes 164
Supplementary Note 4.5.7. Correction factor of cement additives.. 165
Supplementary Note 4.5.8. Correction factor of CO_2 concentration.. 165
Supplementary Note 4.5.9. Correction factor of coating and cover.. 165
Supplementary Note 4.5.10. Breakdown of demolition waste by particle sizes......................... 165
Supplementary Note 4.5.11. Exposure time during the demolition stage.................................. 165
Supplementary Note 4.5.12. Breakdown of secondary uses of demolition wastes 165
Supplementary Note 4.5.13. Market shares of cement used for mortar.................................... 165

Supplementary Note 4.5.14. Breakdown of mortar uses 165
Supplementary Note 4.5.15. Thickness of different mortar uses 165
Supplementary Note 4.5.16. Proportions of masonry walls with rendering 165
Supplementary Note 4.5.17. Thickness of concrete structures.. 165
Supplementary Note 4.5.18. Carbonation rate of mortar 166
Supplementary Note 4.5.19. Loss rates of cement in the construction stage............................ 166
Supplementary Note 4.5.20. Carbonation time of construction waste from concrete 166
Supplementary Note 4.5.21. CKD generation rate based on clinker... 166
Supplementary Note 4.5.22. Proportion of landfilled CKD 166
Supplementary Note 4.5.23. CaO content of CKD 166
Supplementary Note 5. Uncertainties and sensitivities 167
Supplementary Note 5.1. Sensitivity to lifetime 167
Supplementary Note 5.2. Sensitivity to population 167
Supplementary Note 5.3. Lifetime extension..167
Supplementary References 168

Supplementary Figures

Supplementary Figure 1

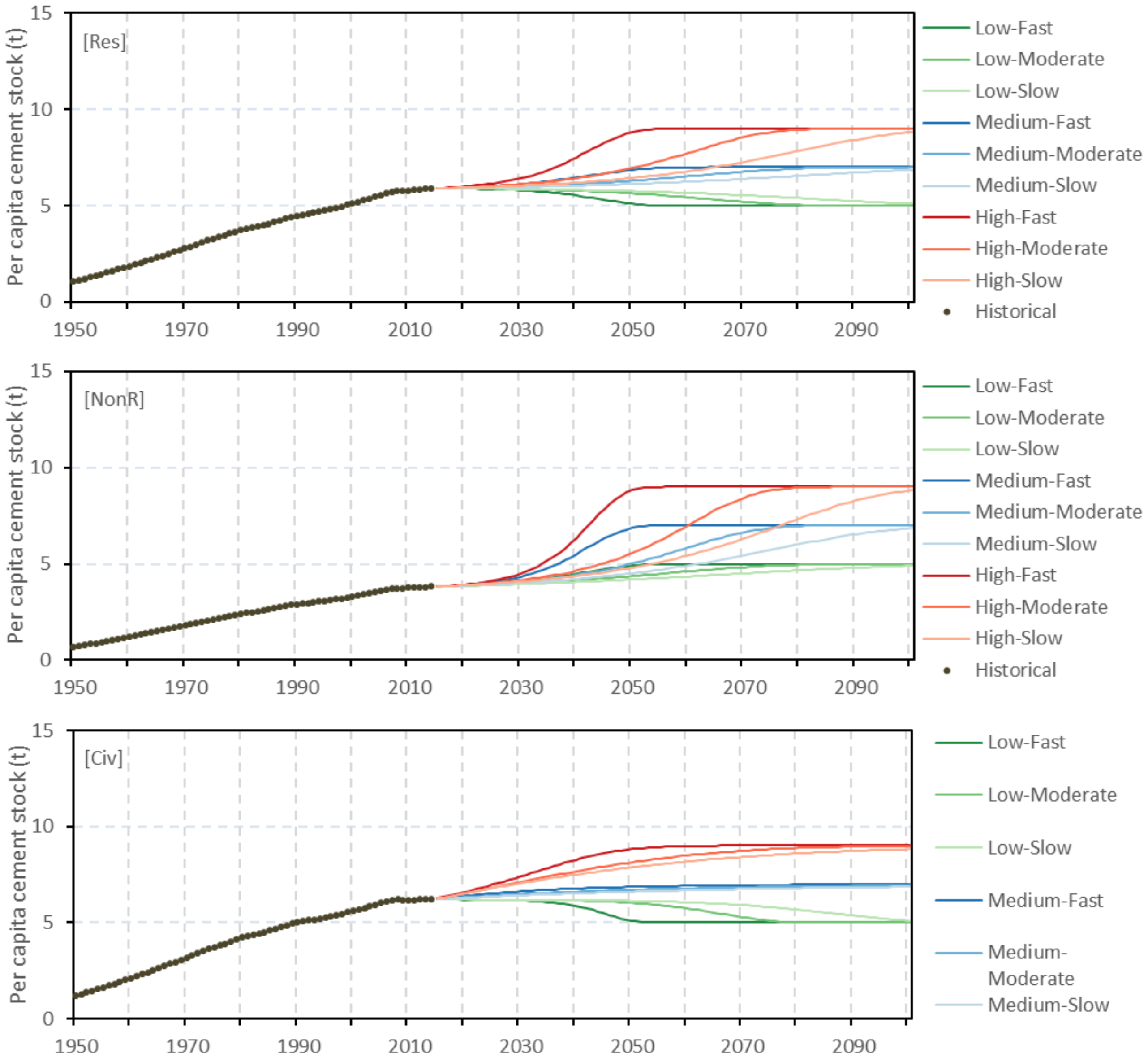

Supplementary Figure 1 | Per capita cement stock patterns in North America by end-use sectors

Supplementary Figure 2

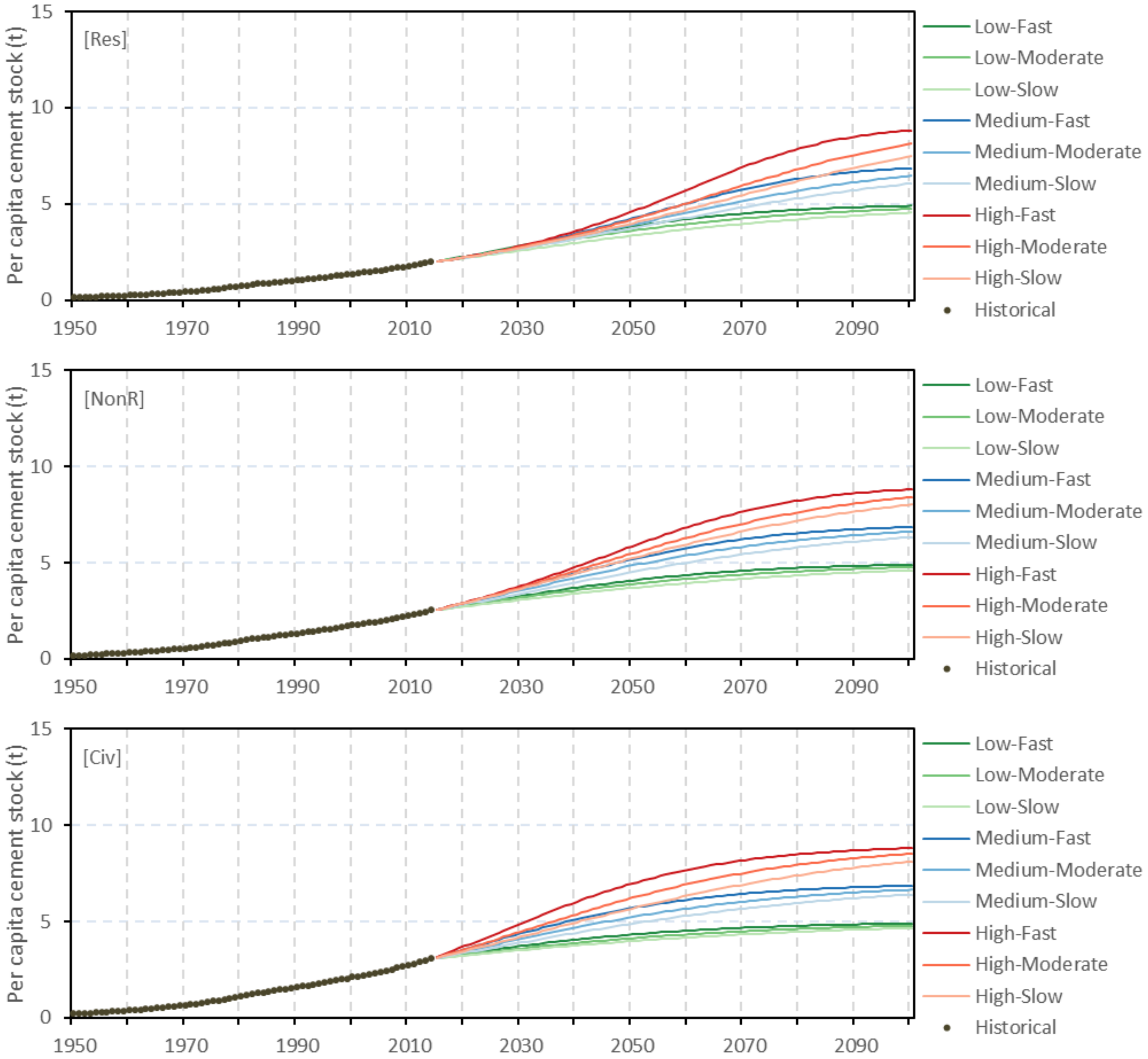

Supplementary Figure 2 | Per capita cement stock patterns in Latin America & Caribbean by end-use sectors

Supplementary Figure 3

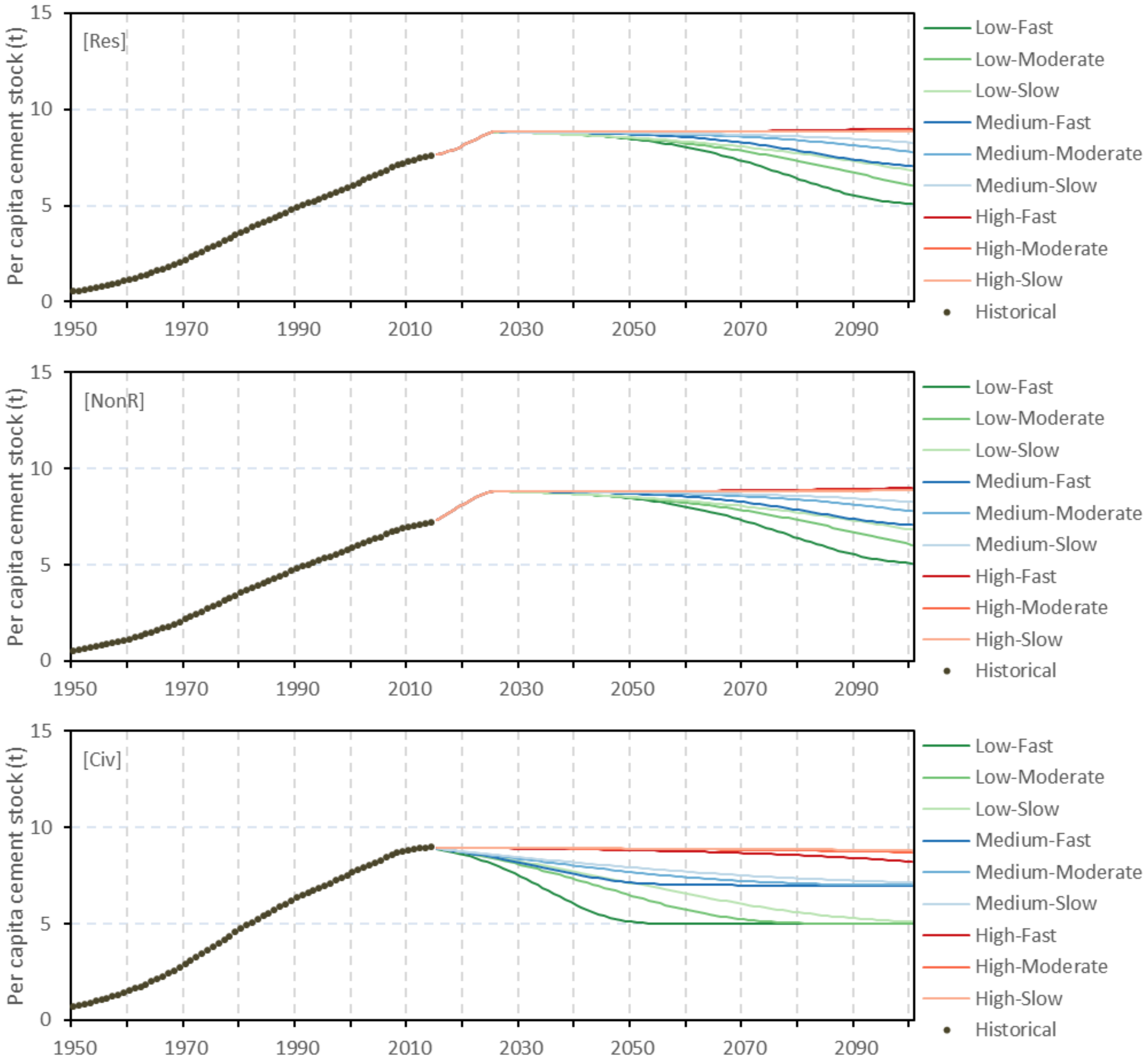

Supplementary Figure 3 | Per capita cement stock patterns in Europe by end-use sectors

Supplementary Figure 4

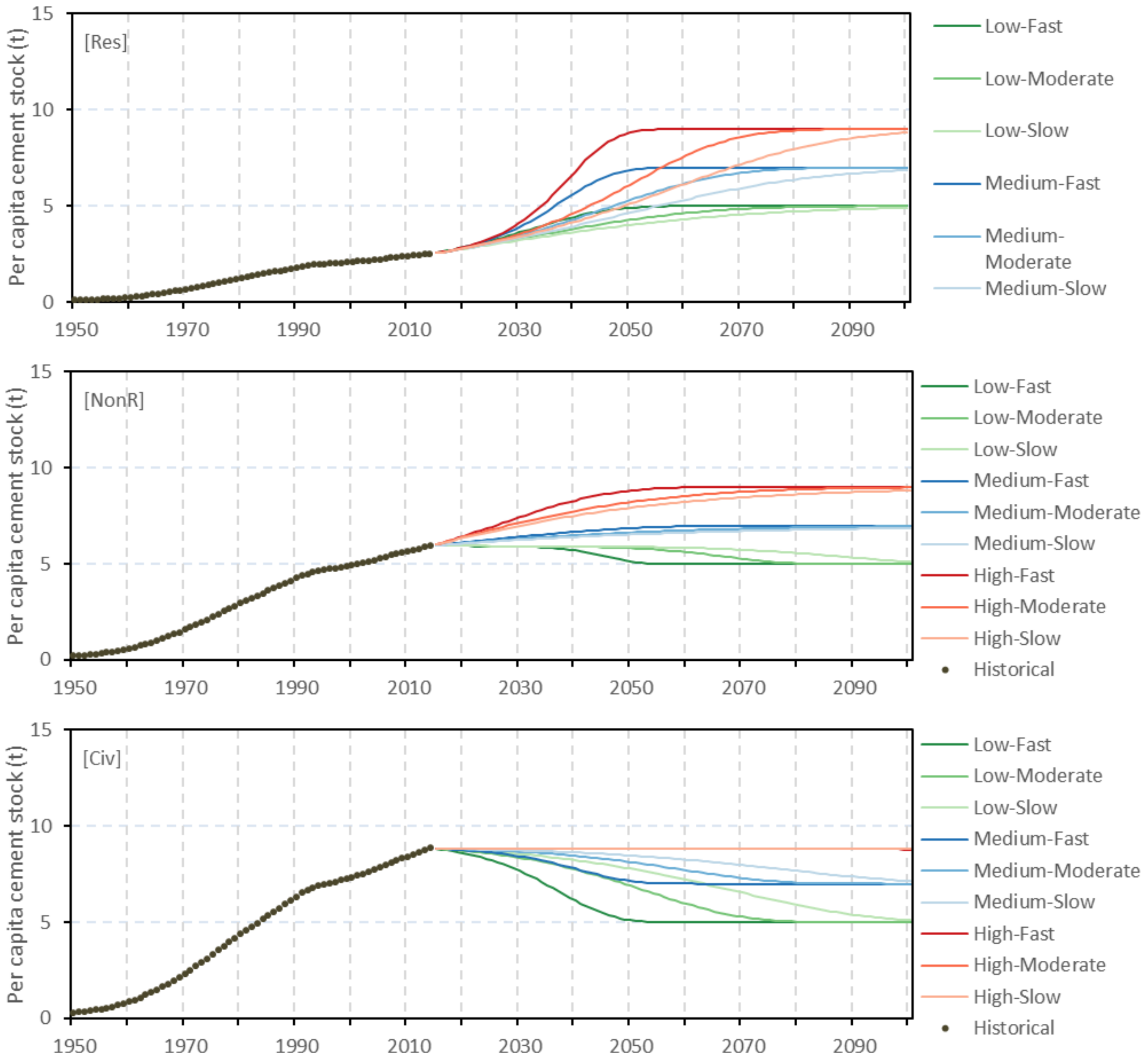

Supplementary Figure 4 | Per capita cement stock patterns in Commonwealth of Independent States by end-use sectors

Supplementary Figure 5

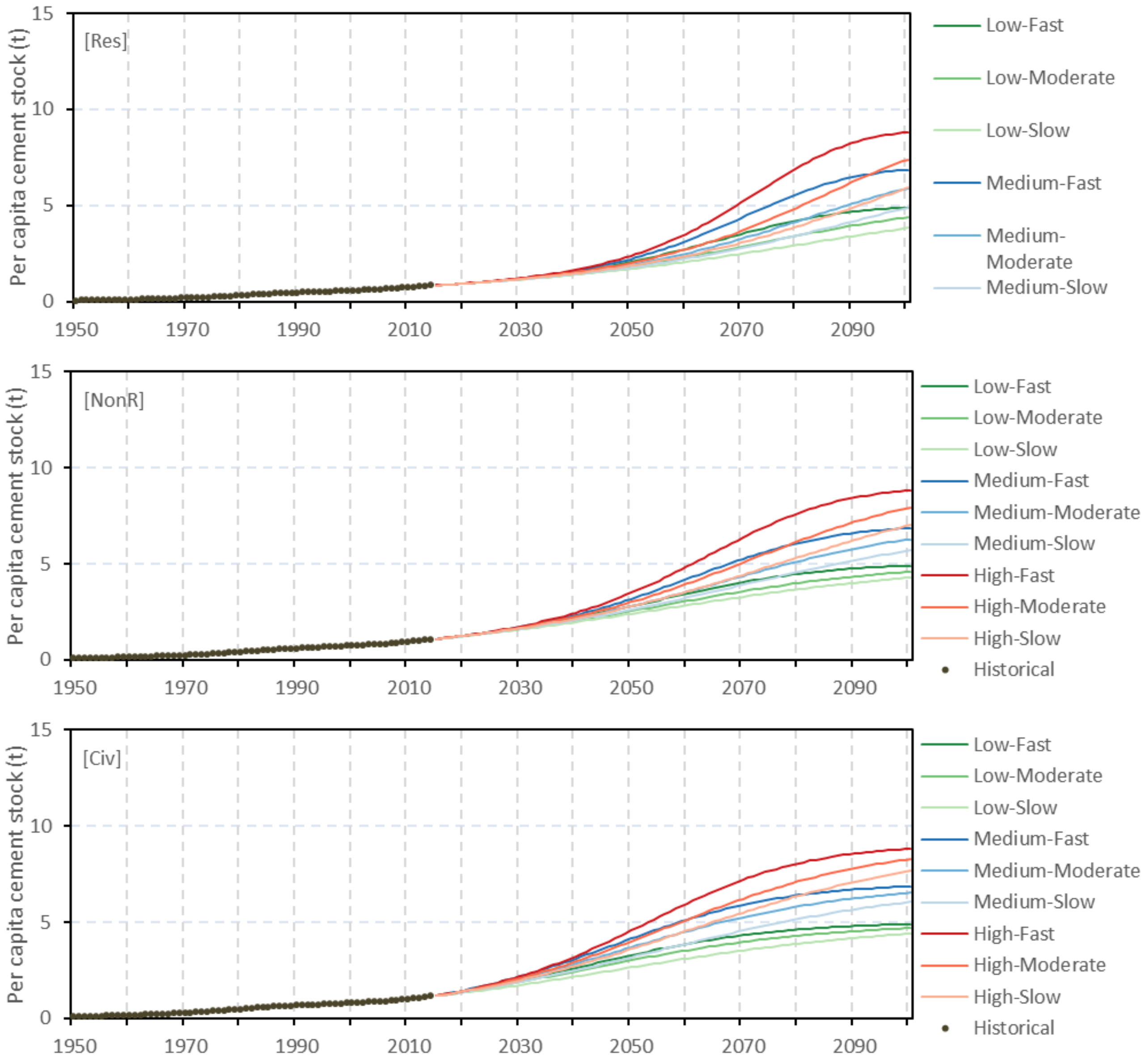

Supplementary Figure 5 | Per capita cement stock patterns in Africa by end-use sectors

Supplementary Figure 6

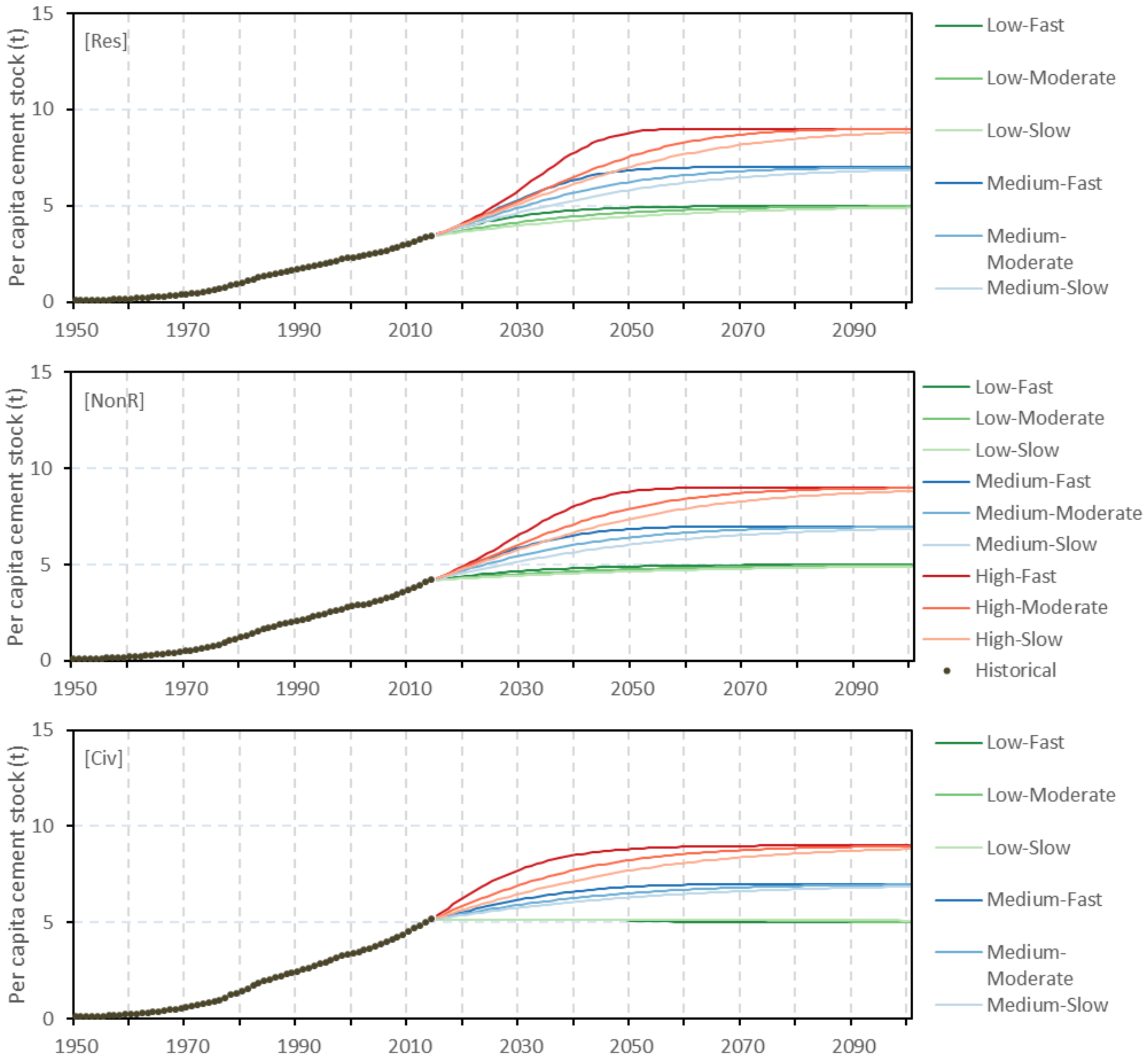

Supplementary Figure 6 | Per capita cement stock patterns in Middle East by end-use sectors

Supplementary Figure 7

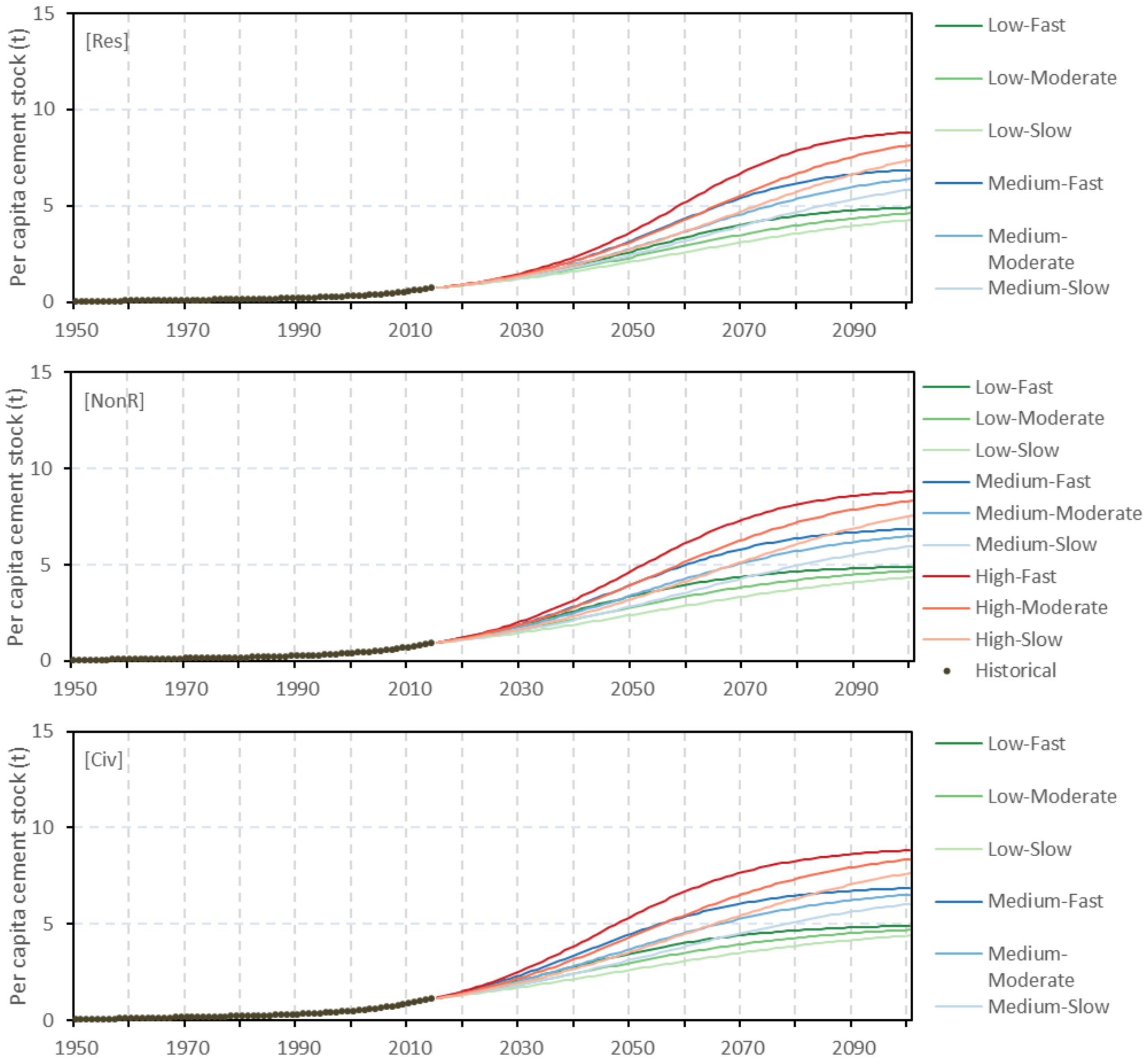

Supplementary Figure 7 | Per capita cement stock patterns in India by end-use sectors

Supplementary Figure 8

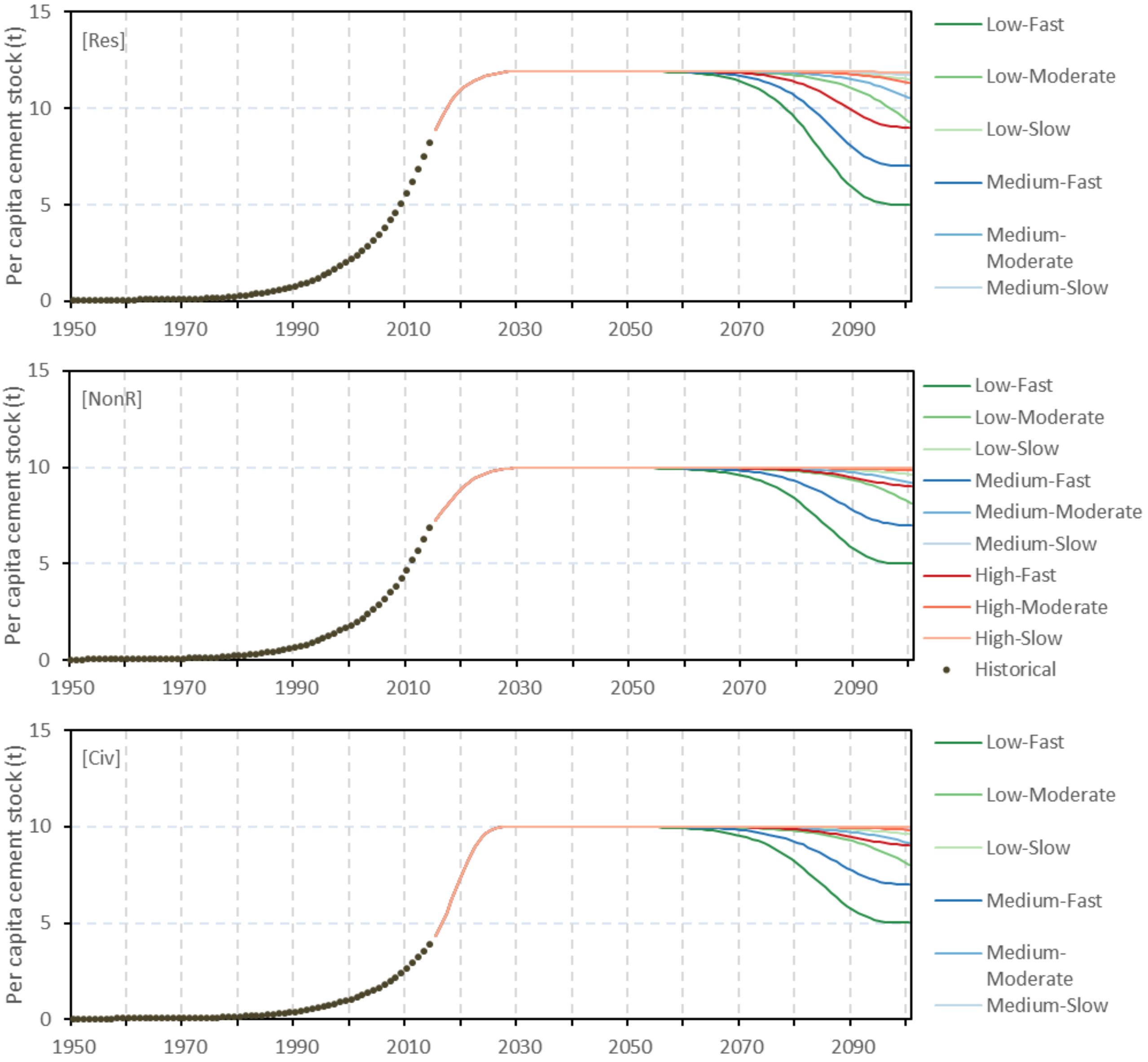

Supplementary Figure 8 | Per capita cement stock patterns in China by end-use sectors

Supplementary Figure 9

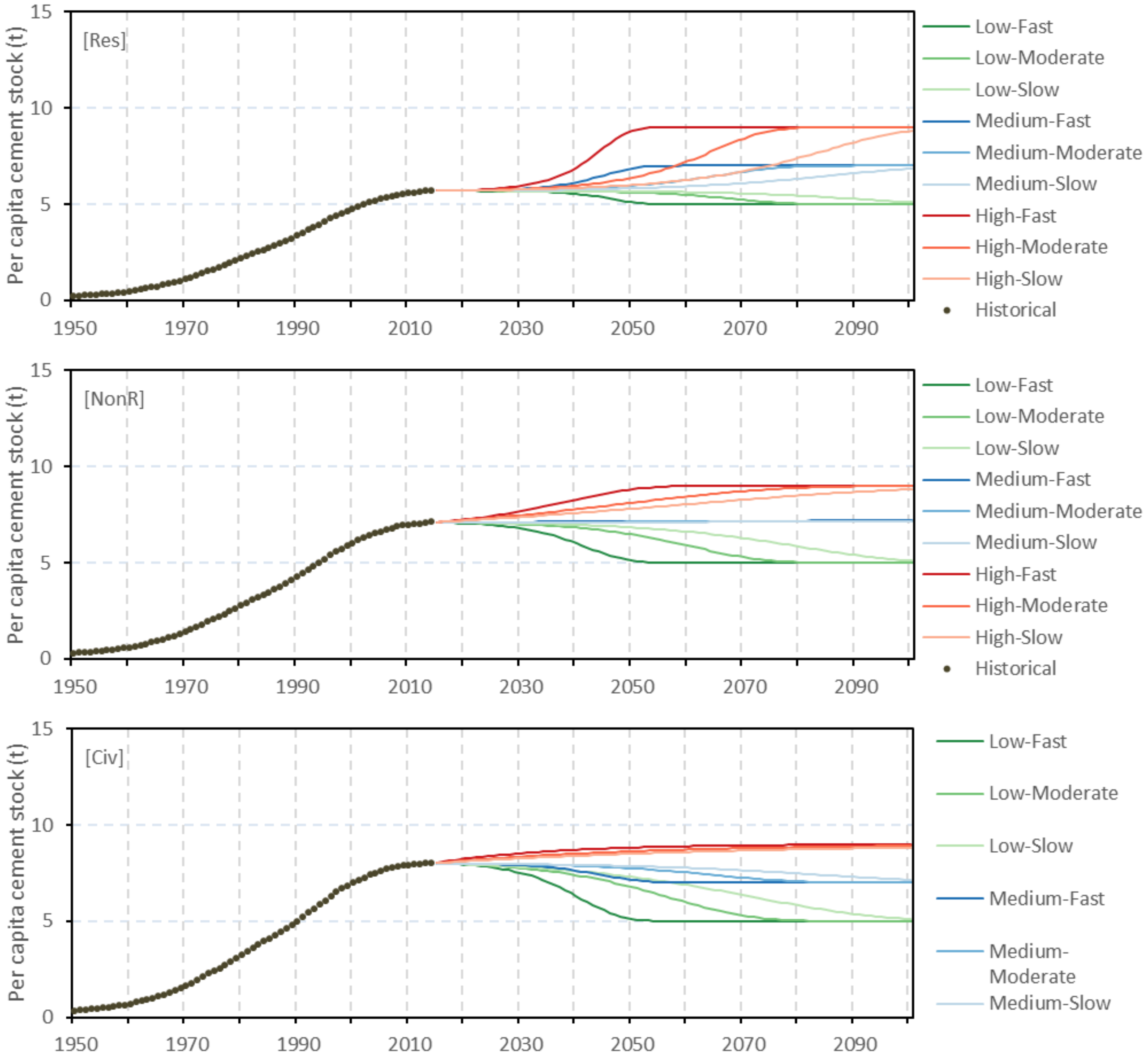

Supplementary Figure 9 | Per capita cement stock patterns in Developed Asia & Oceania by end-use sectors

Supplementary Figure 10

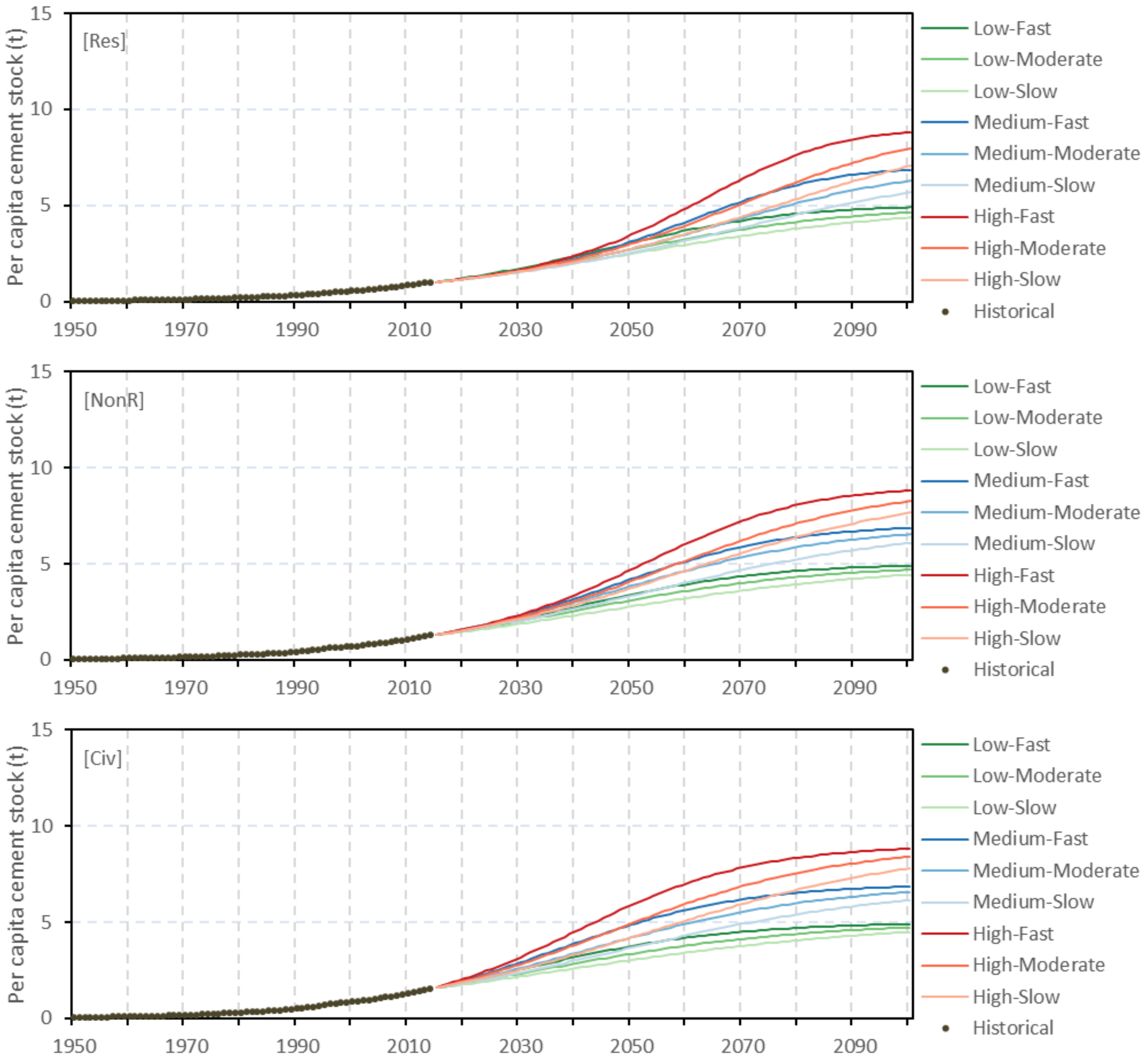

Supplementary Figure 10 | Per capita cement stock patterns in Developing Asia by end-use sectors

Supplementary Figrue 11

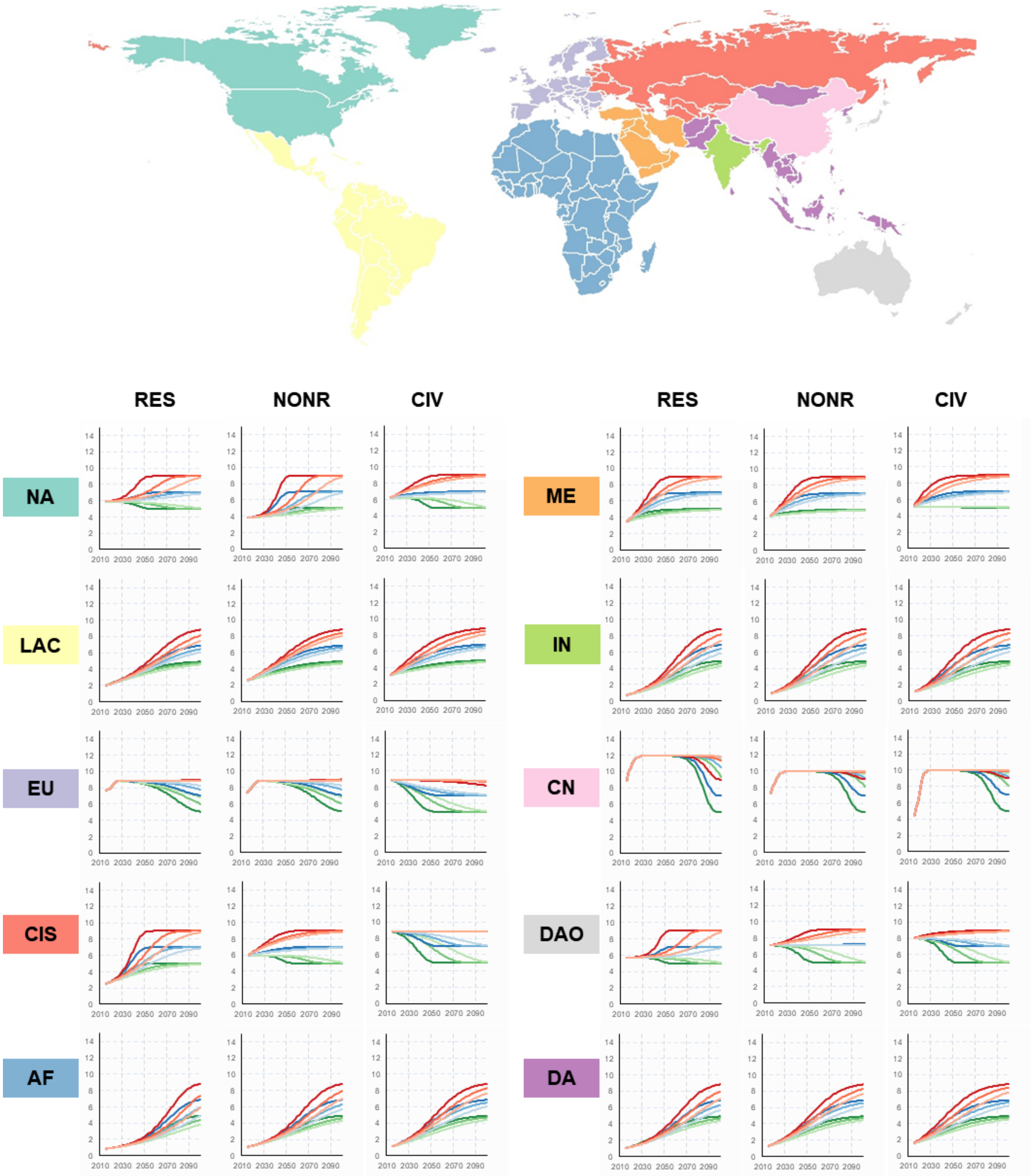

Supplementary Figure 11 | Future patterns of per capita cement in-use stocks (Unit: t/cap)

Supplementary Figure 12

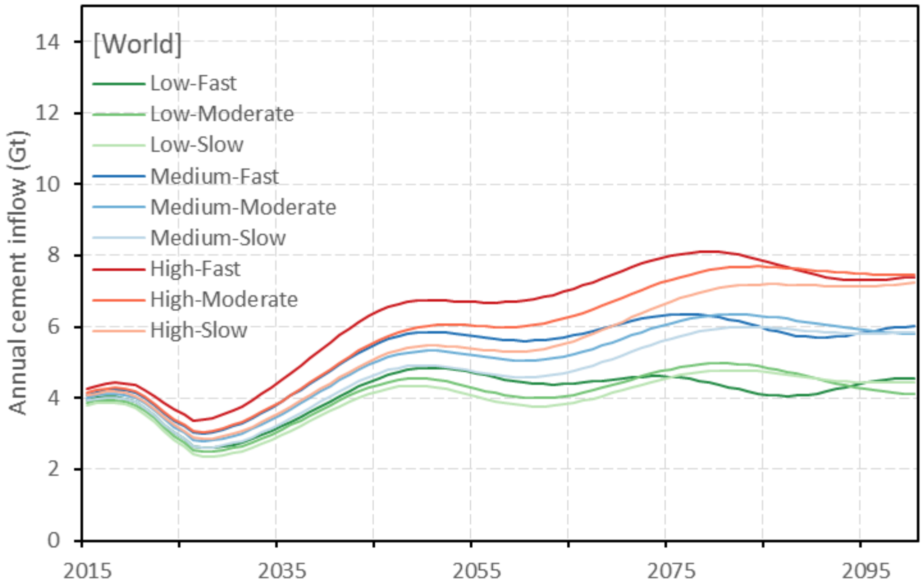

Supplementary Figure 12 | Cement inflows in the world (total)

Supplementary Figure 13

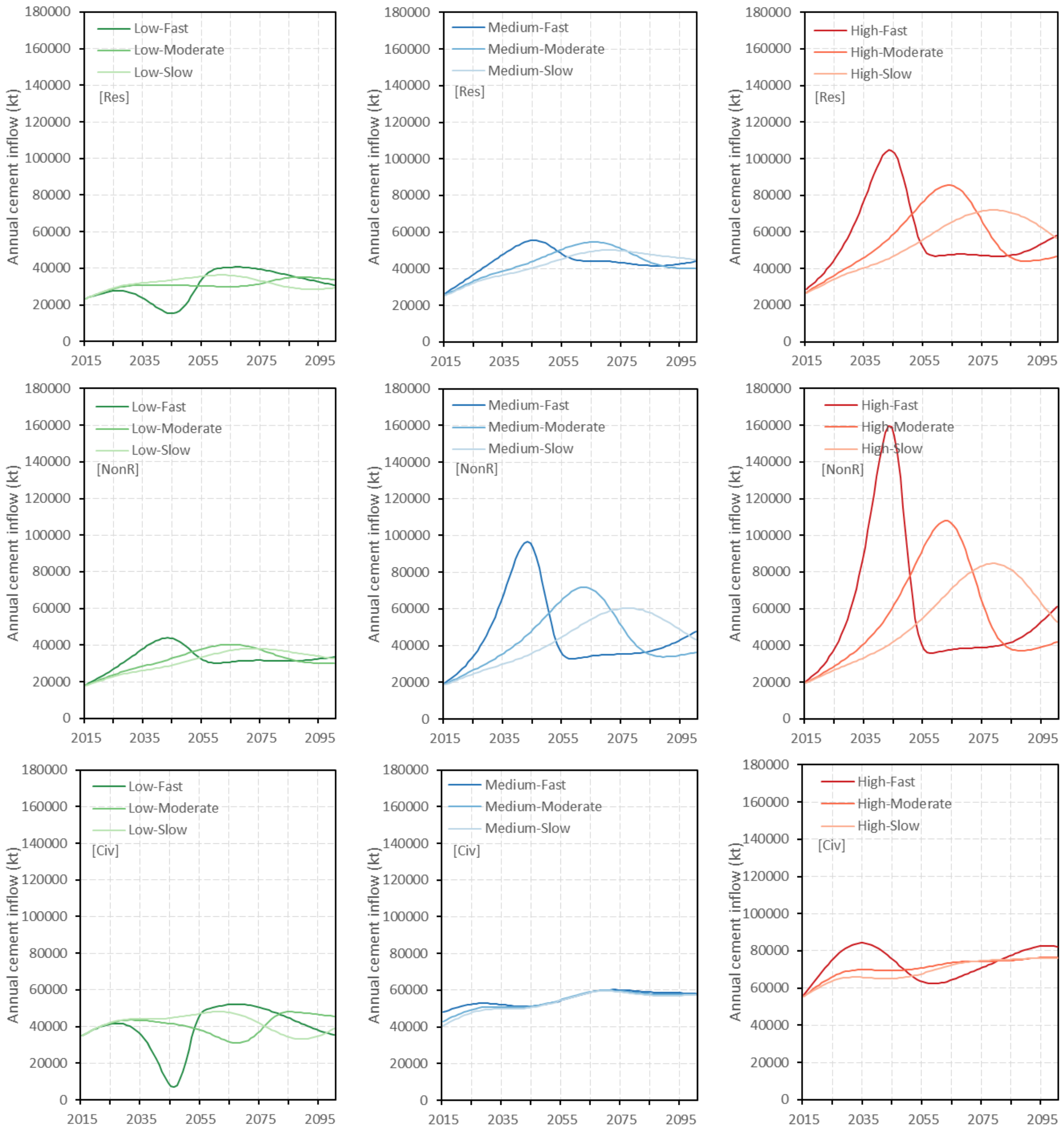

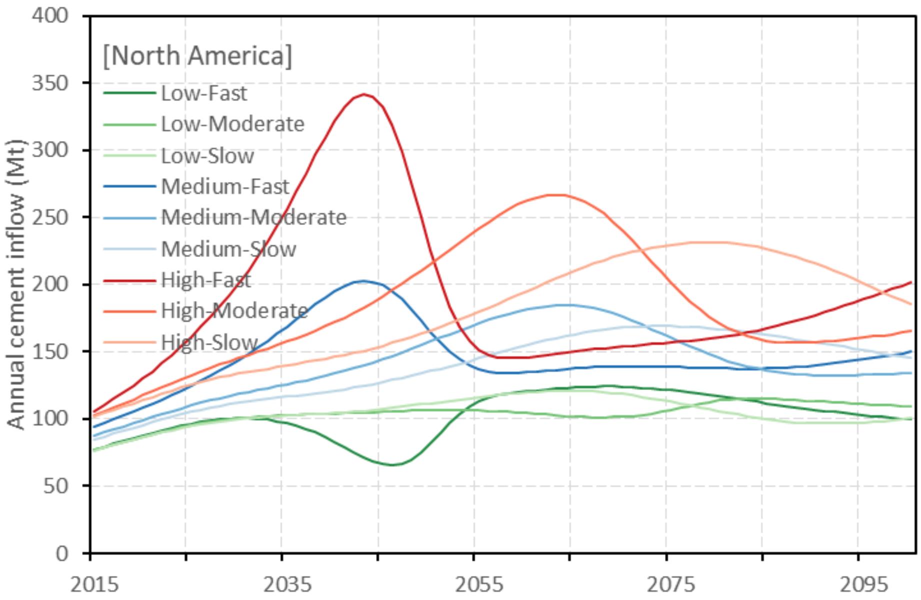

Supplementary Figure 13 | Cement inflows in North America (sectoral and total)

Supplementary Figure 14

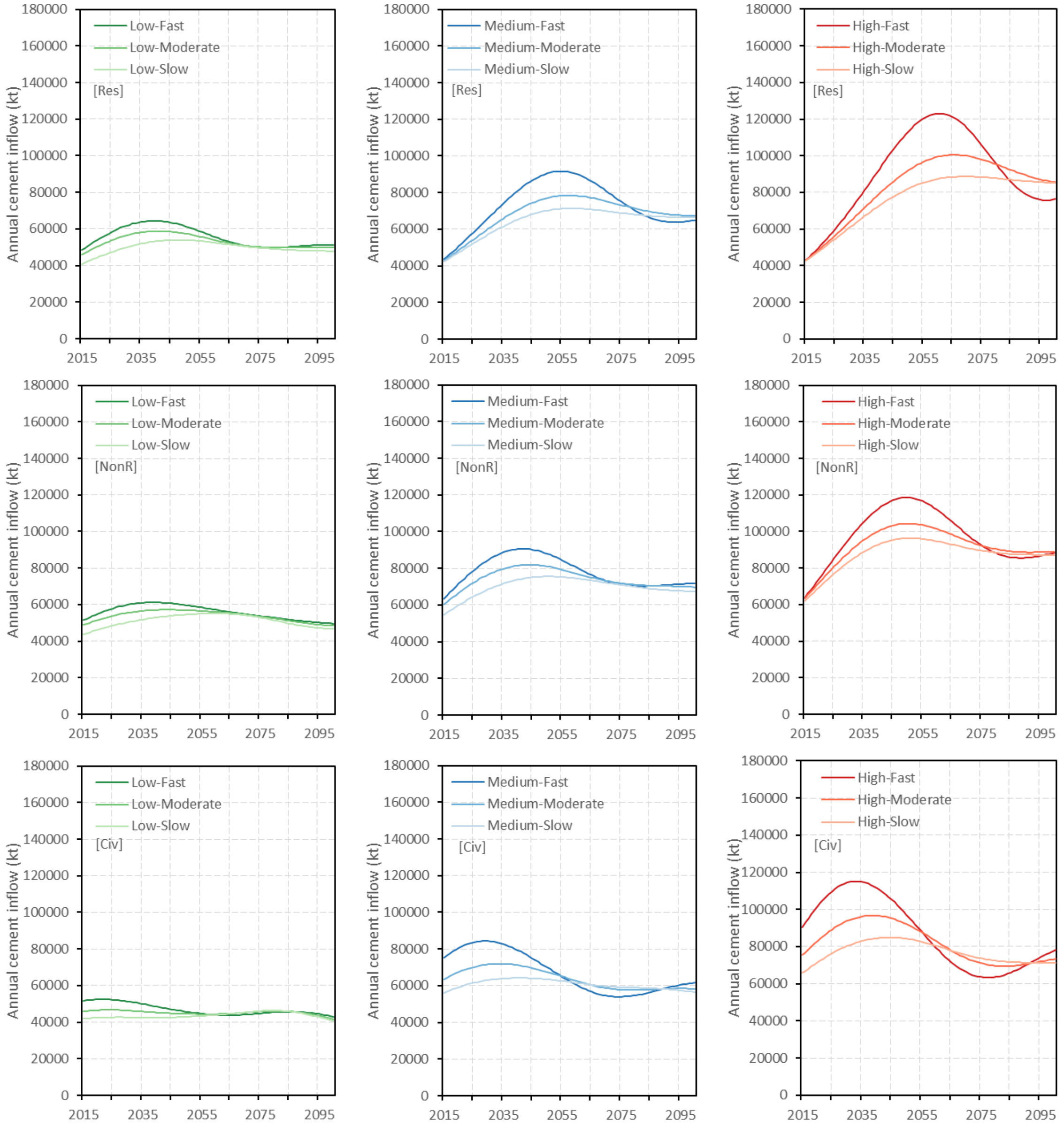

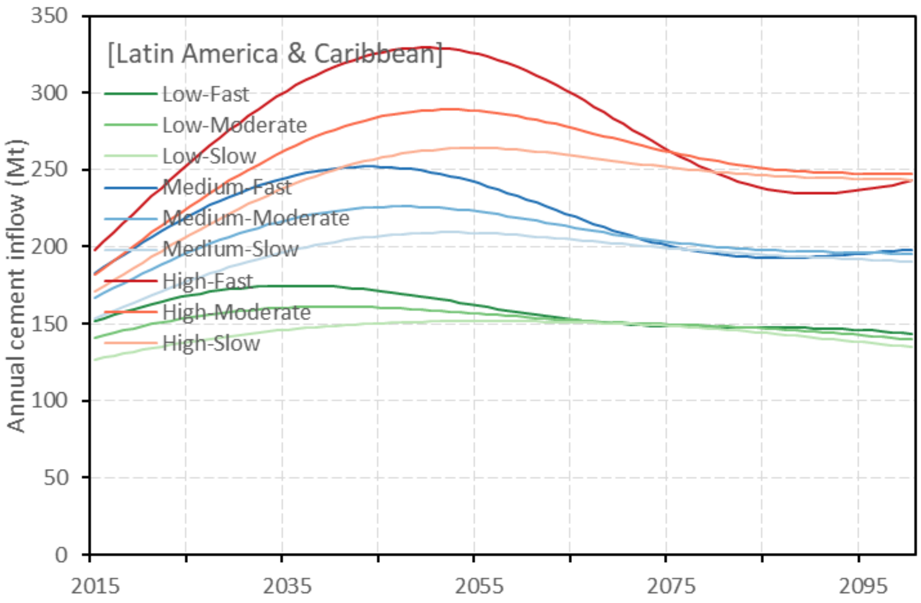

Supplementary Figure 14 | Cement inflows in Latin America & Caribbean (sectoral and total)

Supplementary Figure 15

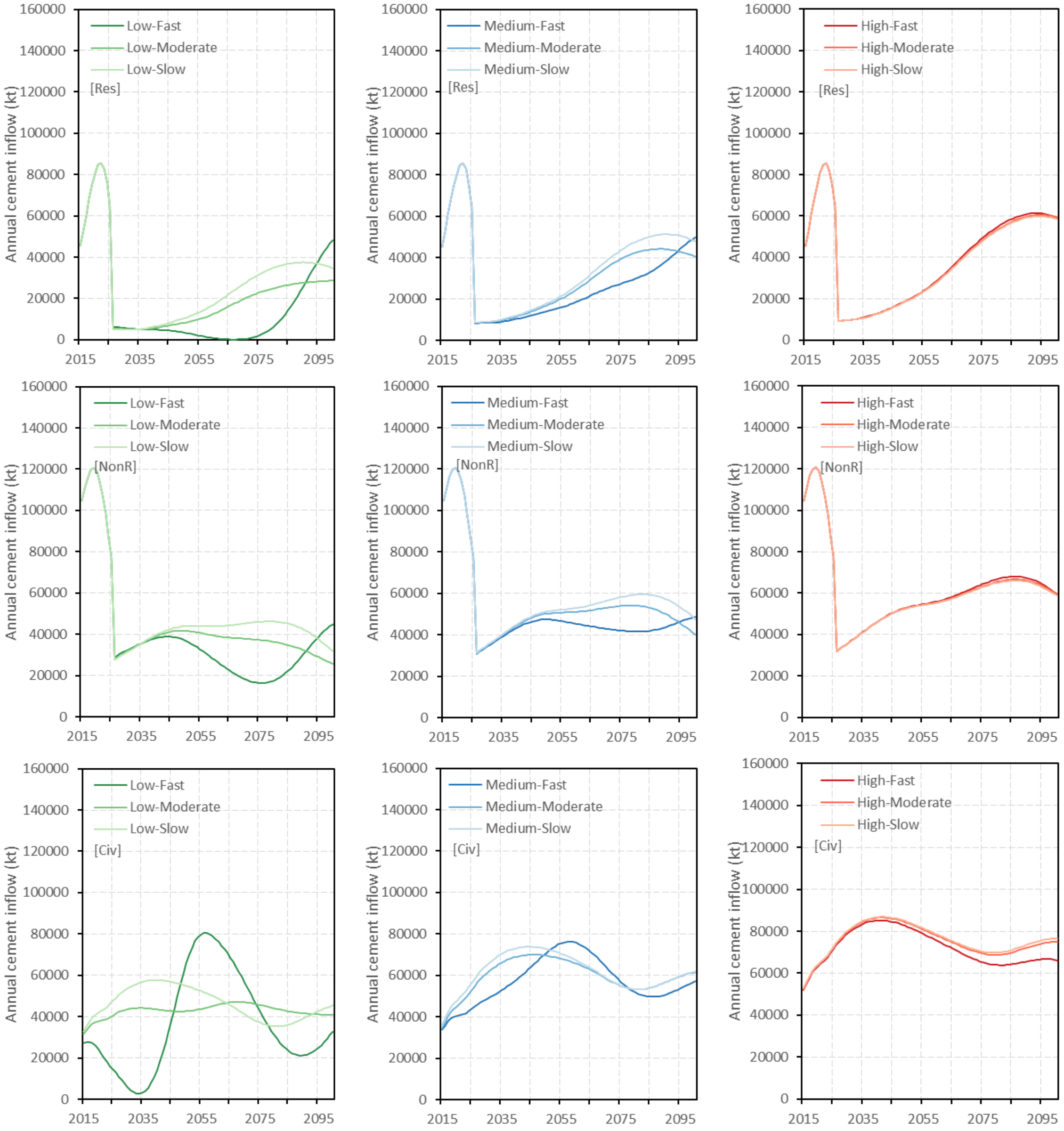

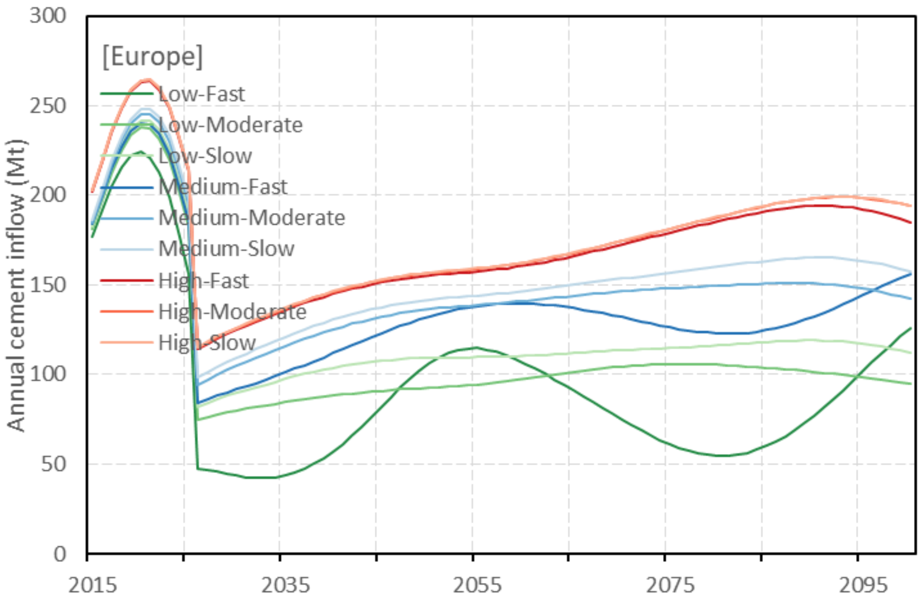

Supplementary Figure 15 | Cement inflows in Europe (sectoral and total)

Supplementary Figrue 16

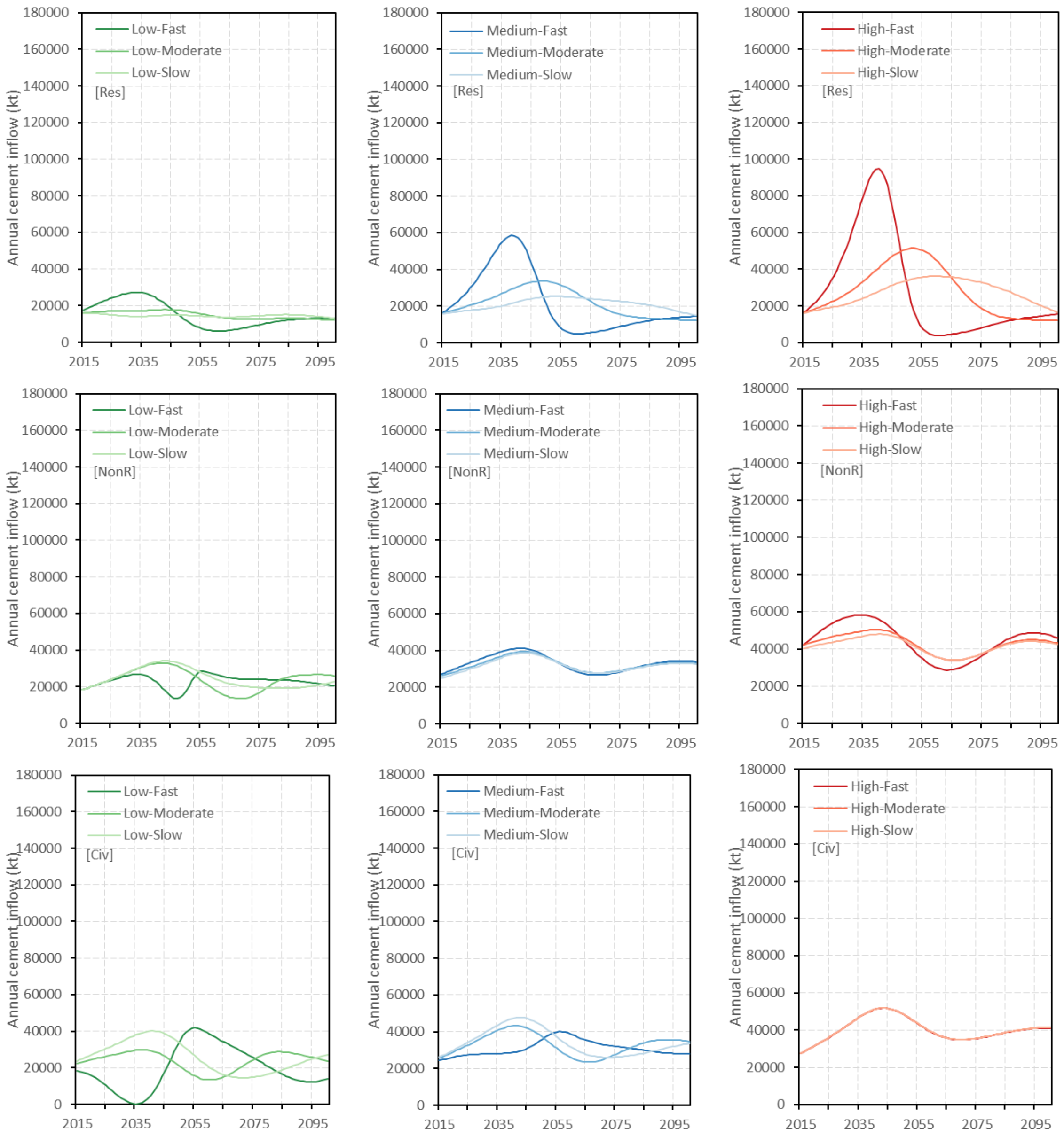

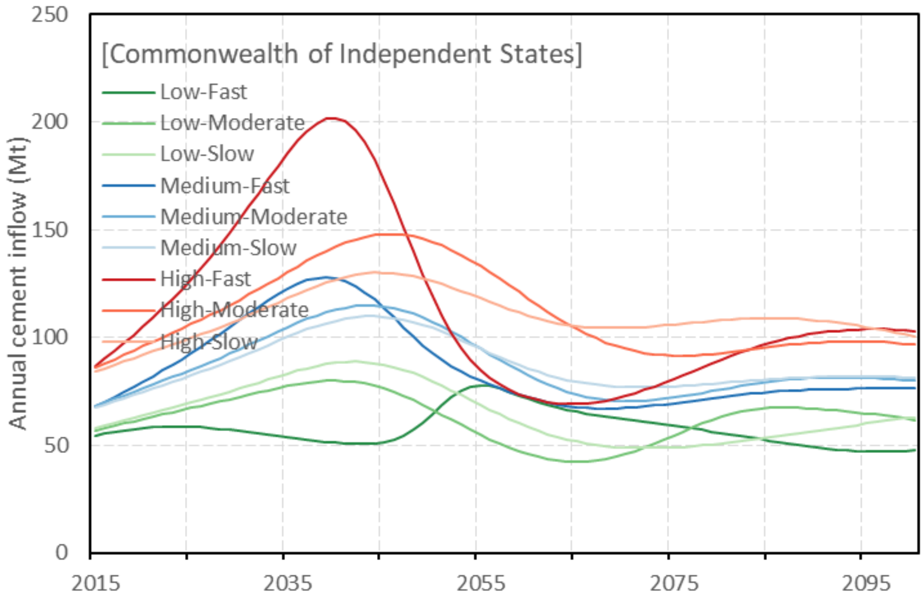

Supplementary Figure 16 | Cement inflows in Commonwealth of Independent States (sectoral and total)

Supplementary Figure 17

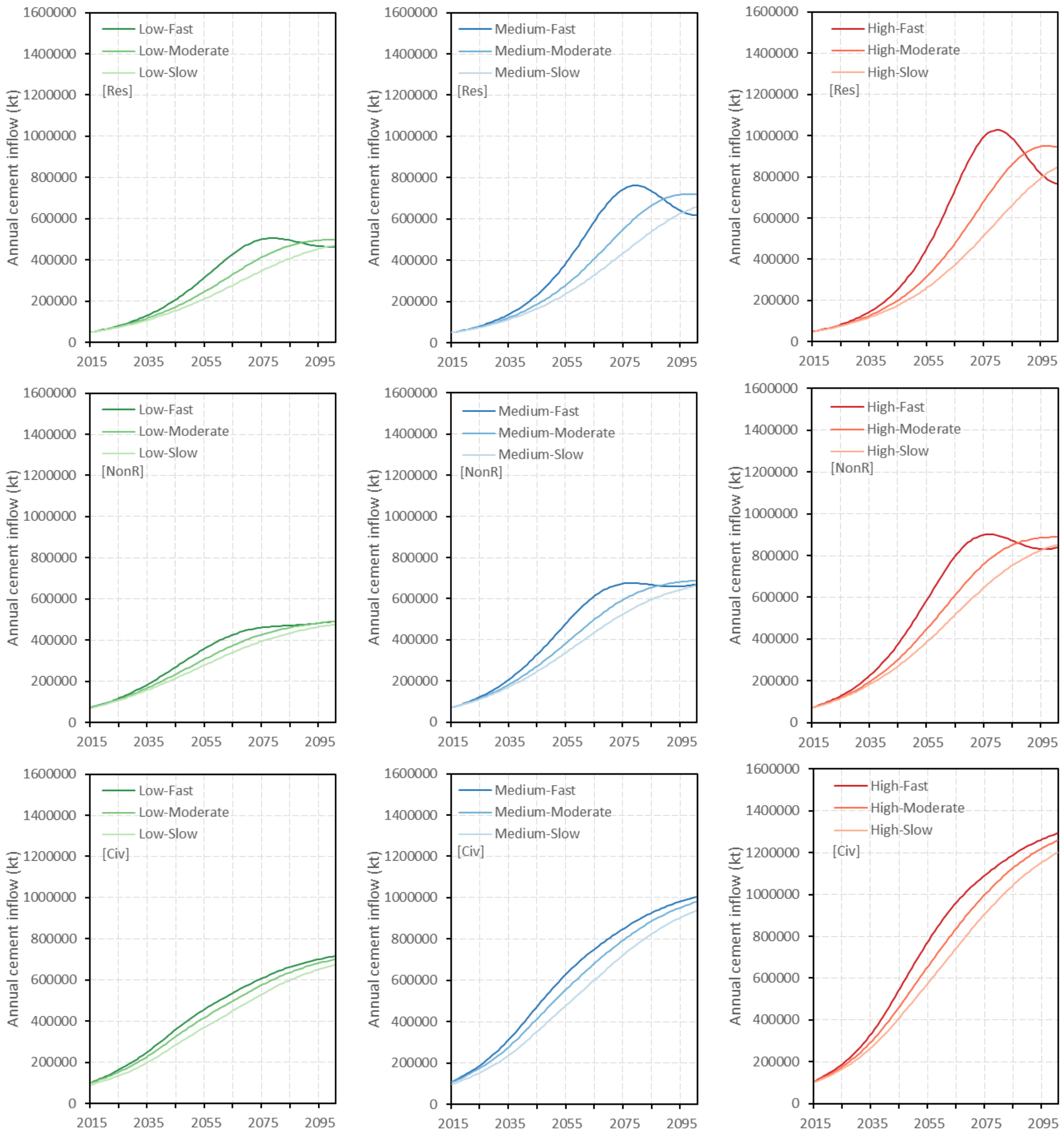

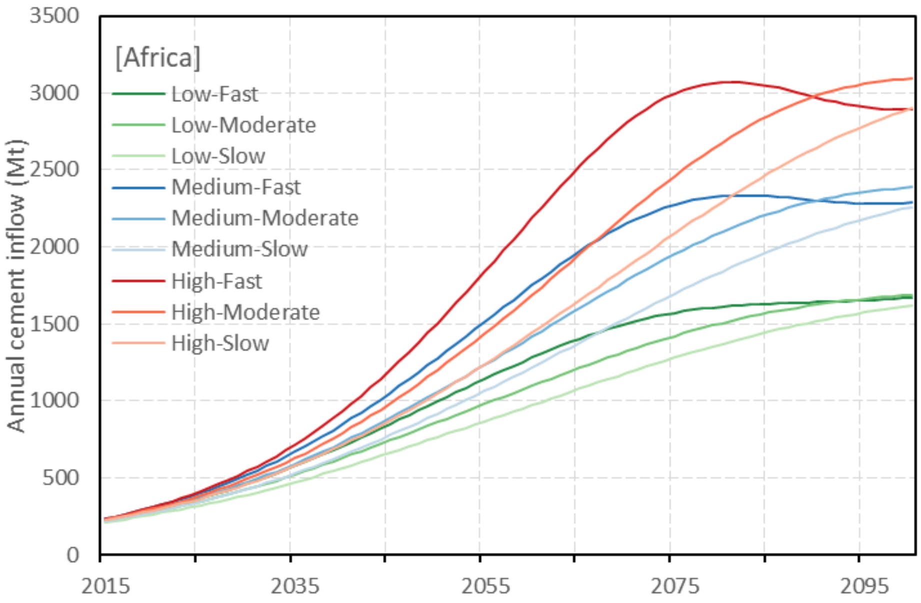

Supplementary Figure 17 | Cement inflows in Africa (sectoral and total)

Supplementary Figure 18

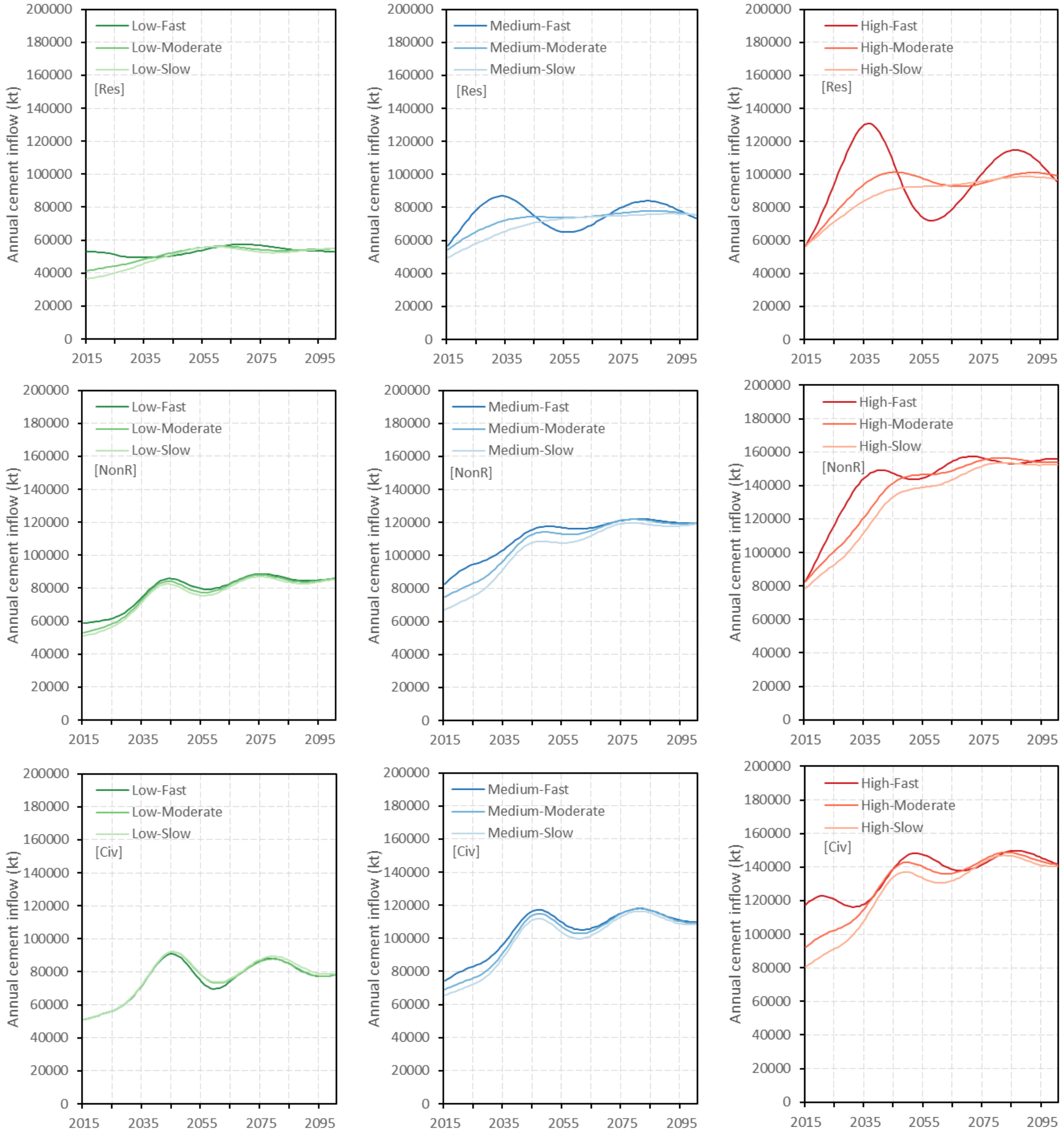

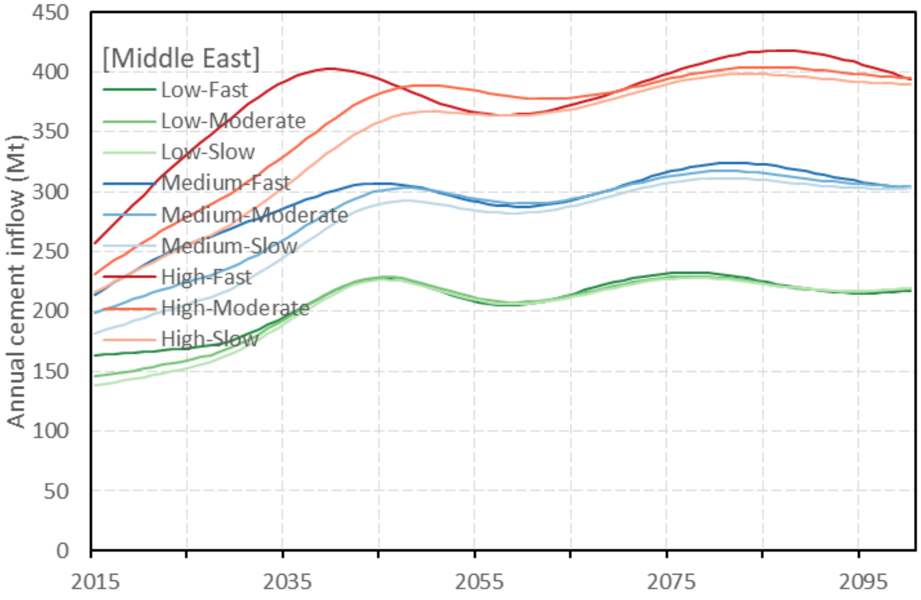

Supplementary Figure 18 | Cement inflows in Middle East (sectoral and total)

Supplementary Figure 19

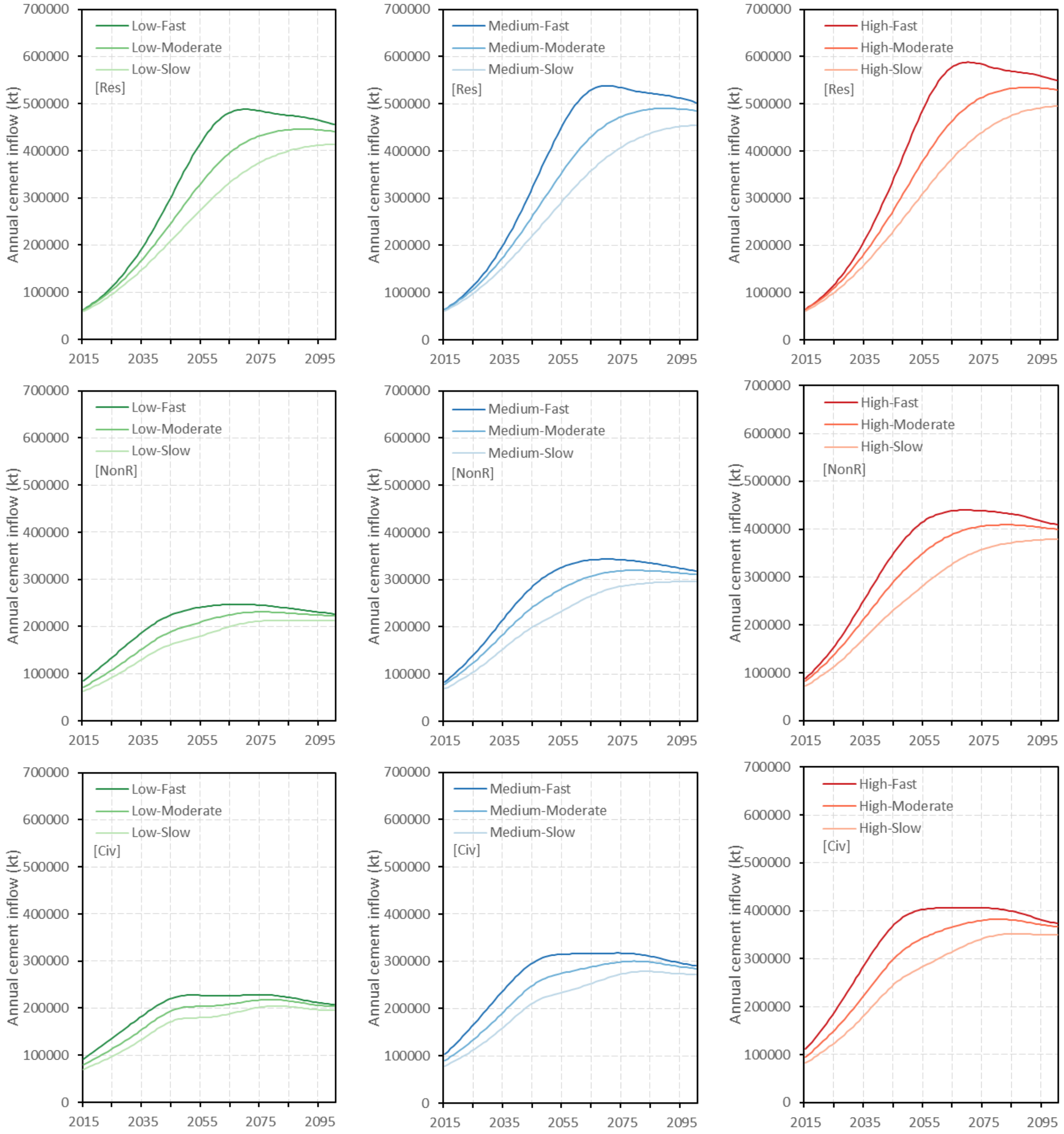

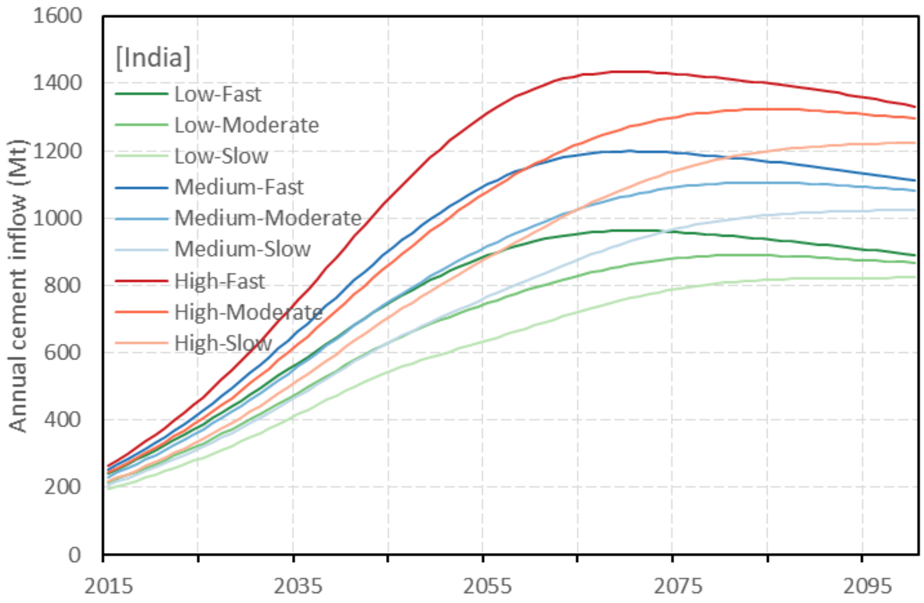

Supplementary Figure 19 | Cement inflows in India (sectoral and total)

Supplementary Figure 20

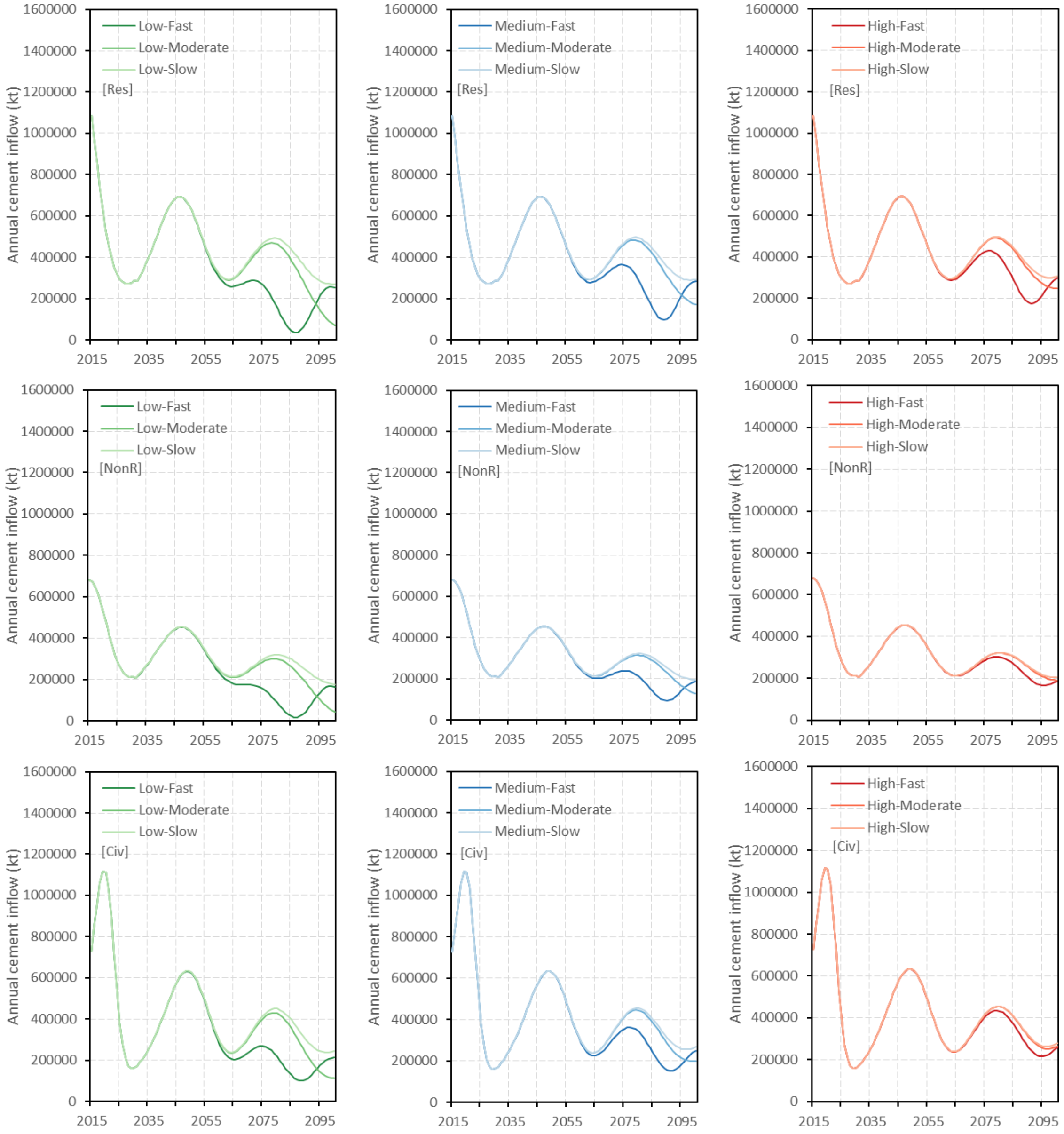

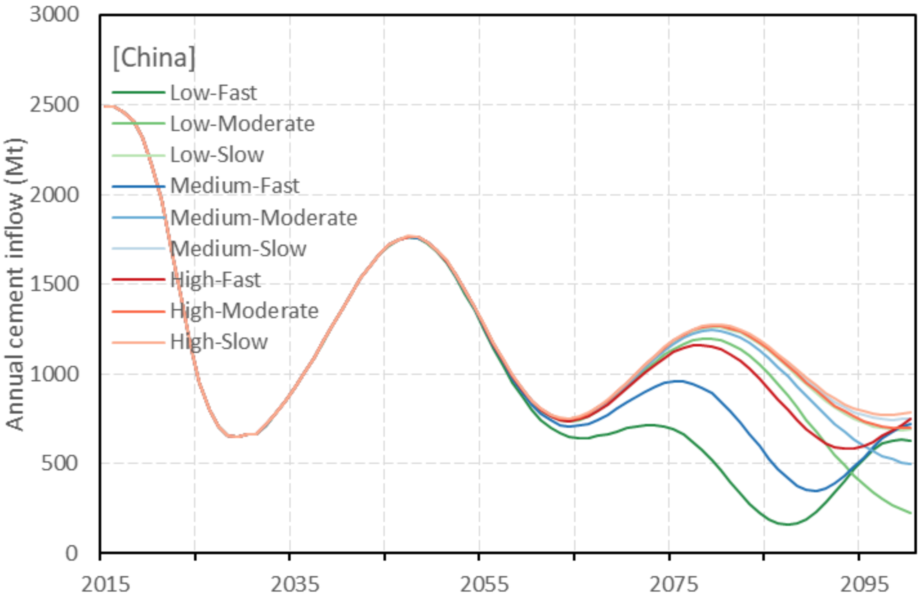

Supplementary Figure 20 | Cement inflows in China (sectoral and total)

Supplementary Figure 21

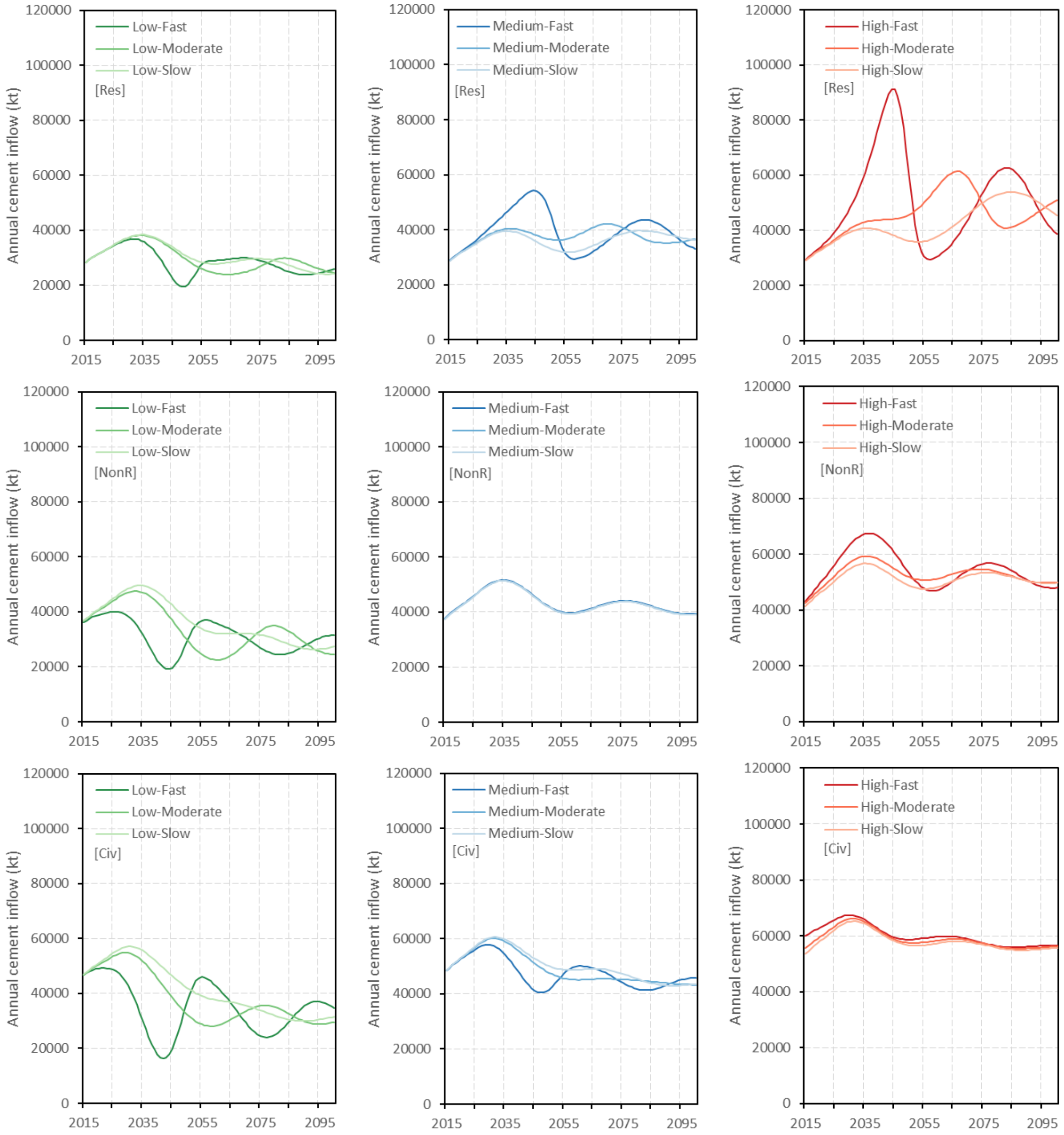

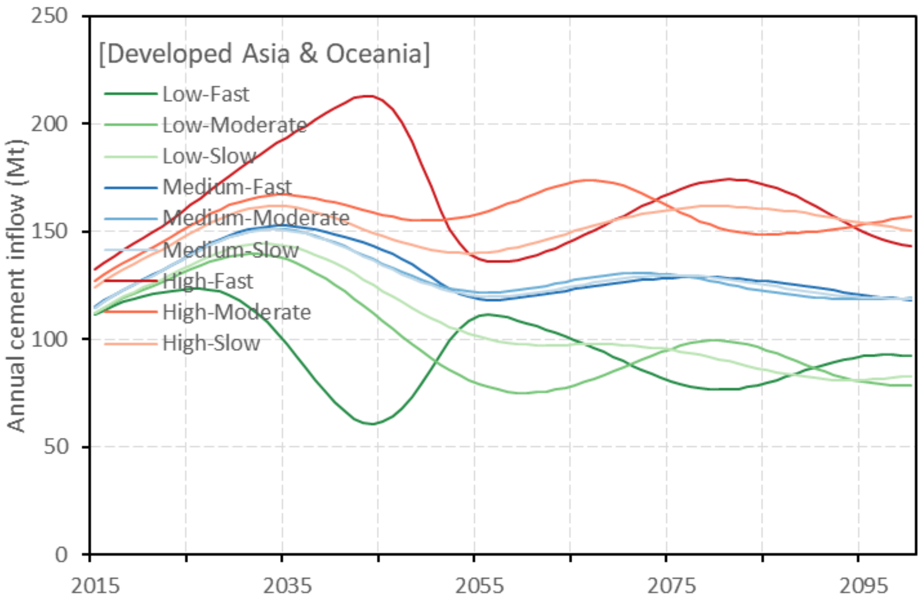

Supplementary Figure 21 | Cement inflows in Developed Asia & Oceania (sectoral and total)

Supplementary Figure 22

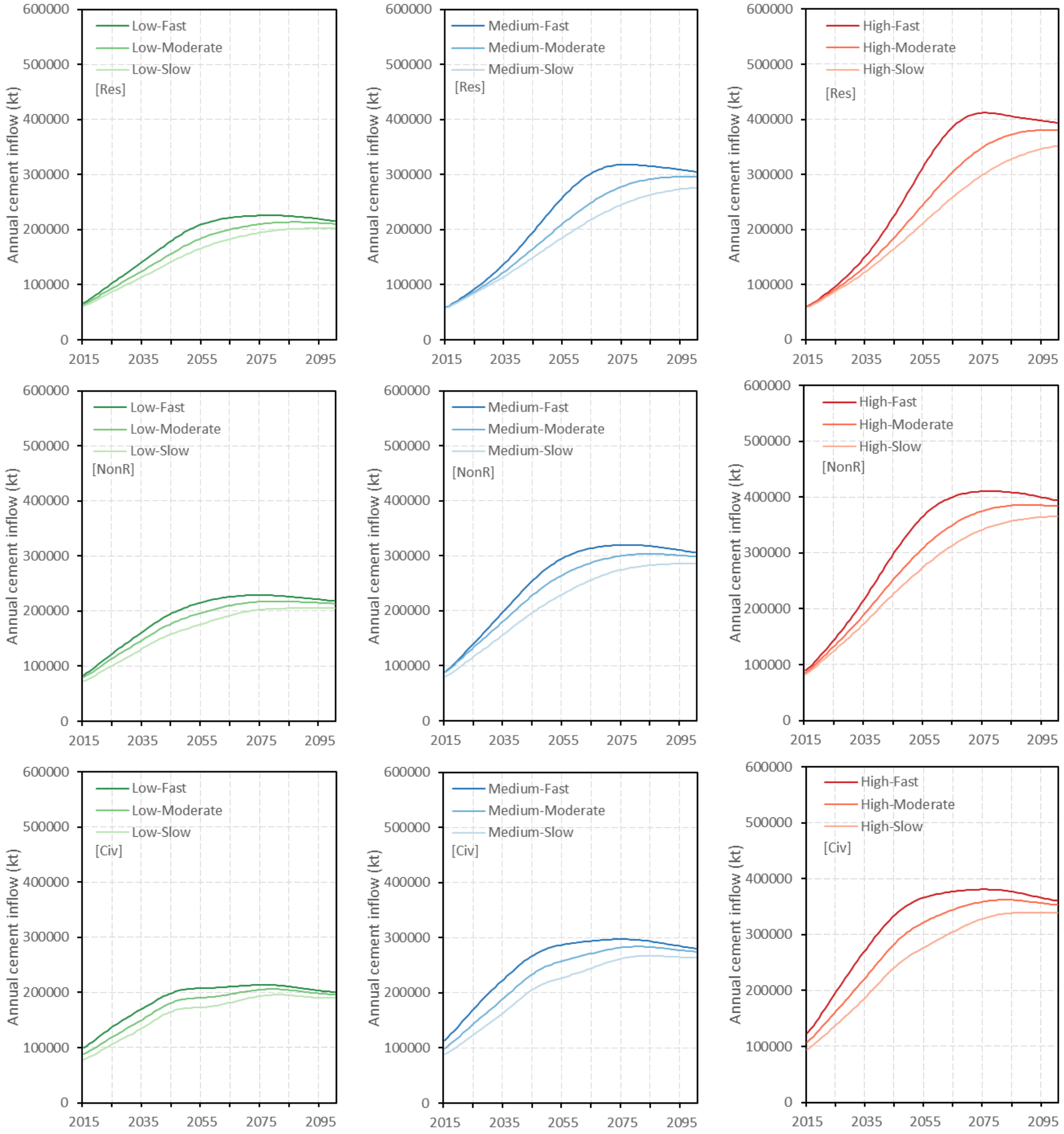

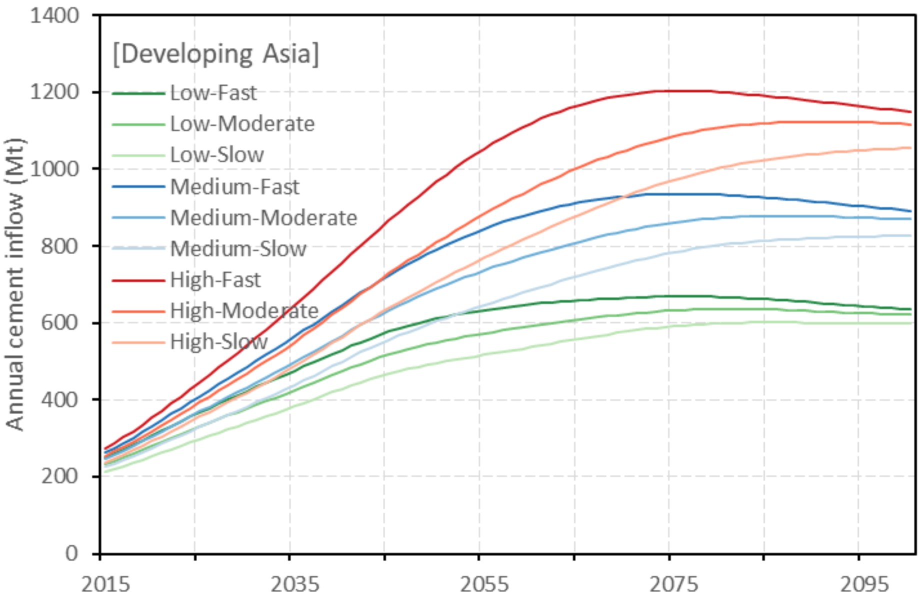

Supplementary Figure 22 | Cement inflows in Developing Asia (sectoral and total)

Supplementary Figure 23

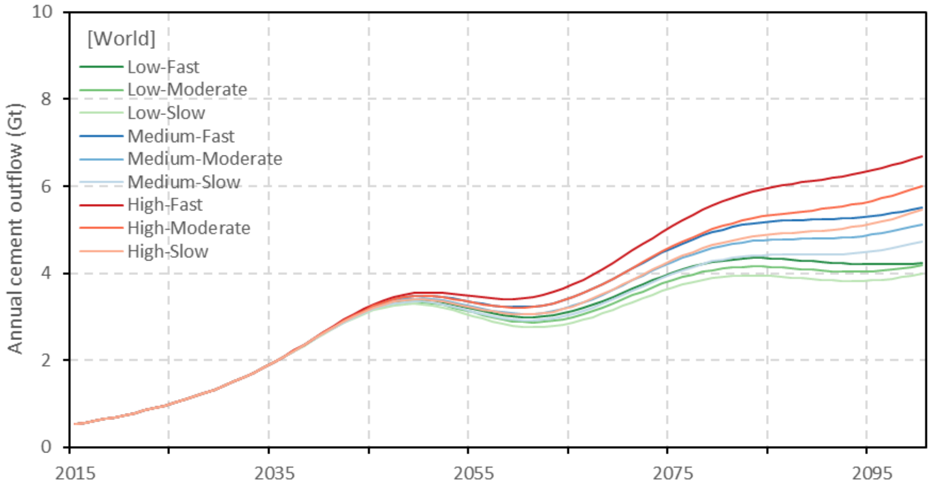

Supplementary Figure 23 | Cement in demolition waste in the world

Supplementary Figure 24

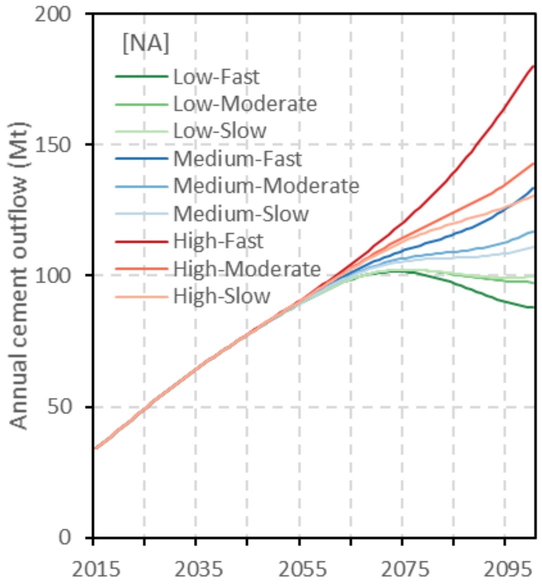

Supplementary Figure 24 | Cement in demolition waste in North America

Supplementary Figure 25

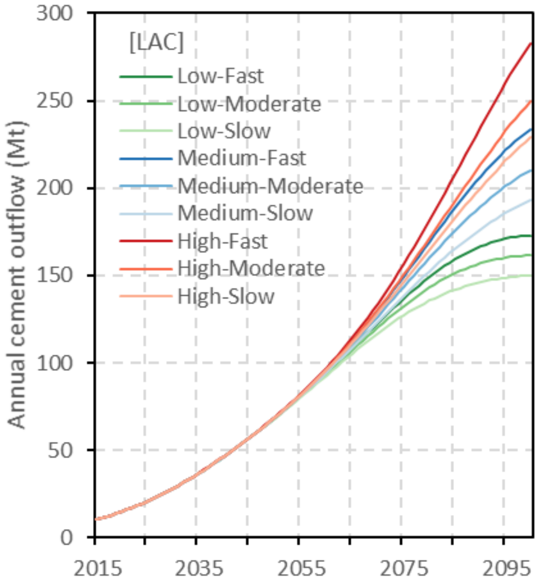

Supplementary Figure 25 | Cement in demolition waste in Latin America & Caribbean

Supplementary Figure 26

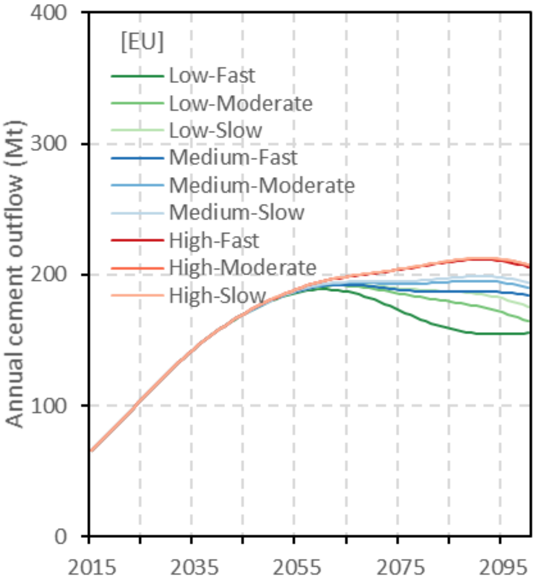

Supplementary Figure 26 | Cement in demolition waste in Europe

Supplementary Figure 27

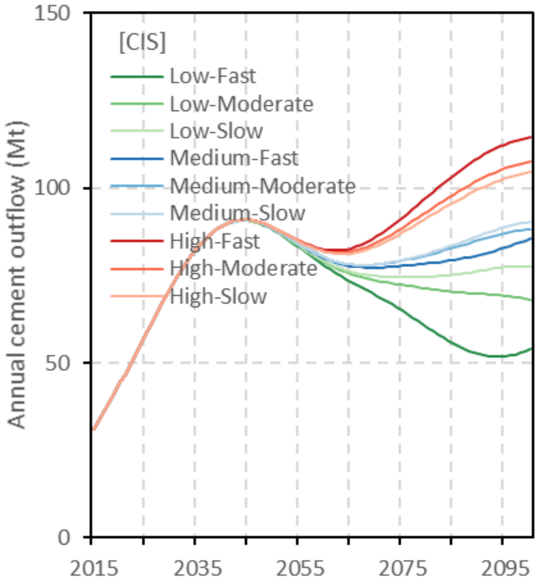

Supplementary Figure 27 | Cement in demolition waste in Commonwealth of Independent States

Supplementary Figure 28

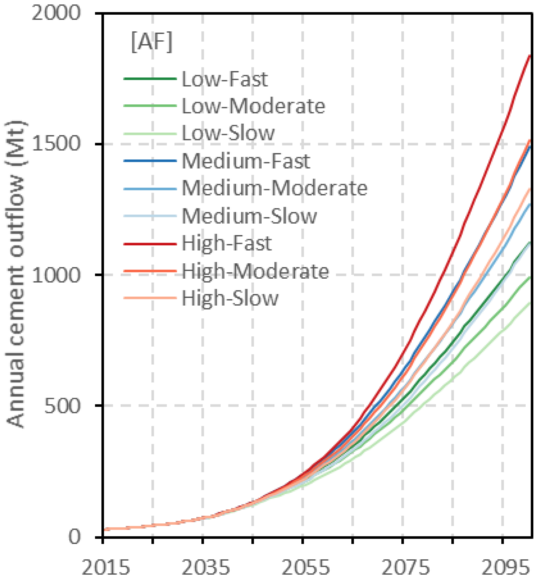

Supplementary Figure 28 | Cement in demolition waste in Arica

Supplementary Figure 29

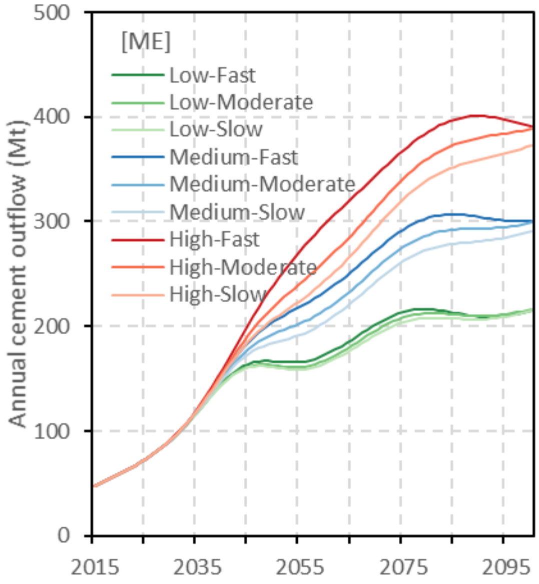

Supplementary Figure 29 | Cement in demolition waste in Middle East

Supplementary Figure 30

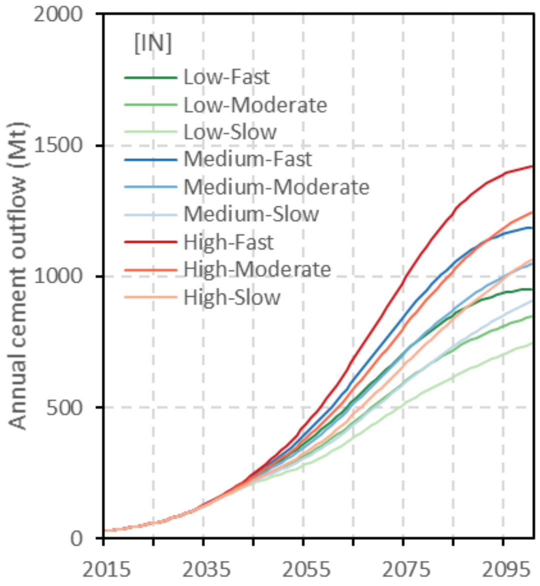

Supplementary Figure 30 | Cement in demolition waste in India

Supplementary Figure 31

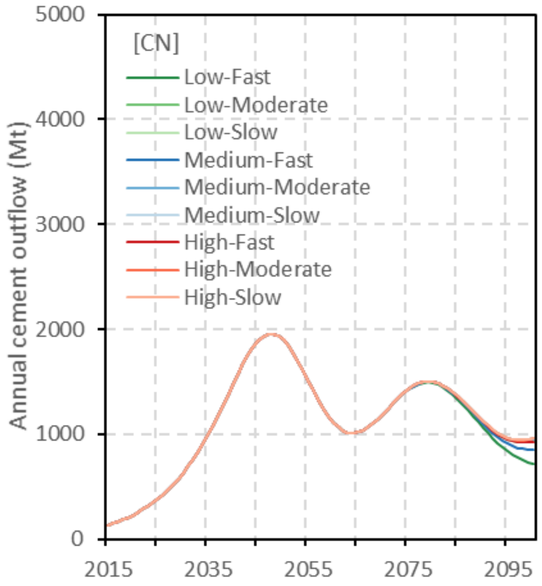

Supplementary Figure 31 | Cement in demolition waste in China

Supplementary Figure 32

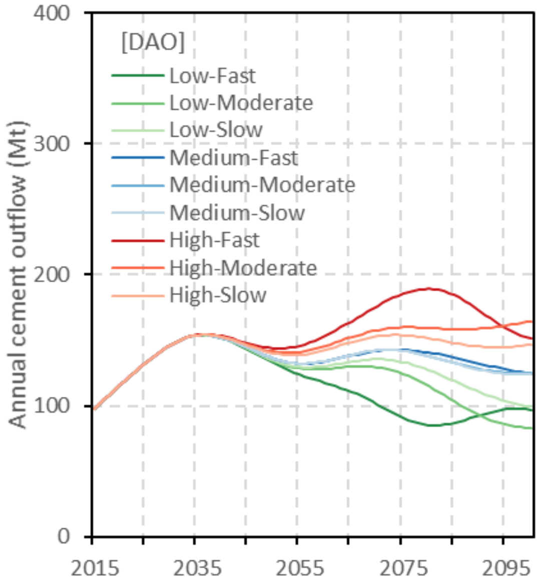

Supplementary Figure 32 | Cement in demolition waste in Developed Asia & Oceania

Supplementary Figure 33

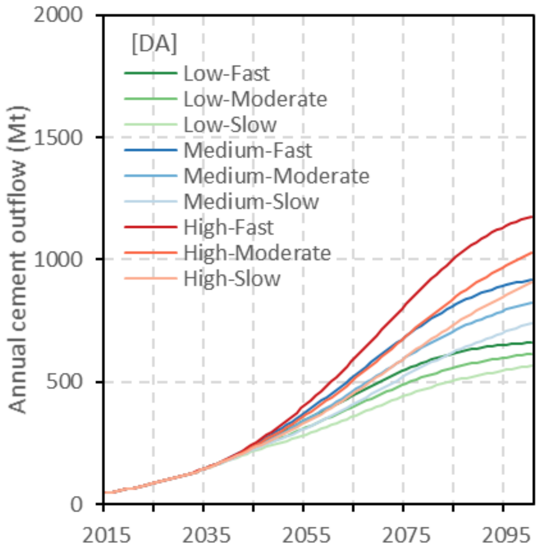

Supplementary Figure 33 | Cement in demolition waste in Developing Asia

Supplementary Figure 34

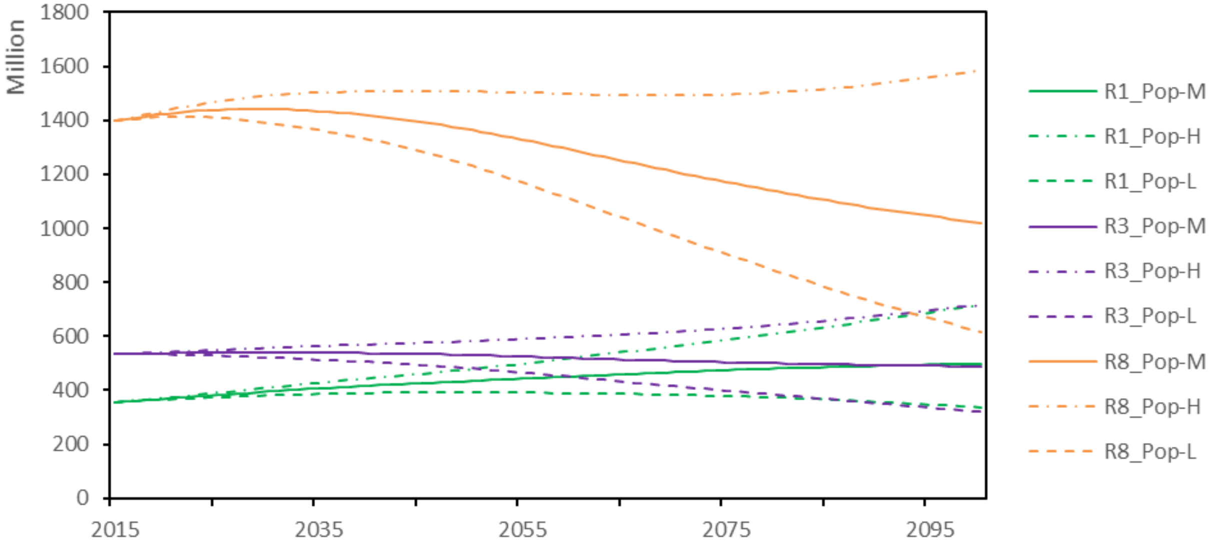

Supplementary Figure 34 | United Nations population forecast of North America (R1), Europe (R3), and China (R8) up to 2100. M: medium-variant; H: high-variant; L: low-variant.

Supplementary Figure 35

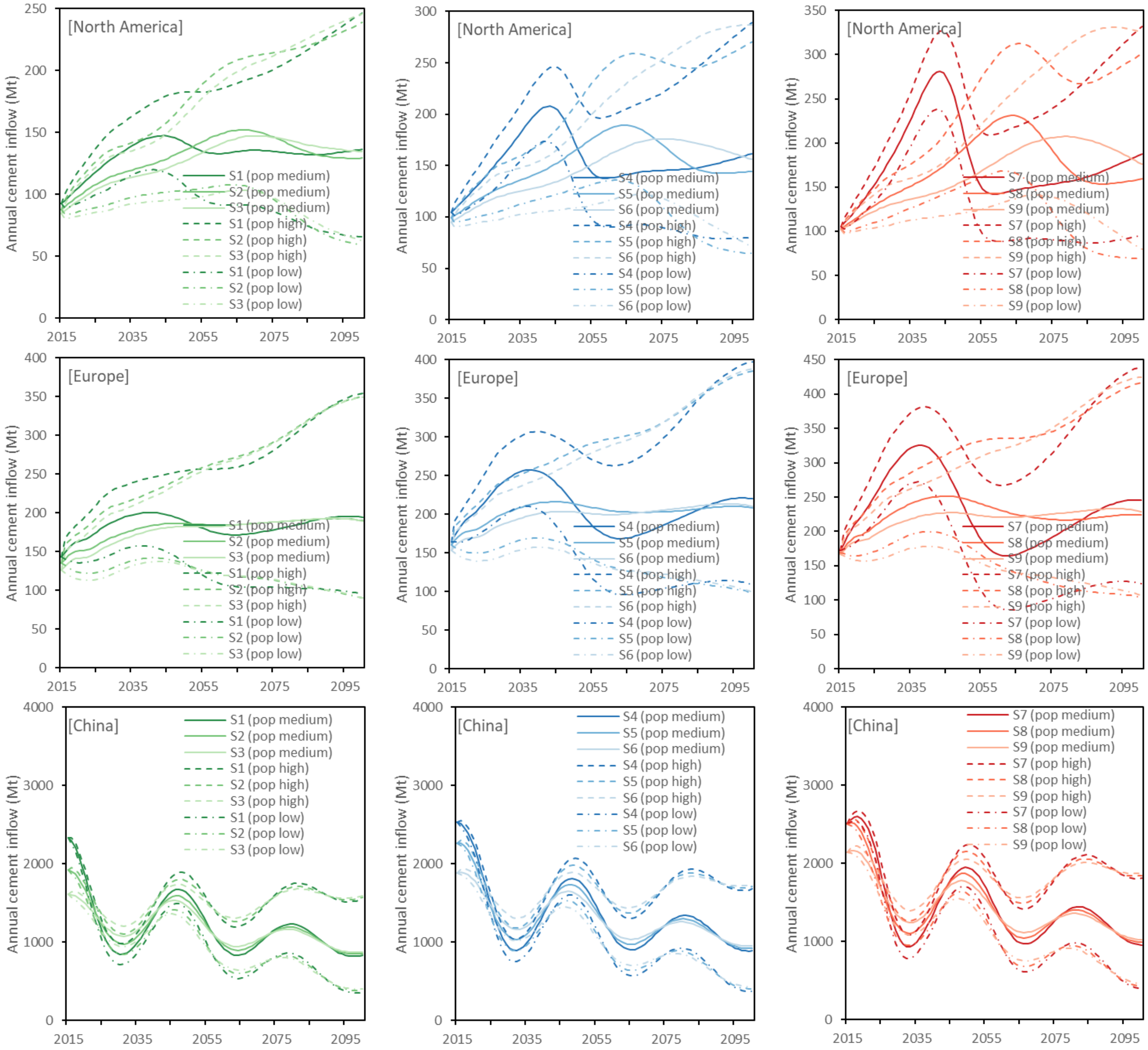

Supplementary Figure 35 | Annual cement inflows, if population is based on the medium-variant scenario, the high-variant scenario, or the low-variant scenario

Supplementary Figure 36

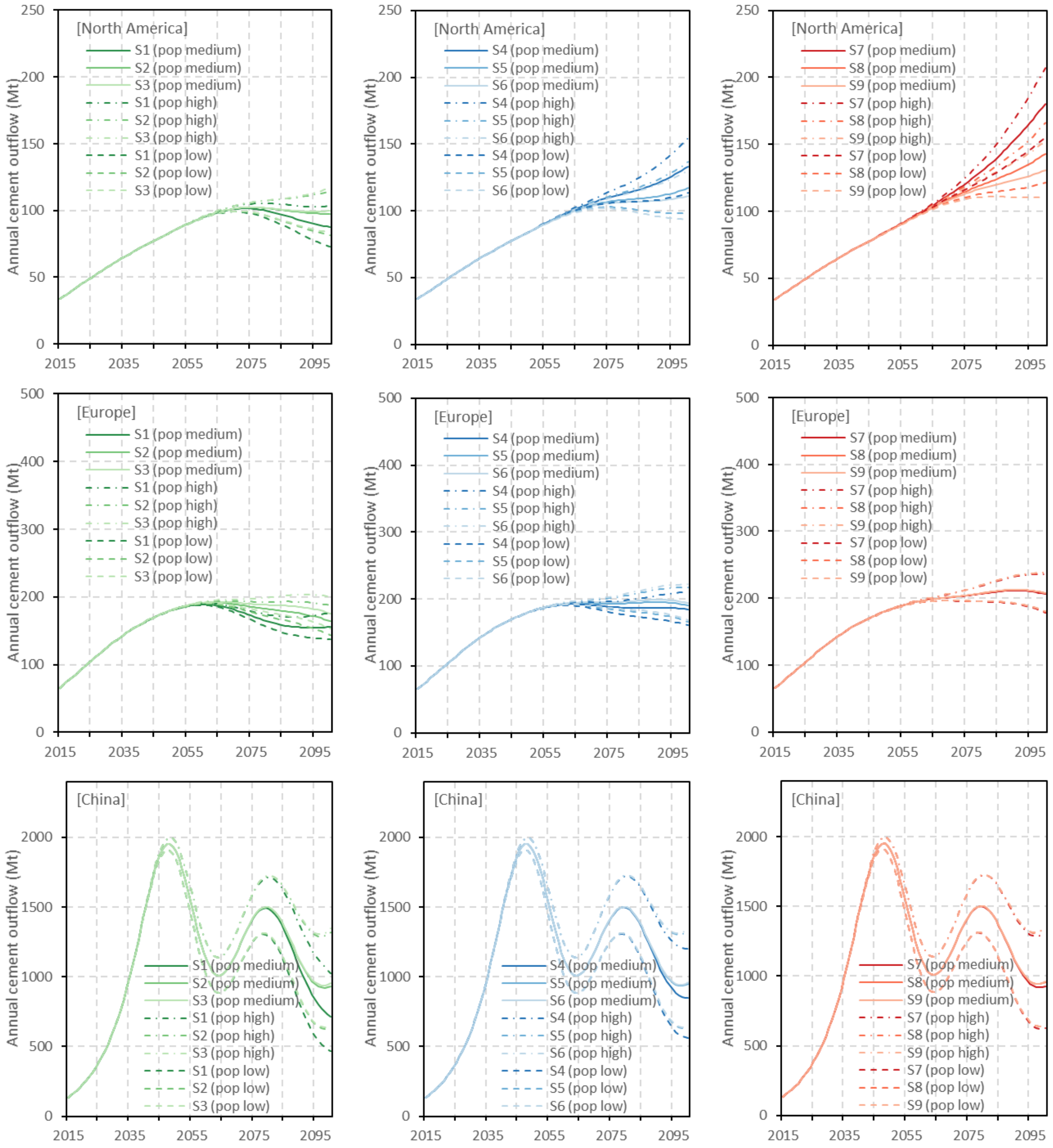

Supplementary Figure 36 | Annual cement outflows, if population is based on the medium-variant scenario, the high-variant scenario, or the low-variant scenario

Supplementary Figure 37

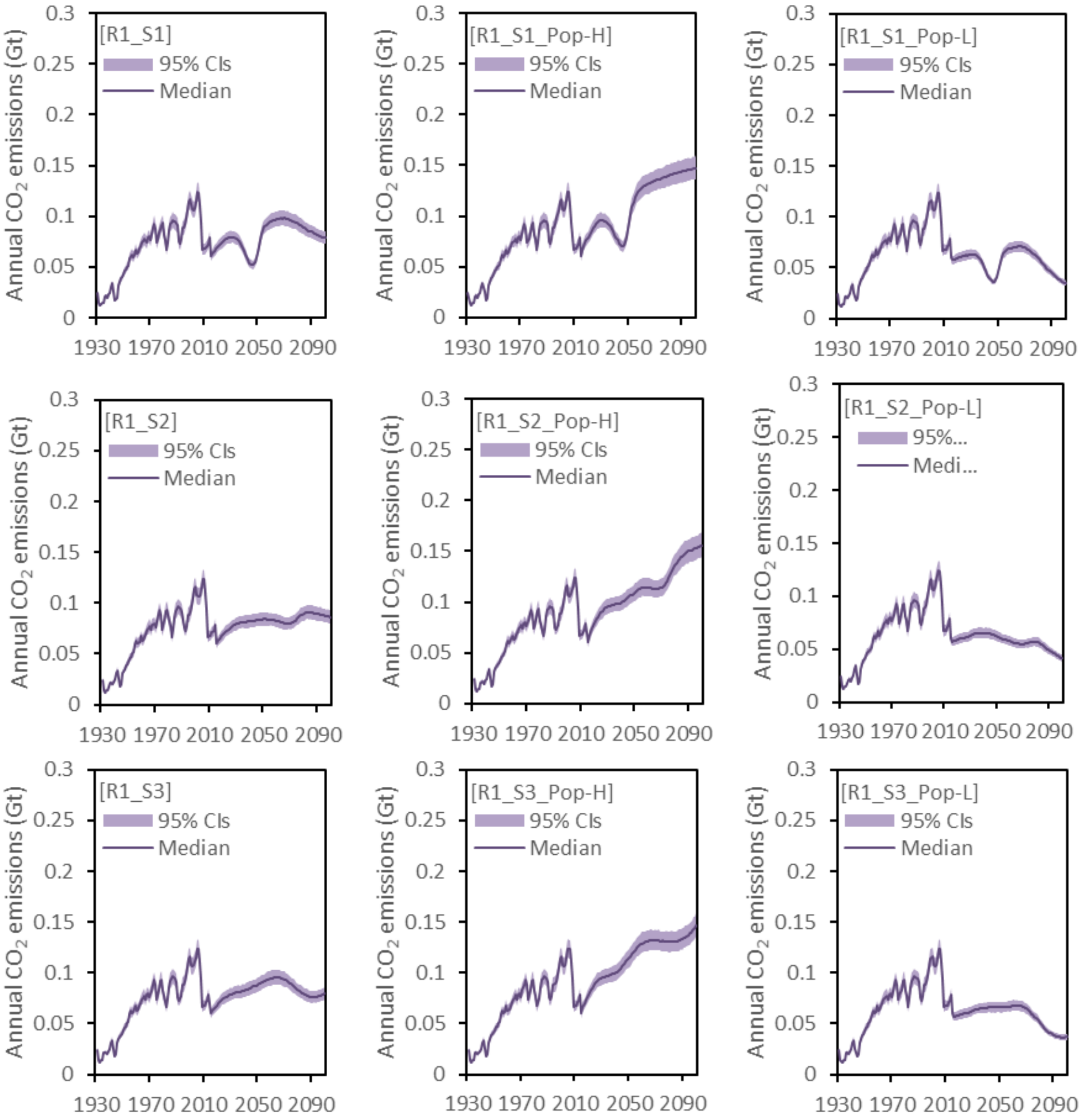

Supplementary Figure 37 | Annual CO_2 emissions in North America under scenarios S1, S2 and S3, if population is based on the medium-variant scenario (left column), the high-variant scenario (middle column), or the low-variant scenario (right column).

Note: The solid lines are the median value of simulated outcomes, and the shaded areas represent the 95% uncertainty range of simulated outcomes. The number of simulation runs is 1,000.

Supplementary Figure 38

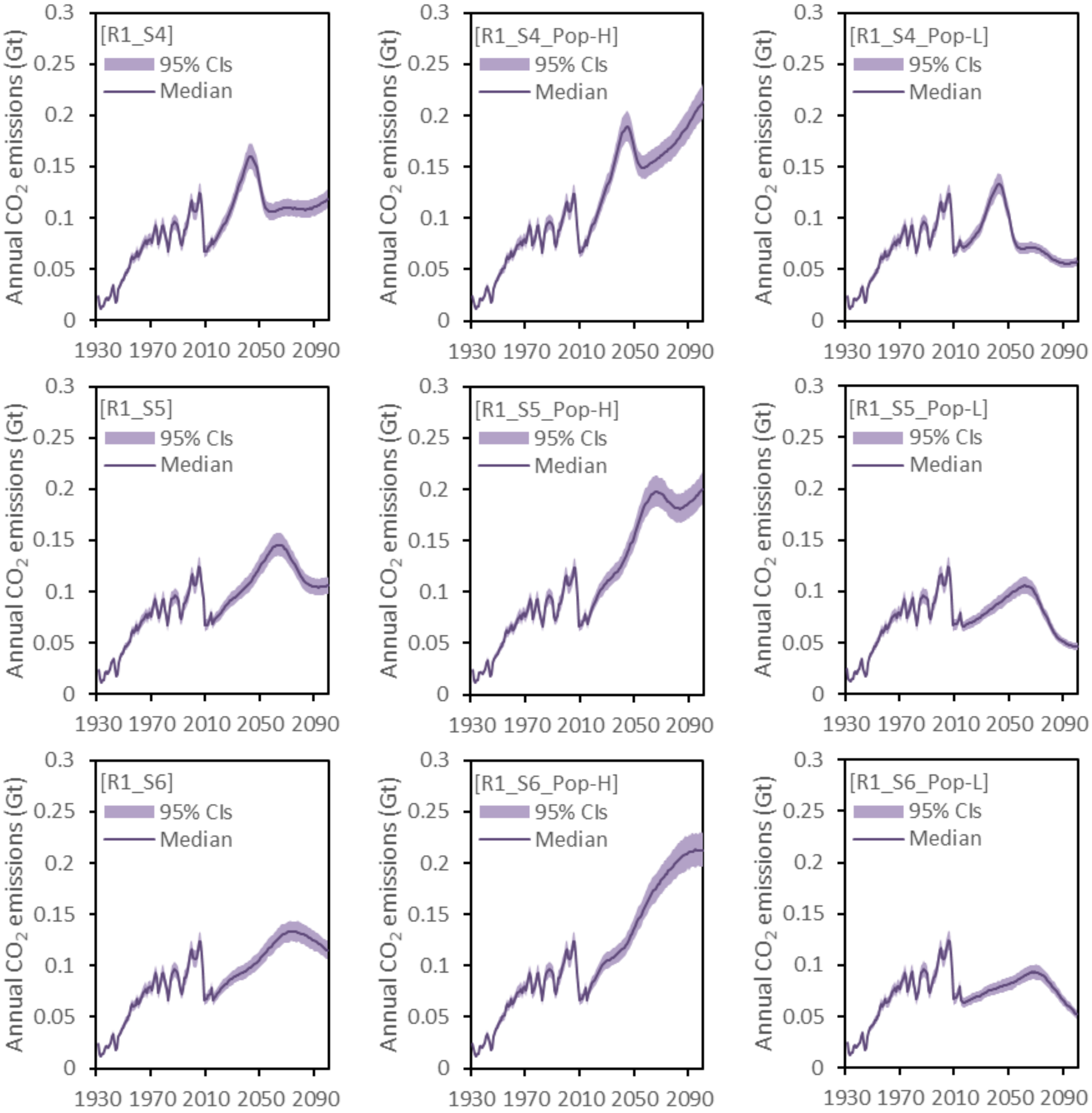

Supplementary Figure 38 | Annual CO_2 emissions in North America under scenarios S4, S5 and S6, if population is based on the medium-variant scenario (left column), the high-variant scenario (middle column), or the low-variant scenario (right column)

Note: The solid lines are the median value of simulated outcomes, and the shaded areas represent the 95% uncertainty range of simulated outcomes. The number of simulation runs is 1,000.

Supplementary Figure 39

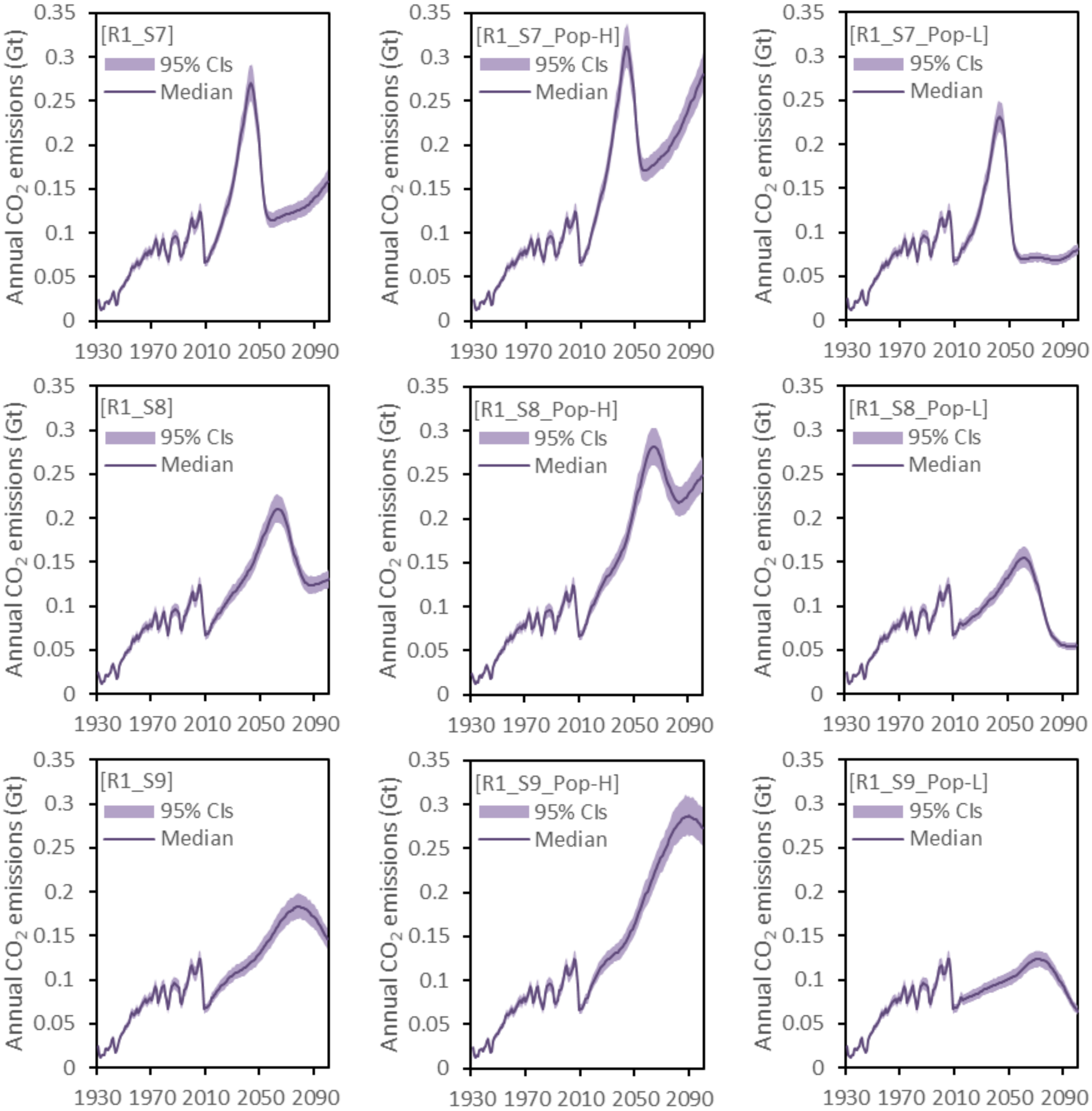

Supplementary Figure 39 | Annual CO_2 emissions in North America under scenarios S7, S8 and S9, if population is based on the medium-variant scenario (left column), the high-variant scenario (middle column), or the low-variant scenario (right column)

Note: The solid lines are the median value of simulated outcomes, and the shaded areas represent the 95% uncertainty range of simulated outcomes. The number of simulation runs is 1,000.

Supplementary Figure 40

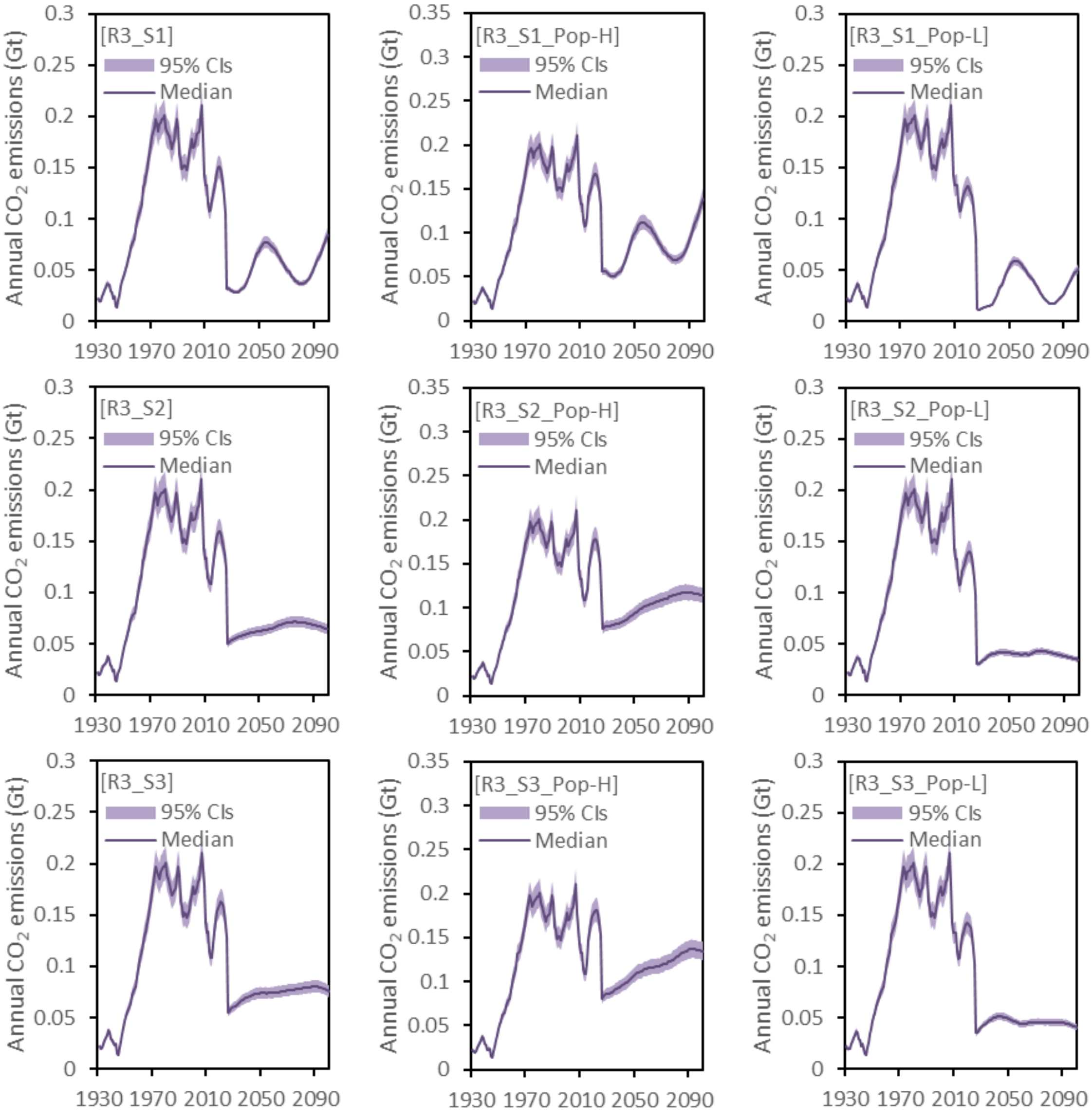

Supplementary Figure 40 | Annual CO_2 emissions in Europe under scenarios S1, S2 and S3, if population is based on the medium-variant scenario (left column), the high-variant scenario (middle column), or the low-variant scenario (right column)

Note: The solid lines are the median value of simulated outcomes, and the shaded areas represent the 95% uncertainty range of simulated outcomes. The number of simulation runs is 1,000.

Supplementary Figure 41

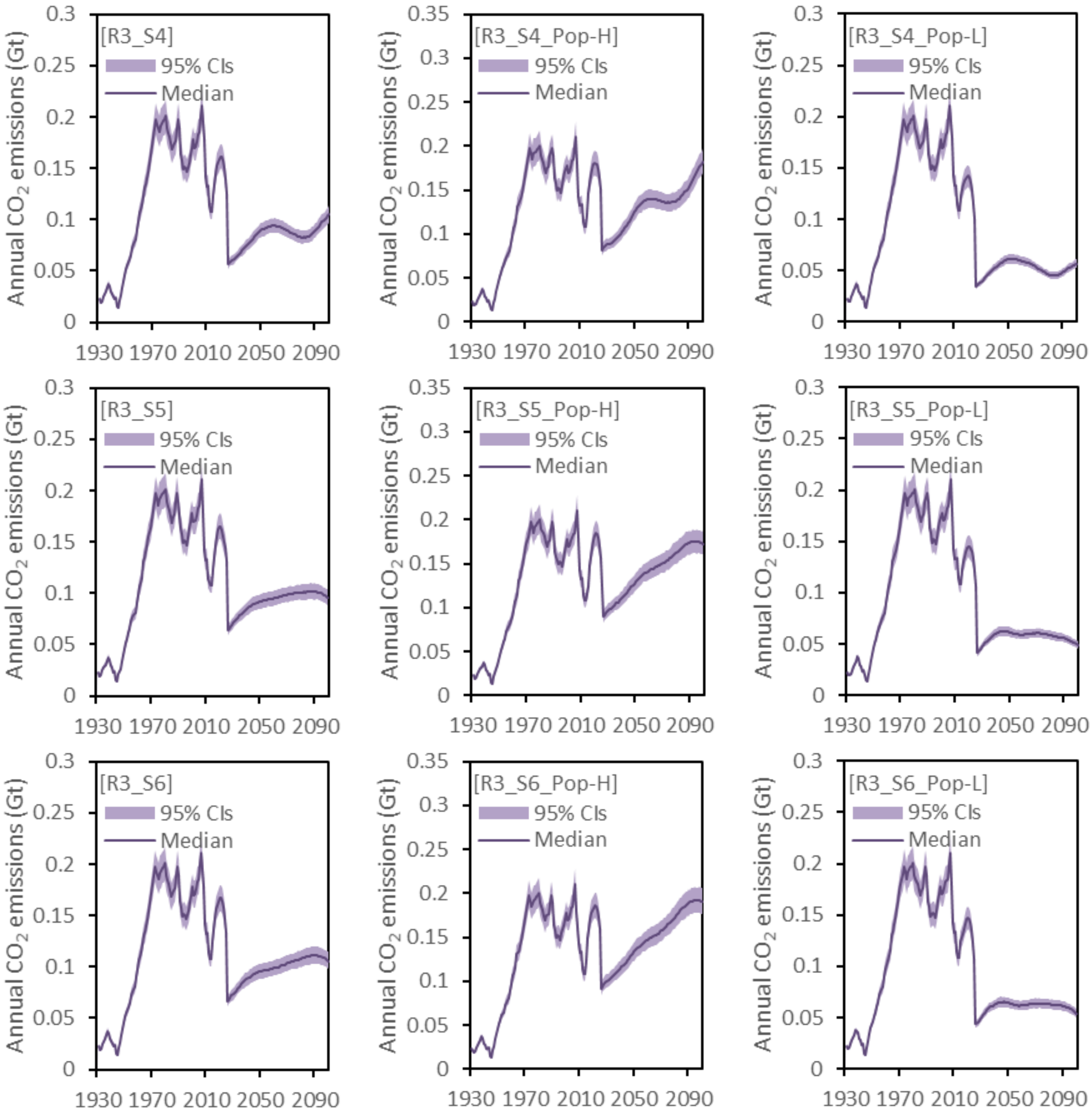

Supplementary Figure 41 | Annual CO_2 emissions in Europe under scenarios S4, S5 and S6, if population is based on the medium-variant scenario (left column), the high-variant scenario (middle column), or the low-variant scenario (right column)

Note: The solid lines are the median value of simulated outcomes, and the shaded areas represent the 95% uncertainty range of simulated outcomes. The number of simulation runs is 1,000.

Supplementary Figure 42

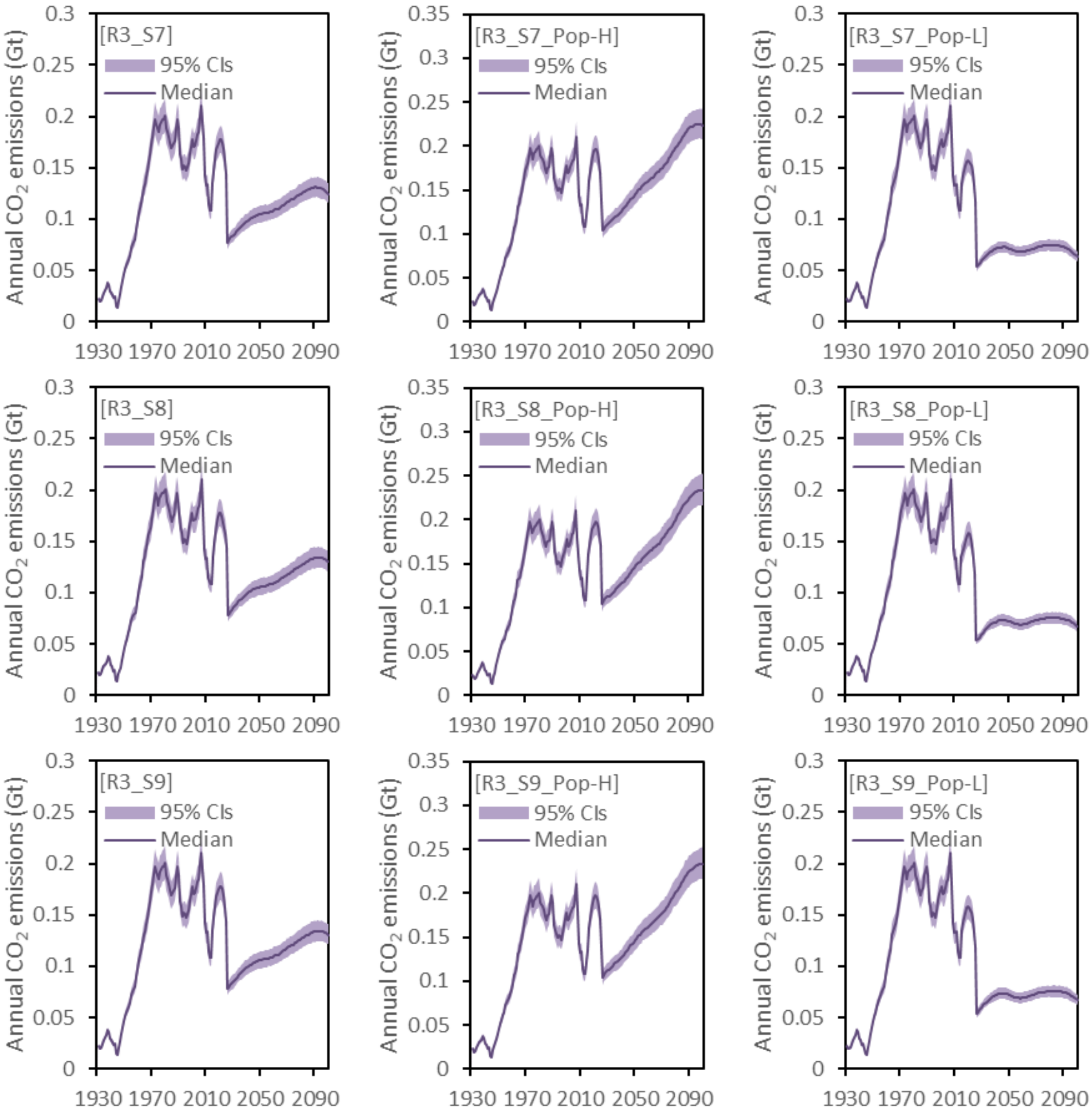

Supplementary Figure 42 | Annual CO_2 emissions in Europe under scenarios S7, S8 and S9, if population is based on the medium-variant scenario (left column), the high-variant scenario (middle column), or the low-variant scenario (right column)

Note: The solid lines are the median value of simulated outcomes, and the shaded areas represent the 95% uncertainty range of simulated outcomes. The number of simulation runs is 1,000.

Supplementary Figure 43

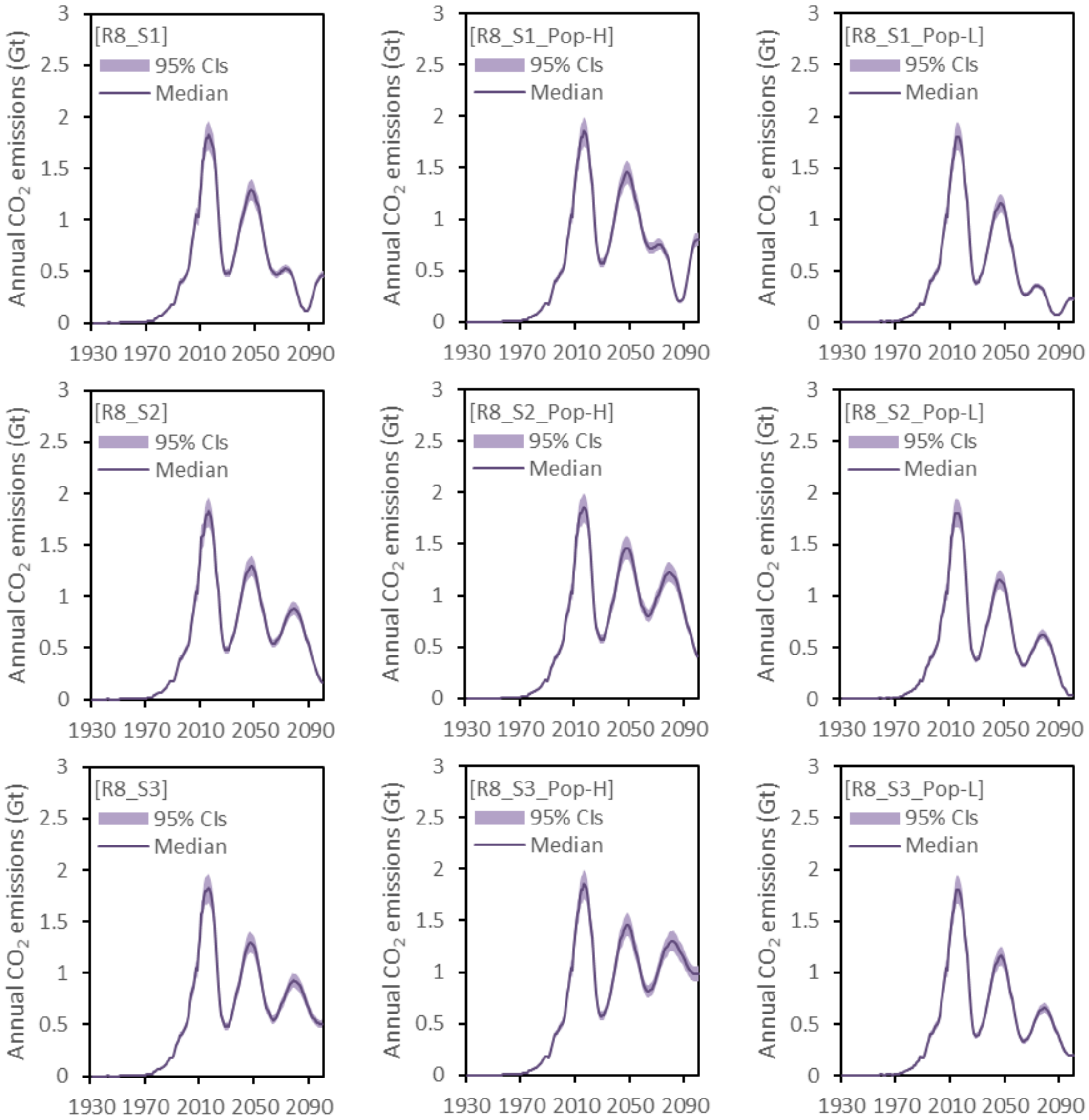

Supplementary Figure 43 | Annual CO_2 emissions in China under scenarios S1, S2 and S3, if population is based on the medium-variant scenario (left column), the high-variant scenario (middle column), or the low-variant scenario (right column)

Note: The solid lines are the median value of simulated outcomes, and the shaded areas represent the 95% uncertainty range of simulated outcomes. The number of simulation runs is 1,000.

Supplementary Figure 44

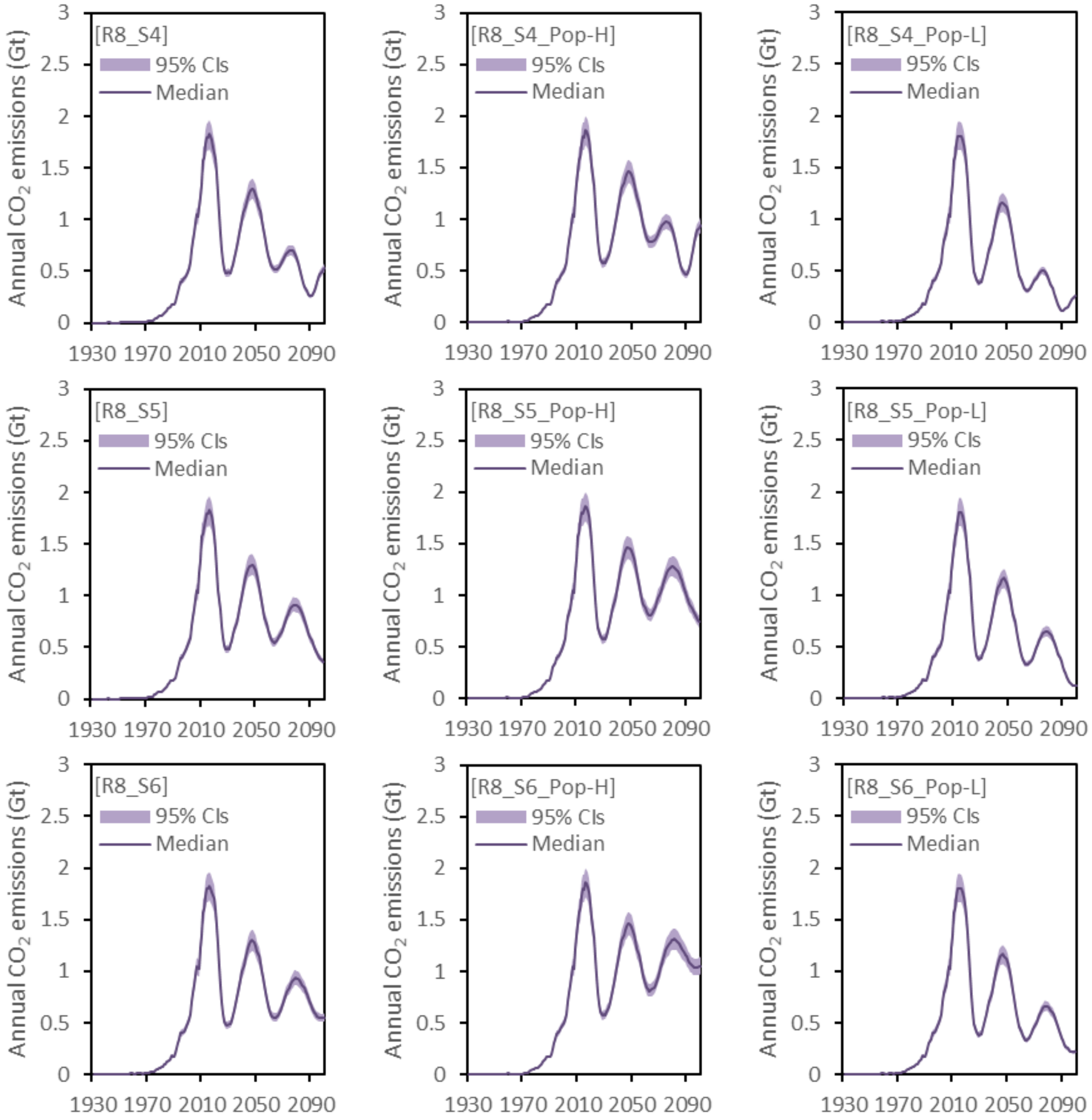

Supplementary Figure 44 | Annual CO_2 emissions in China under scenarios S4, S5 and S6, if population is based on the medium-variant scenario (left column), the high-variant scenario (middle column), or the low-variant scenario (right column)

Note: The solid lines are the median value of simulated outcomes, and the shaded areas represent the 95% uncertainty range of simulated outcomes. The number of simulation runs is 1,000.

Supplementary Figure 45

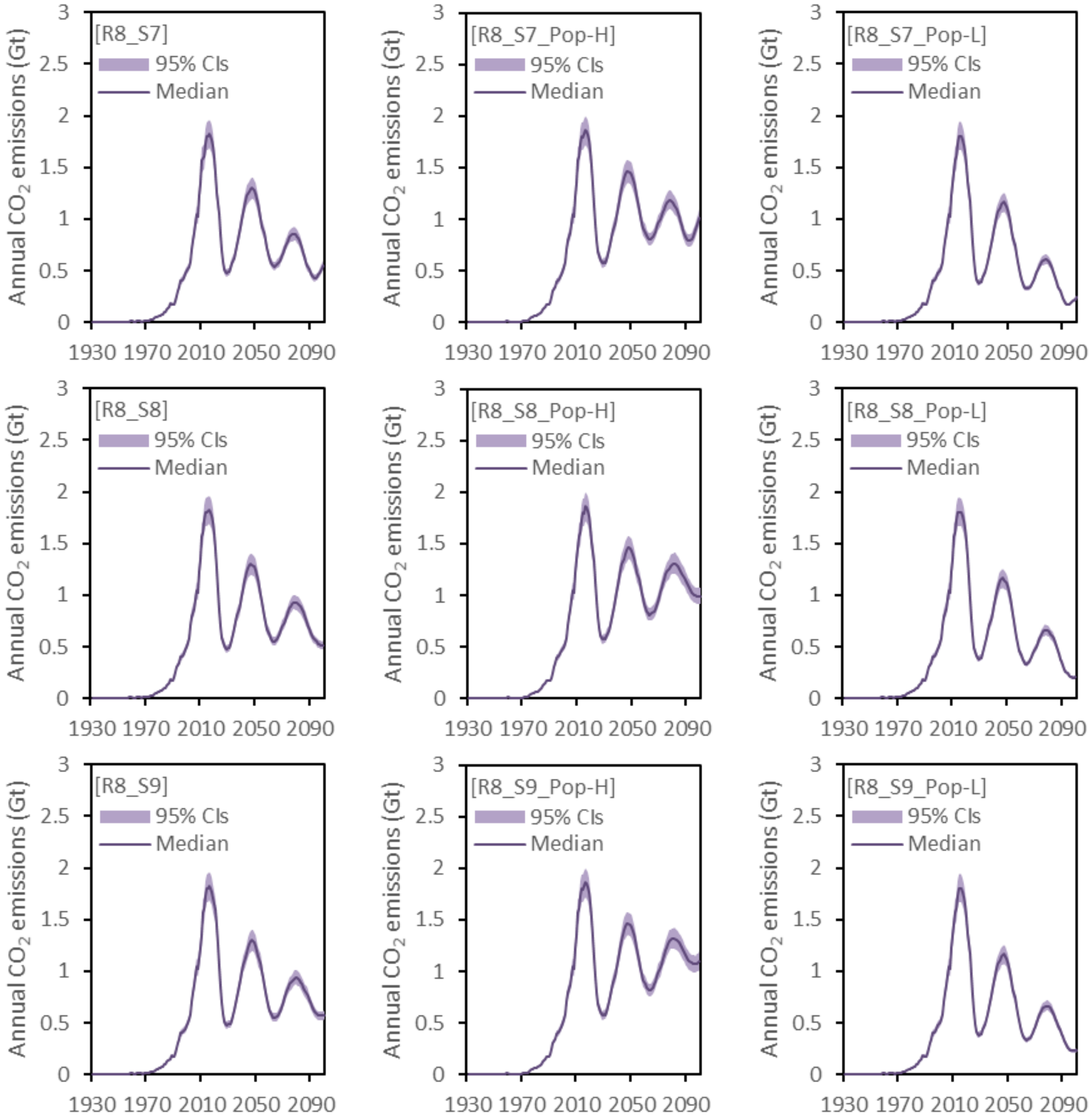

Supplementary Figure 45 | Annual CO_2 emissions in China under scenarios S7, S8 and S9, if population is based on the medium-variant scenario (left column), the high-variant scenario (middle column), or the low-variant scenario (right column)

Note: The solid lines are the median value of simulated outcomes, and the shaded areas represent the 95% uncertainty range of simulated outcomes. The number of simulation runs is 1,000.

Supplementary Figure 46

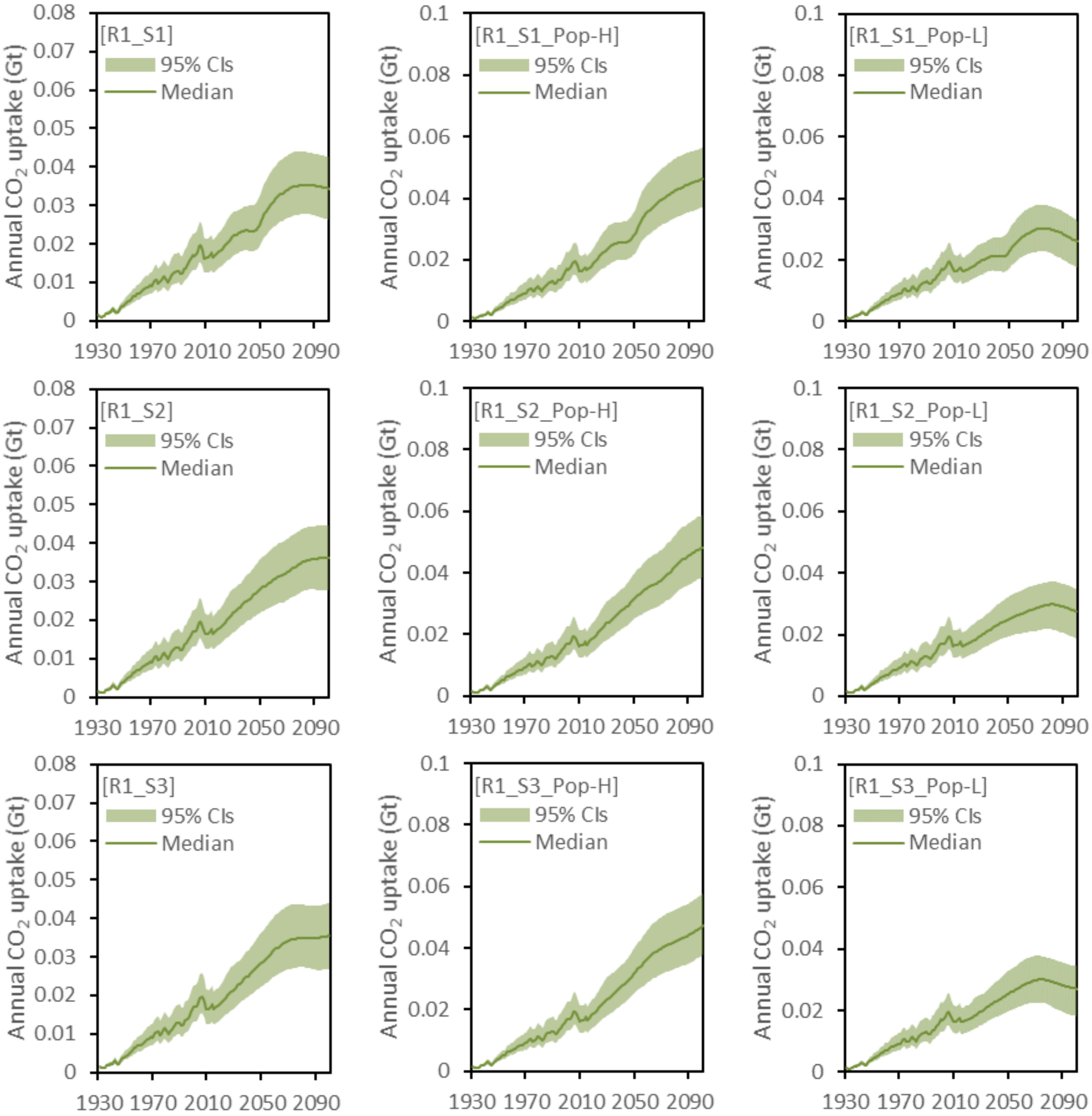

Supplementary Figure 46 | Annual CO_2 uptake in North America under scenarios S1, S2 and S3, if population is based on the medium-variant scenario (left column), the high-variant scenario (middle column), or the low-variant scenario (right column)

Note: The solid lines are the median value of simulated outcomes, and the shaded areas represent the 95% uncertainty range of simulated outcomes. The number of simulation runs is 1,000.

Supplementary Figure 47

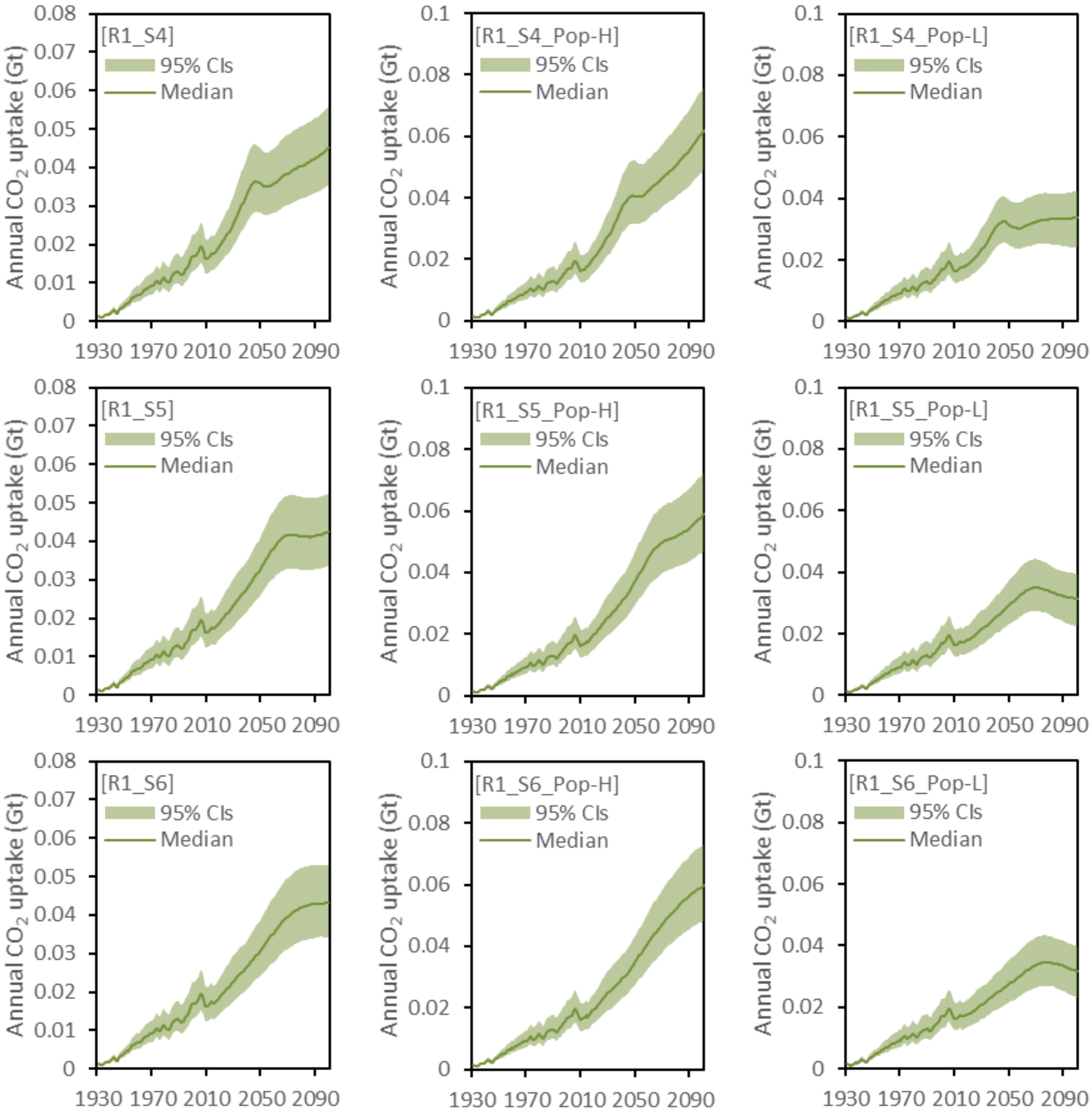

Supplementary Figure 47 | Annual CO_2 uptake in North America under scenarios S4, S5 and S6, if population is based on the medium-variant scenario (left column), the high-variant scenario (middle column), or the low-variant scenario (right column)

Note: The solid lines are the median value of simulated outcomes, and the shaded areas represent the 95% uncertainty range of simulated outcomes. The number of simulation runs is 1,000.

Supplementary Figure 48

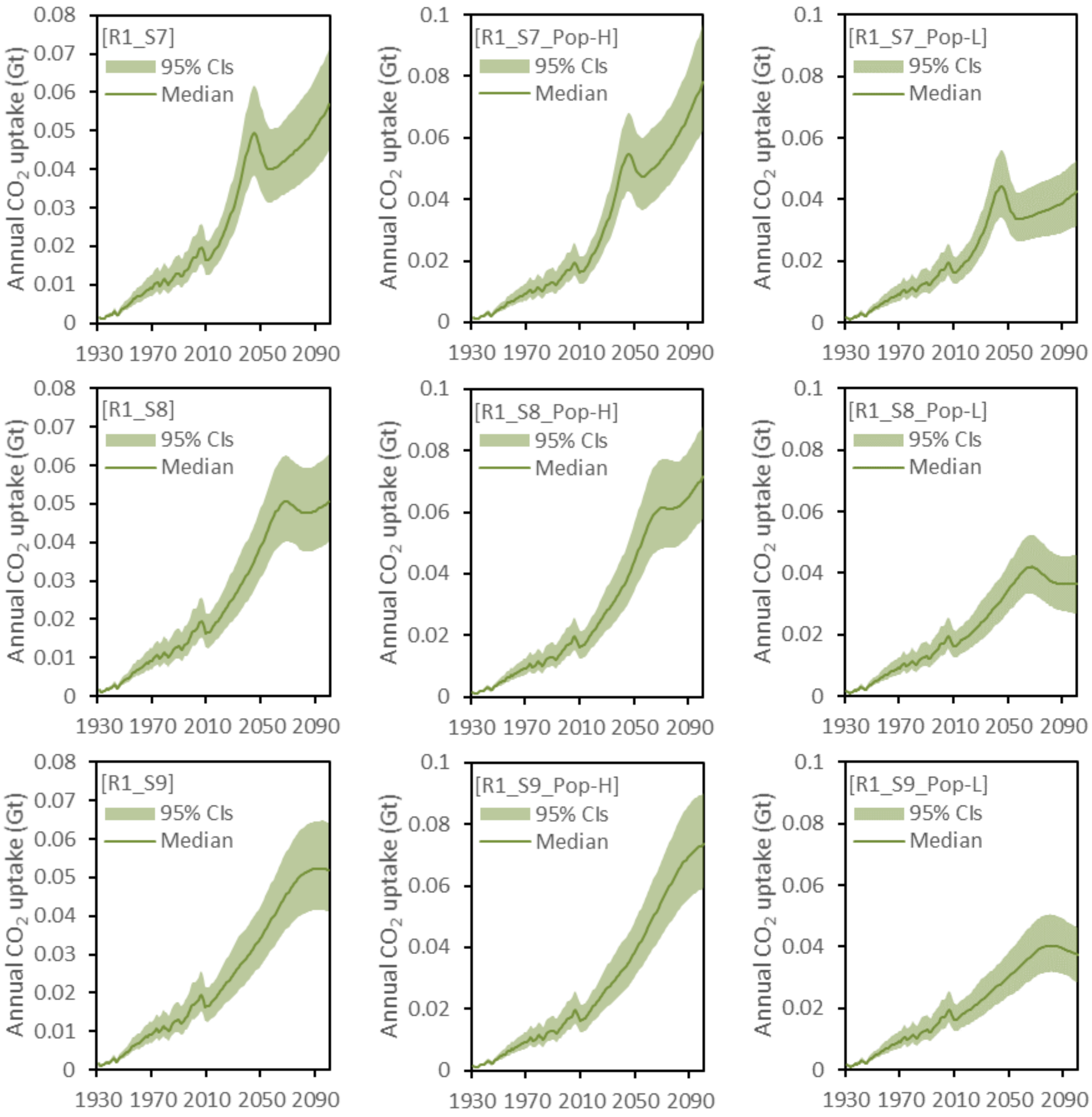

Supplementary Figure 48 | Annual CO_2 uptake in North America under scenarios S7, S8 and S9, if population is based on the medium-variant scenario (left column), the high-variant scenario (middle column), or the low-variant scenario (right column)

Note: The solid lines are the median value of simulated outcomes, and the shaded areas represent the 95% uncertainty range of simulated outcomes. The number of simulation runs is 1,000.

Supplementary Figure 49

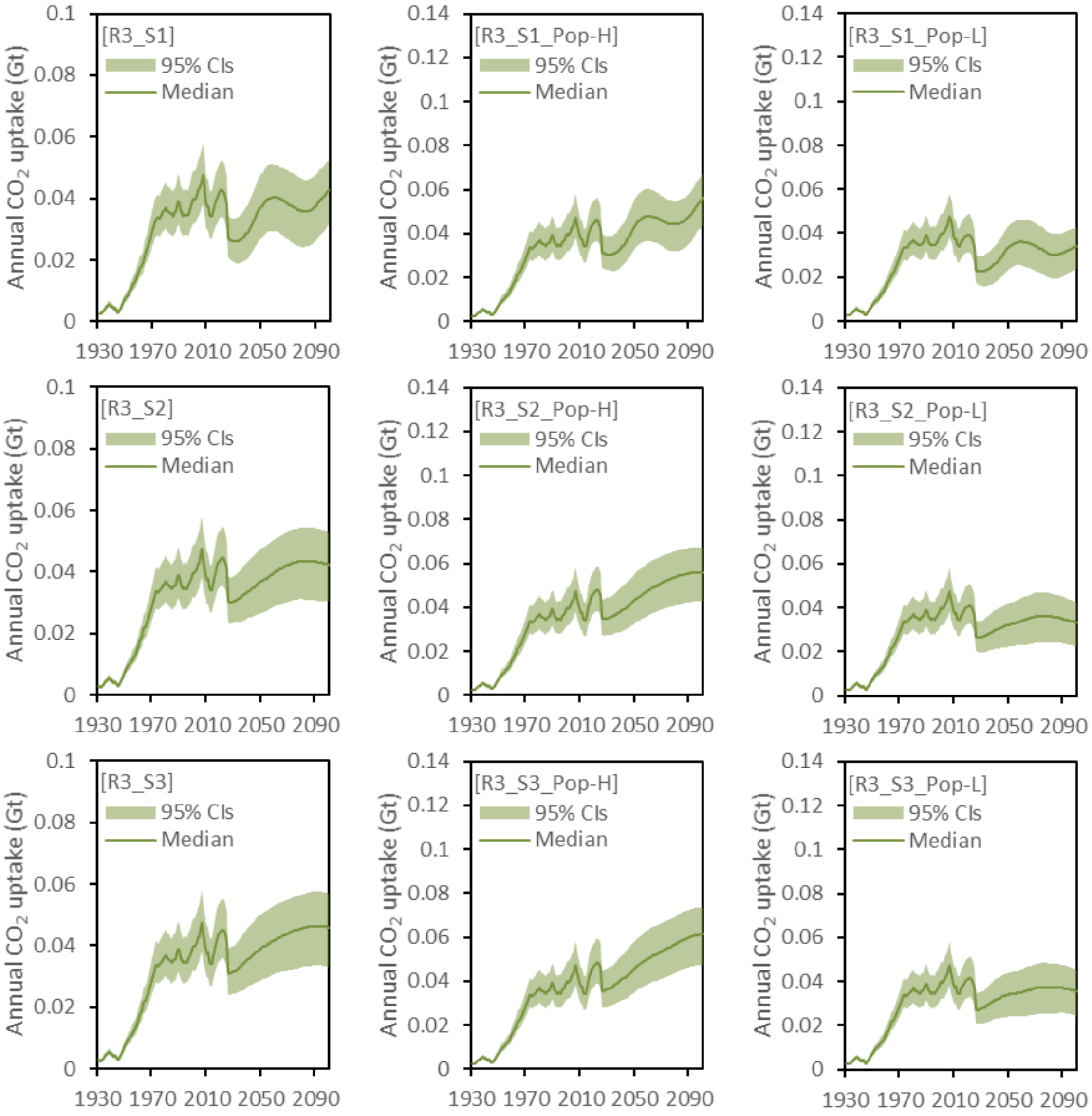

Supplementary Figure 49 | Annual CO_2 uptake in Europe under scenarios S1, S2 and S3, if population is based on the medium-variant scenario (left column), the high-variant scenario (middle column), or the low-variant scenario (right column)

Note: The solid lines are the median value of simulated outcomes, and the shaded areas represent the 95% uncertainty range of simulated outcomes. The number of simulation runs is 1,000.

Supplementary Figrue 50

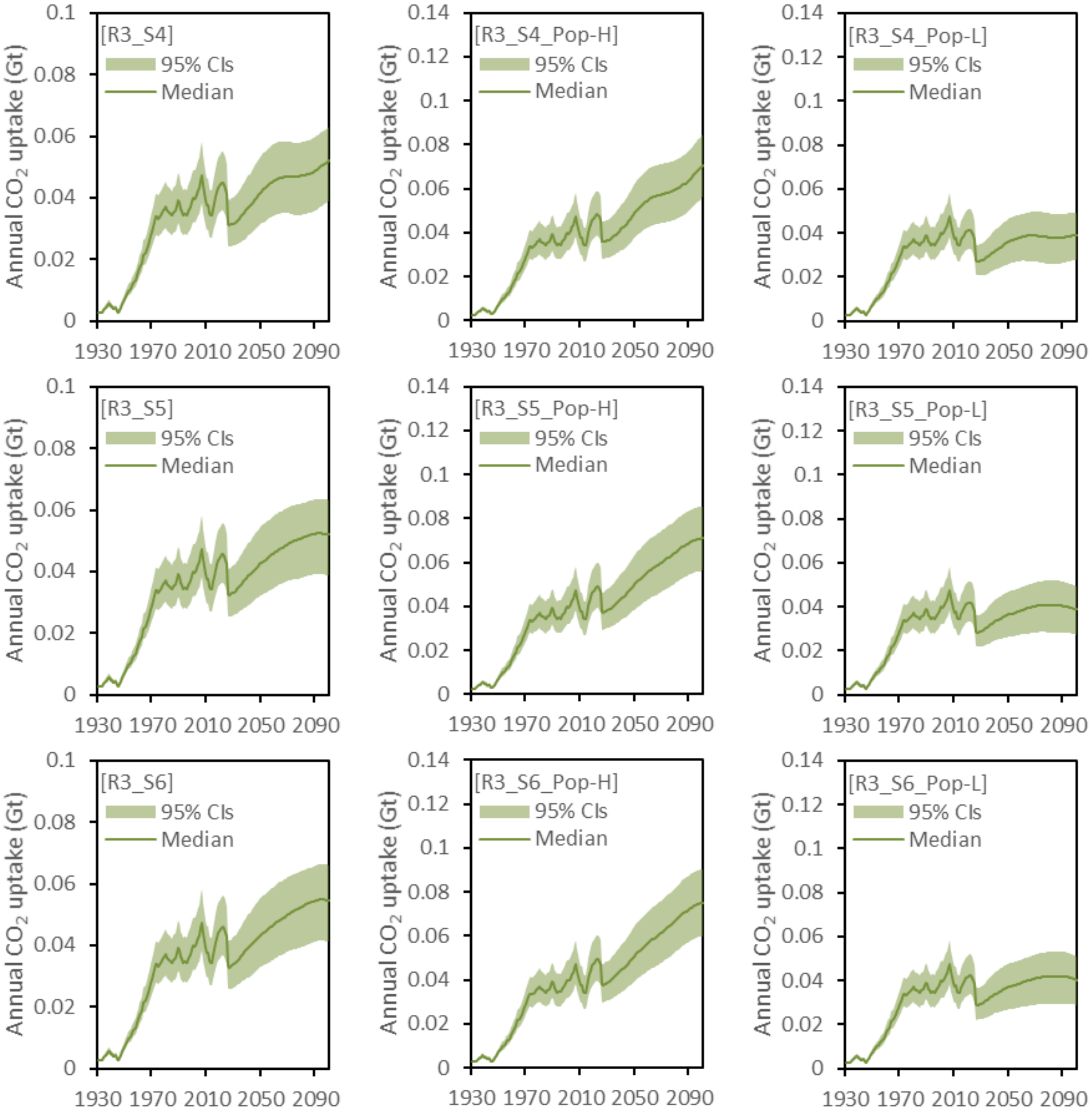

Supplementary Figure 50 | Annual CO_2 uptake in Europe under scenarios S4, S5 and S6, if population is based on the medium-variant scenario (left column), the high-variant scenario (middle column), or the low-variant scenario (right column)

Note: The solid lines are the median value of simulated outcomes, and the shaded areas represent the 95% uncertainty range of simulated outcomes. The number of simulation runs is 1,000.

Supplementary Figure 51

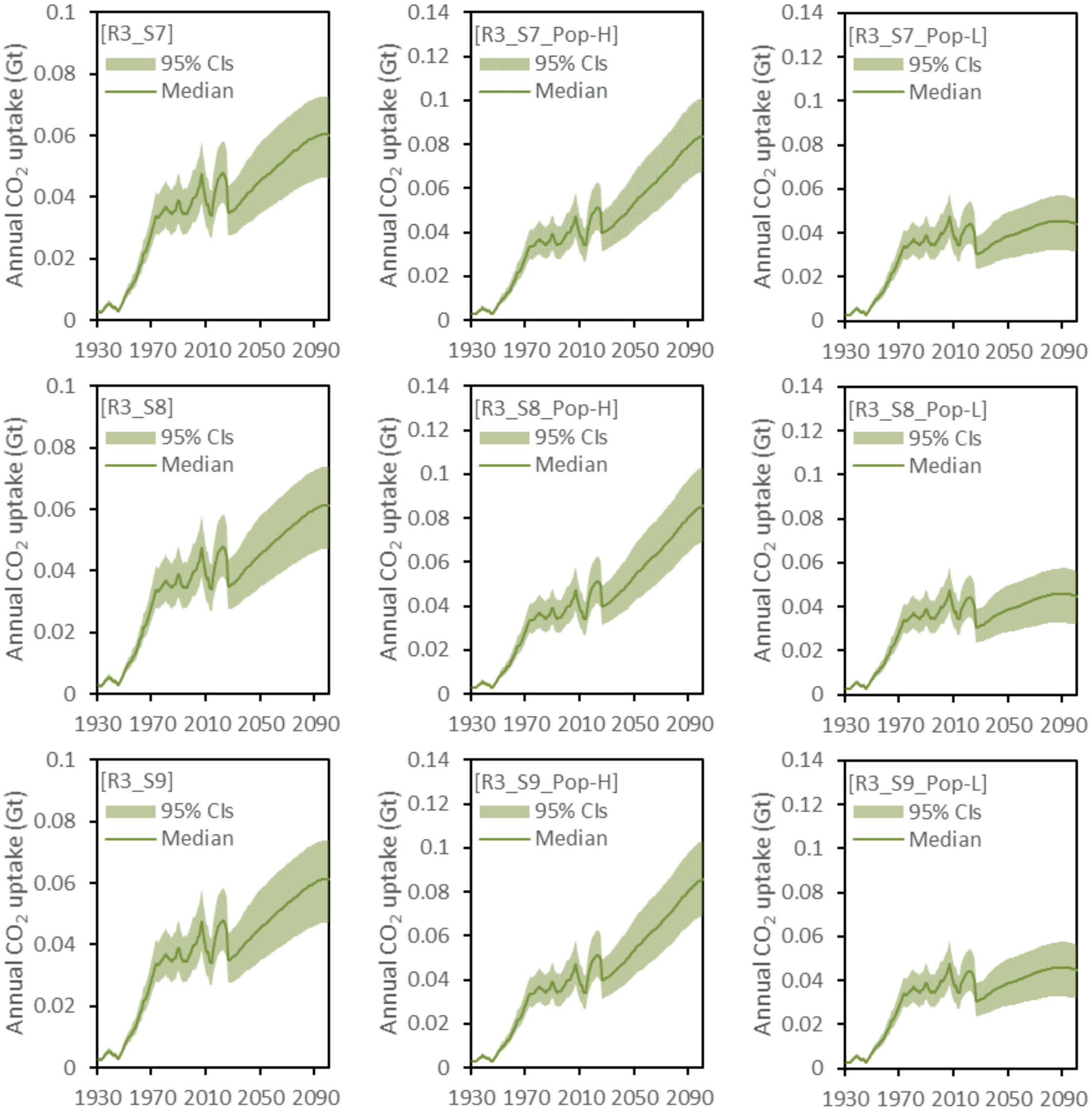

Supplementary Figure 51 | Annual CO_2 uptake in Europe under scenarios S7, S8 and S9, if population is based on the medium-variant scenario (left column), the high-variant scenario (middle column), or the low-variant scenario (right column)

Note: The solid lines are the median value of simulated outcomes, and the shaded areas represent the 95% uncertainty range of simulated outcomes. The number of simulation runs is 1,000.

Supplementary Figure 52

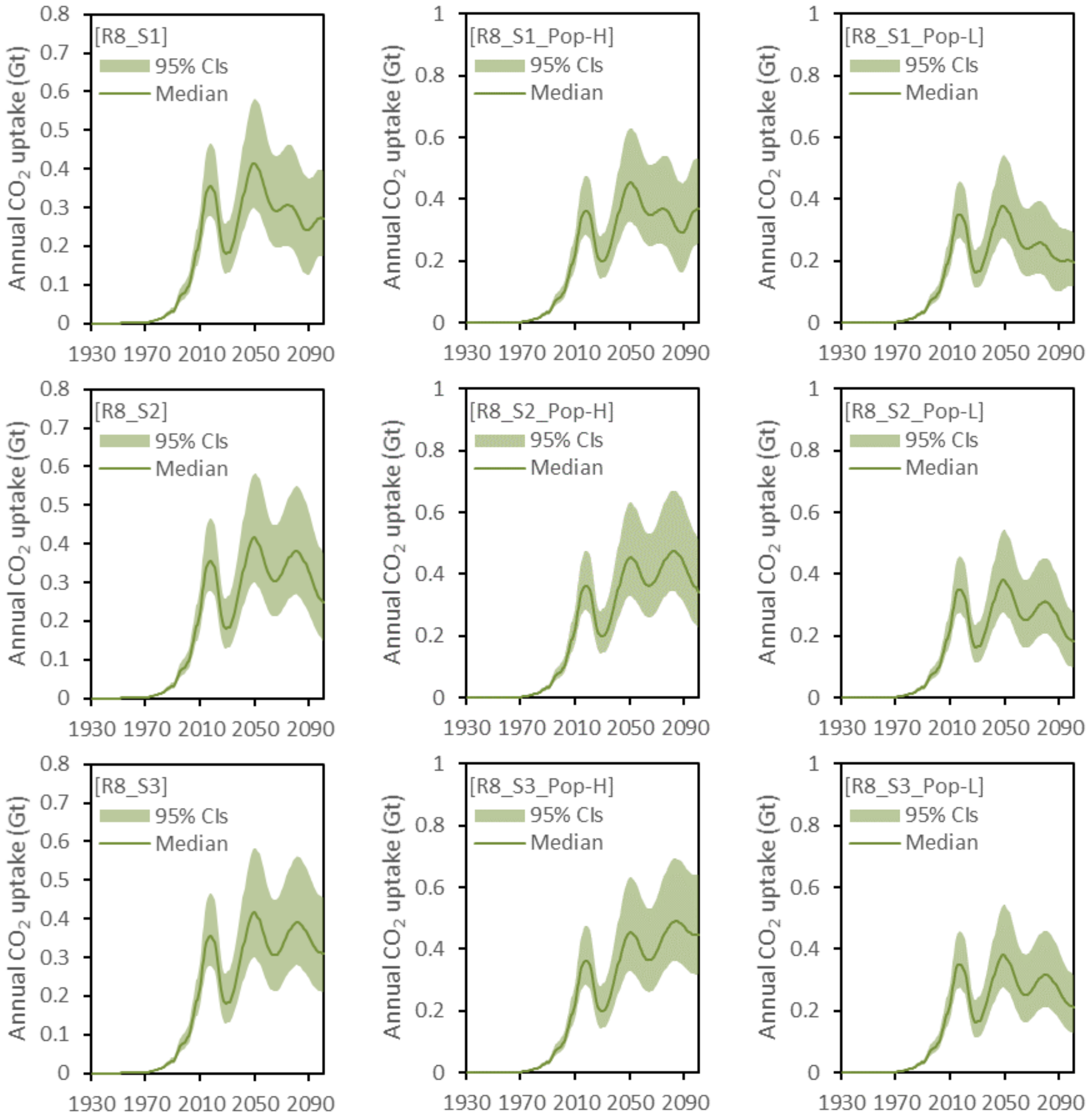

Supplementary Figure 52 | Annual CO_2 uptake in China under scenarios S1, S2 and S3, if population is based on the medium-variant scenario (left column), the high-variant scenario (middle column), or the low-variant scenario (right column)

Note: The solid lines are the median value of simulated outcomes, and the shaded areas represent the 95% uncertainty range of simulated outcomes. The number of simulation runs is 1,000.

Supplementary Figure 53

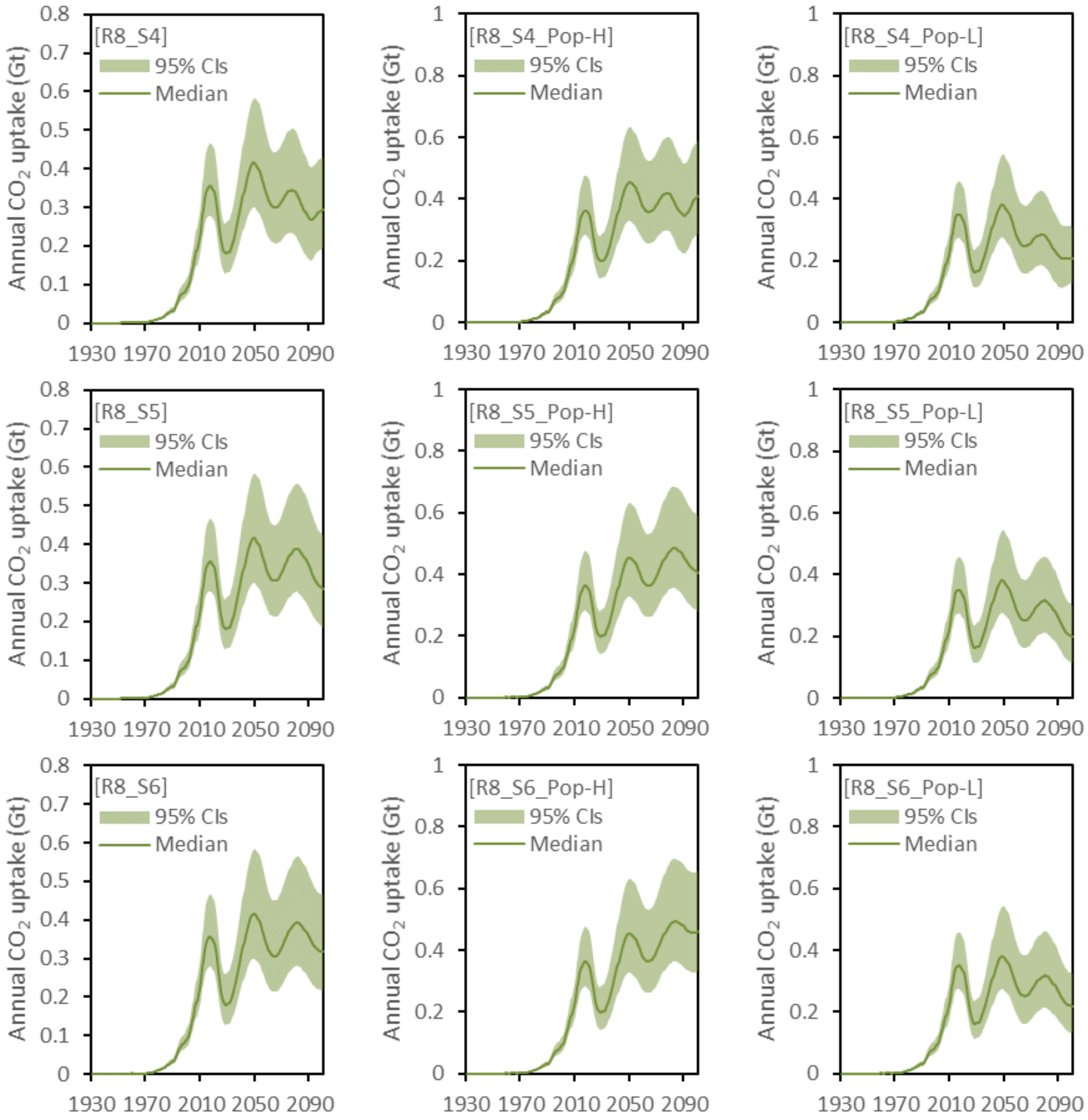

Supplementary Figure 53 | Annual CO_2 uptake in China under scenarios S4, S5 and S6, if population is based on the medium-variant scenario (left column), the high-variant scenario (middle column), or the low-variant scenario (right column)

Note: The solid lines are the median value of simulated outcomes, and the shaded areas represent the 95% uncertainty range of simulated outcomes. The number of simulation runs is 1,000.

Supplementary Figure 54

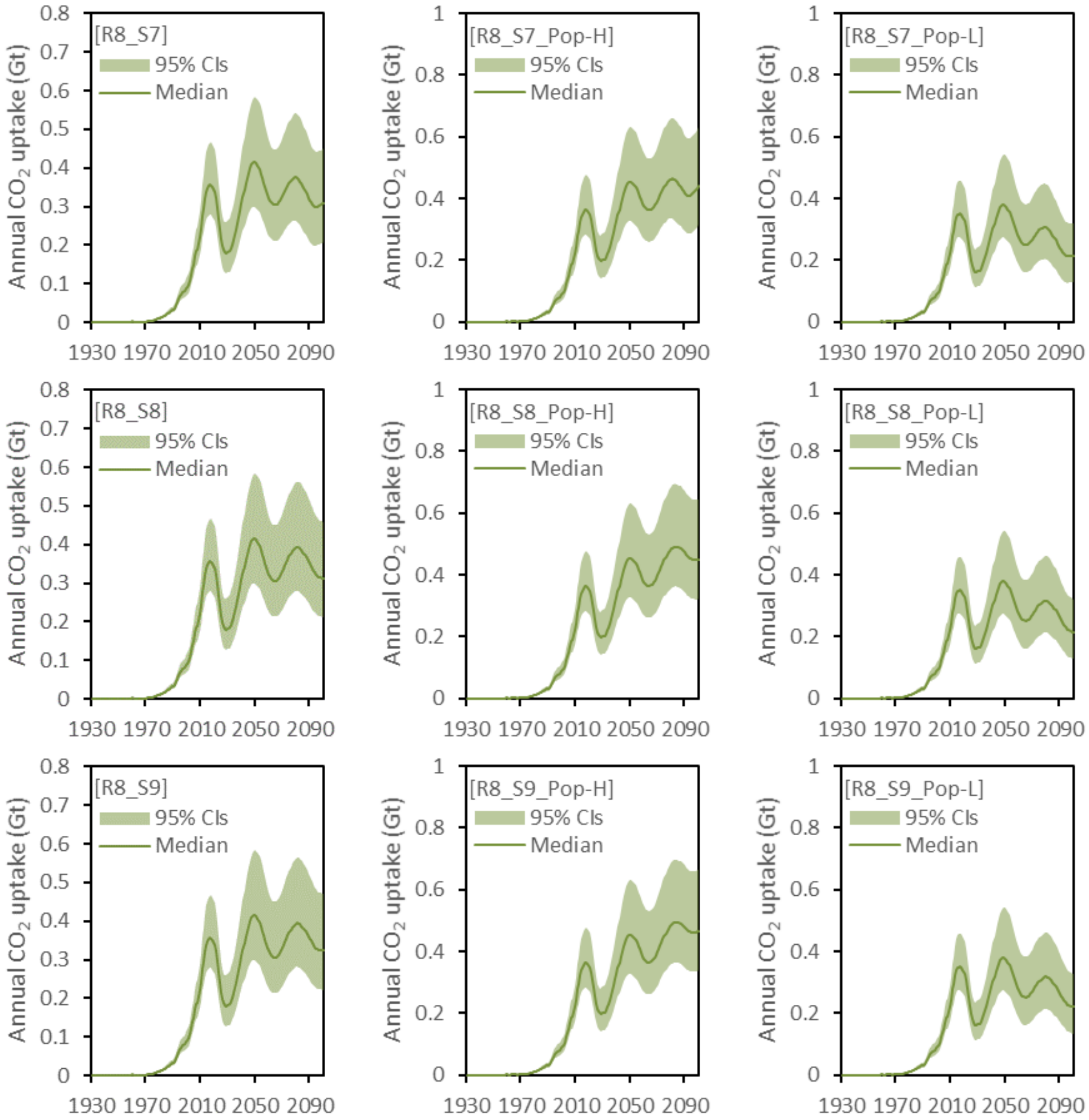

Supplementary Figure 54 | Annual CO_2 uptake in China under scenarios S7, S8 and S9, if population is based on the medium-variant scenario (left column), the high-variant scenario (middle column), or the low-variant scenario (right column)

Note: The solid lines are the median value of simulated outcomes, and the shaded areas represent the 95% uncertainty range of simulated outcomes. The number of simulation runs is 1,000.

Supplementary Figure 55

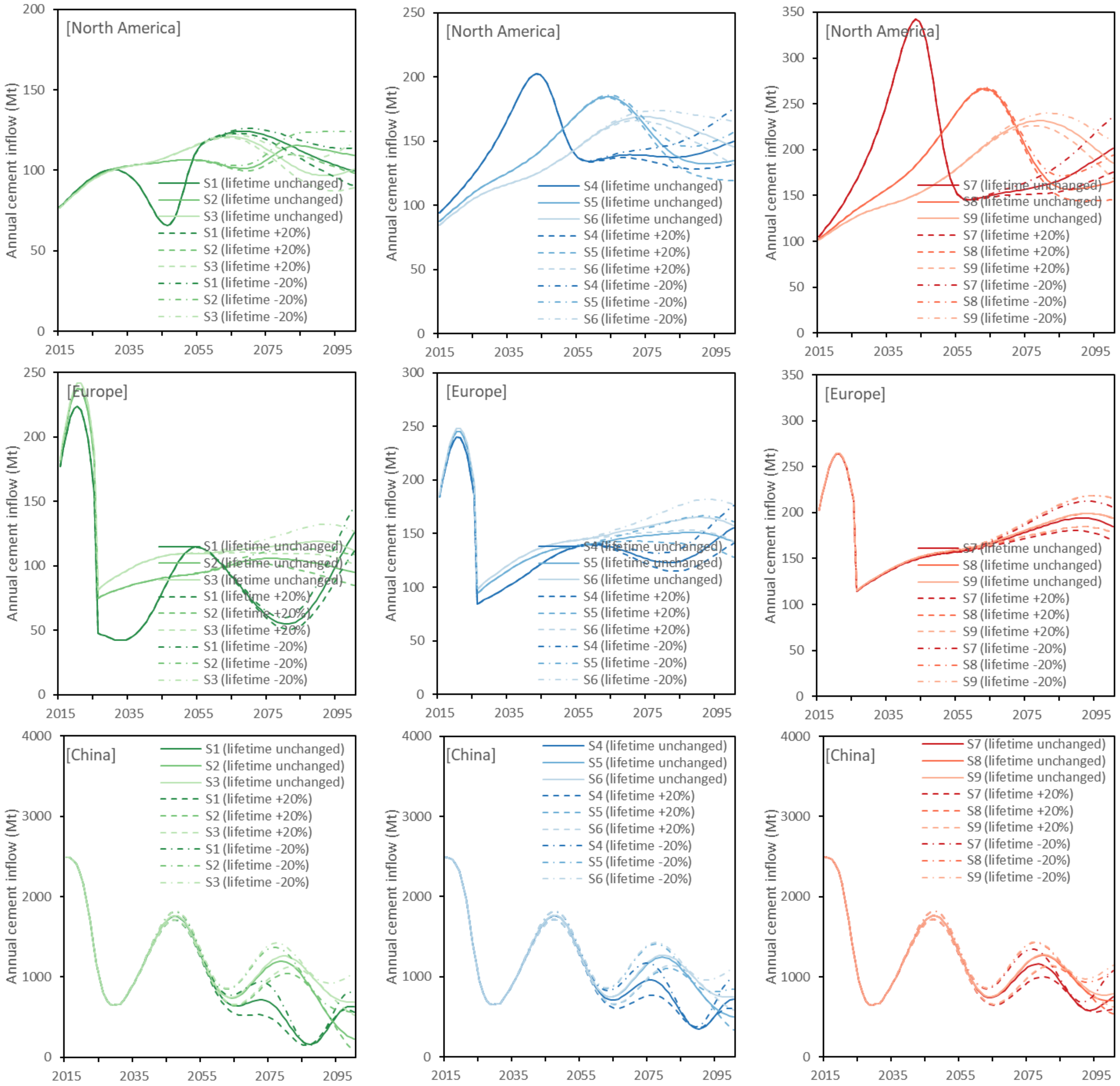

Supplementary Figure 55 | Annual cement inflows, if lifetime is unchanged, increased by 20%, or decreased by 20%

Supplementary Figure 56

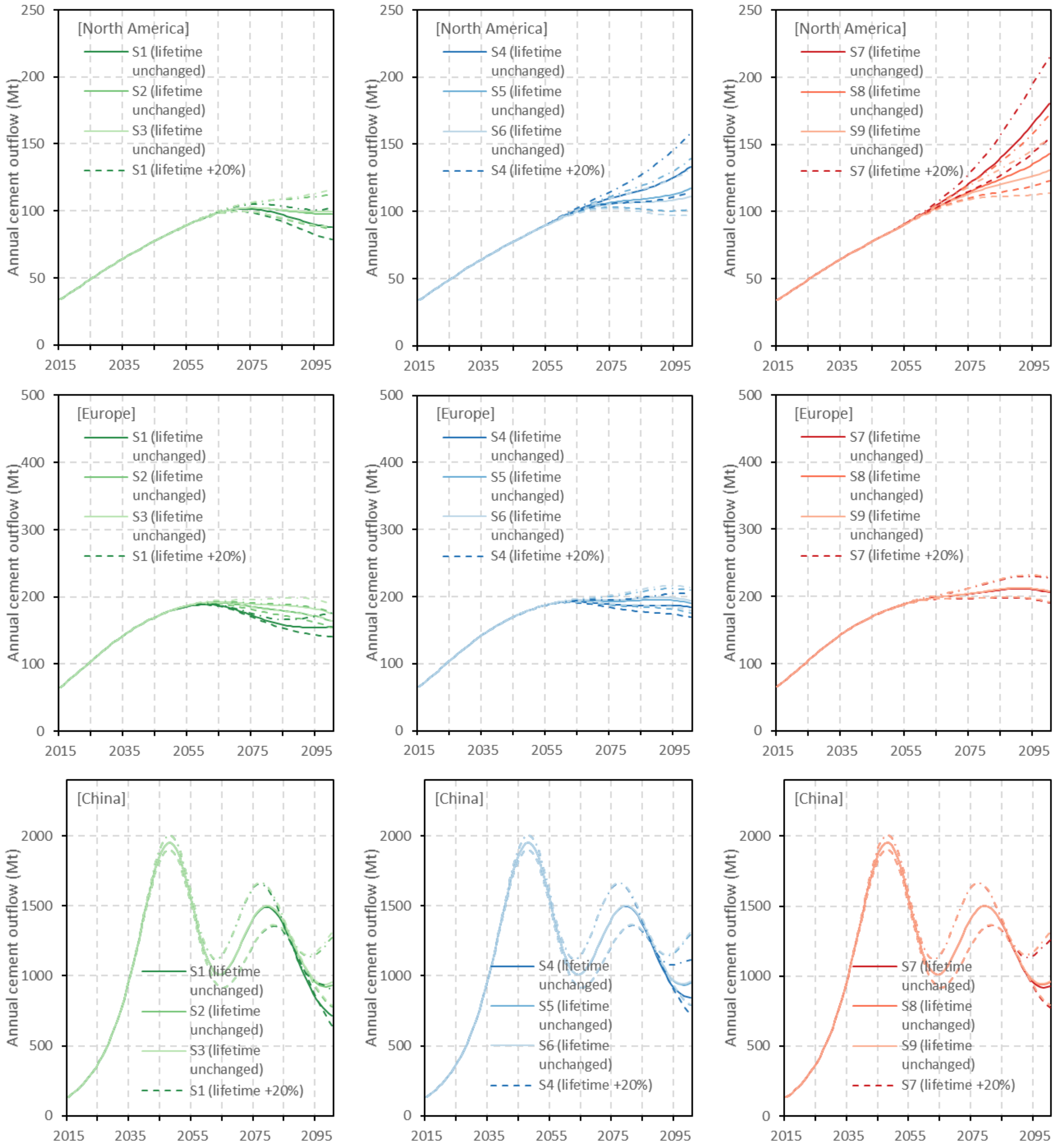

Supplementary Figure 56 | Annual cement outflows, if lifetime is unchanged, increased by 20%, or decreased by 20%

Supplementary Figure 57

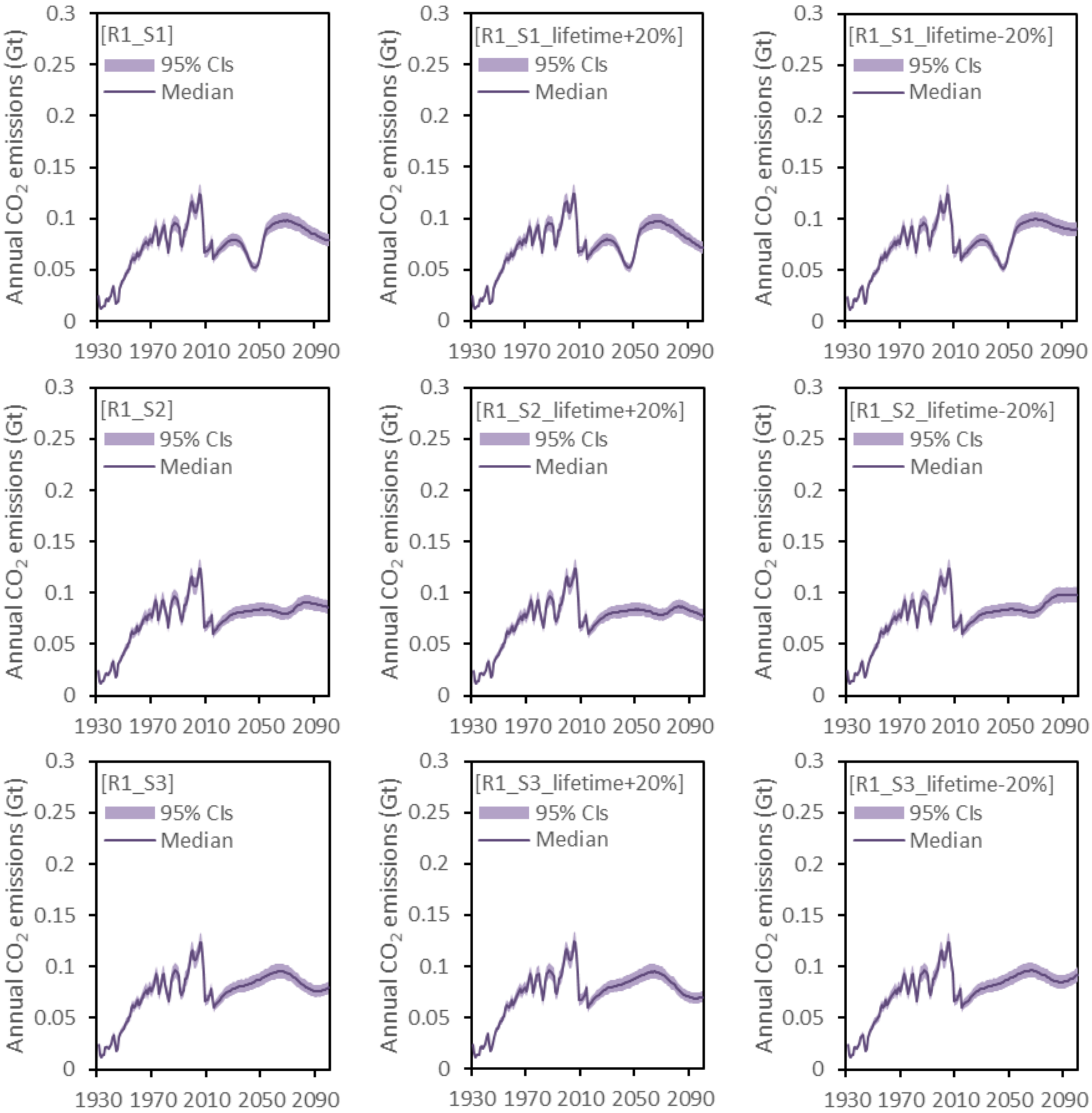

Supplementary Figure 57 | Annual CO_2 emissions in North America under scenarios S1, S2 and S3, if lifetime is unchanged (left column), increased by 20% (middle column), or decreased by 20% (right column)

Note: The solid lines are the median value of simulated outcomes, and the shaded areas represent the 95% uncertainty range of simulated outcomes. The number of simulation runs is 1,000.

Supplementary Figure 58

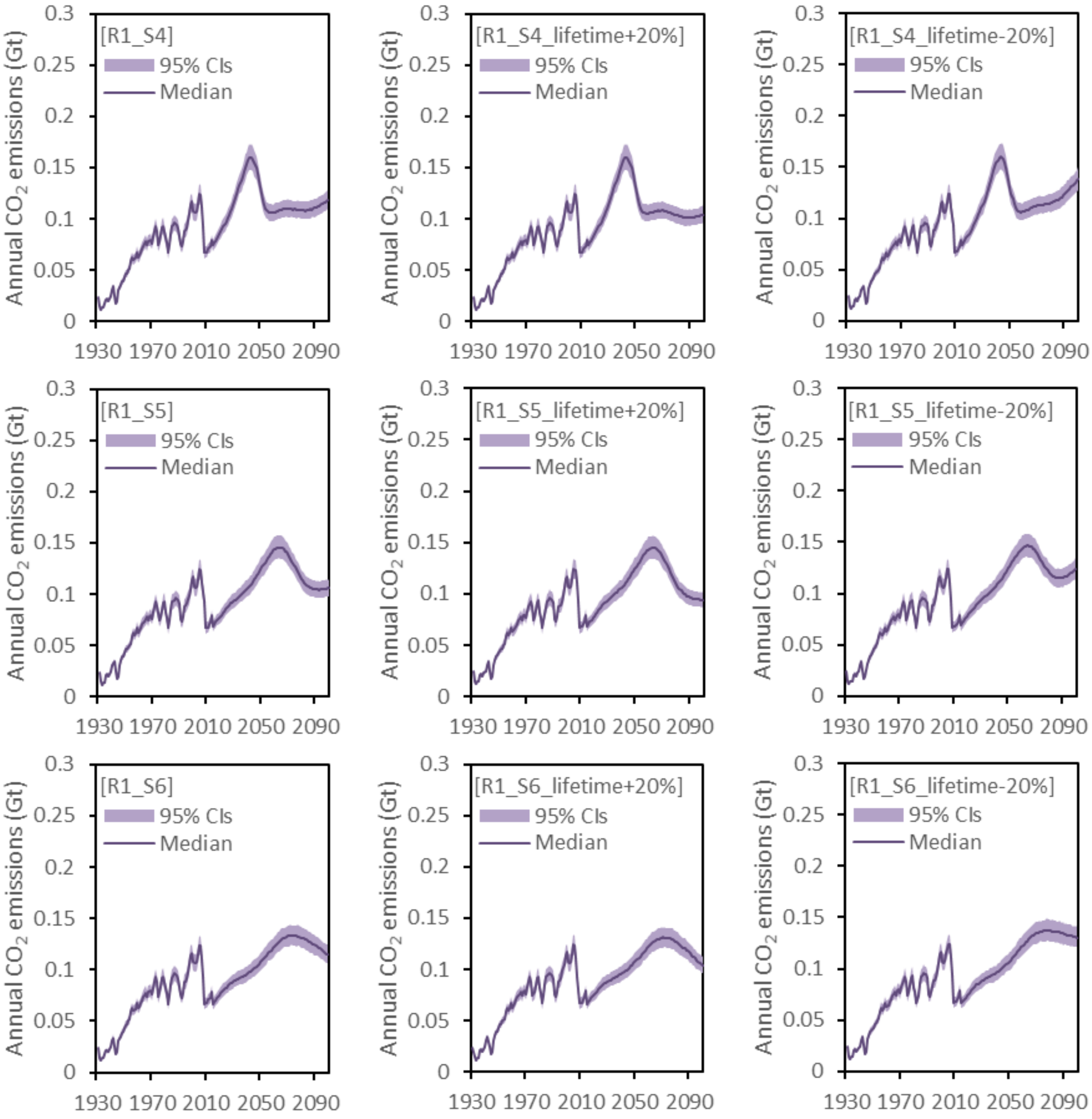

Supplementary Figure 58 | Annual CO_2 emissions in North America under scenarios S4, S5 and S6, if lifetime is unchanged (left column), increased by 20% (middle column), or decreased by 20% (right column)

Note: The solid lines are the median value of simulated outcomes, and the shaded areas represent the 95% uncertainty range of simulated outcomes. The number of simulation runs is 1,000.

Supplementary Figure 59

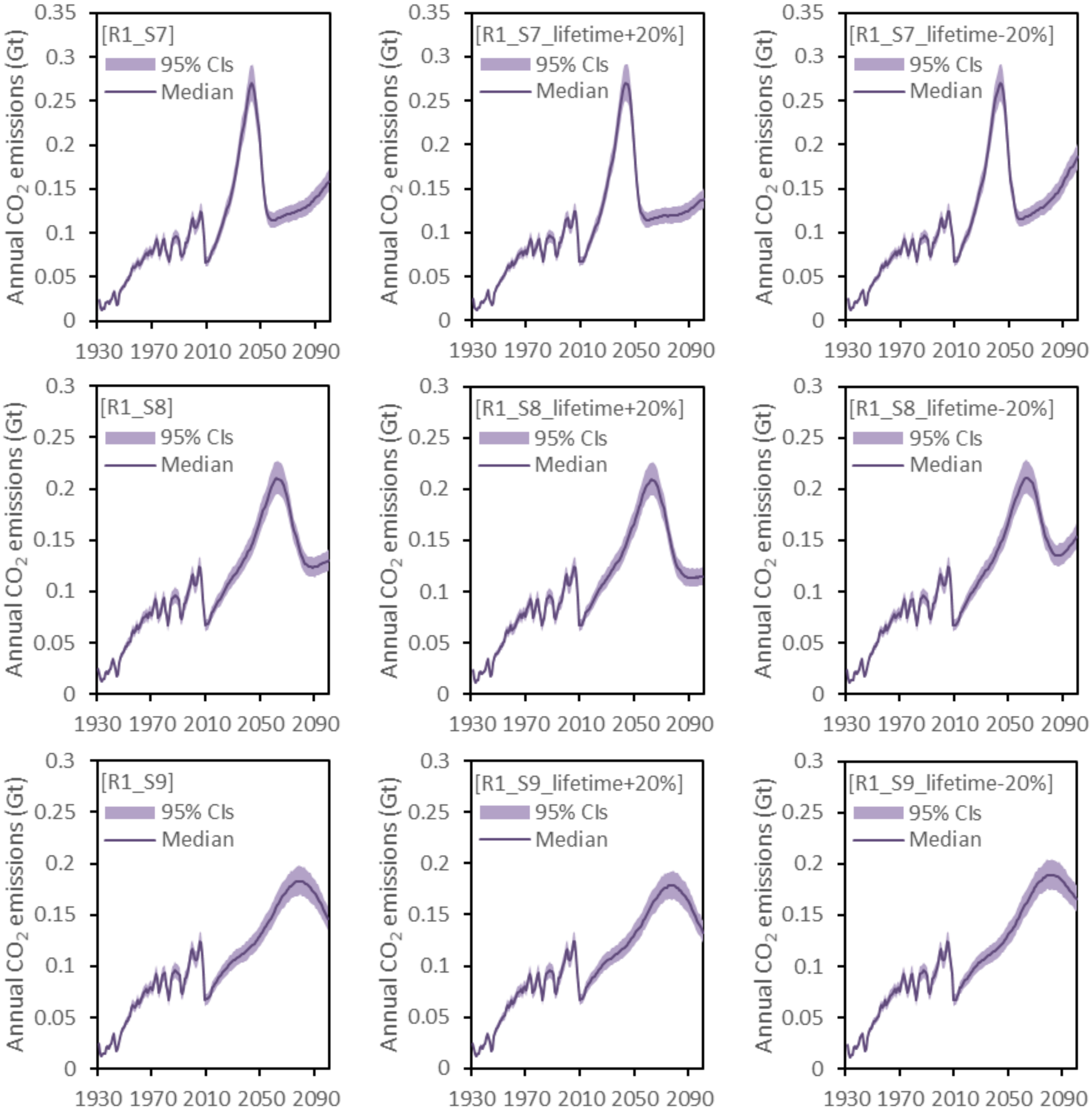

Supplementary Figure 59 | Annual CO_2 emissions in North America under scenarios S7, S8 and S9, if lifetime is unchanged (left column), increased by 20% (middle column), or decreased by 20% (right column)

Note: The solid lines are the median value of simulated outcomes, and the shaded areas represent the 95% uncertainty range of simulated outcomes. The number of simulation runs is 1,000.

Supplementary Figure 60

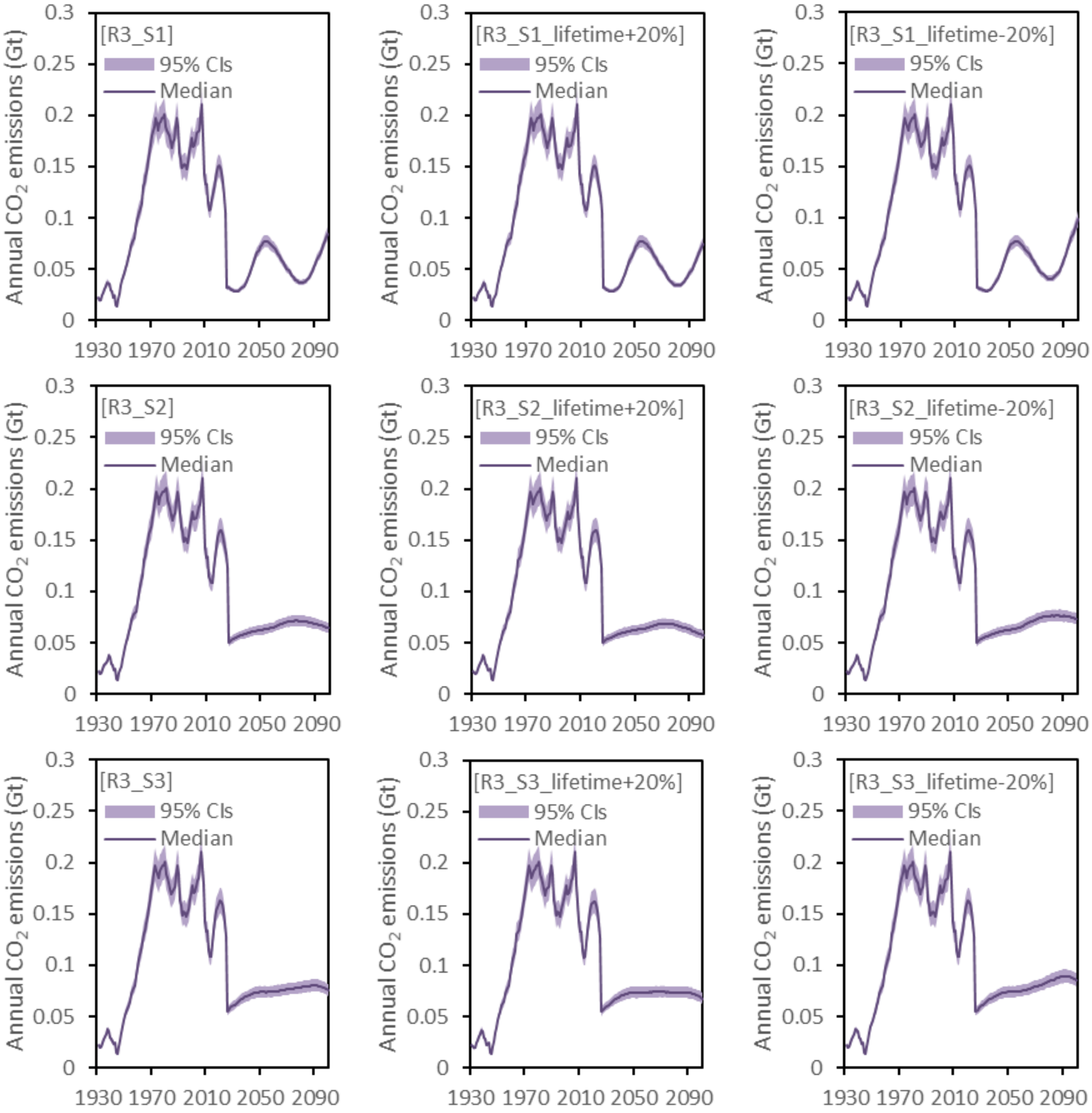

Supplementary Figure 60 | Annual CO_2 emissions in Europe under scenarios S1, S2 and S3, if lifetime is unchanged (left column), increased by 20% (middle column), or decreased by 20% (right column)

Note: The solid lines are the median value of simulated outcomes, and the shaded areas represent the 95% uncertainty range of simulated outcomes. The number of simulation runs is 1,000.

Supplementary Figure 61

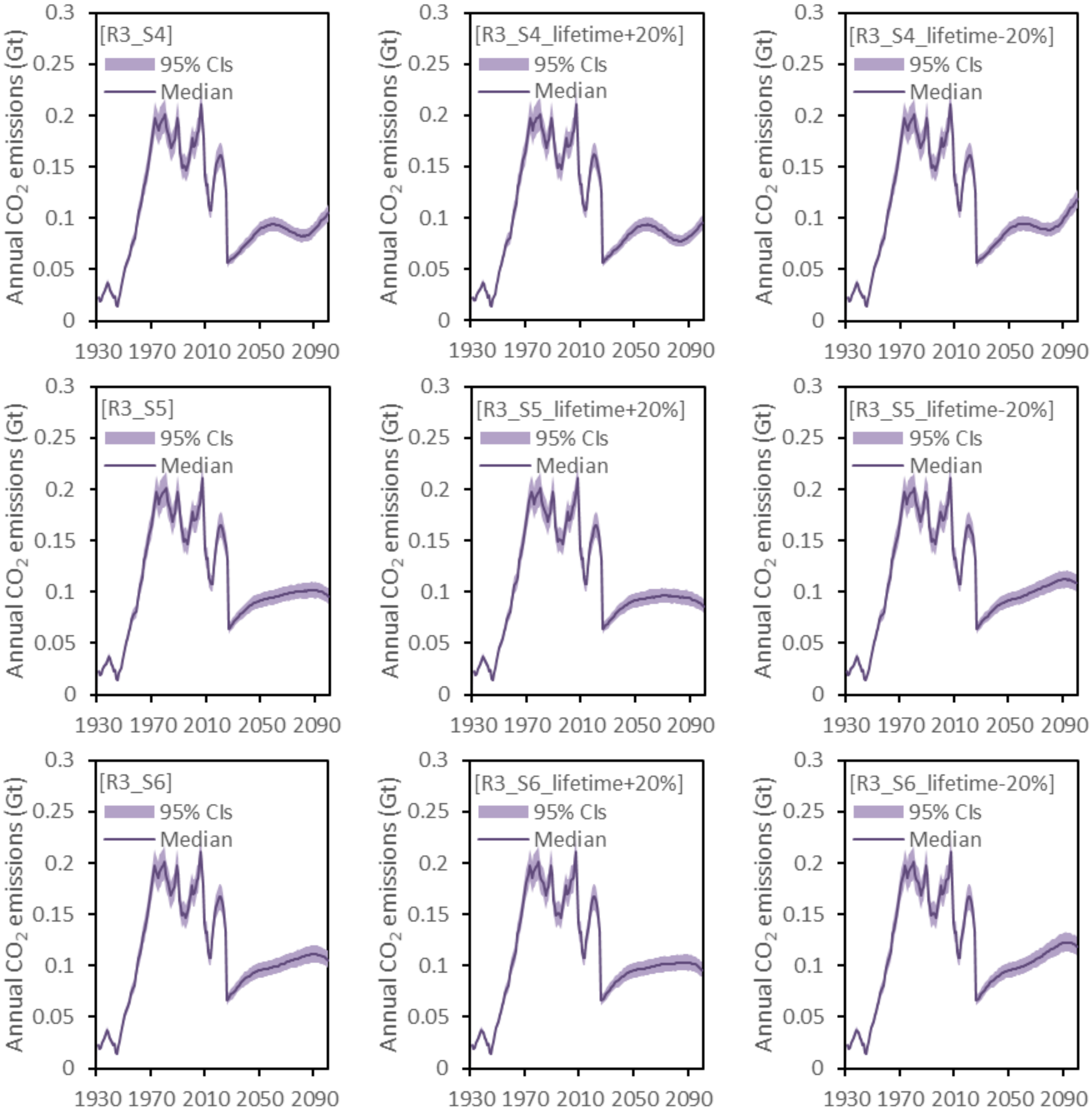

Supplementary Figure 61 | Annual CO_2 emissions in Europe under scenarios S4, S5 and S6, if lifetime is unchanged (left column), increased by 20% (middle column), or decreased by 20% (right column)

Note: The solid lines are the median value of simulated outcomes, and the shaded areas represent the 95% uncertainty range of simulated outcomes. The number of simulation runs is 1,000.

Supplementary Figure 62

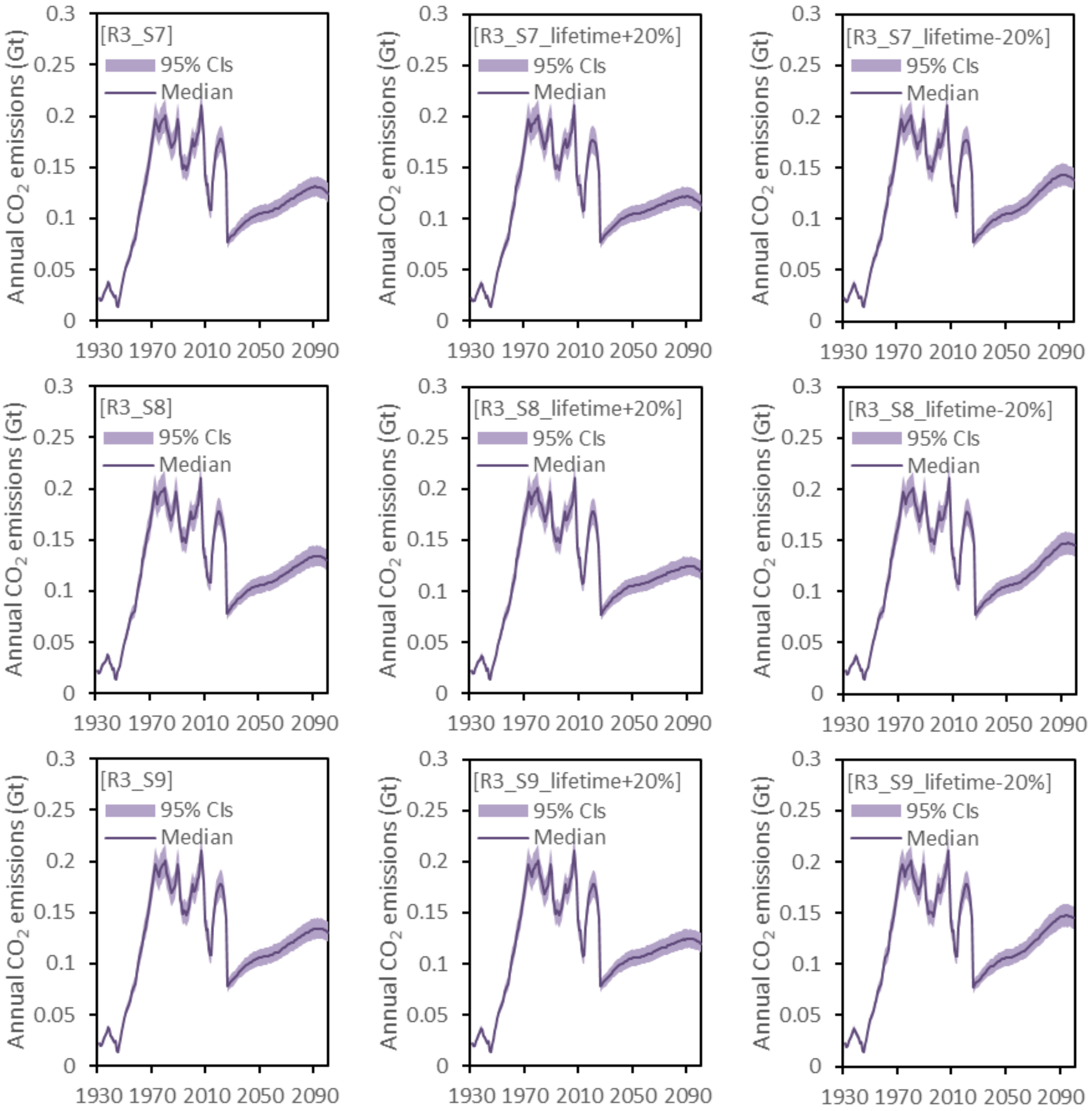

Supplementary Figure 62 | Annual CO_2 emissions in Europe under scenarios S7, S8 and S9, if lifetime is unchanged (left column), increased by 20% (middle column), or decreased by 20% (right column)

Note: The solid lines are the median value of simulated outcomes, and the shaded areas represent the 95% uncertainty range of simulated outcomes. The number of simulation runs is 1,000.

Supplementary Figure 63

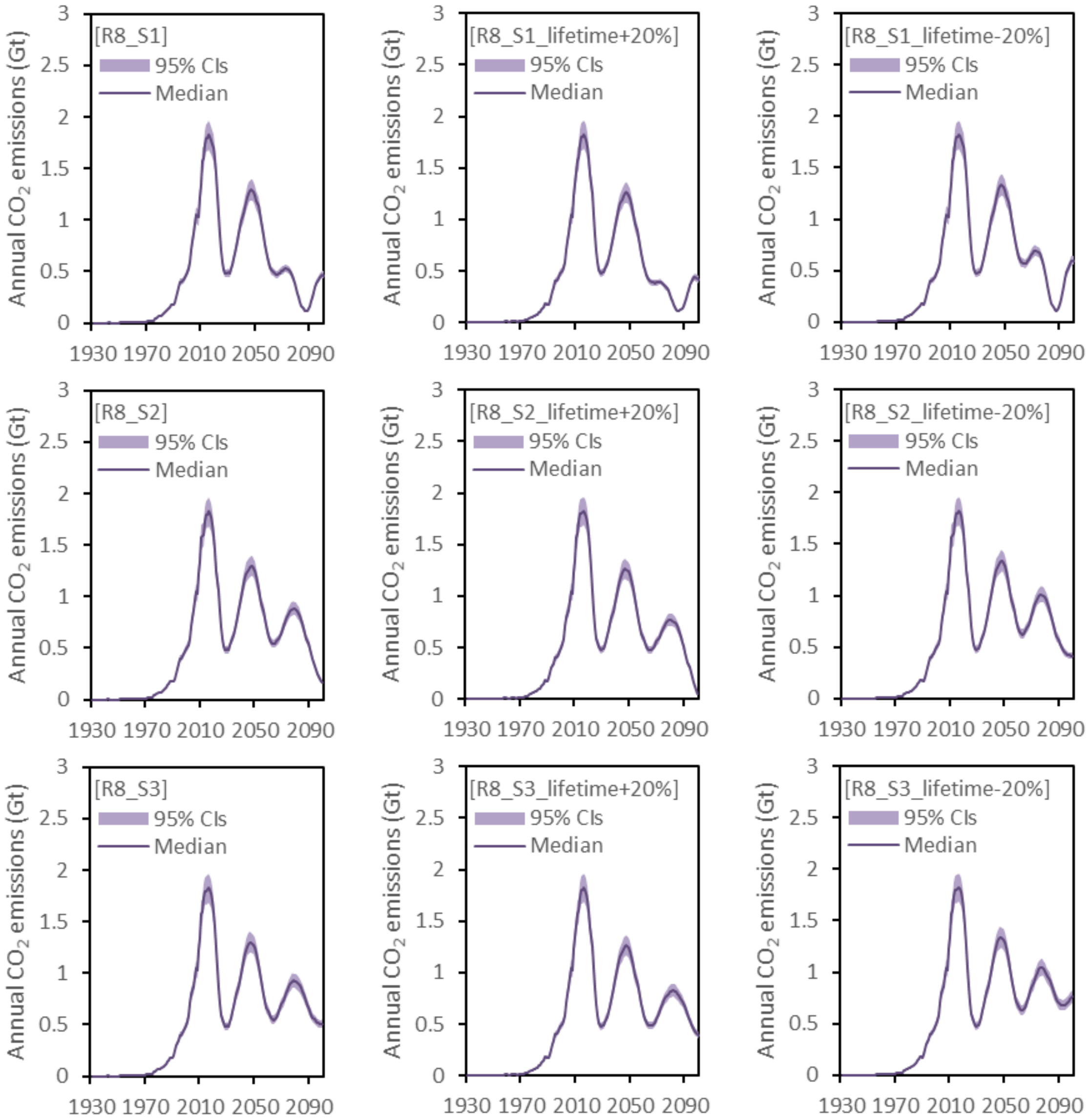

Supplementary Figure 63 | Annual CO_2 emissions in China under scenarios S1, S2 and S3, if lifetime is unchanged (left column), increased by 20% (middle column), or decreased by 20% (right column)

Note: The solid lines are the median value of simulated outcomes, and the shaded areas represent the 95% uncertainty range of simulated outcomes. The number of simulation runs is 1,000.

Supplementary Figure 64

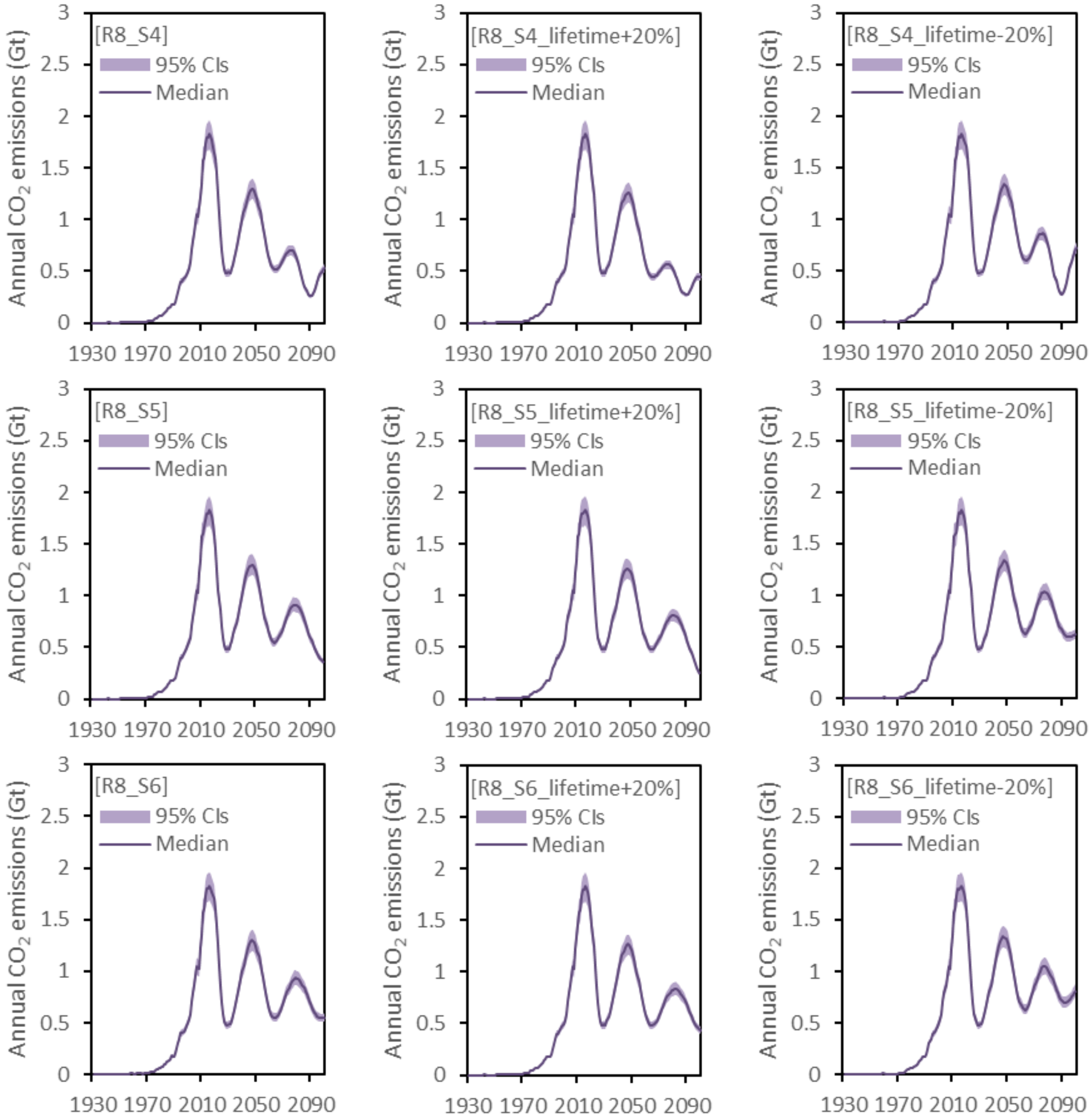

Supplementary Figure 64 | Annual CO_2 emissions in China under scenarios S4, S5 and S6, if lifetime is unchanged (left column), increased by 20% (middle column), or decreased by 20% (right column)

Note: The solid lines are the median value of simulated outcomes, and the shaded areas represent the 95% uncertainty range of simulated outcomes. The number of simulation runs is 1,000.

Supplementary Figure 65

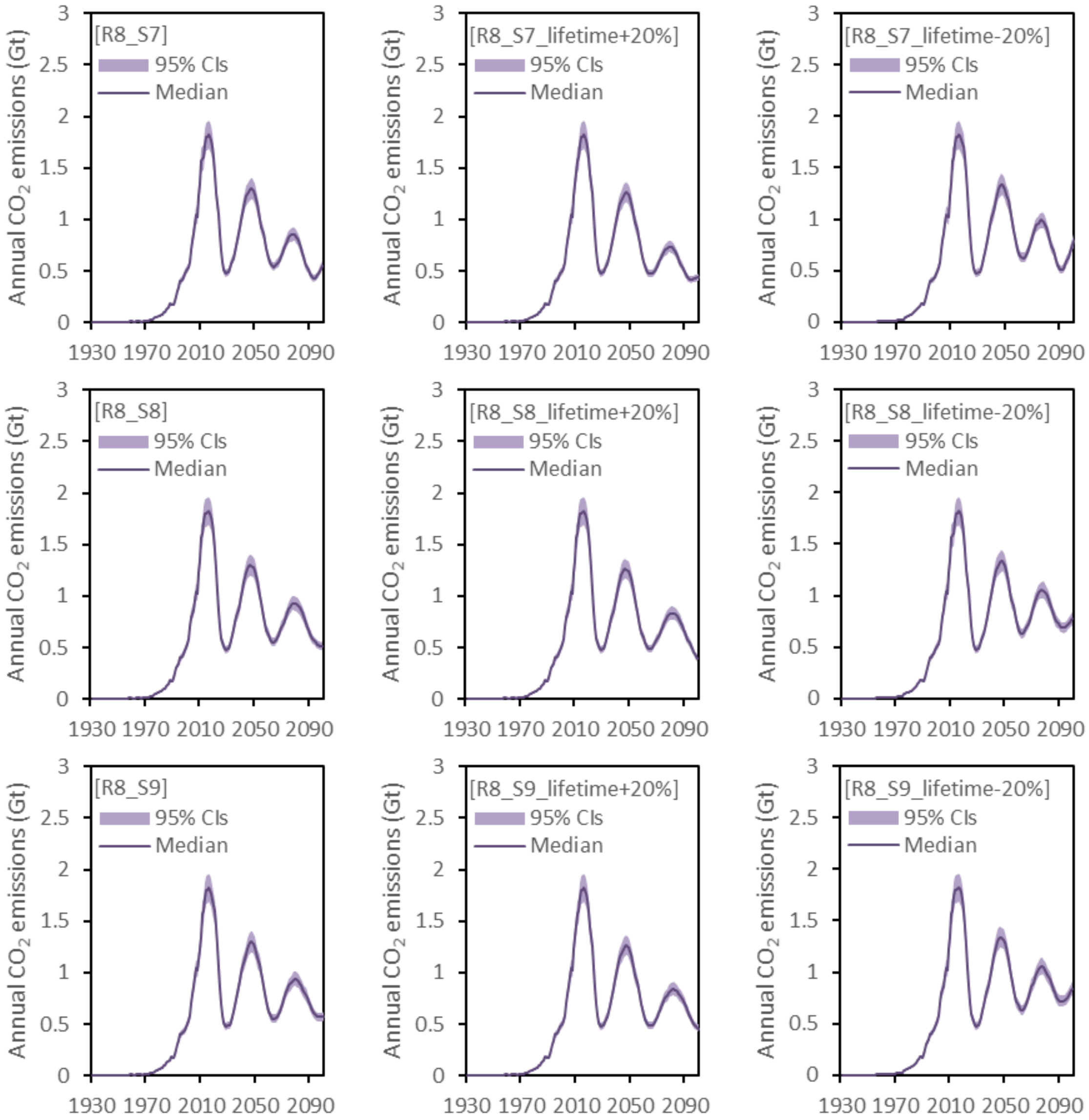

Supplementary Figure 65 | Annual CO_2 emissions in China under scenarios S7, S8 and S9, if lifetime is unchanged (left column), increased by 20% (middle column), or decreased by 20% (right column)

Note: The solid lines are the median value of simulated outcomes, and the shaded areas represent the 95% uncertainty range of simulated outcomes. The number of simulation runs is 1,000.

Supplementary Figure 66

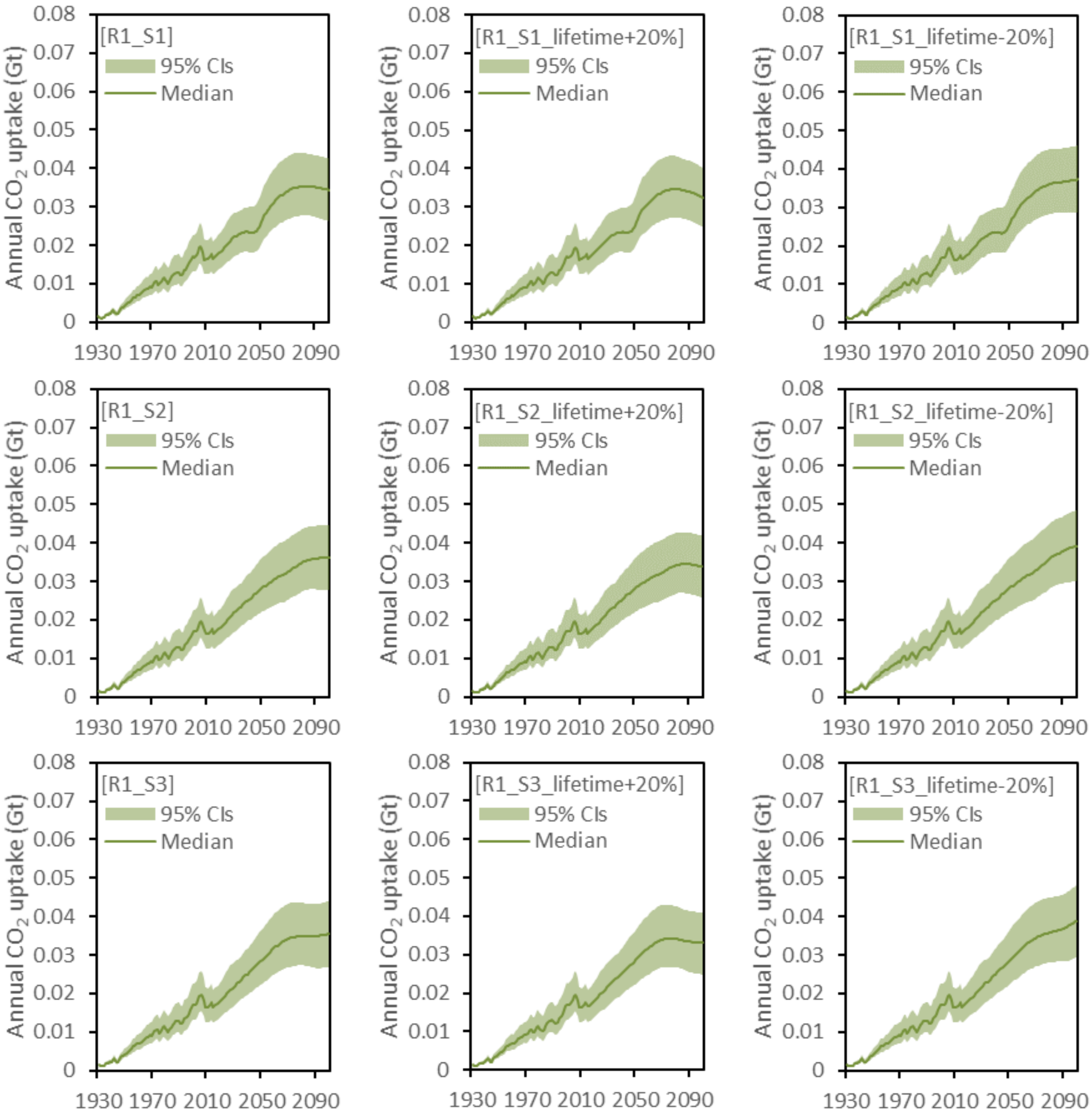

Supplementary Figure 66 | Annual CO_2 uptake in North America under scenarios S1, S2 and S3, if lifetime is unchanged (left column), increased by 20% (middle column), or decreased by 20% (right column)

Note: The solid lines are the median value of simulated outcomes, and the shaded areas represent the 95% uncertainty range of simulated outcomes. The number of simulation runs is 1,000.

Supplementary Figure 67

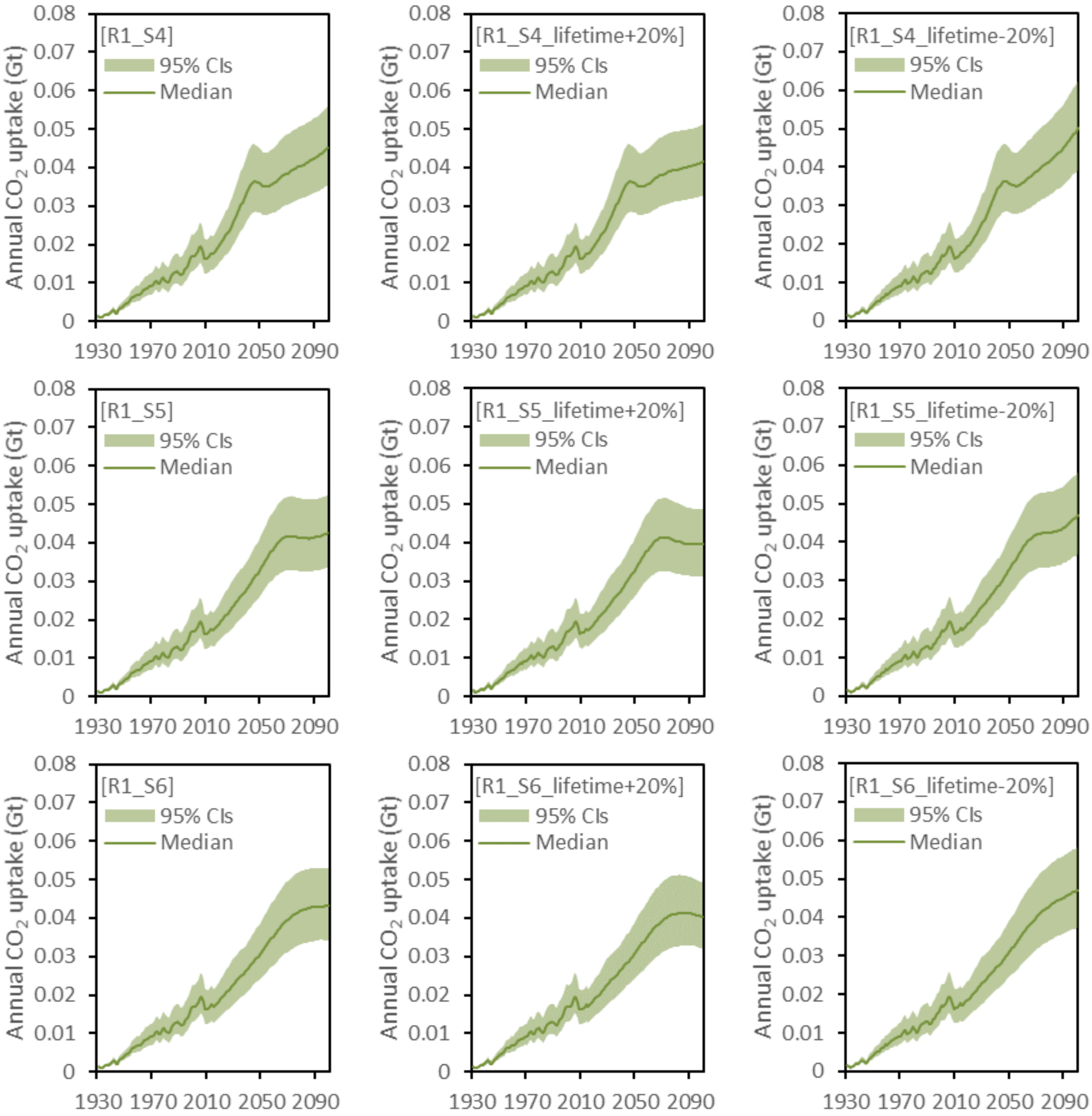

Supplementary Figure 67 | Annual CO_2 uptake in North America under scenarios S4, S5 and S6, if lifetime is unchanged (left column), increased by 20% (middle column), or decreased by 20% (right column)

Note: The solid lines are the median value of simulated outcomes, and the shaded areas represent the 95% uncertainty range of simulated outcomes. The number of simulation runs is 1,000.

Supplementary Figure 68

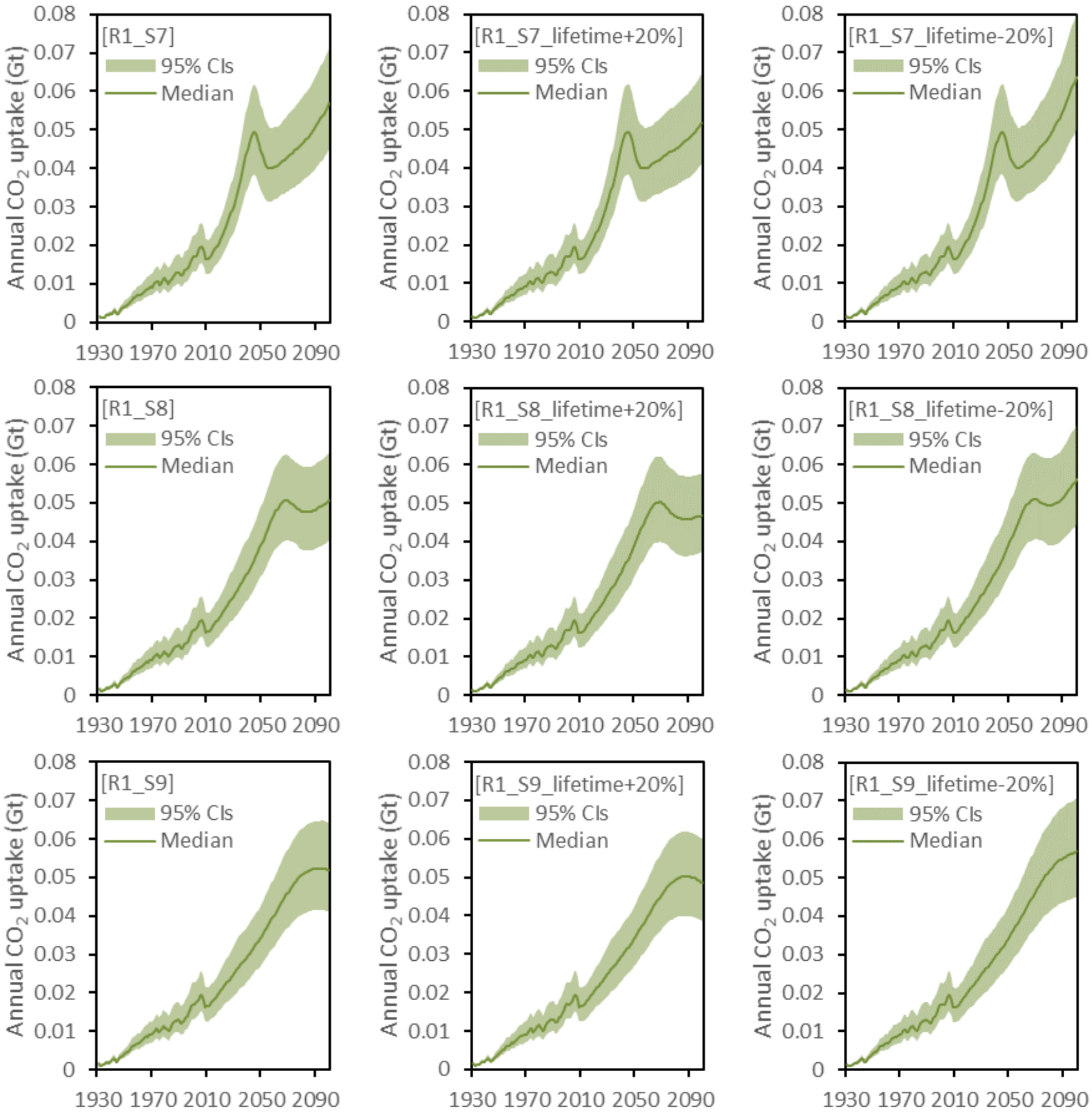

Supplementary Figure 68 | Annual CO_2 uptake in North America under scenarios S7, S8 and S9, if lifetime is unchanged (left column), increased by 20% (middle column), or decreased by 20% (right column)

Note: The solid lines are the median value of simulated outcomes, and the shaded areas represent the 95% uncertainty range of simulated outcomes. The number of simulation runs is 1,000.

Supplementary Figure 69

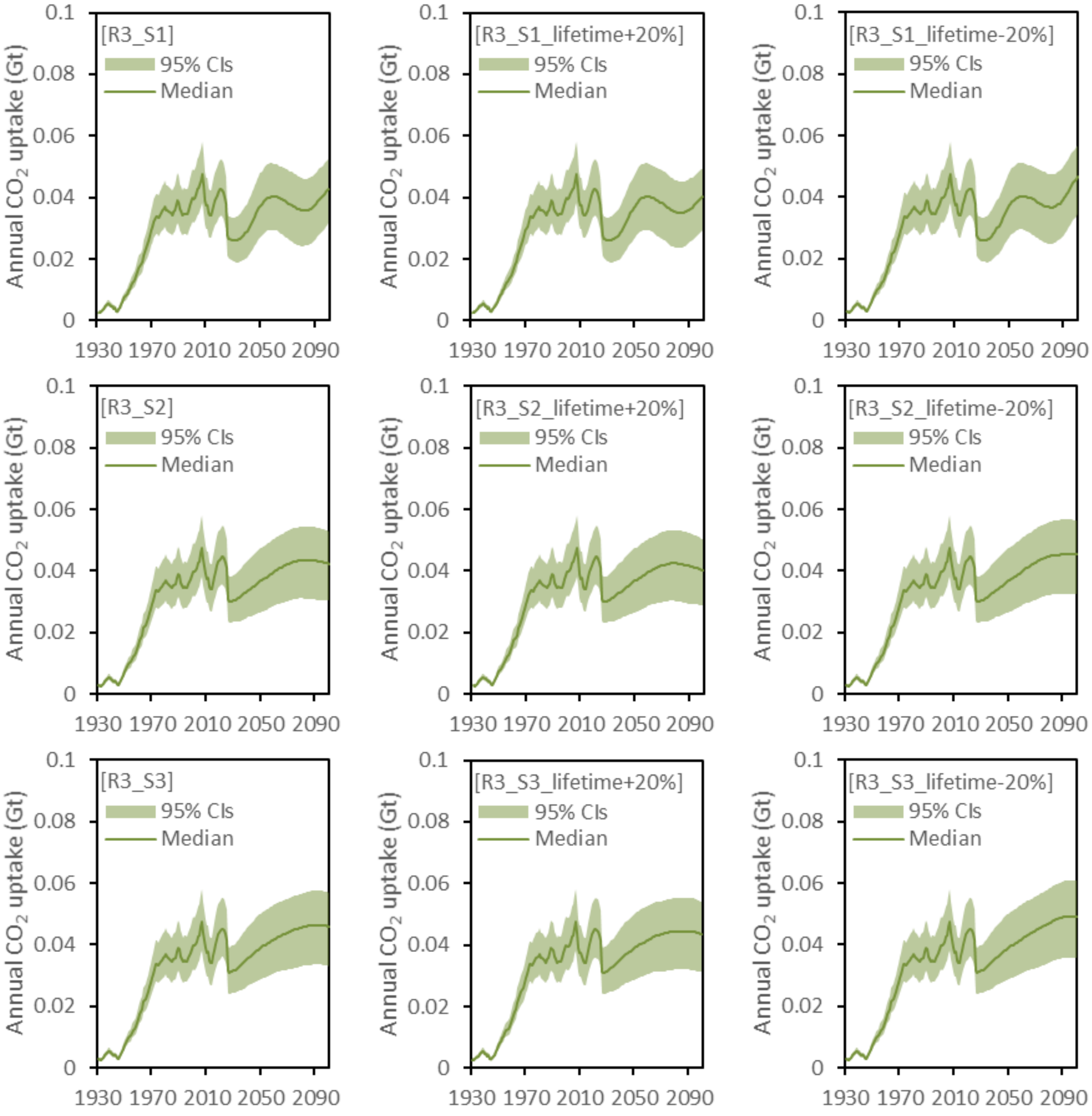

Supplementary Figure 69 | Annual CO_2 uptake in Europe under scenarios S1, S2 and S3, if lifetime is unchanged (left column), increased by 20% (middle column), or decreased by 20% (right column)

Note: The solid lines are the median value of simulated outcomes, and the shaded areas represent the 95% uncertainty range of simulated outcomes. The number of simulation runs is 1,000.

Supplementary Figure 70

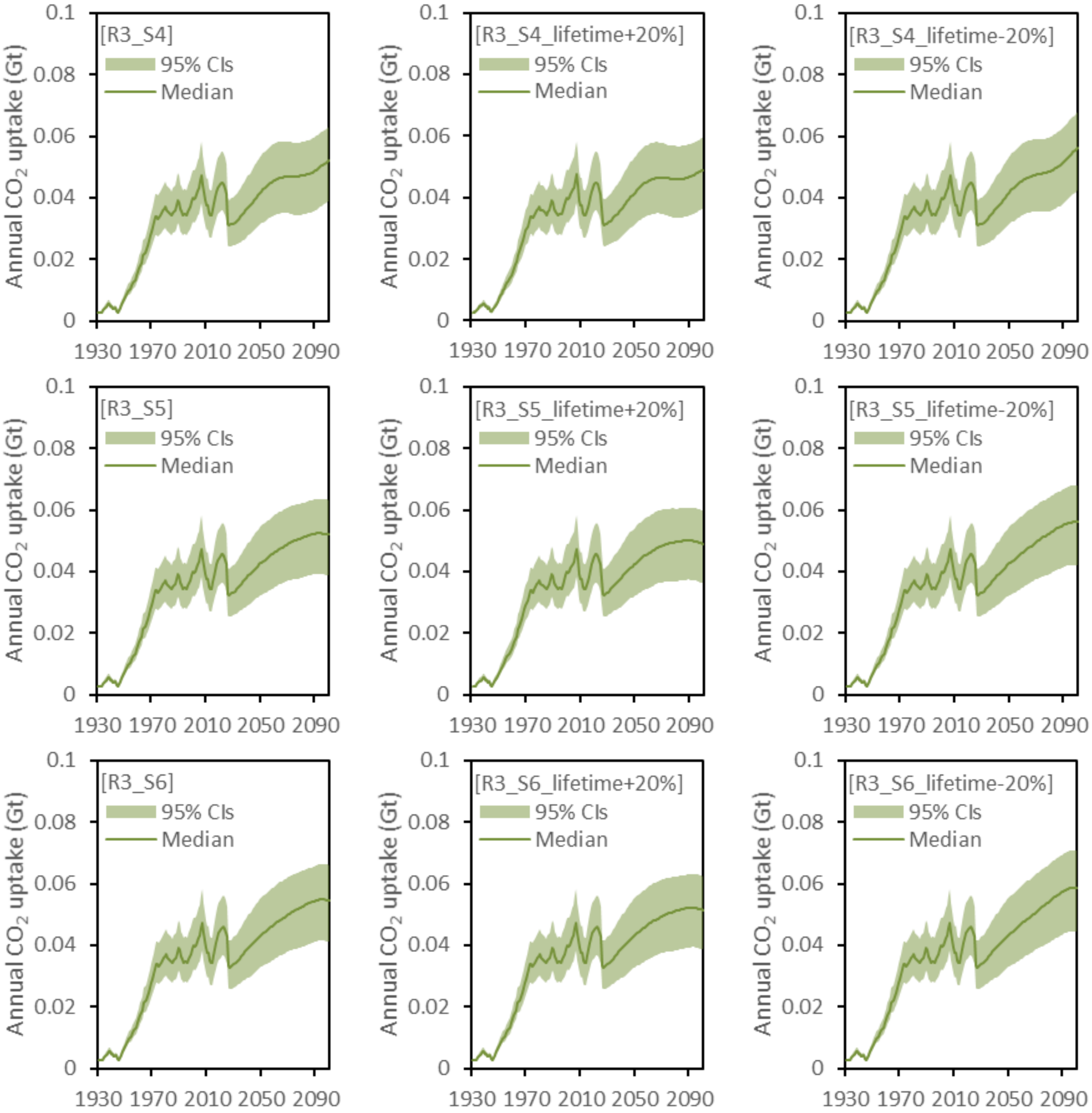

Supplementary Figure 70 | Annual CO_2 uptake in Europe under scenarios S4, S5 and S6, if lifetime is unchanged (left column), increased by 20% (middle column), or decreased by 20% (right column)

Note: The solid lines are the median value of simulated outcomes, and the shaded areas represent the 95% uncertainty range of simulated outcomes. The number of simulation runs is 1,000.

Supplementary Figure 71

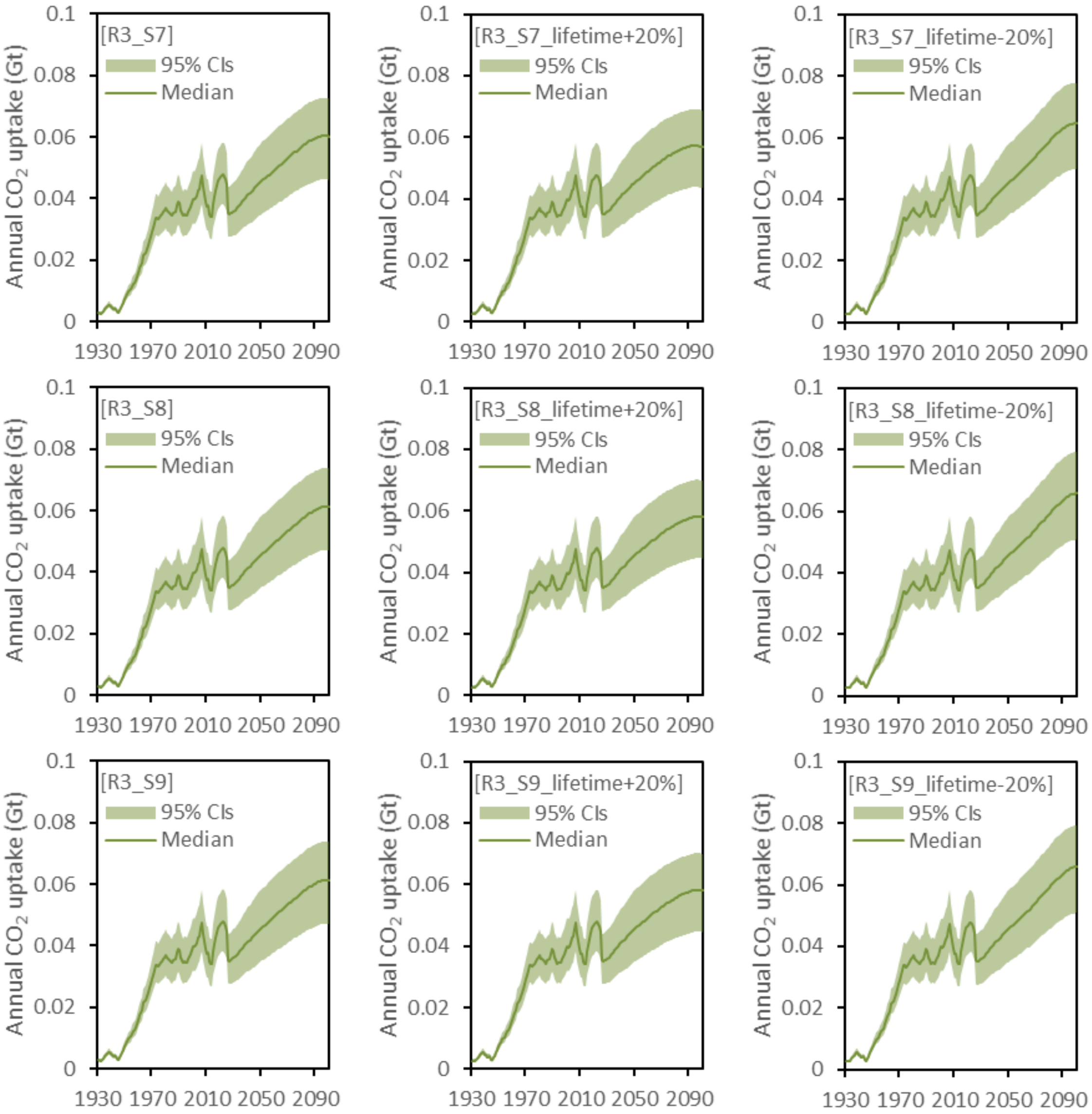

Supplementary Figure 71 | Annual CO_2 uptake in Europe under scenarios S7, S8 and S9, if lifetime is unchanged (left column), increased by 20% (middle column), or decreased by 20% (right column)

Note: The solid lines are the median value of simulated outcomes, and the shaded areas represent the 95% uncertainty range of simulated outcomes. The number of simulation runs is 1,000.

Supplementary Figure 72

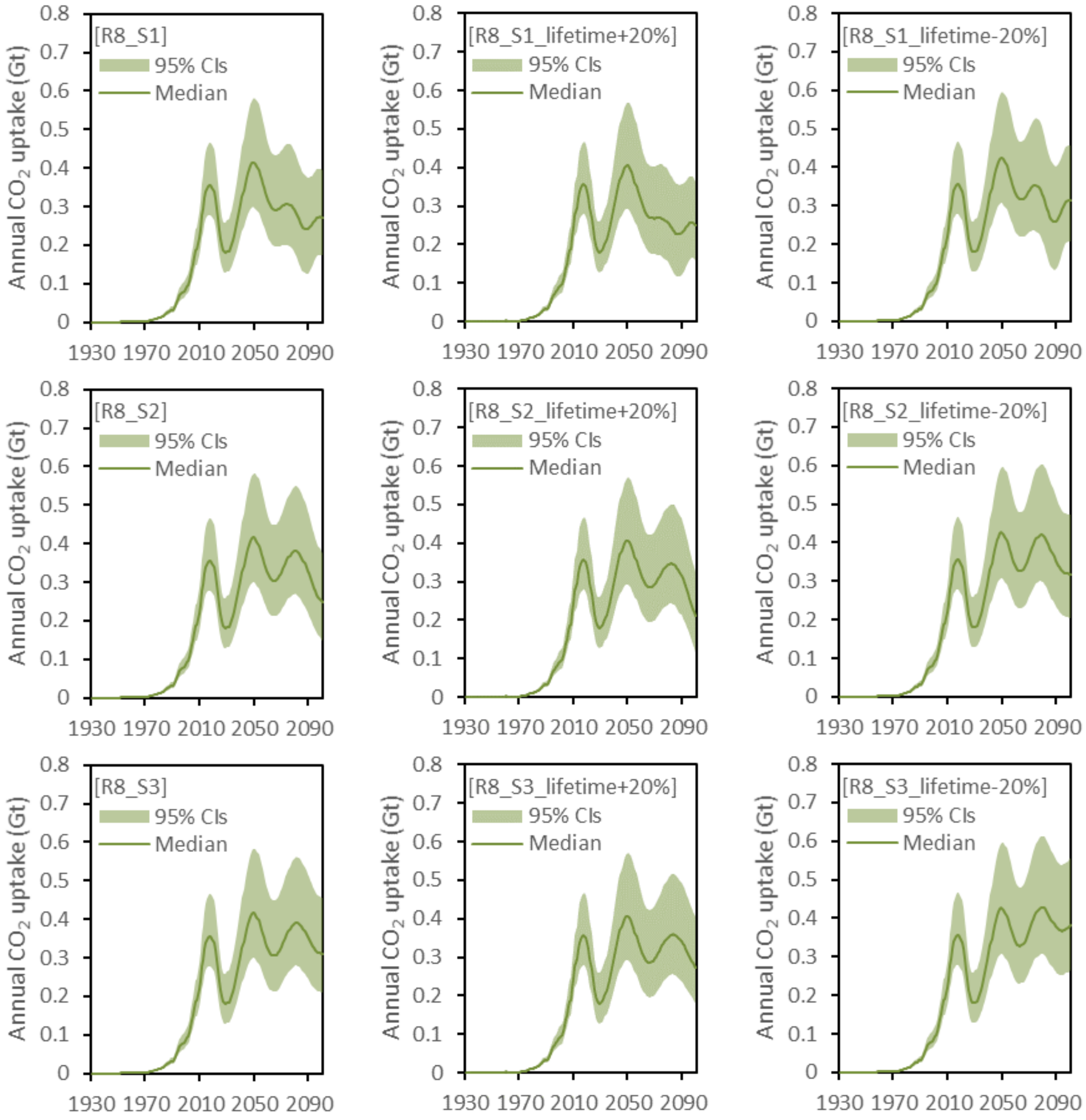

Supplementary Figure 72 | Annual CO_2 uptake in China under scenarios S1, S2 and S3, if lifetime is unchanged (left column), increased by 20% (middle column), or decreased by 20% (right column)

Note: The solid lines are the median value of simulated outcomes, and the shaded areas represent the 95% uncertainty range of simulated outcomes. The number of simulation runs is 1,000.

Supplementary Figure 73

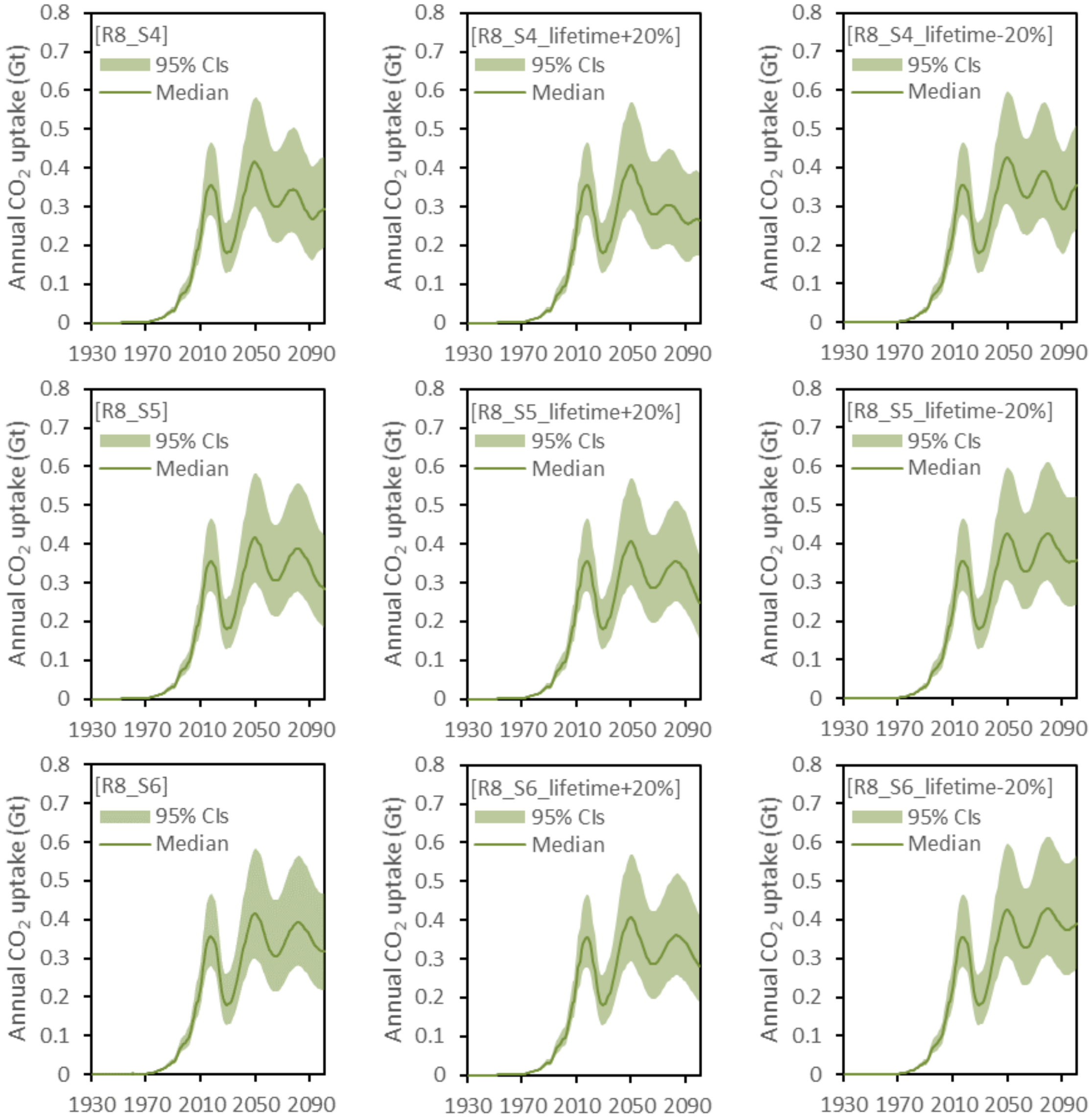

Supplementary Figure 73 | Annual CO_2 uptake in China under scenarios S4, S5 and S6, if lifetime is unchanged (left column), increased by 20% (middle column), or decreased by 20% (right column)

Note: The solid lines are the median value of simulated outcomes, and the shaded areas represent the 95% uncertainty range of simulated outcomes. The number of simulation runs is 1,000.

Supplementary Figure 74

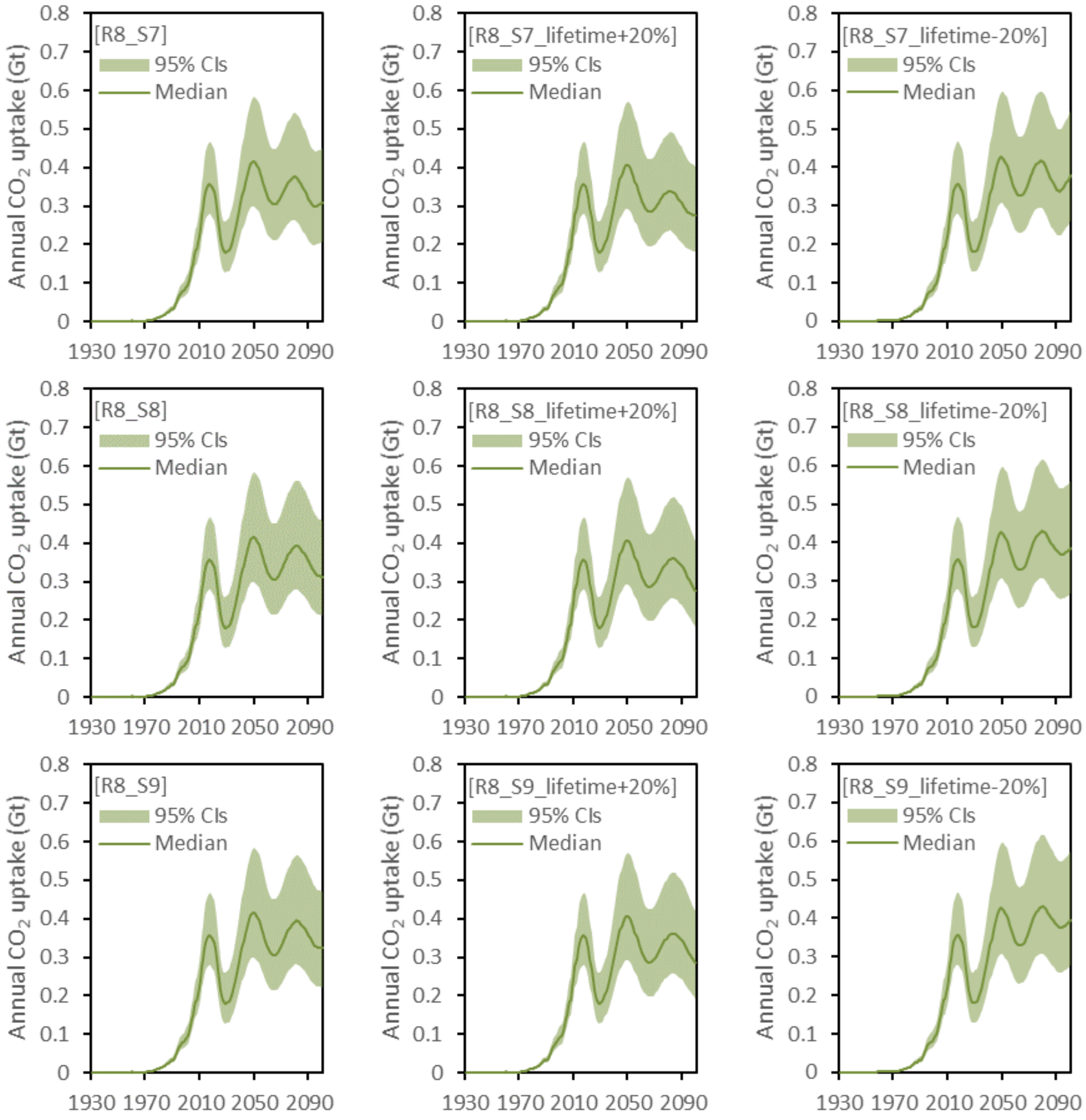

Supplementary Figure 74 | Annual CO_2 uptake in China under scenarios S7, S8 and S9, if lifetime is unchanged (left column), increased by 20% (middle column), or decreased by 20% (right column)

Note: The solid lines are the median value of simulated outcomes, and the shaded areas represent the 95% uncertainty range of simulated outcomes. The number of simulation runs is 1,000.

Supplementary Figure 75

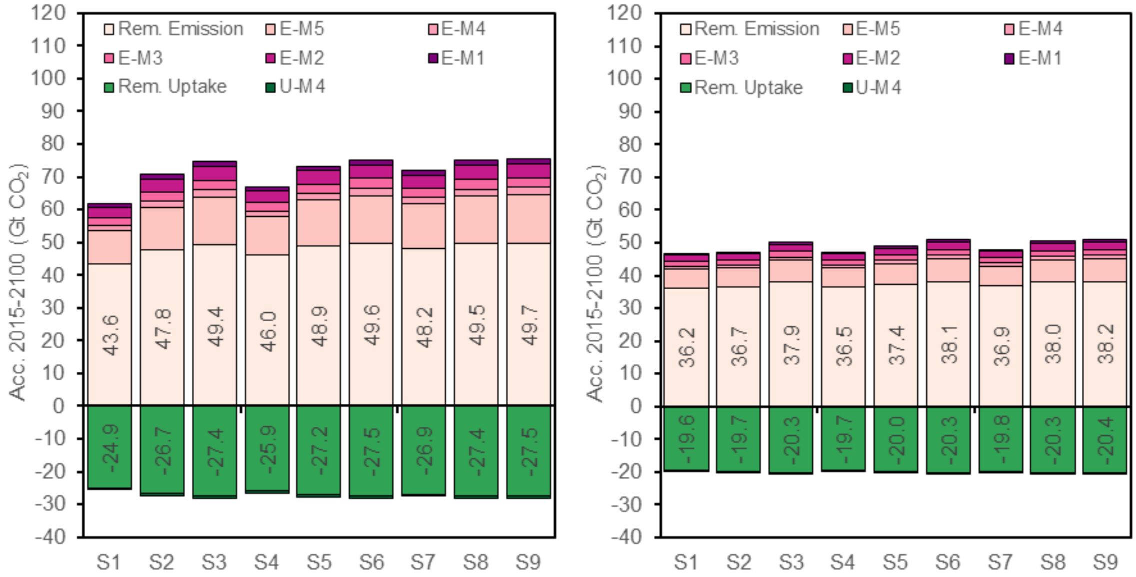

Supplementary Figure 75 | Accumulated mitigation potentials by the five 'supply-side' mitigation measures and accumulated CO_2 uptake without lifetime extended (left) and with lifetime extended (right)

Supplementary Figure 76

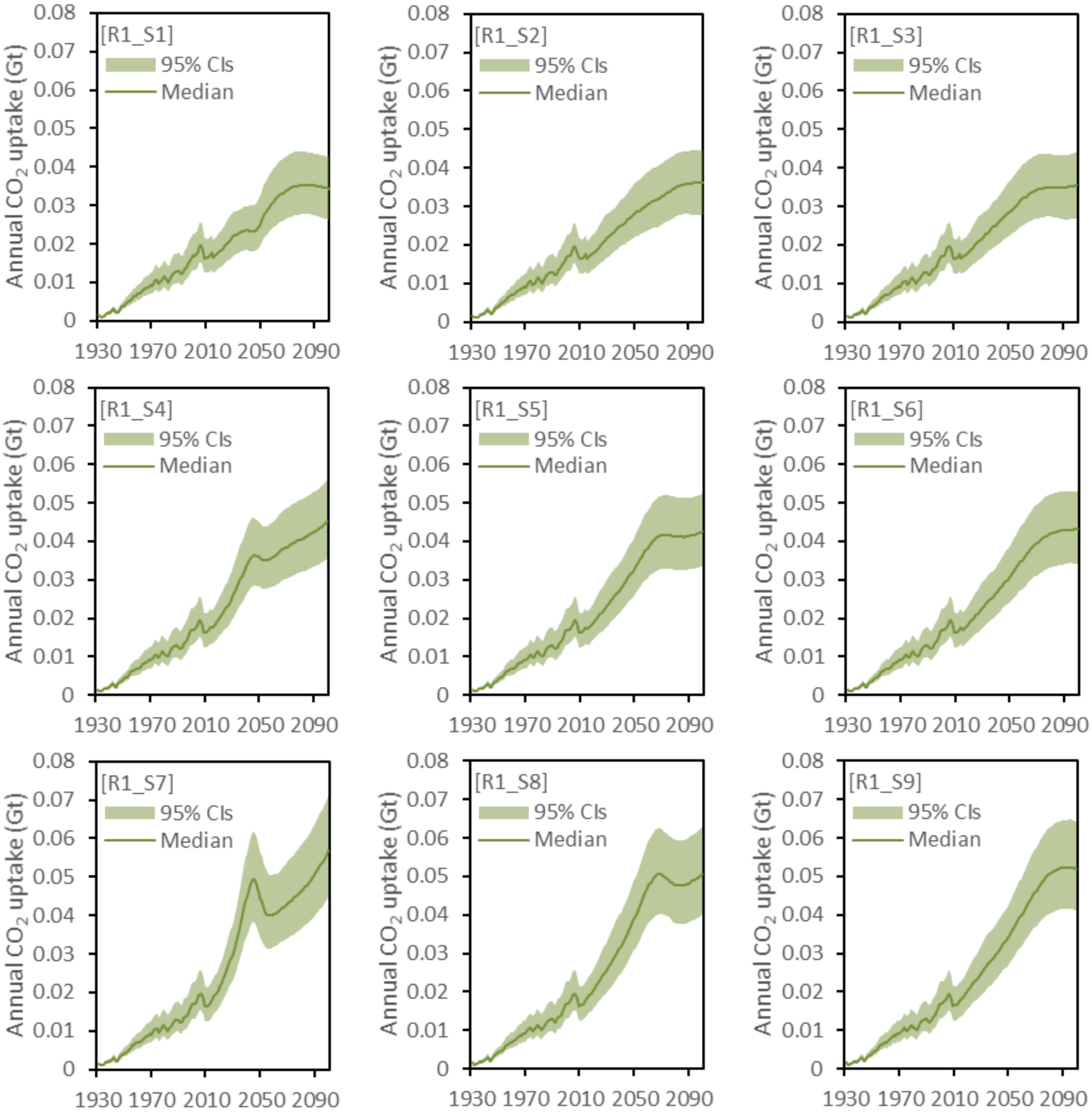

Supplementary Figure 76 | Uncertainties of simulated annual CO_2 uptake in North America

Note: The solid lines are the median value of simulated outcomes, and the shaded areas represent the 95% uncertainty range of simulated outcomes. The number of simulation runs is 1,000.

Supplementary Figure 77

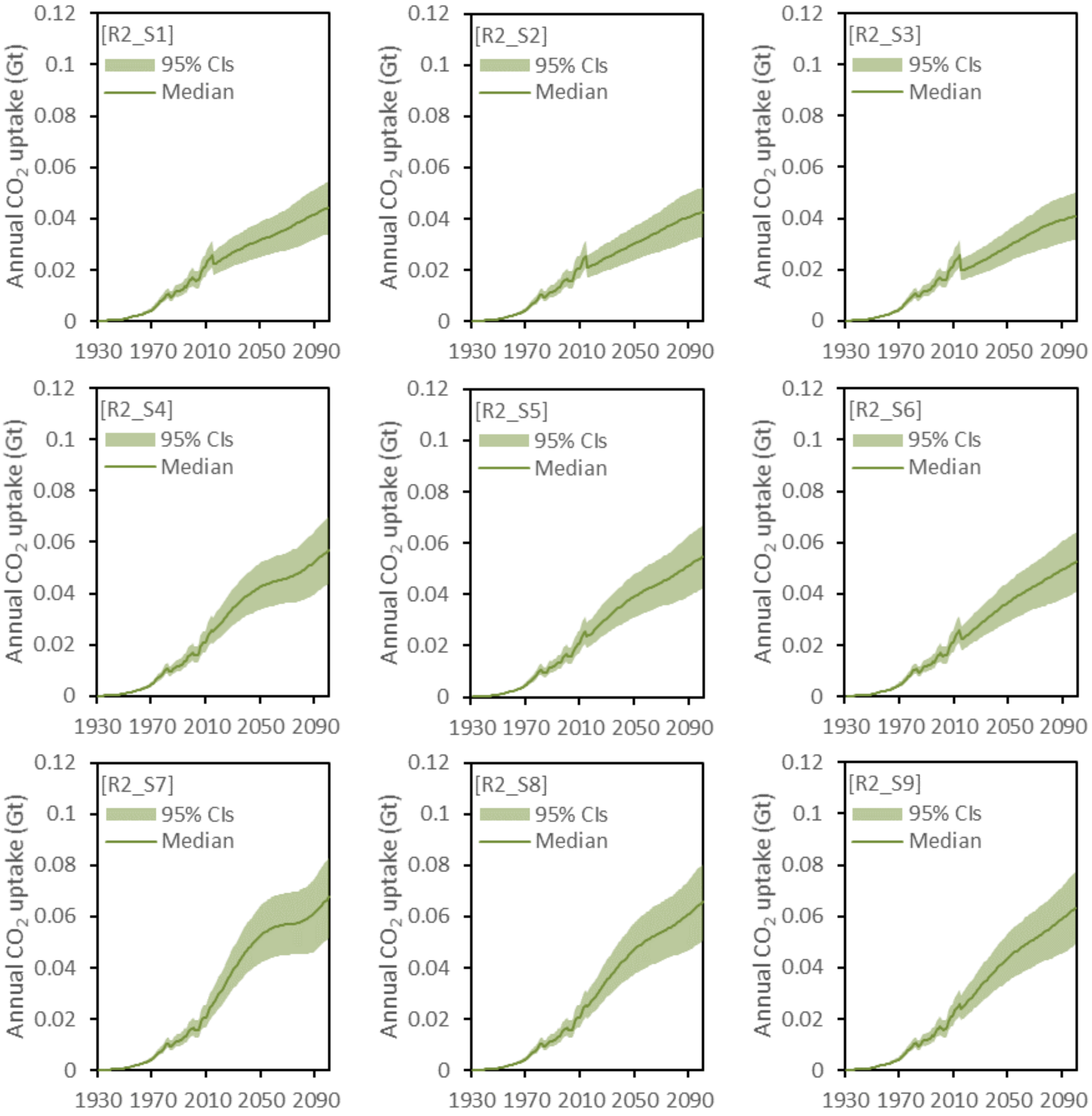

Supplementary Figure 77 | Uncertainties of simulated annual CO_2 uptake in Latin America & Caribbean

Note: The solid lines are the median value of simulated outcomes, and the shaded areas represent the 95% uncertainty range of simulated outcomes. The number of simulation runs is 1,000.

Supplementary Figure 78

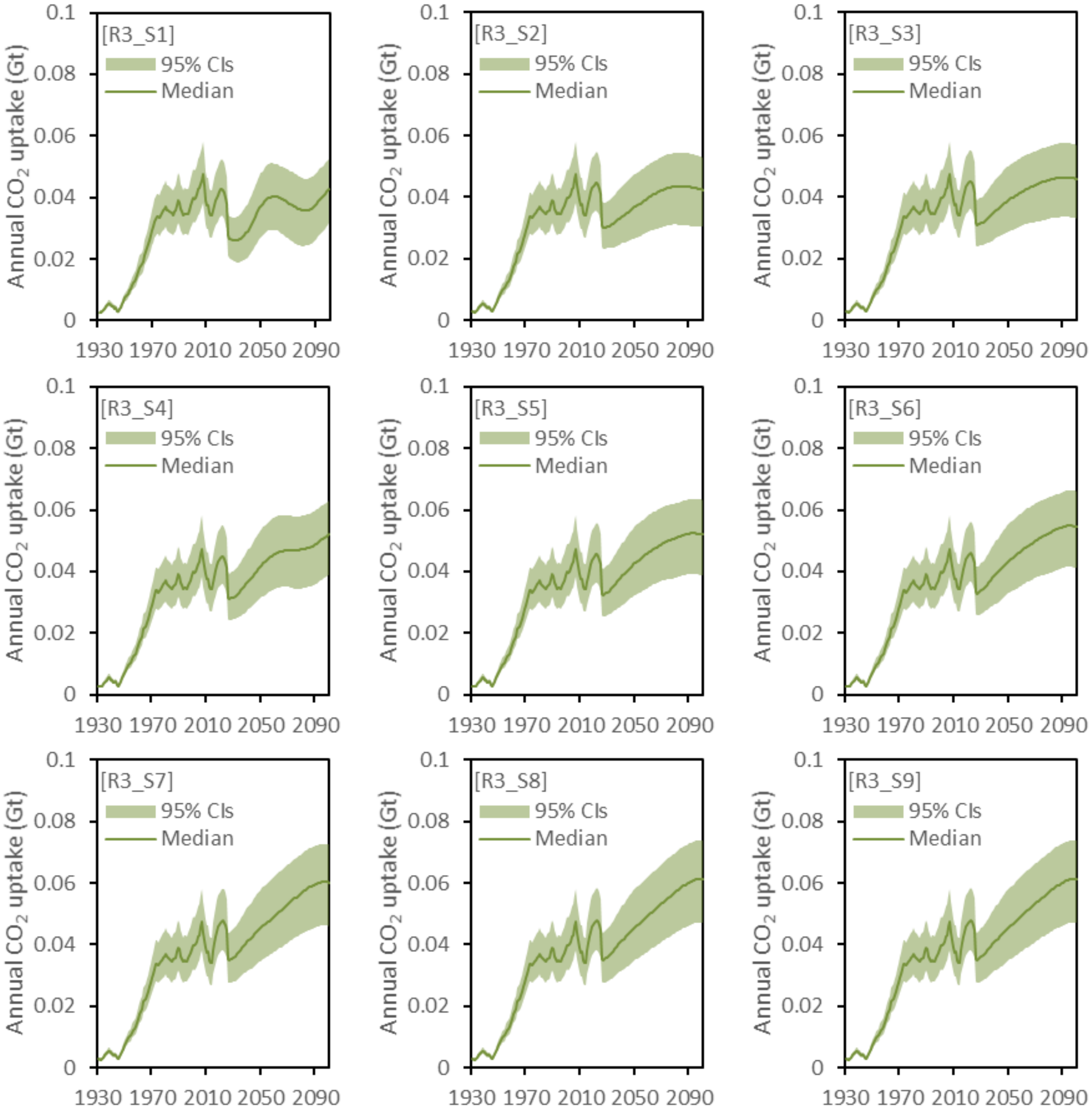

Supplementary Figure 78 | Uncertainties of simulated annual CO_2 uptake in Europe

Note: The solid lines are the median value of simulated outcomes, and the shaded areas represent the 95% uncertainty range of simulated outcomes. The number of simulation runs is 1,000.

Supplementary Figure 79

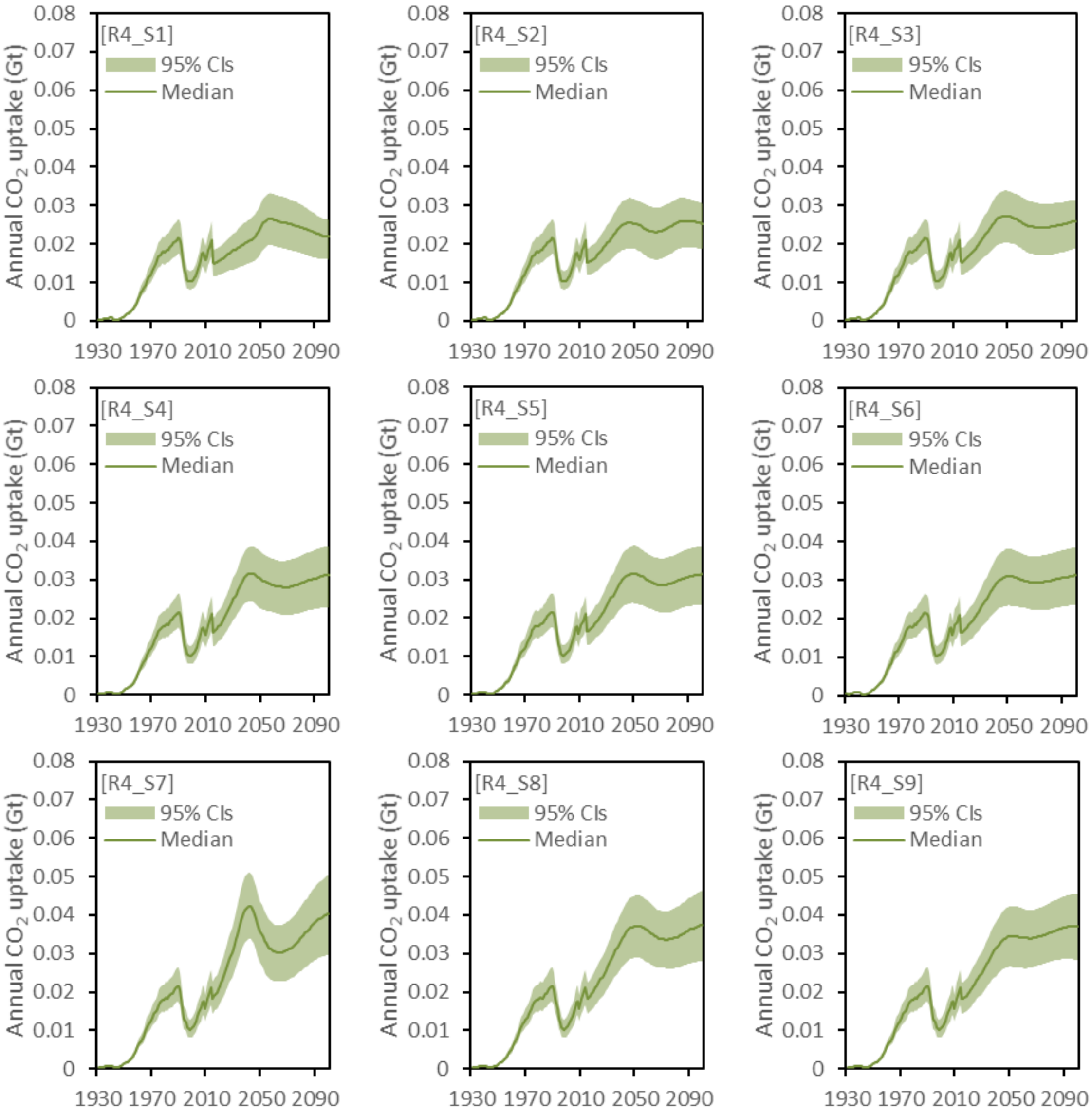

Supplementary Figure 79 | Uncertainties of simulated annual CO_2 uptake in Commonwealth of Independent States

Note: The solid lines are the median value of simulated outcomes, and the shaded areas represent the 95% uncertainty range of simulated outcomes. The number of simulation runs is 1,000.

Supplementary Figure 80

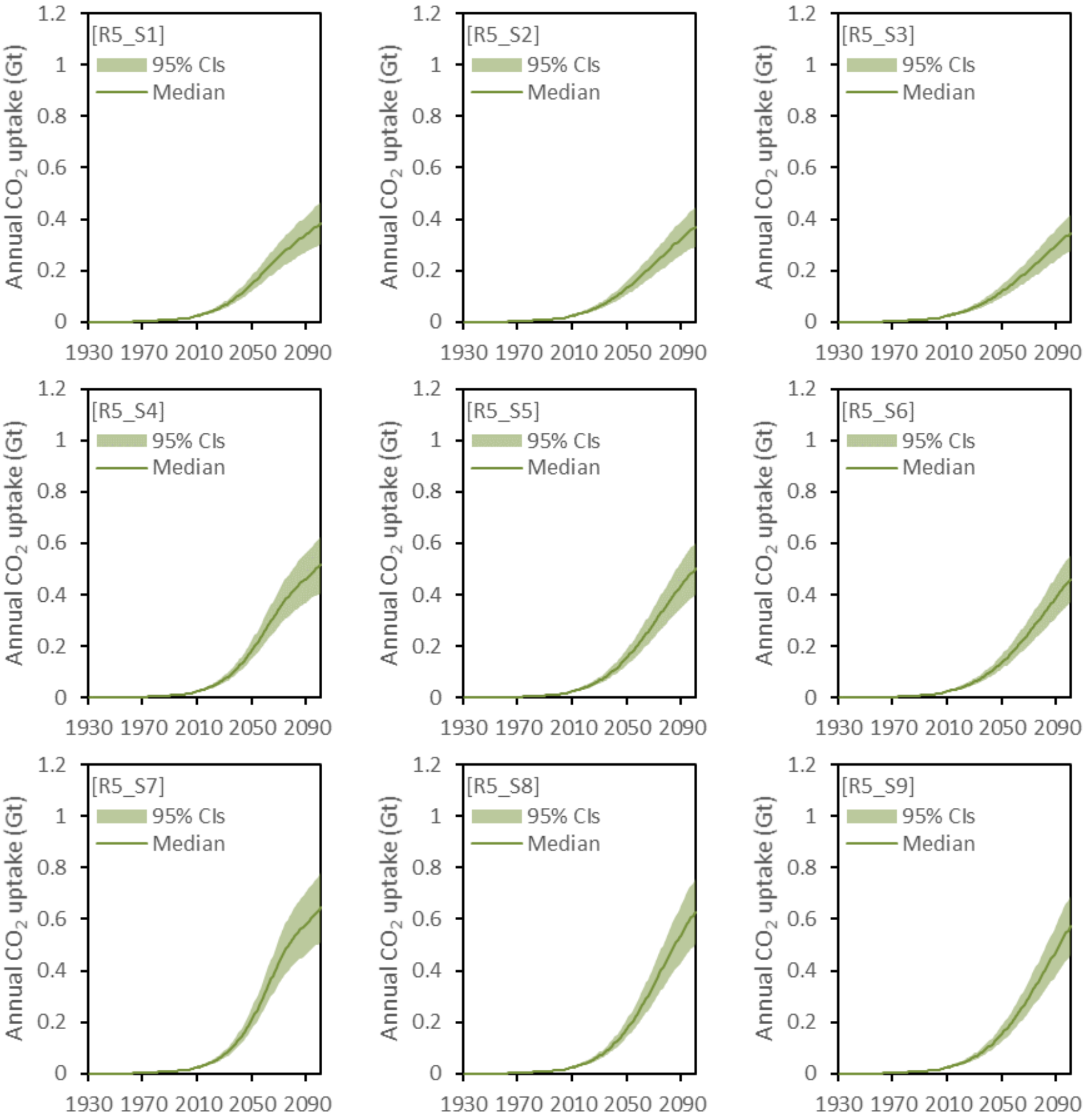

Supplementary Figure 80 | Uncertainties of simulated annual CO_2 uptake in Africa

Note: The solid lines are the median value of simulated outcomes, and the shaded areas represent the 95% uncertainty range of simulated outcomes. The number of simulation runs is 1,000.

Supplementary Figure 81

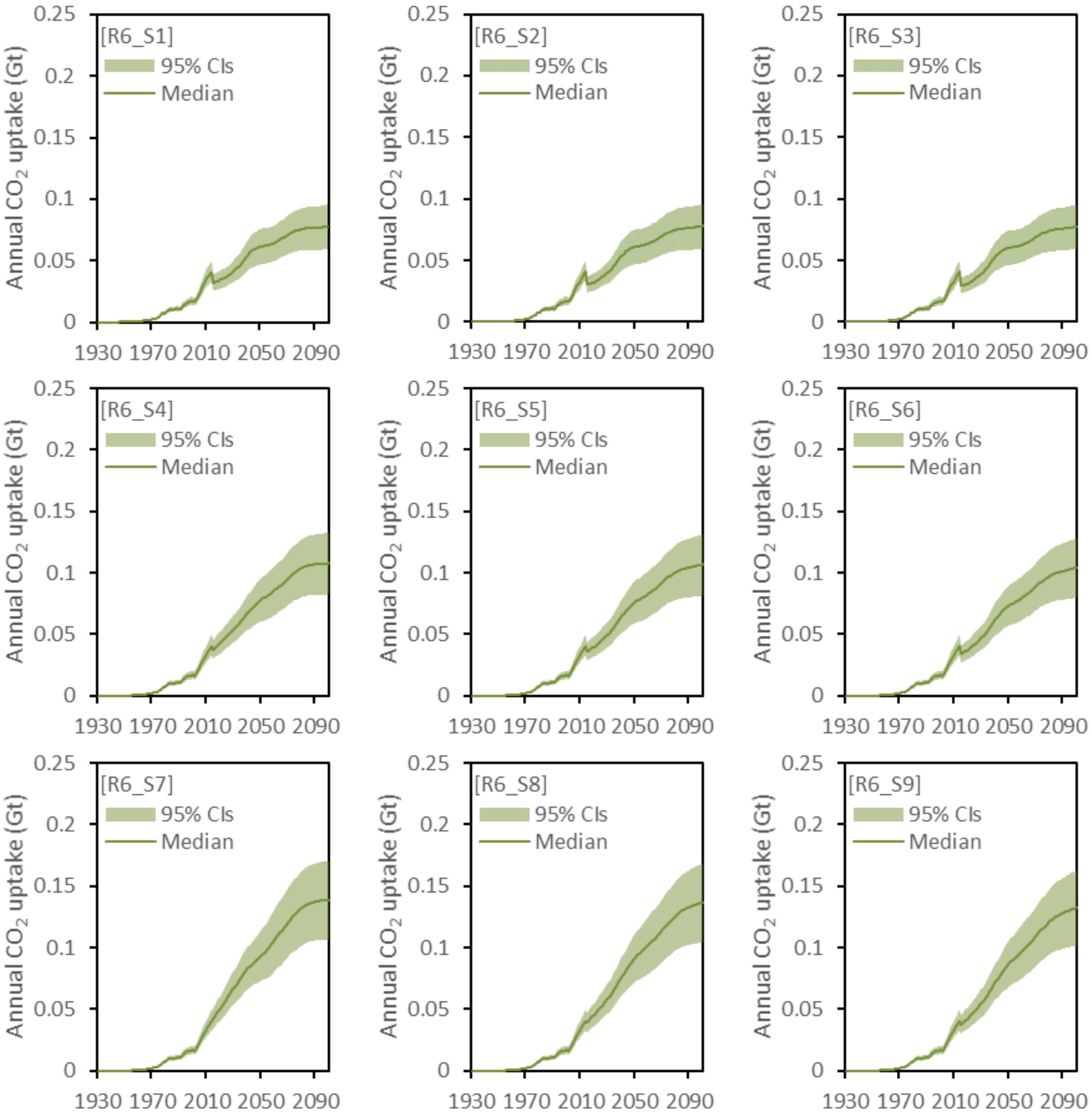

Supplementary Figure 81 | Uncertainties of simulated annual CO_2 uptake in Middle East

Note: The solid lines are the median value of simulated outcomes, and the shaded areas represent the 95% uncertainty range of simulated outcomes. The number of simulation runs is 1,000.

Supplementary Figure 82

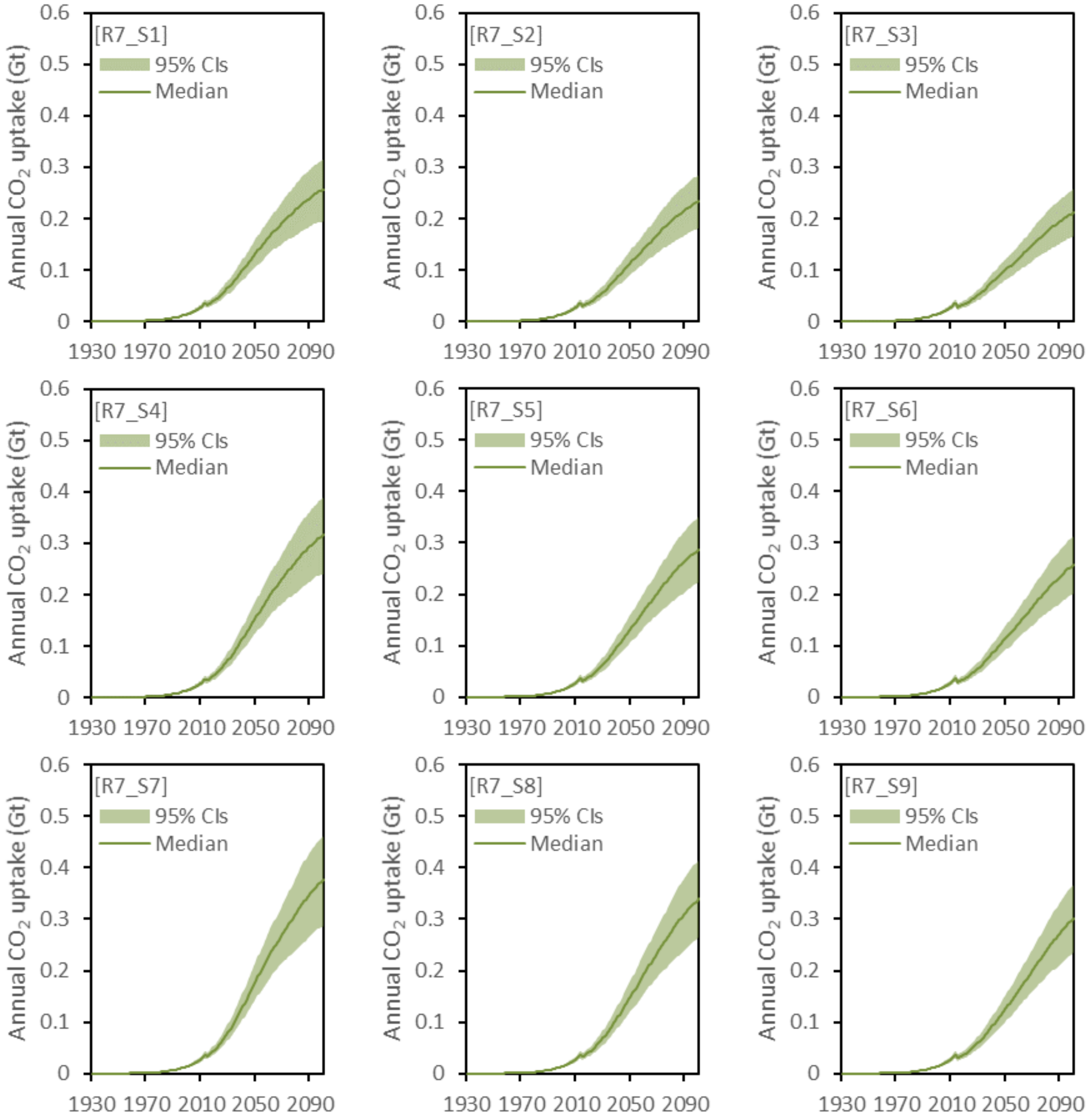

Supplementary Figure 82 | Uncertainties of simulated annual CO_2 uptake in India

Note: The solid lines are the median value of simulated outcomes, and the shaded areas represent the 95% uncertainty range of simulated outcomes. The number of simulation runs is 1,000.

Supplementary Figure 83

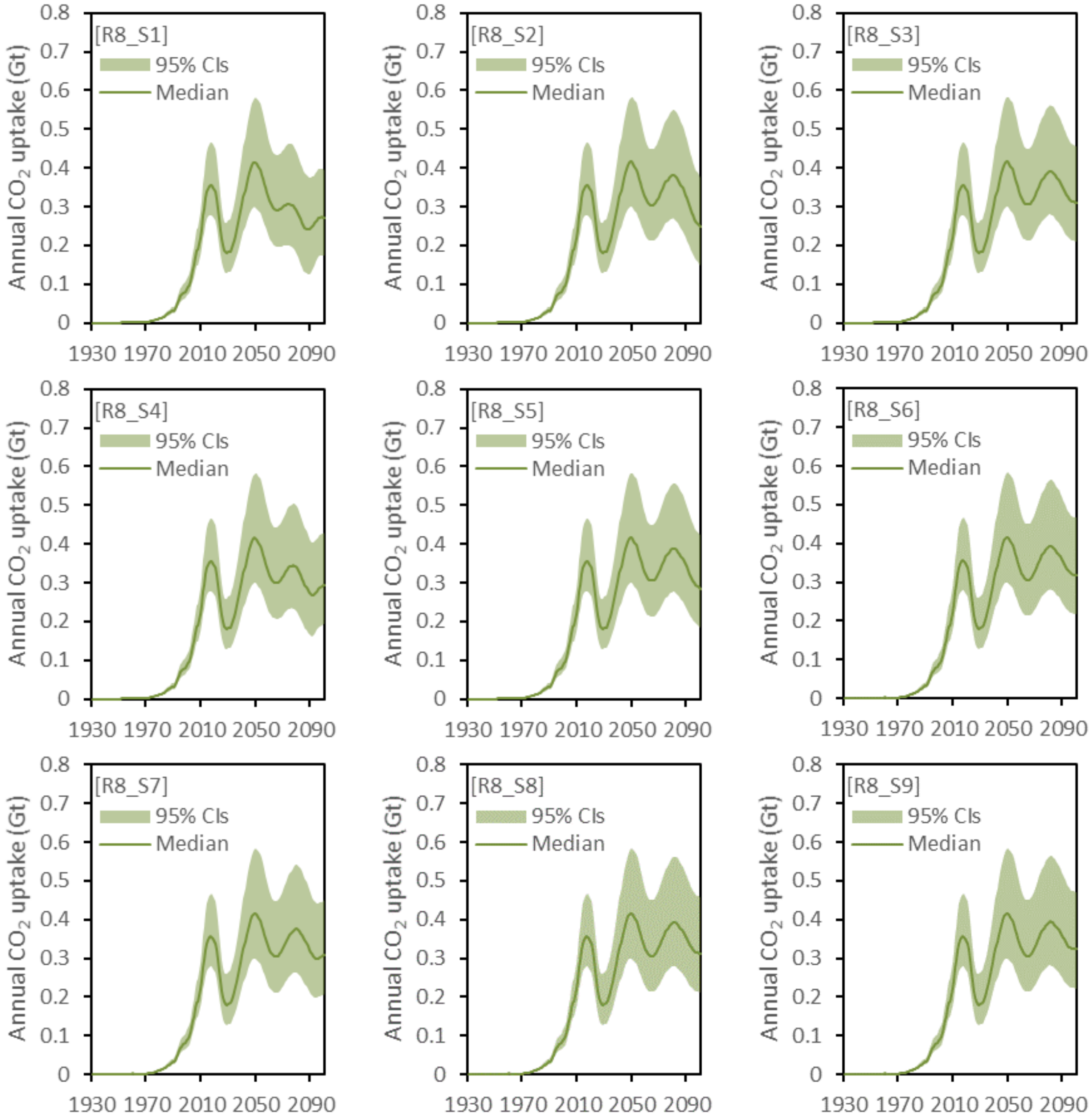

Supplementary Figure 83 | Uncertainties of simulated annual CO_2 uptake in China

Note: The solid lines are the median value of simulated outcomes, and the shaded areas represent the 95% uncertainty range of simulated outcomes. The number of simulation runs is 1,000.

Supplementary Figure 84

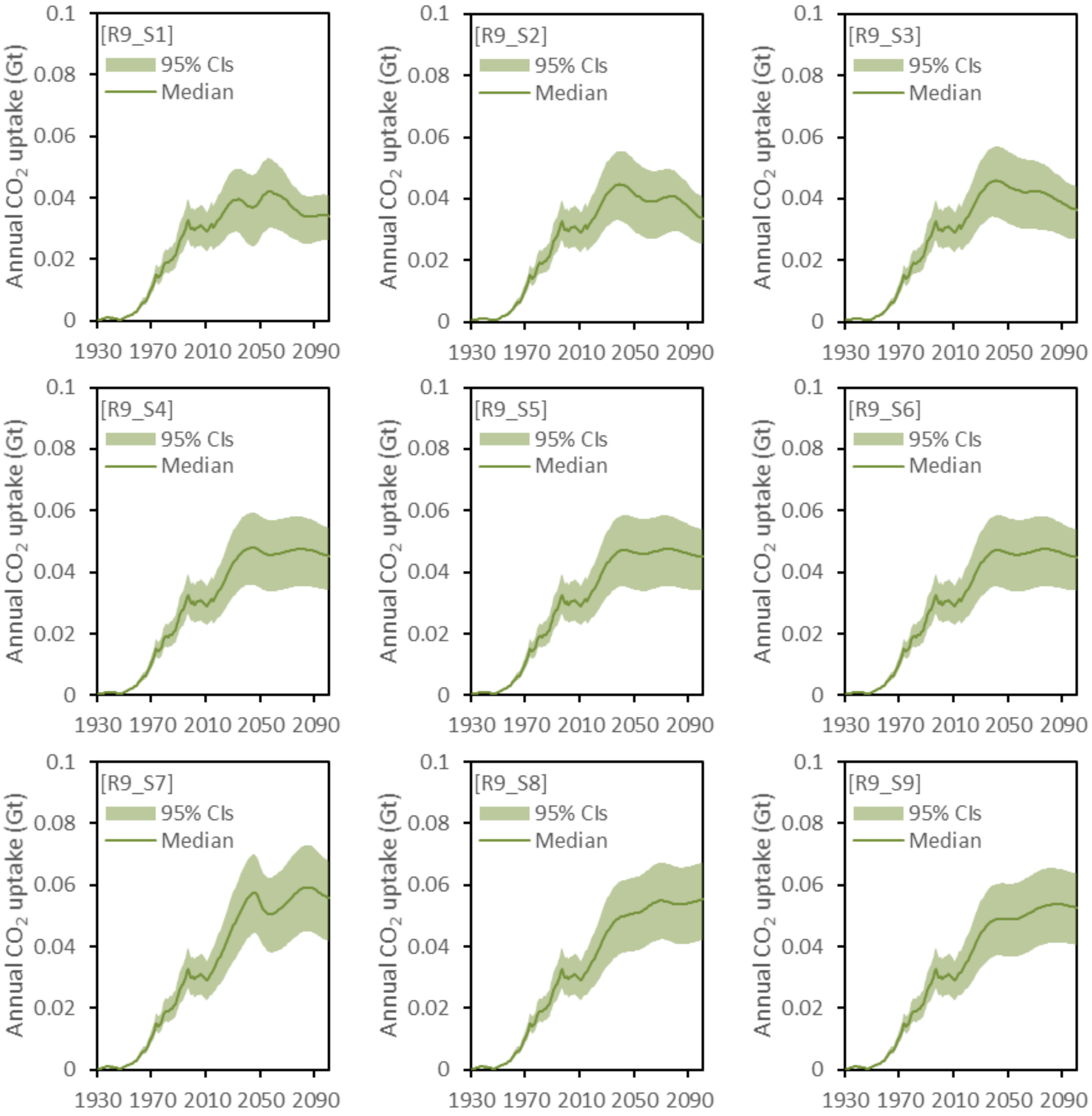

Supplementary Figure 84 | Uncertainties of simulated annual CO_2 uptake in Developed Asia & Oceania

Note: The solid lines are the median value of simulated outcomes, and the shaded areas represent the 95% uncertainty range of simulated outcomes. The number of simulation runs is 1,000.

Supplementary Figure 85

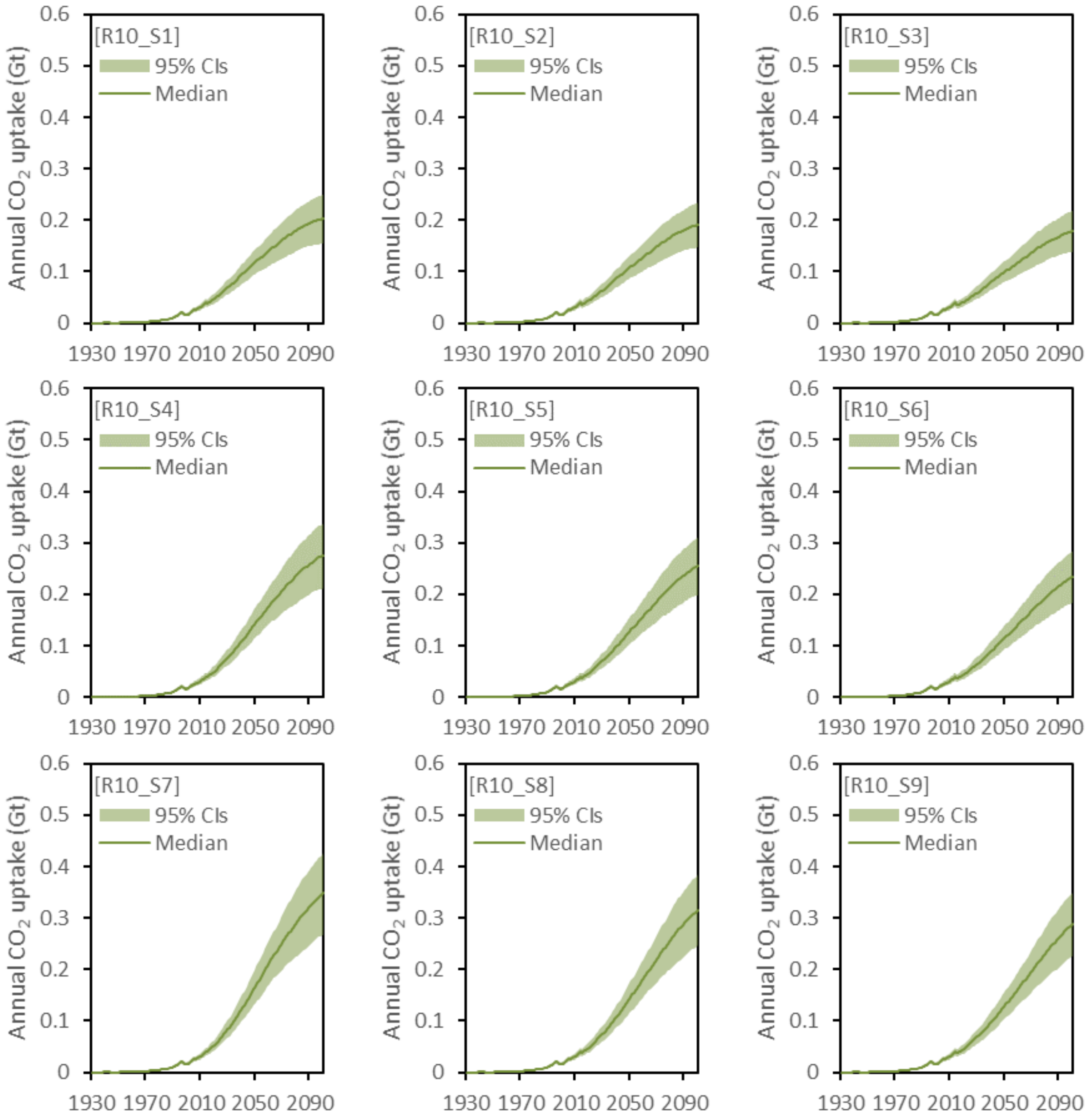

Supplementary Figure 85 | Uncertainties of simulated annual CO_2 uptake in Developing Asia

Note: The solid lines are the median value of simulated outcomes, and the shaded areas represent the 95% uncertainty range of simulated outcomes. The number of simulation runs is 1,000.

Supplementary Figure 86

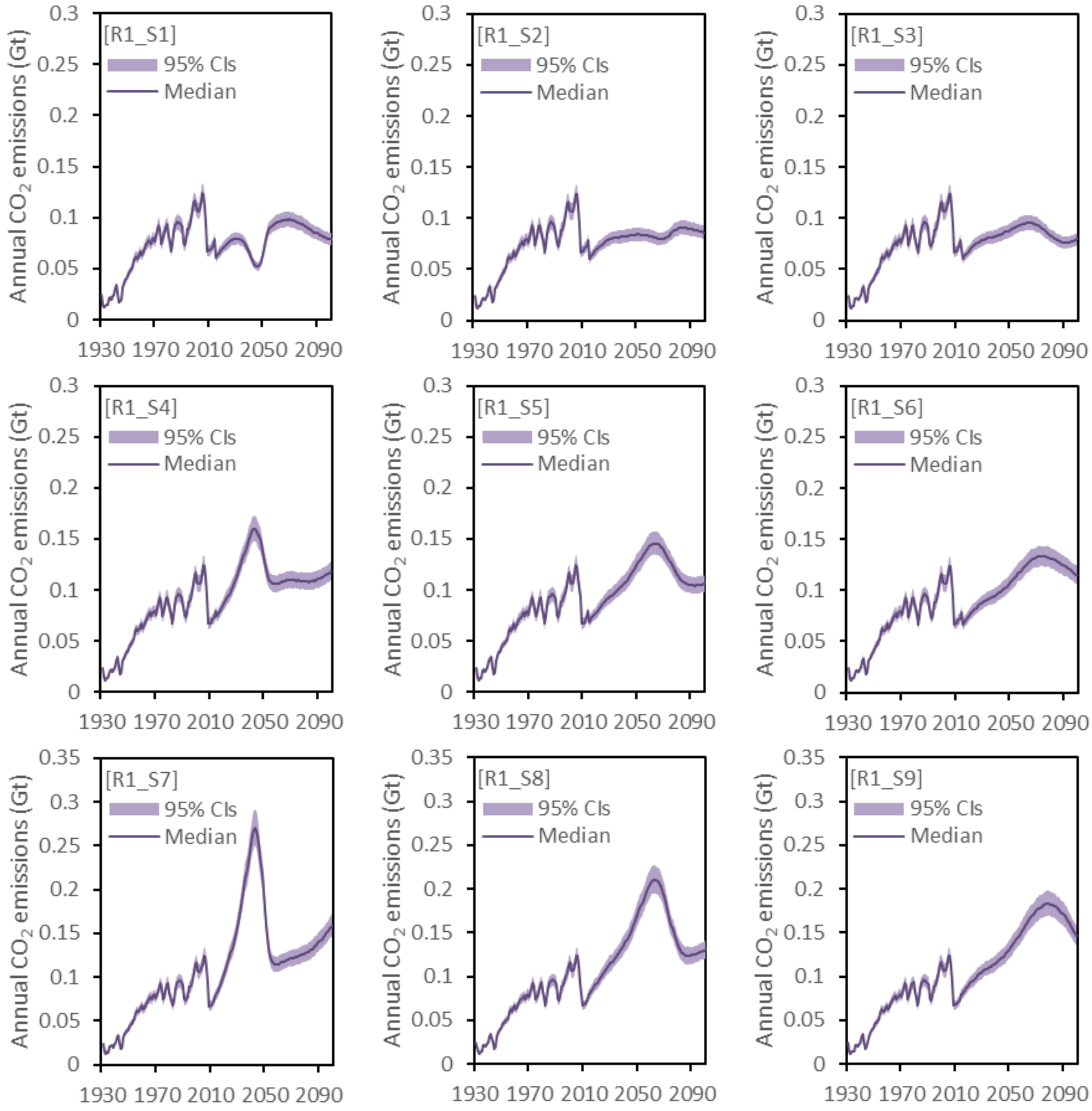

Supplementary Figure 86 | Uncertainties of annual CO_2 emissions in North America

Note: The solid lines are the median value of simulated outcomes, and the shaded areas represent the 95% uncertainty range of simulated outcomes. The number of simulation runs is 1,000.

Supplementary Figure 87

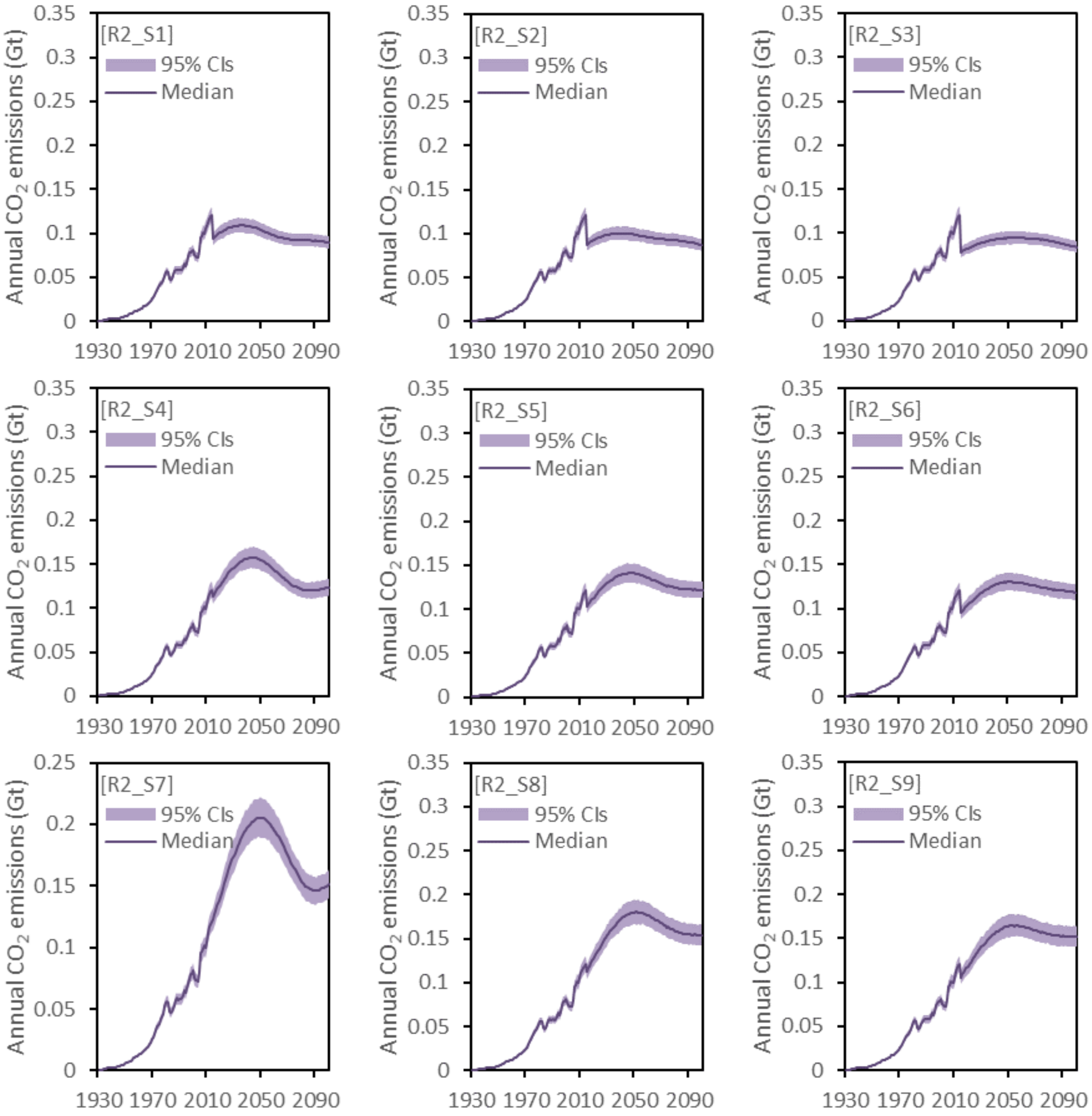

Supplementary Figure 87 | Uncertainties of annual CO_2 emissions in Latin America & Caribbean

Note: The solid lines are the median value of simulated outcomes, and the shaded areas represent the 95% uncertainty range of simulated outcomes. The number of simulation runs is 1,000.

Supplementary Figure 88

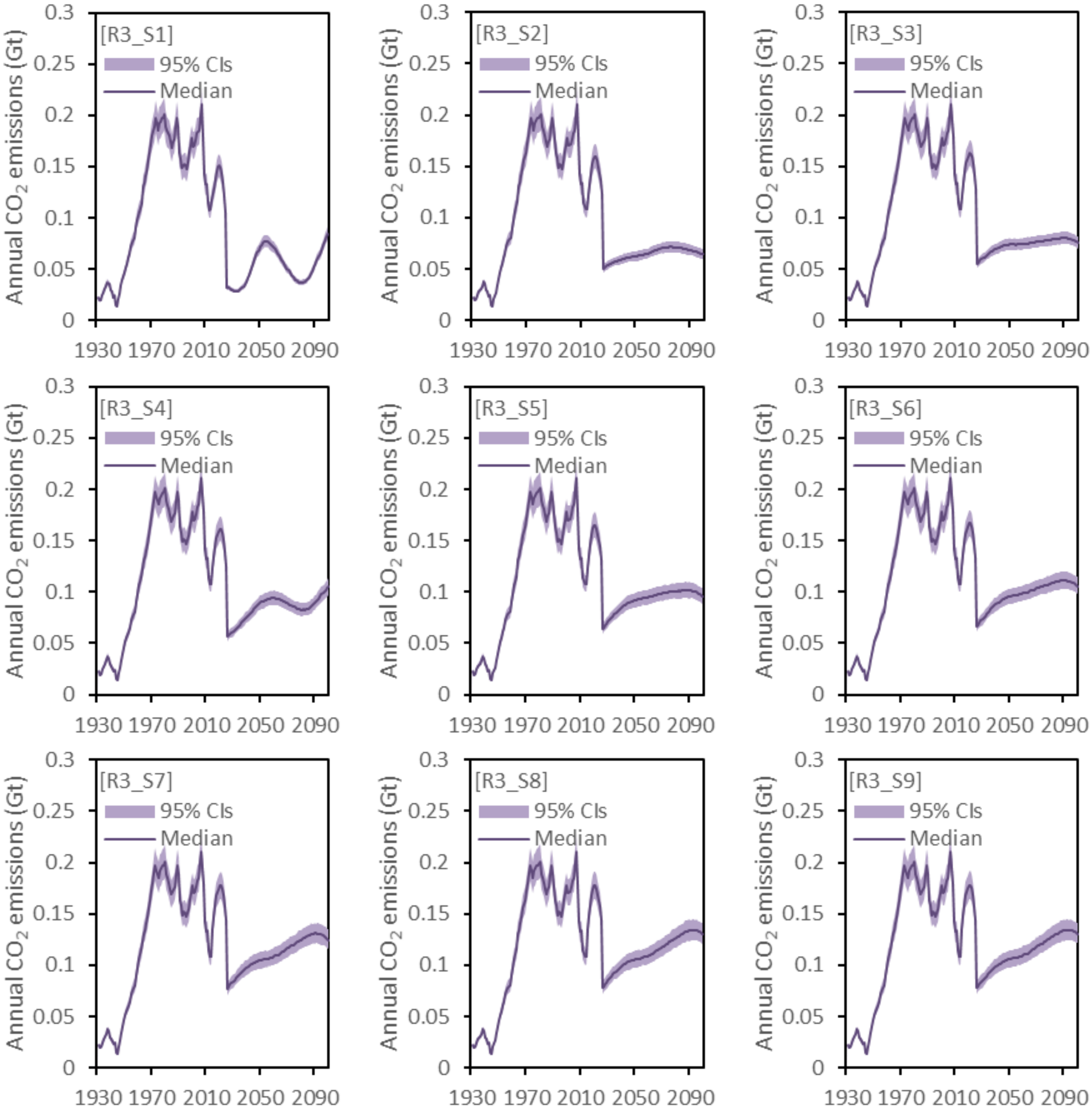

Supplementary Figure 88 | Uncertainties of annual CO_2 emissions in Europe

Note: The solid lines are the median value of simulated outcomes, and the shaded areas represent the 95% uncertainty range of simulated outcomes. The number of simulation runs is 1,000.

Supplementary Figure 89

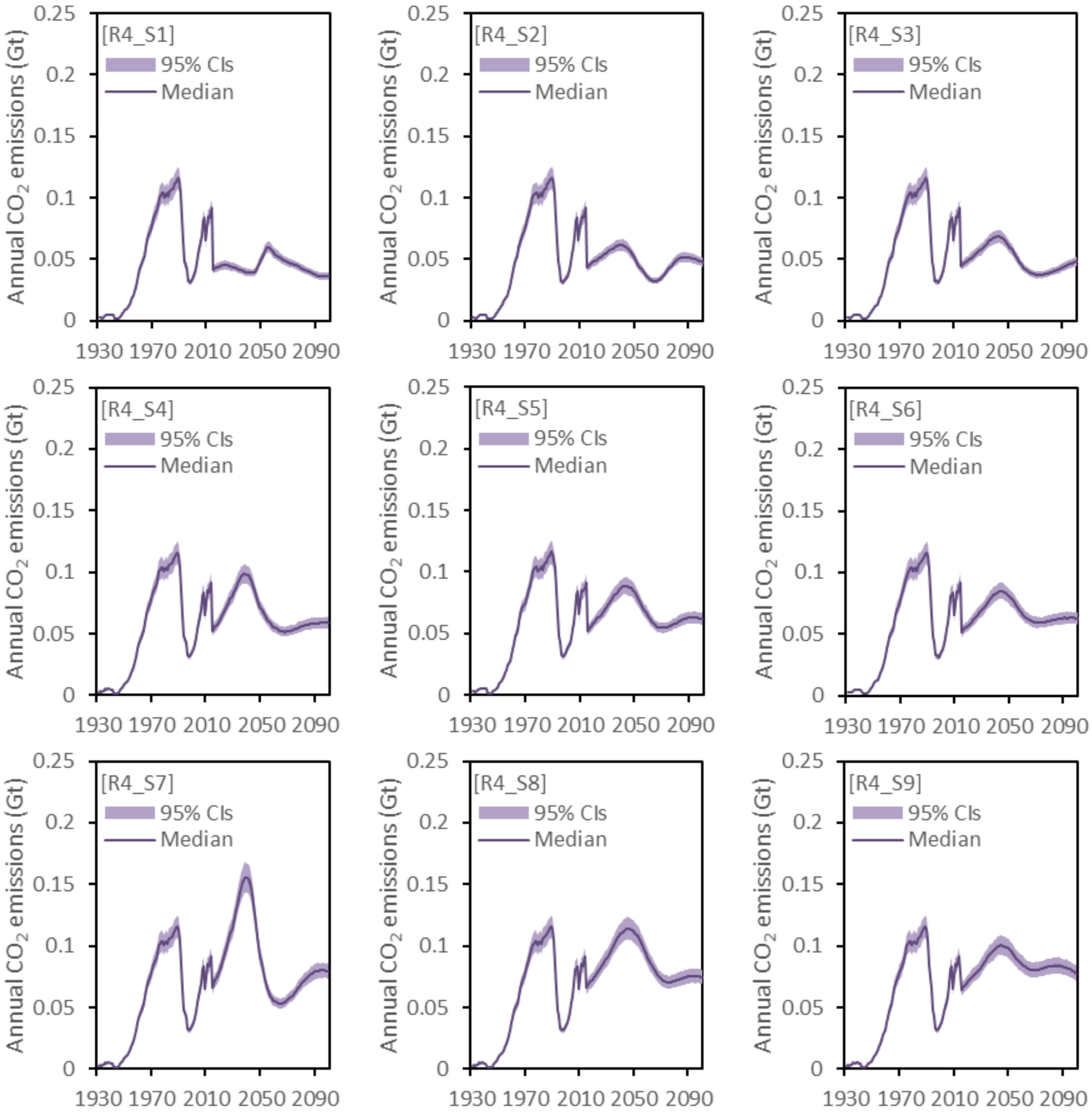

Supplementary Figure 89 | Uncertainties of annual CO_2 emissions in Commonwealth of Independent States

Note: The solid lines are the median value of simulated outcomes, and the shaded areas represent the 95% uncertainty range of simulated outcomes. The number of simulation runs is 1,000.

Supplementary Figure 90

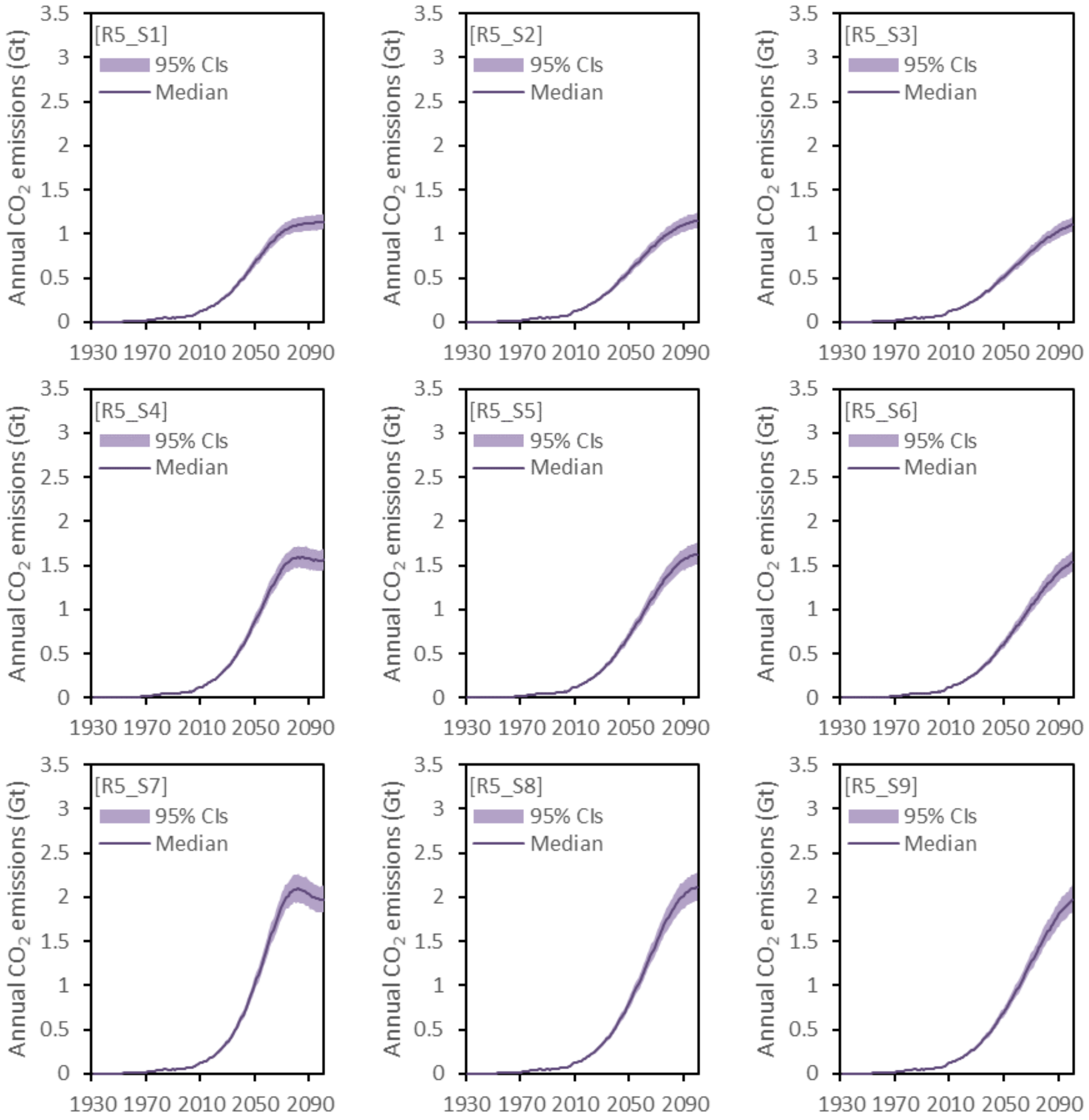

Supplementary Figure 90 | Uncertainties of annual CO_2 emissions in Africa

Note: The solid lines are the median value of simulated outcomes, and the shaded areas represent the 95% uncertainty range of simulated outcomes. The number of simulation runs is 1,000.

Supplementary Figure 91

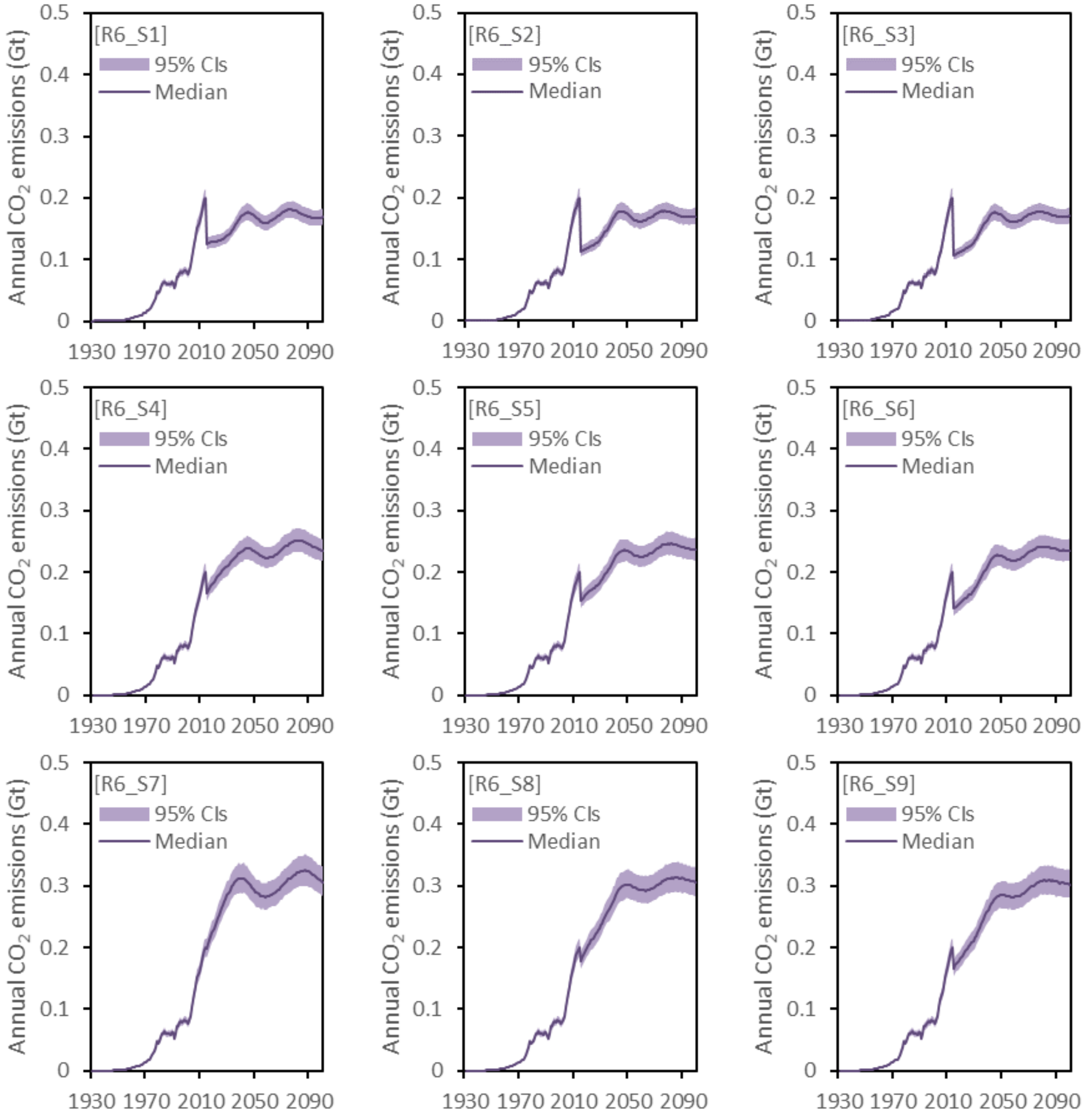

Supplementary Figure 91 | Uncertainties of annual CO_2 emissions in Middle East

Note: The solid lines are the median value of simulated outcomes, and the shaded areas represent the 95% uncertainty range of simulated outcomes. The number of simulation runs is 1,000.

Supplementary Figure 92

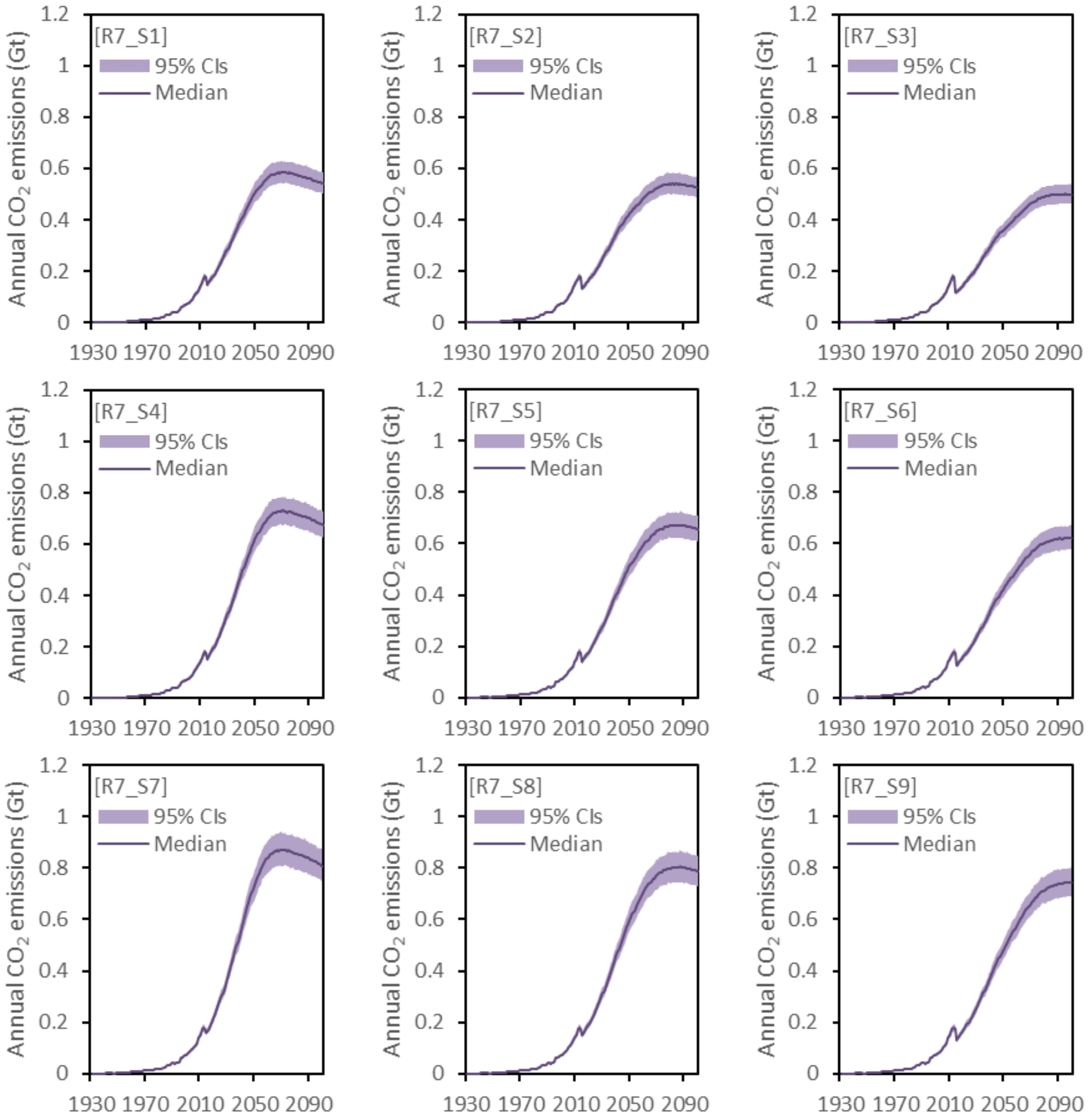

Supplementary Figure 92 | Uncertainties of annual CO_2 emissions in India

Note: The solid lines are the median value of simulated outcomes, and the shaded areas represent the 95% uncertainty range of simulated outcomes. The number of simulation runs is 1,000.

Supplementary Figure 93

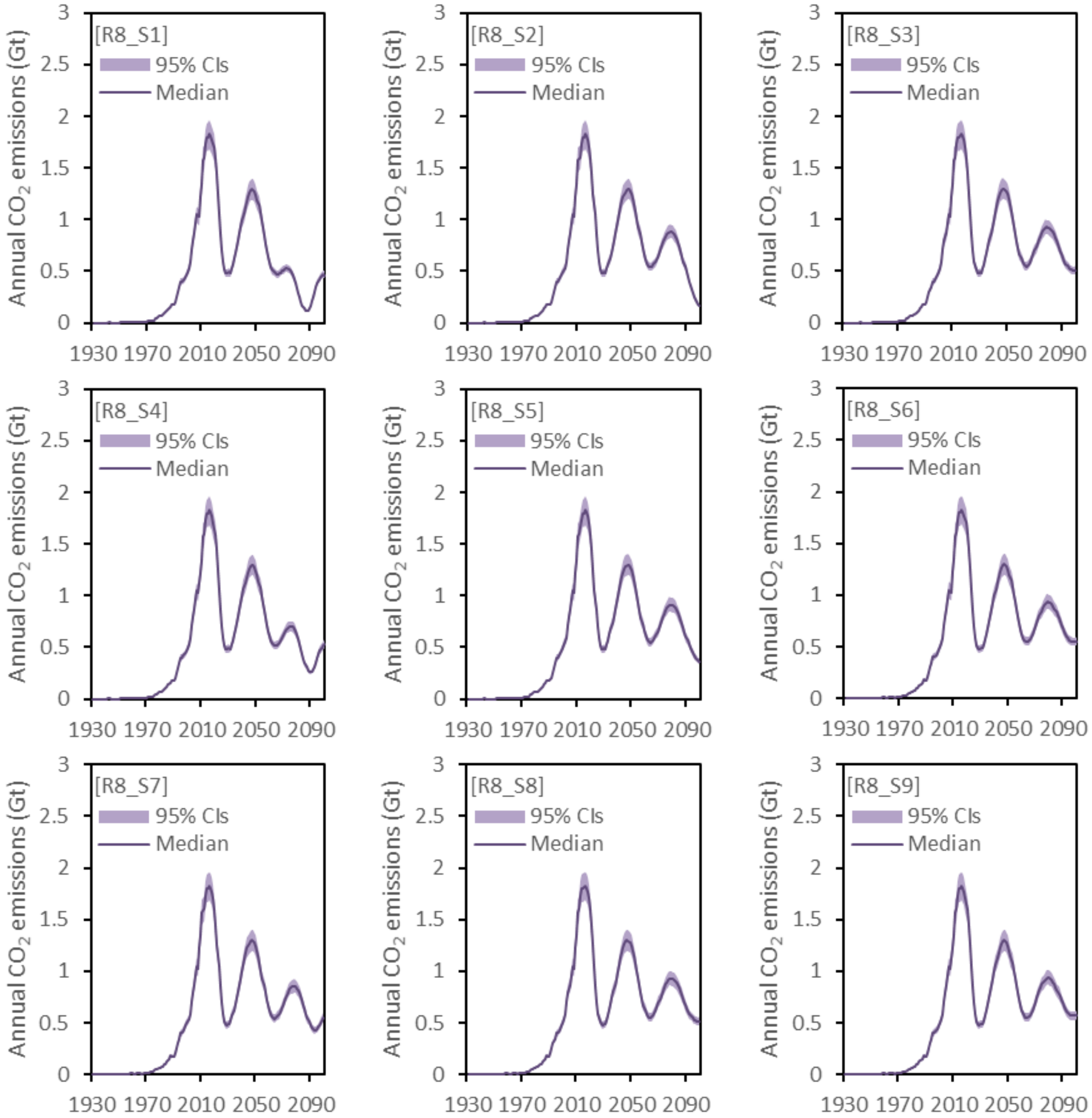

Supplementary Figure 93 | Uncertainties of annual CO_2 emissions in China

Note: The solid lines are the median value of simulated outcomes, and the shaded areas represent the 95% uncertainty range of simulated outcomes. The number of simulation runs is 1,000.

Supplementary Figure 94

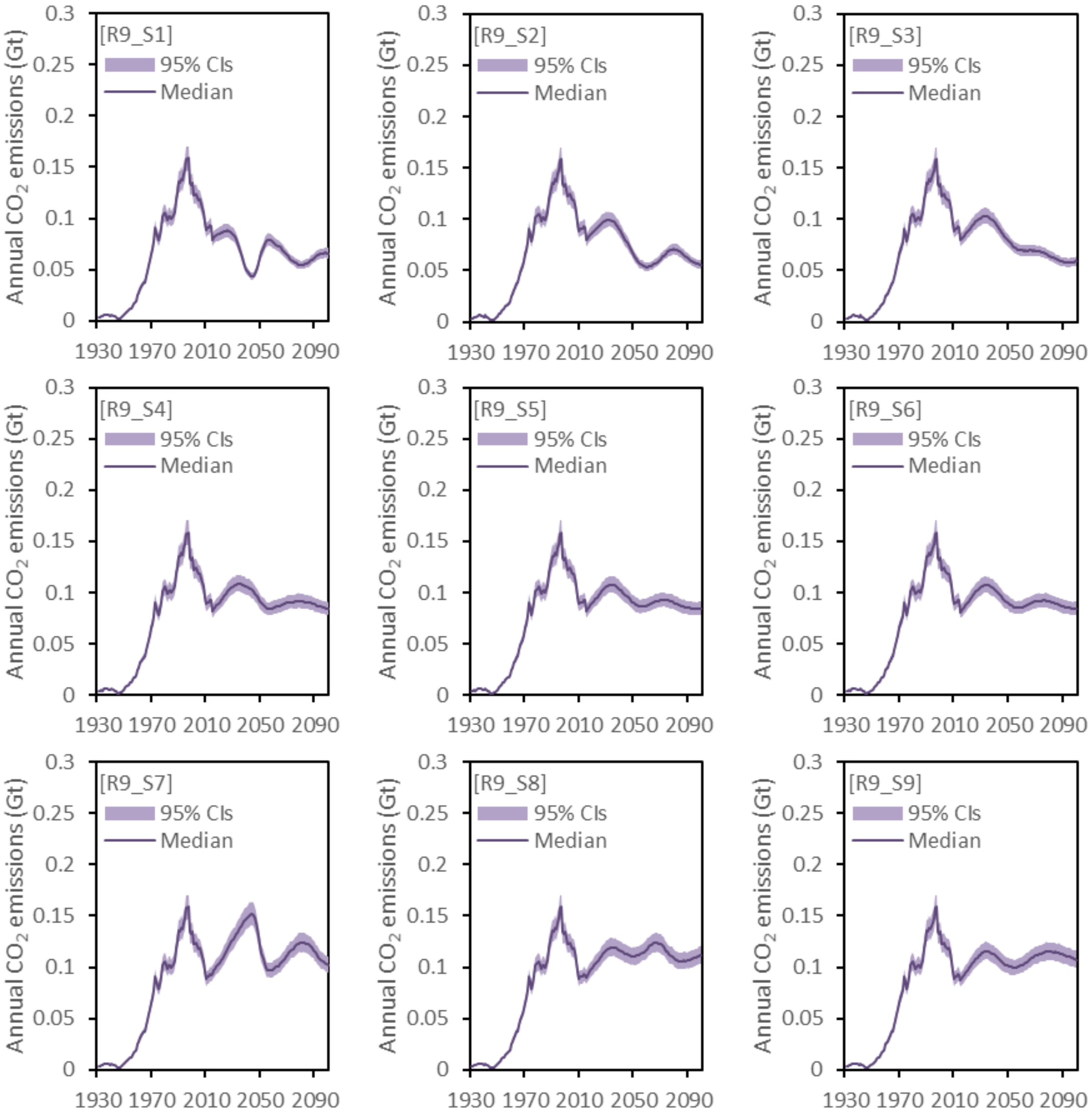

Supplementary Figure 94 | Uncertainties of annual CO_2 emissions in Developed Asia & Oceania

Note: The solid lines are the median value of simulated outcomes, and the shaded areas represent the 95% uncertainty range of simulated outcomes. The number of simulation runs is 1,000.

Supplementary Figure 95

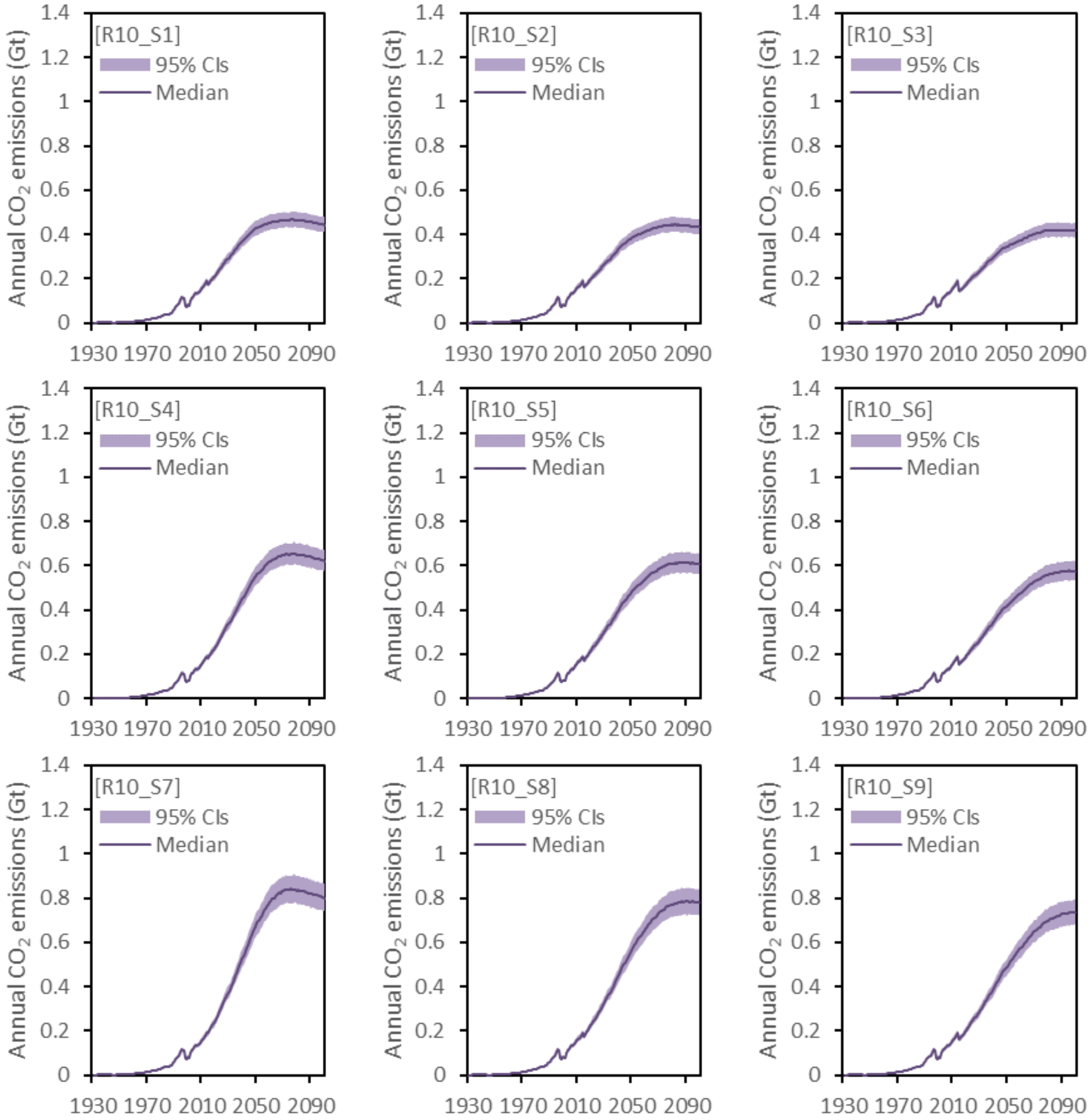

Supplementary Figure 95 | Uncertainties of annual CO_2 emissions in Dev eloping Asia

Note: The solid lines are the median value of simulated outcomes, and the shaded areas represent the 95% uncertainty range of simulated outcomes. The number of simulation runs is 1,000.

Supplementary Figure 96

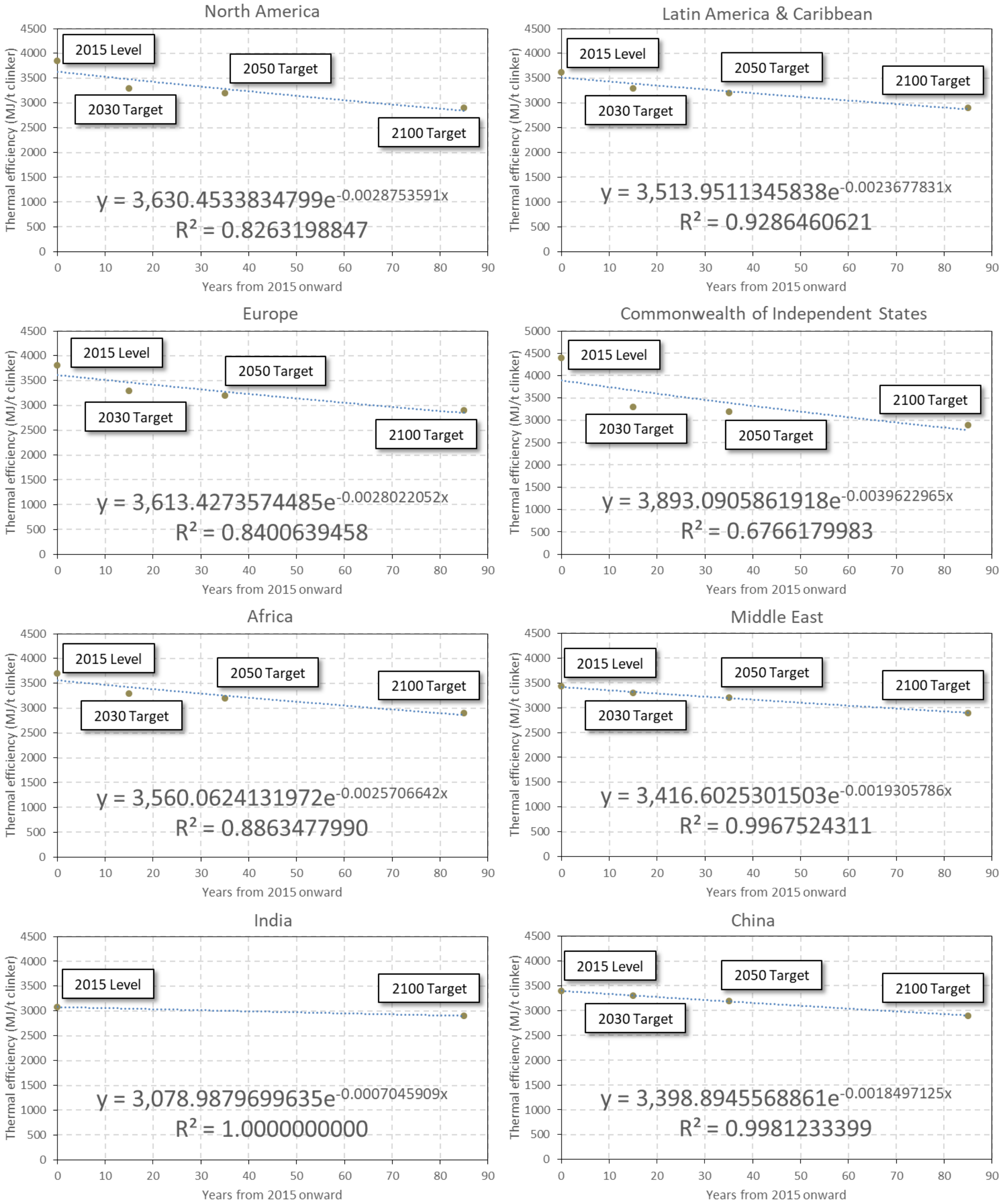

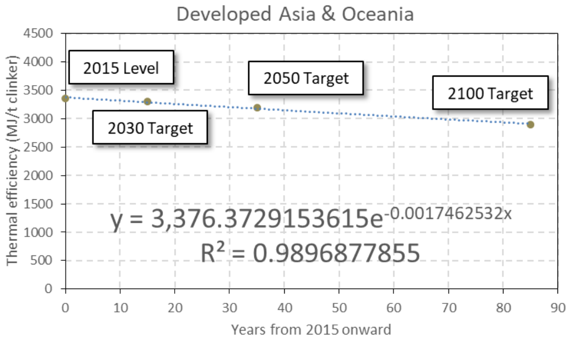

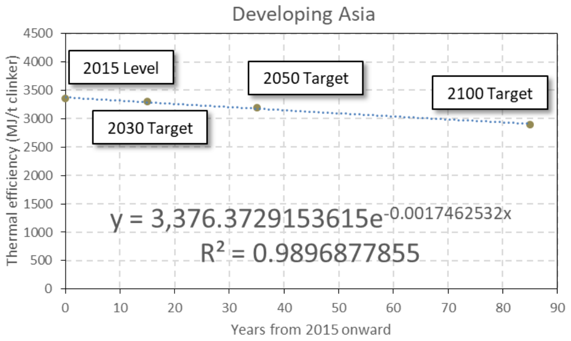

Supplementary Figure 96 | Curve fitting of thermal efficiency improvement by region

Supplementary Figrue 97

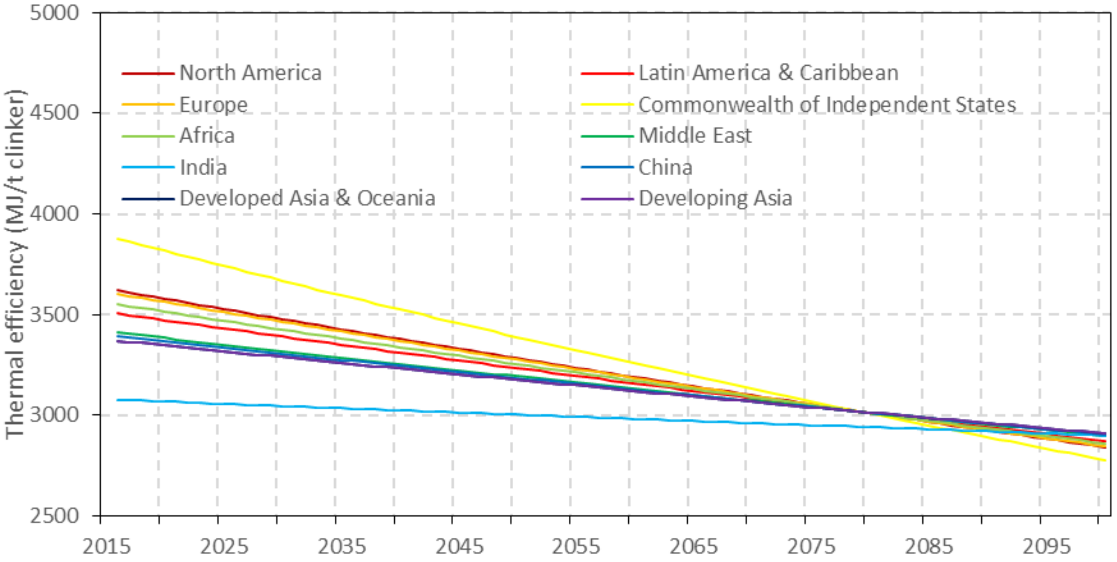

Supplementary Figure 97 | Prediction of thermal efficiency up to 2100 by region

Supplementary Figure 98

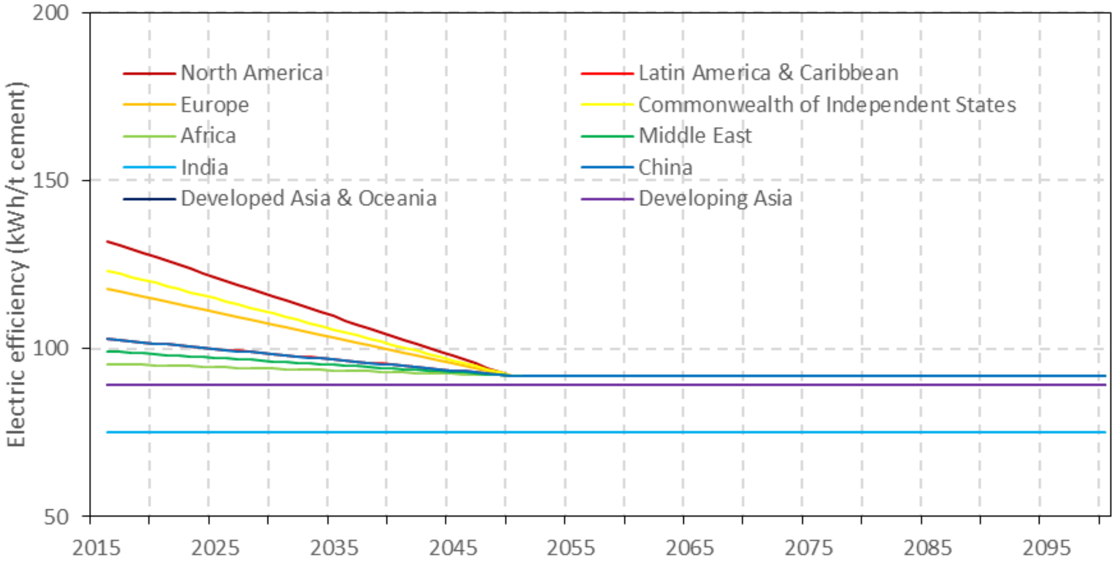

Supplementary Figure 98 | Prediction of electric efficiency up to 2100 by region

Supplementary Figure 99

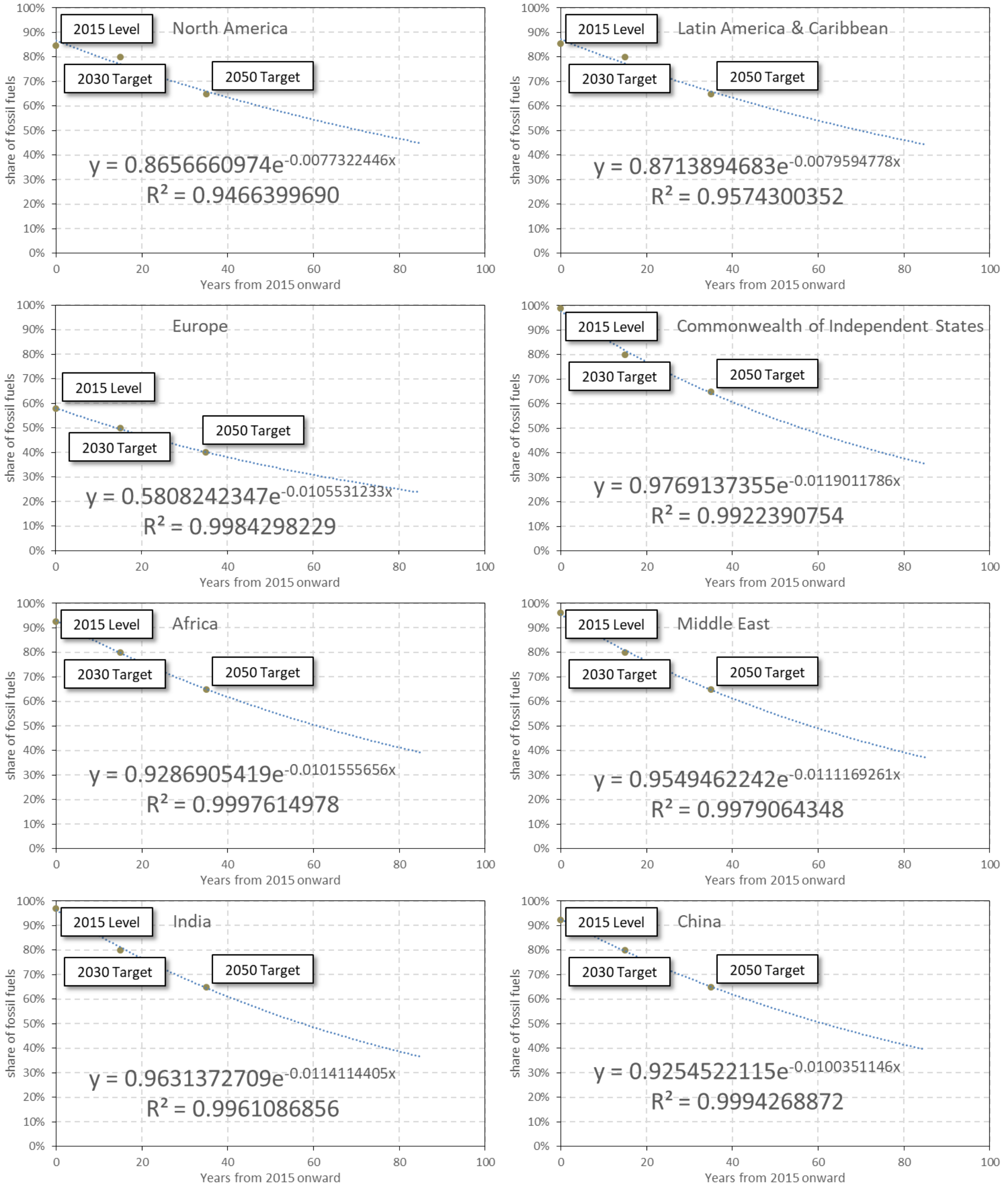

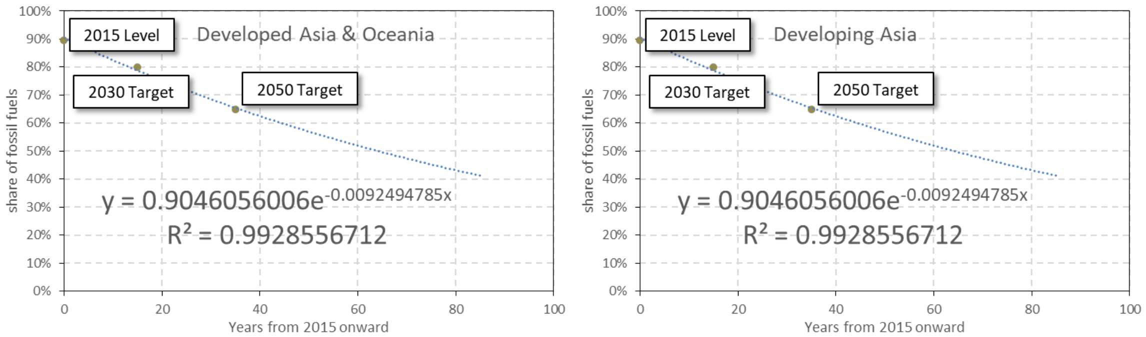

Supplementary Figure 99 | Prediction of share of fossil fuels up to 2100 by region

Supplementary Figure 100

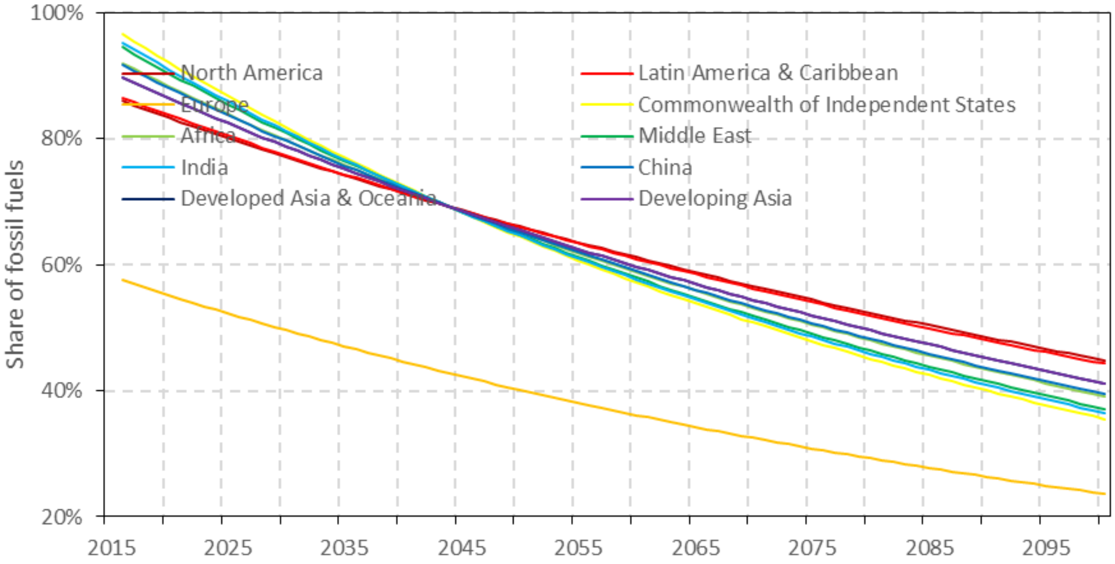

Supplementary Figure 100 | Prediction of share of fossil fuels up to 2100 by region

Supplementary Figure 101

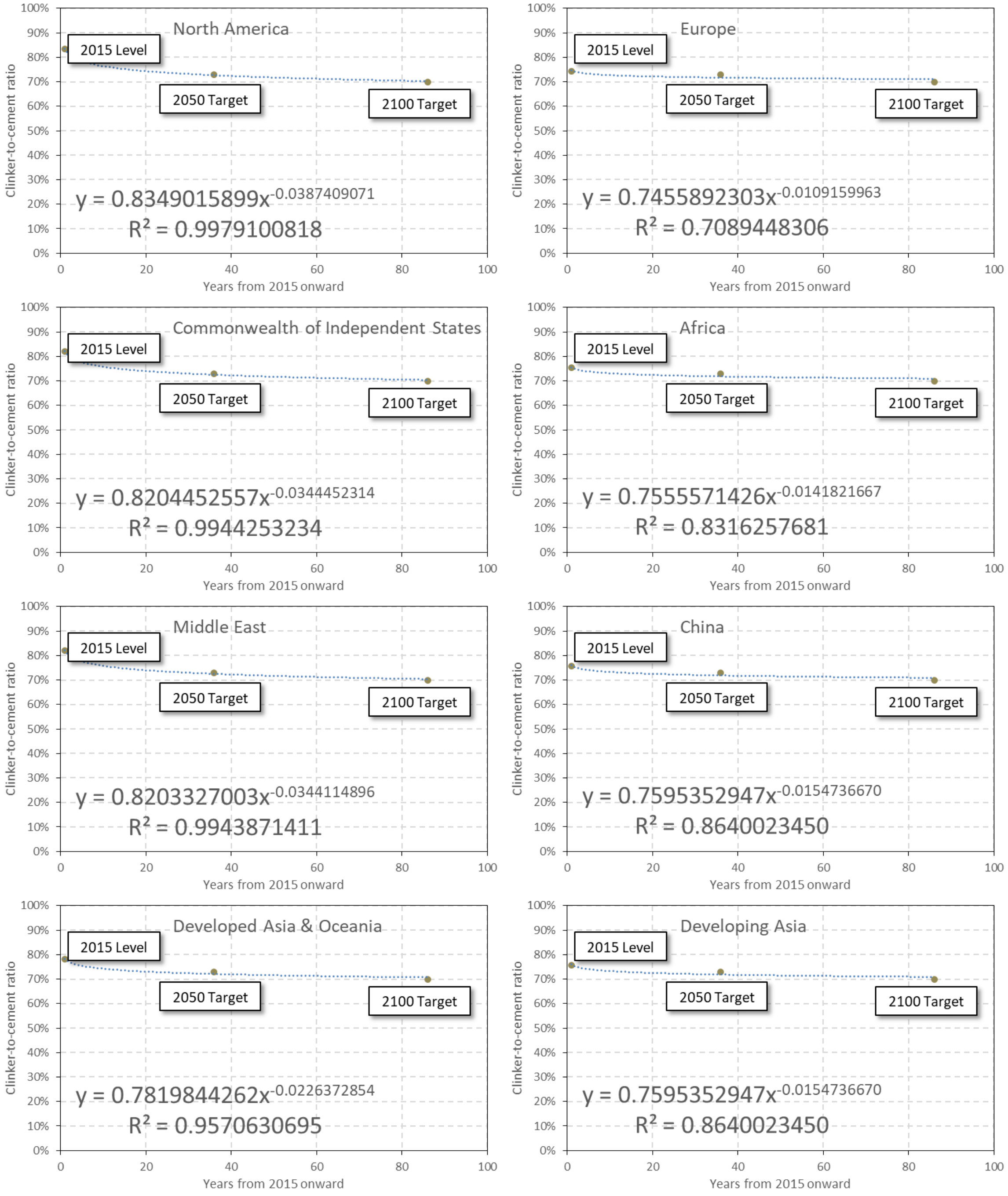

Supplementary Figure 101 | Prediction of clinker-to-cement ratio up to 2100 by region

Supplementary Figure 102

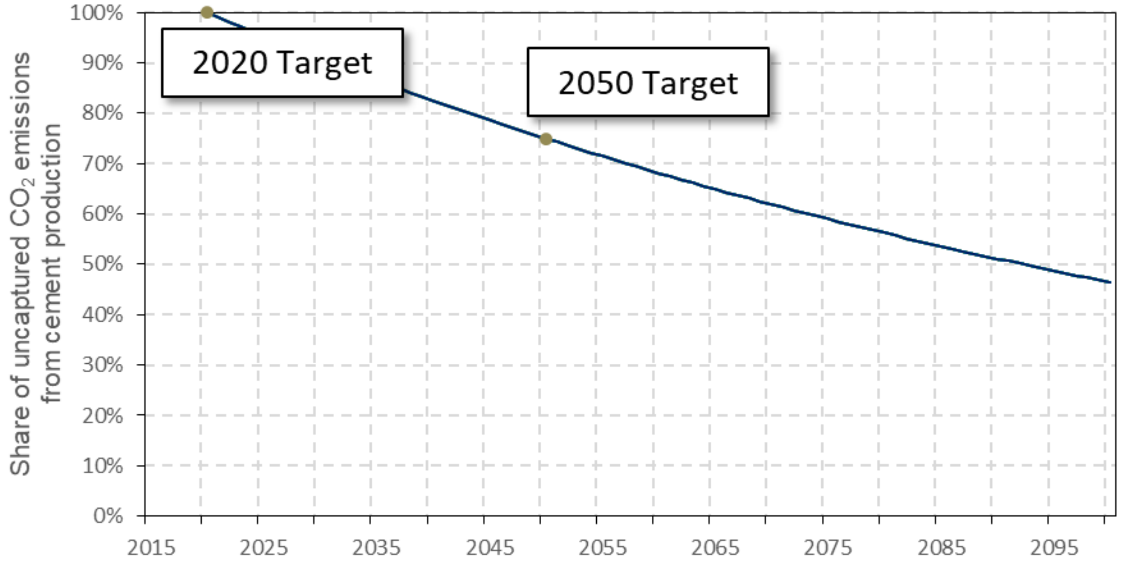

Supplementary Figure 102 | Prediction of the share of uncaptured CO_2 emissions from cement production up to 2100

Supplementary Figure 103

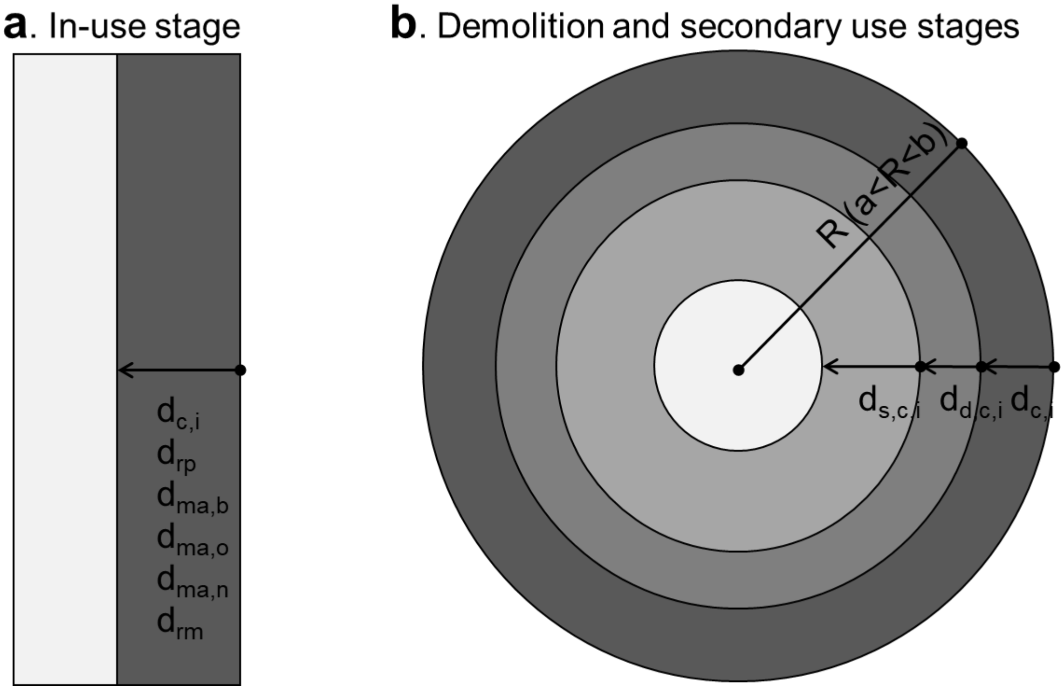

Supplementary Figure 103 | Sketch of carbonation depth during the in-use stage (a) and the demolition and secondary use stages (b).

Note: The definition of symbols is detailed in SUPPLEMENTARY TABLE 6.

Supplementary Figure 104

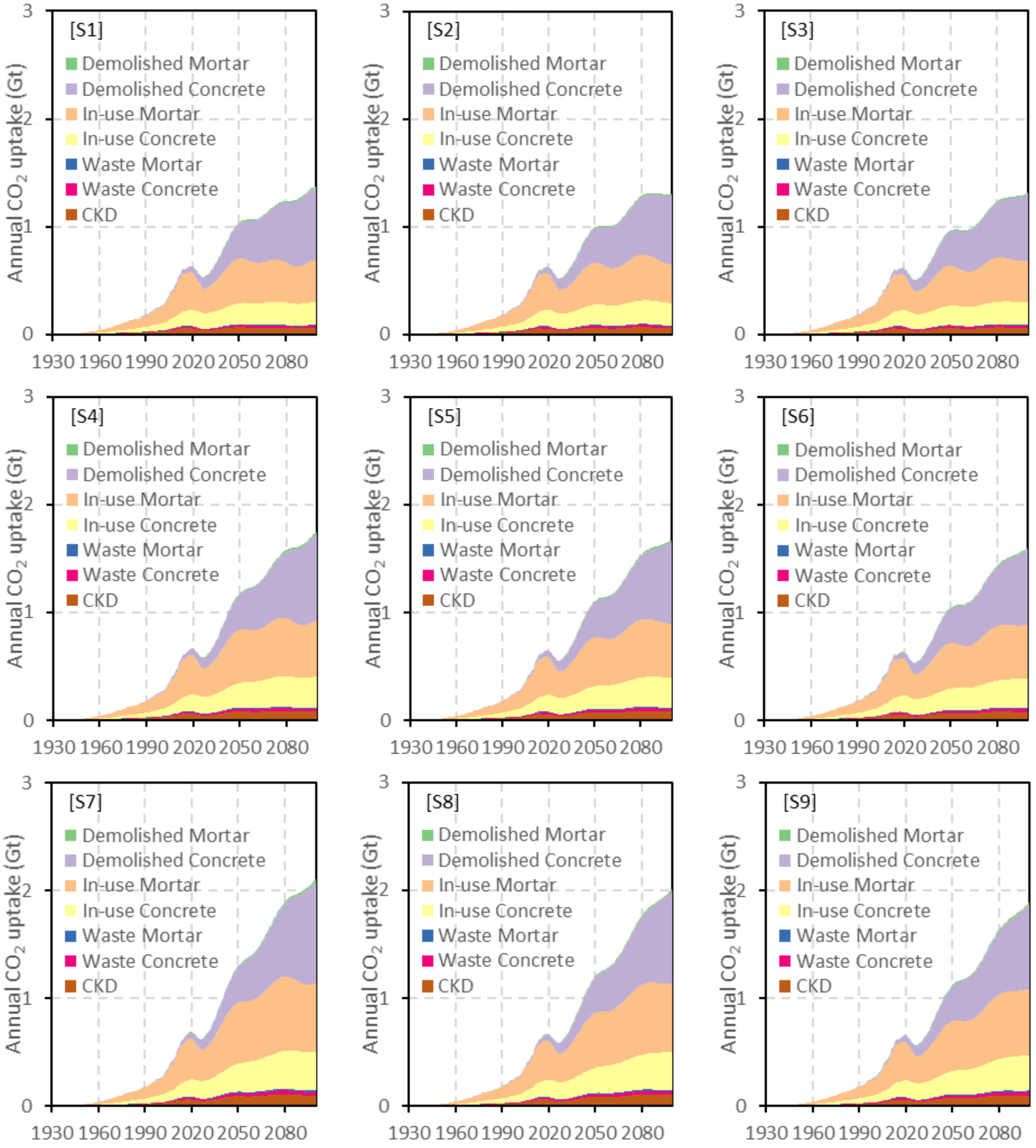

Supplementary Figure 104 | Breakdown of global annual CO_2 uptake without implementing clinker substitution (1930-2100) by sources

Supplementary Figure 105

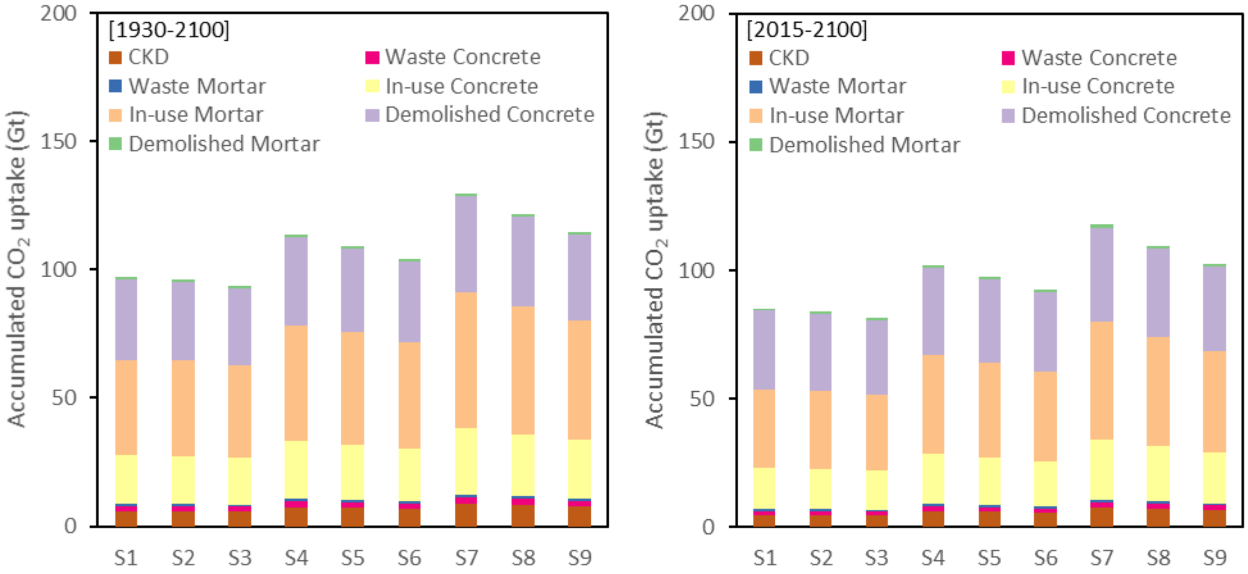

Supplementary Figure 105 | Breakdown of global accumulated CO_2 uptake without implementing clinker substitution from 1930 to 2100 (left) and from 2015 to 2100 (right) by sources

Supplementary Tables

Supplementary Table 1

Supplementary Table 1 | Percentages of secondary uses of demolition wastes

Region/country	Secondary use	Distribution	Mode	Max	Min
China	New concrete	Triangular	0.01	1	0
	Road base	Triangular	2.30	5	2
	Landfill and stacking	Triangular	97.69	80	98
	Bituminous concrete	Triangular	0	1	0
Europe	New concrete	Triangular	0.72	1.8	0.3
	Road base	Triangular	60.42	80	40
	Landfill and stacking	Triangular	38.86	70	20
	Bituminous concrete	Triangular	0	1	0
US	New concrete	Triangular	3.60	5	2.5
	Road base	Triangular	51.00	60	40
	Landfill and stacking	Triangular	40.00	50	30
	Bituminous concrete	Triangular	5.40	6.5	4
Rest of the world	New concrete	Triangular	1.00	2	0
	Road base	Triangular	24.0	34	14
	Landfill and stacking	Triangular	75.00	85	60
	Bituminous concrete	Triangular	0	1	0

Supplementary Table 2

Supplementary Table 2 | Parameters for saturation levels and saturation times (exceptions are marked in bold)

		Residential		Non-Residential		Civil Engineering	
	Scenario	Saturation level (t/capita)	Time reaches 98% of saturation level	Saturation level (t/capita)	Time reaches 98% of saturation level	Saturation level (t/capita)	Time reaches 98% of saturation level
North America	2014 level	5.90		3.80		6.20	
	Low-Fast	5.00	2050	5.00	2050	5.00	2050
	Low-Moderate	5.00	2075	5.00	2075	5.00	2075
	Low-Slow	5.00	2100	5.00	2100	5.00	2100
	Medium-Fast	7.00	2050	7.00	2050	7.00	2050
	Medium-Moderate	7.00	2075	7.00	2075	7.00	2075
	Medium-Slow	7.00	2100	7.00	2100	7.00	2100
	High-Fast	9.00	2050	9.00	2050	9.00	2050
	High-Moderate	9.00	2075	9.00	2075	9.00	2075
	High-Slow	9.00	2100	9.00	2100	9.00	2100
Latin America & Caribbean	2014 level	1.96		2.50		3.05	
	Low-Fast	5.00	2100	5.00	2100	5.00	2100
	Low-Moderate	5.00	2125	5.00	2125	5.00	2125
	Low-Slow	5.00	2150	5.00	2150	5.00	2150
	Medium-Fast	7.00	2100	7.00	2100	7.00	2100
	Medium-Moderate	7.00	2125	7.00	2125	7.00	2125
	Medium-Slow	7.00	2150	7.00	2150	7.00	2150

	High-Fast	9.00	2100	9.00	2100	9.00	2100
	High-Moderate	9.00	2125	9.00	2125	9.00	2125
	High-Slow	9.00	2150	9.00	2150	9.00	2150
Europe	2014 level	7.57		7.18		8.94	
	Low-Fast	5.00	2100	5.00	2100	5.00	2100
	Low-Moderate	5.00	2125	5.00	2125	5.00	2125
	Low-Slow	5.00	2150	5.00	2150	5.00	2150
	Medium-Fast	7.00	2100	7.00	2100	7.00	2100
	Medium-Moderate	7.00	2125	7.00	2125	7.00	2125
	Medium-Slow	7.00	2150	7.00	2150	7.00	2150
	High-Fast	9.00	2100	9.00	2100	9.00	2100
	High-Moderate	9.00	2125	9.00	2125	9.00	2125
	High-Slow	9.00	2150	9.00	2150	9.00	2150
Commonwealth of Independent States	2014 level	2.50		5.93		8.84	
	Low-Fast	5.00	2050	5.00	2050	5.00	2050
	Low-Moderate	5.00	2075	5.00	2075	5.00	2075
	Low-Slow	5.00	2100	5.00	2100	5.00	2100
	Medium-Fast	7.00	2050	7.00	2050	7.00	2050
	Medium-Moderate	7.00	2075	7.00	2075	7.00	2075
	Medium-Slow	7.00	2100	7.00	2100	7.00	2100
	High-Fast	9.00	2050	9.00	2050	9.00	2050

	High-Moderate	9.00	2075	9.00	2075	9.00	2075
	High-Slow	9.00	2100	9.00	2100	9.00	2100
Africa	2014 level	0.83		1.06		1.12	
	Low-Fast	5.00	2100	5.00	2100	5.00	2100
	Low-Moderate	5.00	2125	5.00	2125	5.00	2125
	Low-Slow	5.00	2150	5.00	2150	5.00	2150
	Medium-Fast	7.00	2100	7.00	2100	7.00	2100
	Medium-Moderate	7.00	2125	7.00	2125	7.00	2125
	Medium-Slow	7.00	2150	7.00	2150	7.00	2150
	High-Fast	9.00	2100	9.00	2100	9.00	2100
	High-Moderate	9.00	2125	9.00	2125	9.00	2125
	High-Slow	9.00	2150	9.00	2150	9.00	2150
Middle East	2014 level	3.43		4.17		5.15	
	Low-Fast	5.00	2050	5.00	2050	5.00	2050
	Low-Moderate	5.00	2075	5.00	2075	5.00	2075
	Low-Slow	5.00	2100	5.00	2100	5.00	2100
	Medium-Fast	7.00	2050	7.00	2050	7.00	2050
	Medium-Moderate	7.00	2075	7.00	2075	7.00	2075
	Medium-Slow	7.00	2100	7.00	2100	7.00	2100
	High-Fast	9.00	2050	9.00	2050	9.00	2050
	High-Moderate	9.00	2075	9.00	2075	9.00	2075

	High-Slow	9.00	2100	9.00	2100	9.00	2100
India	2014 level	0.71		0.90		1.10	
	Low-Fast	5.00	2100	5.00	2100	5.00	2100
	Low-Moderate	5.00	2125	5.00	2125	5.00	2125
	Low-Slow	5.00	2150	5.00	2150	5.00	2150
	Medium-Fast	7.00	2100	7.00	2100	7.00	2100
	Medium-Moderate	7.00	2125	7.00	2125	7.00	2125
	Medium-Slow	7.00	2150	7.00	2150	7.00	2150
	High-Fast	9.00	2100	9.00	2100	9.00	2100
	High-Moderate	9.00	2125	9.00	2125	9.00	2125
	High-Slow	9.00	2150	9.00	2150	9.00	2150
China	2014 level	8.19		6.82		3.85	
	Low-Fast	5.00	2100	5.00	2100	5.00	2100
	Low-Moderate	5.00	2125	5.00	2125	5.00	2125
	Low-Slow	5.00	2150	5.00	2150	5.00	2150
	Medium-Fast	7.00	2100	7.00	2100	7.00	2100
	Medium-Moderate	7.00	2125	7.00	2125	7.00	2125
	Medium-Slow	7.00	2150	7.00	2150	7.00	2150
	High-Fast	9.00	2100	9.00	2100	9.00	2100
	High-Moderate	9.00	2125	9.00	2125	9.00	2125
	High-Slow	9.00	2150	9.00	2150	9.00	2150

Developed Asia & Oceania	2014 level	5.69		7.09		8.00	
	Low-Fast	5.00	2050	5.00	2050	5.00	2050
	Low-Moderate	5.00	2075	5.00	2075	5.00	2075
	Low-Slow	5.00	2100	5.00	2100	5.00	2100
	Medium-Fast	7.00	2050	7.00	2050	7.00	2050
	Medium-Moderate	7.00	2075	7.00	2075	7.00	2075
	Medium-Slow	7.00	2100	7.00	2100	7.00	2100
	High-Fast	9.00	2050	9.00	2050	9.00	2050
	High-Moderate	9.00	2075	9.00	2075	9.00	2075
	High-Slow	9.00	2100	9.00	2100	9.00	2100
Developing Asia	2014 level	0.98		1.25		1.53	
	Low-Fast	5.00	2100	5.00	2100	5.00	2100
	Low-Moderate	5.00	2125	5.00	2125	5.00	2125
	Low-Slow	5.00	2150	5.00	2150	5.00	2150
	Medium-Fast	7.00	2100	7.00	2100	7.00	2100
	Medium-Moderate	7.00	2125	7.00	2125	7.00	2125
	Medium-Slow	7.00	2150	7.00	2150	7.00	2150
	High-Fast	9.00	2100	9.00	2100	9.00	2100
	High-Moderate	9.00	2125	9.00	2125	9.00	2125
	High-Slow	9.00	2150	9.00	2150	9.00	2150

Supplementary Table 3

Supplemententray Table 3 | Region definition of 10 regions in the world.

Region name	Region index	Abbr.
North America	1	NA
Latin America & Caribbean	2	LAC
Europe	3	EU
Commonwealth of Independent States	4	CIS
Africa	5	AF
Middle East	6	ME
India	7	IN
China	8	CN
Developed Asia & Oceania	9	DAO
Developing Asia	10	DA

Supplementary Table 4

Supplementary Table 4 | Region Aggregation of 184 countries.

Name	Country code	Region index	Name	Country code	Region index
Afghanistan	4	10	Lebanon	422	6
Albania	8	3	Lesotho	426	5
Algeria	12	5	Liberia	430	5
Andorra	20	3	Libya	434	5
Angola	24	5	China, Macao SAR	446	9
Anguilla	660	2	Madagascar	450	5
Antigua and Barbuda	28	2	Malaysia	458	10
Argentina	32	2	Maldives	462	10
Aruba	533	2	Mali	466	5
Australia	36	9	Malta	470	3
Austria	40	3	Martinique	474	2
Bahamas	44	2	Mauritania	478	5
Bahrain	48	6	Mauritius	480	5
Barbados	52	2	Mayotte	175	5
Belize	84	2	Mexico	484	2
Benin	204	5	Mongolia	496	10
Bermuda	60	1	Montserrat	500	2
Bhutan	64	10	Morocco	504	5
Bolivia	68	2	Mozambique	508	5
Botswana	72	5	Namibia	516	5
Brazil	76	2	Nepal	524	10
Brunei	96	10	Neth. Antilles and Aruba	532	2
Bulgaria	100	3	Netherlands	528	3
Burkina Faso	854	5	New Caledonia	540	9
Burma	104	10	New Zealand	554	9
Burundi	108	5	Nicaragua	558	2
Cambodia	116	10	Niger	562	5
Cameroon	120	5	Nigeria	566	5
Canada	124	1	Norway	579	3
Cape Verde Islands	132	5	Oman	512	6

Central African Rep.	140	5	Palau	585	9
Chad	148	5	Panama	591	2
Chile	152	2	Papua New Guinea	598	9
China	156	8	Paraguay	600	2
Colombia	170	2	Peru	604	2
Comoros	174	5	Philippines	608	10
Congo (Brazzaville)	178	5	Poland	616	3
Congo (Kinshasa)	180	5	Portugal	620	3
Cook Isds	184	9	Qatar	634	6
Costa Rica	188	2	Réunion	638	5
Côte d'Ivoire	384	5	Romania	642	3
Cuba	192	2	Rwanda	646	5
Cyprus	196	3	Saint Kitts and Nevis	659	2
Denmark	208	3	Saint Lucia	662	2
Djibouti	262	5	Saint Pierre and Miquelon	666	1
Dominica	212	2	Saint Vincent and the Grenadines	670	2
Dominican Republic	214	2	Samoa	882	9
Ecuador	218	2	Sao Tome and Principe	678	5
Egypt	818	5	Saudi Arabia	682	6
El Salvador	222	2	Senegal	686	5
Eritrea	232	5	Seychelles	690	5
Ethiopia	231	5	Sierra Leone	694	5
Faeroe Isds	234	3	Singapore	702	9
Fiji	242	9	Solomon Isds	90	9
Finland	246	3	Somalia	706	5
France	251	3	South Africa, sales	710	5
French Guiana	254	2	Spain, including Canary Islands	724	3
French Polynesia	258	9	Sri Lanka	144	10
FS Micronesia	583	9	State of Palestine	275	6
Gabon	266	5	Sudan	729	5

Gambia	270	5	Suriname	740	2
Germany	276	3	Swaziland	748	5
Ghana	288	5	Sweden	752	3
Greece	300	3	Switzerland	757	3
Greenland	304	1	Syria	760	6
Grenada	308	2	Taiwan	158	9
Guadeloupe	312	2	Tanzania	834	5
Guatemala	320	2	Thailand	764	10
Guinea	324	5	Togo	768	5
Guinea-Bissau	624	5	Tonga	776	9
Guyana	328	2	Trinidad and Tobago	780	2
Haiti	332	2	Tunisia	788	5
Honduras	340	2	Turkey	792	6
Hong Kong	344	9	Turks and Caicos Isds	796	2
Hungary	348	3	Tuvalu	798	9
Iceland	352	3	Uganda	800	5
India	699	7	United Arab Emiratese	784	6
Indonesia	360	10	United Kingdom	826	3
Iran	364	6	US Virgin Isds	850	2
Iraq	368	6	United States, including Puerto Rico	842	1
Ireland	372	3	Uruguay	858	2
Israel	376	6	Vanuatu	548	9
Italy	381	3	Venezuela	862	2
Jamaica	388	2	Vietnam	704	10
Japan	392	9	Wallis and Futuna Isds	876	9
Jordan	400	6	Yemen	887	6
Kenya	404	5	U.S.S.R.	810	4
Kiribati	296	9	Yugoslavia	890	3
Korea, North	408	10	Czechoslovakia	200	3
Korea, Republic of	410	9	Rhodesia and Nyasaland, Federation	717	5
Kuwait	414	6	East and West Pakistan	588	10
Laos	418	10	Belgium-Luxembourg	58	3

Note: In order to reconcile the country-specific trade data and production data, several countries were aggregated and tracked back to their former names as follows: i) former Soviet Union: includes Armenia, Azerbaijan, Belarus, Estonia, Georgia, Kazakhstan, Kyrgyzstan, Latvia, Lithuania, Moldova, Russia, Tajikistan, Turkmenistan, Ukraine and Uzbekistan; ii) former Yugoslavia: includes Bosnia and Herzegovina, Croatia, Macedonia, Serbia, Montenegro and Slovenia; iii) former Czechoslovakia: includes Czech Republic and Slovakia; iv) former Rhodesia and Nyasaland Federation: Malawi, Zambia and

Zimbabwe; v) former East and West Pakistan: Bangladesh and Pakistan; and vi) former Belgium-Luxembourg: Belgium and Luxembourg.

Supplementary Table 5

Supplementary Table 5 | Technology mitigation options and their status quo in the global cement industry

Region	E-M1 (Thermal efficiency, MJ/t clinker)		E-M2 (Electric efficiency, KWh/t cement)		E-M3 (Share of fossil fuels)		E-M4 (Clinker-to-cement ratio)		E-M5 (Penetration rate of CCS)	
	1990	2015	1990	2015	1990	2015	1990	2015	1990	2015
NA	4863.0	3847.7	146.2	131.9	96.1%	84.8%	91.8%	83.4%	-	-
LAC	4108.6	3616.6	116.2	104.1	98.0%	85.5%	81.7%	69.9%	-	-
EU	4116.5	3813.5	115.2	115.8	97.6%	57.8%	78.6%	74.3%	-	-
CIS	6421.3	4393.5	127.3	123.2	100%	98.9%	82.2%	81.9%	-	-
AF	4629.6	3707.2	117.7	95.5	100%	92.7%	89.0%	75.3%	-	-
ME	4163.4	3428.7	141.7	99.2	100%	96.1%	84.9%	81.9%	-	-
IN	3921.5	3079.0	99.7	81.5	99.8%	97.1%	86.0%	68.9%	-	-
CH	3470.3	3395.0	103.0	91.1	99.0%	92.3%	87.0%	75.7%	-	-
DAO	3821.3	3352.4	124.1	89.3	100%	89.6%	92.2%	78.0%	-	-
DA	3821.3	3352.4	124.1	89.3	100%	89.6%	87.0%	75.7%	-	-
World Average	4267.5	3544.2	118.5	100.5	98.1%	84.3%	83.3%	74.9%	-	-

Note: All indicators are derived from Cement Sustainability Initiative's "Getting the Numbers Right" (GNR) data[16]; time horizon of GNR data is 1900-2015; Thermal efficiency is derived from the total thermal energy consumption and the total production volumes of cement; Electric efficiency and clinker-to-cement ratio are directly taken from original data; Share of fossil fuels is derived from fuel mix.

Supplementary Table 6

Supplementary Table 6 | List of symbols used in cement carbonation model

Symbol	Meaning	Unit
U_{CKD}	uptake of CO_2 by CKD	kg
W_p	mass of cement production	kg
$C_{clinker}$	clinker-to-cement ratio	kg/kg
r_{CKD}	CKD generation rate based on clinker	kg/kg
$r_{landfill}$	proportion of CKD landfilled	kg/kg
$f_{CaO,CKD}$	average CaO content of CKD	kg/kg
γ_2	proportion of CaO within fully carbonated CKD that converts to $CaCO_3$	kg/kg
M_r	mole ratio of CO_2 to CaO	kg/kg
$U_{w,c}$	uptake of CO_2 by construction waste from concrete	kg
W_c	mass of cement used for concrete	kg
l	loss rate of cement in construction stage	kg/kg
F_c	carbonated mass fraction of construction waste from concrete	kg/kg
f_{CaO}	average mass content of CaO in clinker	kg/kg
γ	proportion of CaO within fully carbonated cement that converts to $CaCO_3$ for concrete cement	kg/kg
$U_{w,m}$	uptake of CO_2 by construction waste from mortar	kg
W_m	mass of cement used for mortar	kg
F_m	carbonated fraction of construction waste from mortar	kg/kg
γ_1	proportion of CaO within fully carbonated cement that converts to $CaCO_3$ for mortar cement	kg/kg
i	concrete strength classes	-
$k_{c,i}$	carbonation rate of concrete during the use stage	$mm/\sqrt{yr}$
$\beta_{i,ec}$	effect of exposure conditions on carbonation rate of concrete	-
β_{ad}	effect of cement additives on carbonation rate of concrete	-
β_{CO_2}	effect of CO_2 concentration on carbonation rate of concrete	-
β_{cc}	effect of coating and cover on carbonation rate of concrete	-
$d_{c,i}$	carbonated depth of concrete in the use stage	mm
t_{use}	a certain period of time during the use stage	yr
$V_{c,i}$	volume of carbonated concrete during the use stage	m^3
$W_{c,i}$	mass of cement used in different concrete classes	kg
$C_{c,i}$	concrete cement content	kg/m^3
T_w	average thickness of concrete structures	mm
$W_{c,use}$	mass of carbonated cement used in concrete over a certain period of time during the use stage	kg
$U_{c,use}$	uptake of CO_2 by concrete during the use stage	kg
$d_{d,c,i}$	carbonated depths during the demolition stage	mm
$k_{d,c,i}$	carbonation coefficient of concrete in open air exposure conditions	$mm/\sqrt{yr}$
t_d	average exposure time during the demolition stage	yr
$F_{d,i}$	carbonated fraction of demolished concrete during the demolition stage	kg/kg
R	radius of demolition waste particles	mm
a	minimum diameter of demolition waste particles	mm
b	maximum diameter of demolition waste particles	mm
$\mathrm{W}_{c,d}$	mass of carbonated cement used in concrete during the demolition stage	kg
$W_{c,i,d}$	mass of carbonated cement used in different concrete classes during the demolition stage	kg
$W_{c,i,use}$	mass of carbonated cement used in different concrete classes during the use stage	kg
$U_{c,d}$	uptake of CO_2 by concrete during the demolition stage	kg
$d_{s,c,i}$	carbonated depths during the secondary use stage	mm
$k_{s,c,i}$	carbonation coefficient in buried conditions	$mm/\sqrt{yr}$
t_s	a certain period of time during the secondary use stage	yr

$d_{t,c,i}$	total carbonated depths during demolition and secondary use stages	mm
$W_{c,i,s}$	mass of carbonated cement used in different concrete classes during the secondary use stage	kg
$\mathrm{W}_{c,s}$	mass of carbonated cement used in concrete during the secondary use stage	kg
$U_{c,s}$	uptake of CO_2 by concrete during the secondary use stage	kg
d_{rp}	depth of carbonated mortar used for rendering and plastering	mm
k_m	carbonation rate of mortar	$mm/\sqrt{yr}$
F_{rp}	carbonated fraction of cement used for rendering and plastering	kg/kg
T_{rp}	thickness of rendering and plastering	mm
$U_{m,rp}$	uptake of CO_2 by mortar used for rendering and plastering	kg
r_{rp}	percentage of mortar used for rendering and plastering	-
F_{rp}	carbonated fraction of cement used for rendering and plastering	kg/kg
$d_{ma,b}$	depth of carbonated mortar for masonry rendered on both sides	mm
t_r	time that mortar for rendering is fully carbonated	yr
$F_{ma,b}$	carbonated fraction of mortar used for masonry rendered on both sides	kg/kg
$U_{m,ma,b}$	uptake of CO_2 by mortar used for masonry rendered on both sides	kg
r_{ma}	percentage of mortar used for masonry	-
r_b	percentage of masonry walls with rendering on both sides	-
$d_{ma,o}$	depth of carbonated mortar for masonry rendered on only one side	mm
$F_{ma,o}$	carbonated fraction of mortar used for masonry rendered on only one side	kg/kg
$U_{m,ma,o}$	uptake of CO_2 by mortar used for masonry rendered on only one side	kg
r_o	percentage of masonry walls with rendering on only one side	-
$d_{ma,n}$	depth of carbonated mortar for masonry without rendering	mm
$F_{ma,n}$	carbonated fraction of mortar used for masonry without rendering	kg/kg
$U_{m,ma,n}$	uptake of CO_2 by mortar used for masonry without rendering	kg
r_n	percentage of masonry walls without rendering	-
d_{rm}	depth of carbonated mortar used for repairing and maintaining	mm
F_{rm}	carbonated fraction of cement used for repairing and maintenance	kg/kg
T_{rm}	thickness of repairing and maintenance	mm
$U_{m,rm}$	uptake of CO_2 by mortar used for repairing and maintenance	kg
r_{rm}	percentage of mortar used for repairing and maintenance	-

Supplementary Table 7

Supplementary Table 7 | Market shares of concrete cement

Region/country	Distribution	Scale	Shape	Max	Min
China[24]	Weibull	73.4%	13	87.4%	47.2%
Europe[35,36]	Weibull	74.9%	14.8	87.8%	62.3%
US[37]	Weibull	89.1%	25.5	90.8%	70.0%
Rest of the world (refer to Europe)	Weibull	74.9%	14.8	87.8%	62.3%

Supplementary Table 8

Supplementary Table 8 | Market shares of concrete strength classes across regions and countries

Region/country	Strength class	Distribution	Scale	Shape	Max	Min
China[24]	≤C15	Weibull	16.50%	3.5	33.50%	0.00%
	C16-C23	Weibull	13.70%	3	25.80%	0.00%
	C23-C35	Weibull	66.00%	7	82.80%	41.60%
	>C35	Weibull	11.60%	3.5	23.40%	0.00%
Europe[21,35,36]	≤C15	Weibull	5.50%	12	8.00%	2.90%
	C16-C23	Weibull	40.70%	12	54.00%	18.90%
	C23-C35	Weibull	46.80%	16	62.90%	32.00%
	>C35	Weibull	10.90%	12	13.50%	8.00%
US[35,38,39]	≤C15	Weibull	22.20%	12	40.00%	0.00%
	C16-C23	Weibull	40.50%	12	60.00%	5.00%
	C23-C35	Weibull	29.50%	8	80.00%	20.00%
	>C35	Weibull	12.70%	16	15.00%	10.00%
Rest of the world (refer to Europe)	≤C15	Weibull	5.50%	12	8.00%	2.90%
	C16-C23	Weibull	40.70%	12	54.00%	18.90%
	C23-C35	Weibull	46.80%	16	62.90%	32.00%
	>C35	Weibull	10.90%	12	13.50%	8.00%

Supplementary Table 9

Supplementary Table 9 | Cement content of concrete

Strength class	Distribution	Max	Min
≤C15[24]	Uniform	288	165
C16-C23[24]	Uniform	390	240
C23-C35[24]	Uniform	400	280
>C35[24]	Uniform	670	300

Supplementary Table 10

Supplementary Table 10 | Carbonation rates of different strength classes in different exposure conditions

Exposure condition	Region/country	Strength class	Distribution	Max	Min
Indoor, outdoor exposed, and outdoor sheltered	China[24]	≤C15	Uniform	13.9	6.1
		C16-C23	Uniform	9.8	3.9
		C23-C35	Uniform	7	2.4
		>C35	Uniform	4	1.3
	Europe[21]	≤C15	Uniform	15	5
		C16-C23	Uniform	9	2.5
		C23-C35	Uniform	6	1.5
		>C35	Uniform	3.5	1
	US[33]	≤C15	Uniform	7.1	2.15
		C16-C23	Uniform	6.9	3.5
		C23-C35	Uniform	5.4	2.7
		>C35	Uniform	3.8	2.5
	Rest of the world (refer to Europe)	≤C15	Uniform	15	5
		C16-C23	Uniform	9	2.5
		C23-C35	Uniform	6	1.5
		>C35	Uniform	3.5	1
Buried	China[24]	≤C15	Uniform	3.8	1.9
		C16-C23	Uniform	1.9	1.0
		C23-C35	Uniform	1.0	0.7
		>C35	Uniform	0.5	0.3
	Europe[21]	≤C15	Uniform	3	2
		C16-C23	Uniform	1.5	1
		C23-C35	Uniform	1	0.75
		>C35	Uniform	0.75	0.5
	US[33]	≤C15	Uniform	3	2
		C16-C23	Uniform	1.5	1
		C23-C35	Uniform	1	0.75
		>C35	Uniform	0.75	0.5
	Rest of the world (refer to Europe)	≤C15	Uniform	3	2
		C16-C23	Uniform	1.5	1
		C23-C35	Uniform	1	0.75
		>C35	Uniform	0.75	0.5

Supplementary Table 11

Supplementary Table 11 | Percentages of different particle sizes

Region/country	Secondary use	Particle size	Distribution	Max	Min	Relation
China[24]	New concrete	<5 mm	Uniform	20.0%	2.1%	"<5mm"+"5-10mm"+"10-20mm"+"20-40mm" = 100%
		5-10 mm	Uniform	41.2%	17.5%	
		10-20 mm	Uniform	45.0%	32.0%	
		20-40 mm	Uniform	26.7%	10.0%	
	Road base	<1 mm	Uniform	25.9%	5.1%	"<1mm"+"1-10mm"+"10-30mm"+">30mm" = 100%
		1-10 mm	Uniform	36.7%	20.0%	
		10-30 mm	Uniform	60.0%	26.7%	
		30-53 mm	Uniform	35.0%	0.0%	
	Landfill and stacking	<10 mm	Uniform	25.6%	10.9%	"<10mm"+"10-30mm"+"30-50mm"+">50mm" = 100%
		10-30 mm	Uniform	35.4%	19.5%	
		30-50 mm	Uniform	26.8%	10.6%	
		>50 mm	Uniform	48.4%	24.8%	
	Bituminous concrete	<5 mm	Uniform	20.0%	2.1%	"<5mm"+"5-10mm"+"10-20mm"+"20-40mm" = 100%
		5-10 mm	Uniform	41.2%	17.5%	
		10-20 mm	Uniform	45.0%	32.0%	
		20-40 mm	Uniform	26.7%	10.0%	
Europe[24]	New concrete	<5 mm	Uniform	36.0%	22.5%	"<5mm"+"5-10mm"+"10-20mm"+"20-40mm" = 100%
		5-10 mm	Uniform	15.0%	12.5%	
		10-20 mm	Uniform	44.0%	20.0%	
		20-40 mm	Uniform	45.0%	5.0%	
	Road base	<1 mm	Uniform	21.0%	10.0%	"<1mm"+"1-10mm"+"10-30mm"+">30mm" = 100%
		1-10 mm	Uniform	30.0%	25.0%	
		10-30 mm	Uniform	44.0%	20.0%	
		30-53 mm	Uniform	45.0%	5.0%	
	Landfill and stacking	<10 mm	Uniform	25.6%	12.2%	"<10mm"+"10-30mm"+"30-50mm"+">50mm" = 100%
		10-30 mm	Uniform	35.4%	19.5%	
		30-50 mm	Uniform	22.5%	10.6%	
		>50 mm	Uniform	48.4%	24.8%	
	Bituminous concrete	<5 mm	Uniform	36.0%	22.5%	"<5mm"+"5-10mm"+"10-20mm"+"20-40mm" = 100%
		5-10 mm	Uniform	15.0%	12.5%	
		10-20 mm	Uniform	44.0%	20.0%	
		20-40 mm	Uniform	45.0%	5.0%	
US[24]	New concrete	<5 mm	Uniform	36.0%	22.5%	"<5mm"+"5-10mm"+"10-20mm"+"20-40mm" = 100%
		5-10 mm	Uniform	15.0%	12.5%	
		10-20 mm	Uniform	44.0%	20.0%	
		20-40 mm	Uniform	45.0%	5.0%	
	Road base	<1 mm	Uniform	21.0%	10.0%	"<1mm"+"1-10mm"+"10-30mm"+">30mm" = 100%
		1-10 mm	Uniform	30.0%	25.0%	
		10-30 mm	Uniform	44.0%	20.0%	
		30-53 mm	Uniform	45.0%	5.0%	
	Landfill and stacking	<10 mm	Uniform	25.6%	10.9%	"<10mm"+"10-30mm"+"30-50mm"+">50mm" = 100%
		10-30 mm	Uniform	35.4%	19.5%	
		30-50 mm	Uniform	26.8%	10.6%	
		>50 mm	Uniform	48.4%	24.8%	
	Bituminous concrete	<5 mm	Uniform	36.0%	22.5%	"<5mm"+"5-10mm"+"10-20mm"+"20-40mm" = 100%
		5-10 mm	Uniform	15.0%	12.5%	
		10-20 mm	Uniform	44.0%	20.0%	
		20-40 mm	Uniform	45.0%	5.0%	
Rest of the world[24]	New concrete	<5 mm	Uniform	37.0%	15.0%	"<5mm"+"5-10mm"+"10-20mm"+"20-40mm" = 100%
		5-10 mm	Uniform	23.0%	12.0%	
		10-20 mm	Uniform	46.0%	24.0%	
		20-40 mm	Uniform	39.0%	16.0%	

	Road base	<1 mm	Uniform	24.7%	10.0%	"<1mm"+"1-10mm"+"10-30mm"+">30mm" = 100%
		1-10 mm	Uniform	28.0%	20.3%	
		10-30 mm	Uniform	51.3%	35.3%	
		30-53 mm	Uniform	26.0%	10.7%	
	Landfill and stacking	<10 mm	Uniform	25.6%	12.2%	"<10mm"+"10-30mm"+"30-50mm"+">50mm" = 100%
		10-30 mm	Uniform	35.4%	19.5%	
		30-50 mm	Uniform	22.5%	10.6%	
		>50 mm	Uniform	48.4%	24.8%	
	Bituminous concrete	<5 mm	Uniform	37.0%	15.0%	"<5mm"+"5-10mm"+"10-20mm"+"20-40mm" = 100%
		5-10 mm	Uniform	23.0%	12.0%	
		10-20 mm	Uniform	46.0%	24.0%	
		20-40 mm	Uniform	39.0%	16.0%	

Supplementary Table 12

Supplementary Table 12 | Exposure time during demolition stage

Region/country	Distribution	Scale	Shape	Max	Min
China[24]	Weibull	0.5	4	0.8	0.1
Europe[21,23,43]	Weibull	0.5	4	0.7	0.1
US (refer to Europe)	Weibull	0.5	4	0.7	0.1
Rest of the world (refer to China)	Weibull	0.5	4	1	0.1

Supplementary Table 13

Supplementary Table 13 | Market shares of mortar cement

Region/country	Distribution	Scale	Shape	Max	Min
China[24]	Weibull	30.8%	12	91.7%	10.0%
Europe[35,36]	Weibull	29.0%	12	37.1%	12.0%
US[37]	Weibull	13.2%	12.5	29.6%	9.1%
Rest of the world (refer to Europe)	Weibull	29.0%	12	37.1%	12.0%

Supplementary Table 14

Supplementary Table 14 | Percentages of mortar uses

Region/country	Mortar use	Distribution	Scale	Shape	Max	Min
China[24]	Rendering and plastering	Weibull	52.4%	14	72.5%	24.0%
	Masonry	Weibull	18.8%	12	52.2%	1.7%
	Repairing and maintenance	Weibull	33.2%	10	59.9%	13.3%
Europe[24]	Rendering and plastering	Weibull	52.4%	14	72.5%	24.0%
	Masonry	Weibull	18.8%	12	52.2%	1.7%
	Repairing and maintenance	Weibull	33.2%	10	59.9%	13.3%
US[24]	Rendering and plastering	Weibull	39.4%	12	57.2%	12.9%
	Masonry	Weibull	32.8%	12	43.8%	12.5%
	Repairing and maintenance	Weibull	31.8%	12	72.2%	12.6%
Rest of the world[24]	Rendering and plastering	Weibull	52.4%	14	72.5%	24.0%
	Masonry	Weibull	18.8%	12	52.2%	1.7%
	Repairing and maintenance	Weibull	33.2%	10	59.9%	13.3%

Supplementary Table 15

Supplementary Table 15 | Thickness of different mortar uses

Mortar use	Distribution	Scale	Shape	Max	Min
Rendering and plastering[24]	Weibull	22	4	80	3
Masonry[24]	Weibull	11	8	20	5
Repairing and maintenance[24]	Weibull	26.8	7	50	10

Supplementary Table 16

Supplementary Table 16 | Proportions of masonry walls with rendering

Rendering type	Distribution	Mode	Max	Min
Both sides	Triangular	60%	90%	40%
Only one side	Triangular	30%	50%	10%
Without rendering	Triangular	10%	20%	0%

Supplementary Note 1. Geographic coverage and aggregation

We aggregated 184 countries or districts into 10 regions (see SUPPLEMENTRAY TABLE 3 and SUPPLEMENTARY TABLE 4), because country-specific modeling requires country-specific assumptions on future stock development: a global model cannot reflect the differences between industrialized and developing regions.

Supplementary Note 2. Dynamic material flow analysis model

We employed a dynamic material flow analysis (MFA) model for simulating the past, present, and future stocks and flows of cement related materials: a top-down stock-flow estimation approach was developed for quantifying the retrospective cement cycle (1931-2014) in our previous study[1]; a stock-driven approach was developed for exploring the prospective cement cycle (2015-2100). The cement cycle is primarily characterized by un-hydrated hydraulic cement equivalent, namely the un-hydrated cement embodied in the relevant physical flows along the cement cycle, excluding inert materials that are used as aggregate in concrete and mortar.

Supplementary Note 2.1. Top-down stock-flow estimation approach (Retrospective cycle: 1930-2014)

The top-down stock-flow estimation approach is based on time series of historical inflow data, including production and trade statistics (for details of methods and data sources, we refer to the supporting information of our previous study[1]).

$$AC_{t_n} = CP_{t_n} + CI_{t_n} - CE_{t_n} + CPI_{t_n} - CPE_{t_n} \quad (1)$$

$$IN_{i,j,t_n} = AC_{t_n} \times IP_i \times EP_j \quad (2)$$

where AC_{t_n} denotes the apparent consumption of cement at a certain year t_n; CP_{t_n} denotes the production of cement at a certain year t_n; $CI_{t_n}$and CE_{t_n} denote the import and export of cement, respectively; CPI_{t_n}, and CPE_{t_n} denote the import and export of cement products (cement applications, e.g., articles made of cement), respectively; IN_{i,j,t_n} denotes the inflow of cement product i to end-use sector j at a certain year t_n; IP_i denotes the market share of cement used for cement product i; and EP_j denotes the market share of cement used for end-use sector j.

Given the inflows derived from the apparent consumption data, the in-use cement stock ST_{i,t_n} in one end-use sector at a certain year t_n is the sum of apparent consumption survived in each year.

$$ST_{i,j,t_n} = \sum_{t_0}^{t_n} S_j(t, t_n) \times IN_{i,j,t} dt \quad (3)$$

where $S_j(t, t_n)$ denotes the survival rate distribution function, which represents the survival rate after given specified time $t - t_n$, i.e., it is the complementary cumulative distribution function of lifetime distribution.

Landfilled cement kiln dust (CKD) was estimated according to cement production, clinker-to-cement ratio, CKD generation rate based on clinker, and proportion of CKD landfilled. Construction waste generation was estimated according to the loss rate of cement for cement products in the construction stage.

Demolition wastes (outflows) from in-use stocks were simulated according to inflows of cement and demolition rate distribution functions that directly represent the cumulative distribution function of lifetime distribution of end-use products.

$$O_{j,t_n} = \int_{t_0}^{t_n} L_j(t, t_n) \times IN_{j,t} dt \quad (4)$$

$$L_j(t, t_n) = \frac{dS_j(t_n - t)}{dt} \quad (5)$$

where $L_j(t, t_n)$ denotes the lifetime distribution function of an end-use product, which is the probability that a product entering at time t and leaving use at the time t_n.

In this model, we adopted lifetimes with the normal distributions from our previous study[1]. The mean of the normal distributions is determined based on a comprehensive literature review conducted in our previous study[1]. The standard deviation of the normal distribution is set as 1/5 of the mean, which is smaller than those employed in the previous DMFA studies on steel and aluminum[2,3]. This is because cement is ubiquitously used in buildings and structures of which the demolition tends to concentrate around the mean of the lifetime. Unlike cement, steel and aluminum are used in products of which the discarding tends to happen more evenly. One can expect that if the standard deviation is greater or smaller, the curve of lifetime distribution will have a longer or shorter tail.

Supplementary Note 2.2. Stock-driven approach (Prospective cycle: 2015-2100)

The future cement cycle was simulated by a stock-driven approach, using the in-use stock as the main driver[4]. The future flows were computed backward from assumed stock patterns and product lifetime. The stock-driven approach was first introduced by Müller[5] and has been extensively applied to materials (e.g., steel[2,6,7], aluminum[3], and copper[8]) with a long service lifetime. The stock-driven approach employed in these studies is based on a general hypothesis that all world regions will eventually benefit from the same services provided by in-use stocks as matured societies do today[2,9].

In order to choose saturation level and time independently for the different regions, we used a four-parameter logistic and Gompertz combined model[3] to explore the growth curve of future per-capita cement stock.

$$ST_{j,t_n} = \frac{ST_{j,sat}}{1 + (\frac{ST_{j,sat}}{SP_{j,0}} - 1) \times exp\,(A \times (1 - exp\,(B \times (t_n - t_0))))} \quad (6)$$

where ST_{j,t_n} denotes per capita stock at year t_n; $ST_{j,sat}$ refers to per capita stock saturation level; SP_0 refers to the beginning of per capita stock ($t_0 = 2014$); A and B denote parameters that determine the curve of growth patterns and when the per capita stock reaches 98% of the saturation level at a given time.

The inflows of cement to end-use sectors were determined as the sum of the net additions to stock and demolition wastes.

$$IN_{j,t_n} = ST_{j,t_n} \times P_{t_n} - ST_{j,t_{n-1}} \times P_{t_{n-1}} + O_{j,t_n} \quad (7)$$

$$O_{j,t_n} = \int_{t_0}^{t_{n-1}} L_j(t, t_n) \times IN_{j,t} dt \quad \textbf{(8)}$$

where P_{t_n} denotes population. The population data were taken from the medium variant of the United Nations population forecast up to 2100[10].

Supplementary Note 2.3. Scenario indicators by regions and by end-use sectors

SUPPLEMENTARY FIGURE 11 provides an overview of saturation levels and saturation times chosen for the different end-use sectors and regions. Our central premise is that the cement stocks in all regions will eventually saturate at a certain level and a certain time. Nine stock-driven scenarios were created for the future development of cement stocks in different end-use sectors up to 2100, varying by stock saturation level (t/capita) and approximate time of reaching saturation (reaching 98% of the saturation level). Given the regional heterogeneity of socioeconomic and geographic circumstances, we set varying saturation levels (i.e., low, medium, and high) and times (fast, medium, slow) in different regions to model a smooth transition of the development of cement stocks and flows.

According to the observations on historical cement stocks[1], we assumed the saturation times of five regions (i.e., North America, Europe, Commonwealth of Independent States, Middle East, and Developed Asia & Oceania) would be comparatively earlier. This is because the stock level in these regions reached a considerably high level, and its growth started to slow down. We assumed the saturation times of four regions (i.e., Latin America & Caribbean, Africa, India, and Developing Asia), where the stock level maintained at a low level, would be comparatively later. For China, per capita stock reached a high level and grew exponentially, and hence, we assumed the saturation times are even earlier.

We assumed that eight regions (i.e., Latin America & Caribbean, Europe, Commonwealth of Independent States, Africa, Middle East, India, Developed Asia & Oceania, and Developing Asia) would reach the same saturation levels. For North America and China, their saturation levels were slightly adapted to smooth the transition of the development of cement stocks and flows (see SUPPLEMENTARY TABLE 2).

Supplementary Note 2.4. Cement inflows (sectoral and total)

The stock-driven approach was employed to simulate inflows and outflows from 2015 to 2100. Annual cement inflows were driven by changes in cement stocks and annual cement outflows, as shown in Eq.7. The changes in cement stocks were determined by population and per capita cement stock. Annual cement outflows were determined by historical cement inflows and lifetime. The stock-driven simulations were conducted by sectors, and the sectoral outputs were subsequently used to calculate the CO_2 emissions and CO_2 uptake along the entire cement cycle. Intermediate outputs from the simulations are shown in the following figures.

In all of the nine stock-driven scenarios, the global cement demand reaches a low in 2030 (ranging from 3.4 to 4.6 Gt yr^{-1}), rises rapidly thereafter, and climbs up to a high in 2050 (ranging from 5.2 to 7.9 Gt yr^{-1}). Higher saturation levels and earlier saturation times bring forward the peak of cement demand, leading to varying increase factors (1.8-2.2 times) by 2100 compared to the 2014 level (4.2 Gt yr^{-1}). Our estimates of cement demand in the year 2050 (5.2-7.9 Gt yr^{-1}) are higher than those estimated by the International Energy Agency technology roadmap for the global cement industry (4.7-

5.1 Gt yr^{-1})[11,12]. The latter estimates are based on GDP growth, which is a less robust predictor of long-term material use[13].

Supplementary Note 3. Cement technology roadmap

In cement production, CO_2 emissions are directly generated during the production of clinker. Clinker is a nodular intermediate product that is then finely inter-ground with a certain proportion of calcium sulfate (mainly gypsum)[14] into a grey powder, ordinary Portland cement. It may also be mixed with other (cementitious) materials to make blended Portland cement. Indirect CO_2 emissions primarily arise from electricity use in quarrying, crushing and grinding of raw materials, blending and grinding of clinker, and operating of the rotary kiln.

We extracted five 'supply-side' mitigation measures for CO_2 emission reduction in cement production based on the Cement Technology Roadmap[11,12]: thermal efficiency (E-M1), electric efficiency (E-M2), alternative fuel (E-M3), clinker substitution (E-M4), and carbon capture and storage (E-M5). The roadmap is based on model data for the global cement industry in the context of IEA's Energy Technology Perspectives (ETP) BLUE Map scenario[15] and a set of technology papers developed by the European Cement Research Academy (ECRA). The ETP BLUE map scenario is underpinned by a bottom-up MARKAL (an acronym for MARKet and ALlocation) model that uses cost optimization to identify least-cost mixes of energy technologies and fuels to meet energy demand, given constraints such as the availability of natural resources[12]. Based on an understanding of technology mitigation options and their status quo in the global cement industry (see SUPPLEMENTARY TABLE 5 derived from Cement Sustainability Initiative (CSI)'s "Getting the Numbers Right (GNR)" data[16]), the roadmap outlines the implementation timeline and reduction potentials of the five mitigation measures. GNR database consists of data collected from individual companies. According to CSI's reporting standard[17], direct CO_2 emissions occurring from sources that are owned or controlled by the company and indirect CO_2 emissions from the generation of purchased electricity consumed in the company's owned or controlled equipment are included in the CO_2 emissions accounting. CO_2 emissions from off-site transports of mineral inputs and products are not included. These off-site CO_2 emissions are typically small and difficult to quantify consistently, because these transports are often carried out by third parties.

Uncertainties of CO_2 emissions were evaluated by the Monte Carlo method, following the practice recommended by the 2006 IPCC guidelines for National Greenhouse Gas Inventories[14]. Due to the fact that GNR database is based on company-level reporting, we applied the uncertainty ranges for company-level emissions factors:

- Values for process emission factor per tonne of clinker (kg CO_2/t clinker) were randomly sampled from a Normal distribution with a relative standard deviation of 1.5%[14].
- Values for clinker-to-cement ratio (t clinker/t cement) were randomly sampled from a Normal distribution with a relative standard deviation of 1.5%[14].
- Values for thermal efficiency (MJ/t clinker) were randomly sampled from a Normal distribution with a relative standard deviation of 2.5%[14].
- Values for thermal emission factor (kg CO_2/MJ) were randomly sampled from a Normal distribution with a relative standard deviation of 1.5%[18].
- Values for electric efficiency (kWh/t cement) were randomly sampled from a Normal distribution with a relative standard deviation of 2.5%[14].
- Values for electric emission factor (kg CO_2/kWh) were randomly sampled from a Normal distribution with a relative standard deviation of 1.5%[18].

Supplementary Note 3.1. Thermal efficiency

Theoretically, the minimum thermal energy demand for clinker production, including raw material drying and endothermic reactions of raw materials with required temperatures up to 1450˚C, is 1850-2800 MJ/t clinker[19,20]. The thermal efficiency of cement production is mainly determined by six factors: (i) moisture content and chemical composition of raw materials, (ii) types and burnability of raw materials; (iii) production capacity of the plant; (iv) technical status of the plant; (v) caloric value and reactivity of fuel mix; and (vi) kiln operation.

The average kiln capacity of cement plants will increase globally due to: i) the maximum capacities of cement kilns are expected to increase in the future (already increased significantly from ca. 5000 t/day in the 1990s to ca. 10000 t/day for today); ii) existing smaller cement kiln is to be gradually phased out by larger capacities. Current best available technology (BAT) for six-stage pre-heater and pre-calcination kilns is in the range of 2900MJ/t clinker and 3300 MJ/t clinker[15]. Cement production is highly capital intensive, and the lifetime of cement plants is typically 30-50 years. However, up-to-date equipment is not only found in emerging markets (e.g., Asia and some parts of Eastern Europe) but also in existing cement plants because their original equipment has been replaced with modernized equipment continuously.

Based on the above understanding of thermal energy technologies, it was expected that the thermal energy efficiency of cement production might decrease to 3300 MJ/t clinker by 2030 and 3200 MJ/t clinker by 2050[19,20]. Further, we assumed that the thermal energy efficiency of cement production might decrease to 2900 MJ/t clinker (BAT) by 2100. We used a curved line to fit the trend of thermal energy demand in the future.

Supplementary Note 3.2. Electric efficiency

In cement production using a dry process, the typical breakdown of electric energy consumption is[19,20] 5% for raw material extraction and blending, 24% for raw material grinding, 6% for raw material homogenization, 22% for rotary kiln operation, 38% for cement grinding, and 5% for conveying, packing and loading. Therefore, the grinding technology has a significant impact on electric consumption, meaning that a significant improvement in electric efficiency requires replacing outdated technologies (e.g., ball mills) to modernized technologies (e.g., highly efficient vertical roller mills or high-pressure grinding rolls). Grinding technologies in emerging markets are typically more updated than established markets because retrofitting the grinding technologies are highly capital intensive. There are no real breakthrough technologies in sight today; however, continuous update of grinding technologies are to be expected in existing cement plants. The roadmap assumed that electric energy efficiency might decrease to 92 KWh/t cement by 2050. We assumed the electric efficiency of cement production in those countries where electric technologies were already sufficiently updated would not decrease in the future.

Supplementary Note 3.3. Alternative fuel

Conventional fuels used in cement production can be substituted by alternative fuels, such as waste (including biogenic and non-biogenic waste sources) and biomass. Alternative fuels can be less carbon-intensive than coal. However, chemical constituents in alternative fuels can become incorporated into clinker and modify its properties/performance, which poses a constraint to their use.

Typical alternative fuels used in cement industry include pre-treated industrial and municipal solid wastes, discarded tires, waste oil and solvents, plastics, textiles and paper residues, and biomass (e.g., animal meal, logs, wood chips and residues, recycled wood and paper, agricultural residues, sewage sludge, and biomass crops). Promoting alternative fuels in cement production relies more on political and legal aspects, including waste management legislation, local waste collection networks, alternative fuel costs, and the level of social acceptance. The use of alternative fuels varies across regions, and therefore, we assumed that the share of fossil fuels will drop to 50% by 2030 and 40% by 2050 in EU[12], and drop to 80% by 2030 and 65% by 2050 in other regions. We used the fitted curves to predict the further replacement of fossil fuels up to 2100.

Supplementary Note 3.4. Clinker substitution

Portland cement clinker is the main component in most types of cement. Portland cement clinker is finely inter-ground with gypsum to control its setting properties and also sometimes blended with other (cementitious) materials to further modify performance, including blast furnace slag, fly ash, limestone, and natural volcanic materials. Clinker substitution is notably subject to the availability of these materials, their properties, the intended application of cement, national standards, and common practice and acceptance by contractors. It was assumed that the clinker-to-cement ratio would gradually decrease to 73% by 2050 in the high cement demand scenario (4.4 Gt at 2050)[12]; furthermore, we assumed that the clinker-to-cement ratio would gradually decrease to 70% by 2100, except regions (i.e., LAC and IN) where the clinker-to-cement ratio is already below 70%.

Supplementary Note 3.5. Carbon capture and storage (CCS)

During cement production, CO_2 is emitted from fuel combustion and limestone calcination in the kiln. Only a few CCS technologies seem appropriate for cement production: post-combustion technologies and oxyfuel technology. Chemical absorption with amine-based solvents is a promising post-combustion technology because its operational experiences are available from several industries (e.g., chemical and gas industry). In the long run, membrane technologies seem to be a candidate, while physical absorption or mineral carbonation appears to be less feasible due to a lack of sustained mass streams of sorbents[19,20]. Oxyfuel technology aims to generate a comparatively pure CO_2 stream by using oxygen instead of air in the cement kiln firing. Thus the purified CO_2 streams could be transported or stored with less effort[19,20].

Captured CO_2 by CCS technologies can also be further utilized (carbon capture and utilization; CCU) as a feedstock to produce chemicals and fuels; however, these CCU technologies are not commercially feasible and limited to laboratory scale[20]. From a technical point of view, carbon capture technologies in the cement industry are not likely to be commercially available before 2020[11,12]. A reduction efficiency of 80% has been tested out by 10-20 large kiln projects globally. In the medium-term, it is assumed that more full-scale demonstration projects will be realized between 2020 and 2030[11,12]; subsequently, approximately 25% of CO_2 emissions from cement production in 2050 would be captured by CCS technologies[11]. We extrapolated the trend further on up to 2100.

Supplementary Note 4. Cement carbonation model

To assess the CO_2 uptake by cement materials, we paired the dynamic material flow analysis model with a physicochemical carbonation model, which has been employed in both national scales[21–23] and global scale[24]. The physicochemical carbonation model was parameterized with data for exposed surface area, thickness, exposure conditions (atmospheric CO_2 concentrations in different regions and exposure time). Based on Fick's diffusion law[25] and carbonation rate coefficients under different exposure conditions, we modeled CO_2 uptake by different cement related materials in different stages of the cement cycle (see SUPPLEMENTARY FIGURE 103). To be consistent with the dynamic MFA model, we slightly tailored the physicochemical carbonation model by using a survival function[26,27], instead of an average lifetime[24], to determine the probability that cement materials enter into use survive after a certain time. In addition, we estimated carbon uptake by construction cement waste and cement kiln dust (CKD) using the generation rate of CKD per tonne of clinker and its carbonation fraction.

Data and uncertainties of parameters for the physicochemical carbonation model were adopted from the global cement carbonation model[24]. We employed the same Monte Carlo method used in the global cement carbonation model[24] to estimate uncertainties in CO_2 uptake. Data compilation and model descriptions are elaborated in the following sections. Data on annual cement production were taken from the DMFA model. Data on the clinker-to-cement ratio were collected from the Cement Sustainability Initiative's "Getting the Numbers Right" data[16], which is used for accounting CO_2 emissions in the cement production in Supplementary Note 3. Data on all other parameters are tabulated in Supplementary Note 4.5.

Supplementary Note 4.1. Uptake by cement kiln dust

Given its fine particle size, substantial carbonation of CKD occurs within the first two days in landfills, and then full carbonation is achieved within one year[28,29]. The annual uptake of CO_2 by CKD (U_{CKD}; kg) was calculated based on annual mass of cement production (W_p; kg cement), clinker-to-cement ratio ($C_{clinker}$; kg/kg cement), CKD generation rate based on clinker (r_{CKD}; kg/kg clinker), proportion of CKD landfilled ($r_{landfill}$; kg/kg CKD), average CaO content of CKD ($f_{CaO,CKD}$; kg/kg CKD), proportion of CaO within fully carbonated CKD that converts to $CaCO_3$ (γ_2; kg/kg; it is assumed to be 100%), and mole ratio of CO_2 to CaO (M_r; kg/kg; 0.785).

$$U_{CKD} = W_p \times C_{clinker} \times r_{CKD} \times r_{landfill} \times f_{CaO,CKD} \times \gamma_2 \times M_r \quad \textbf{(9)}$$

Supplementary Note 4.2. Uptake by construction wastes

Most of the construction wastes are in small pieces and are backfilled or landfilled after the completion of building projects. Given the fineness of construction wastes, wastes from mortar are assumed to be completely carbonated in one year, and wastes from concrete are assumed to be completely carbonated over five years.

The annual uptake of CO_2 by construction waste from concrete ($U_{w,c}$; kg) is calculated based on the annual mass of cement used for concrete (W_c; kg), loss rate of cement in the construction stage (l; kg/kg), annual carbonated mass fraction of construction waste from concrete (F_c; kg/kg; 0.2), clinker-to-cement mass ratio ($C_{clinker}$; kg/kg), average mass content of CaO in clinker (f_{CaO}; kg/kg), proportion

of CaO within fully carbonated cement that converts to $CaCO_3$ for concrete cement (γ; kg/kg), and mole ratio of CO_2 to CaO (M_r; kg/kg; 0.785).

$$U_{w,c} = W_c \times l \times F_c \times C_{clinker} \times f_{CaO} \times \gamma \times M_r \quad (10)$$

The annual uptake of CO_2 by construction waste from mortar ($U_{w,m}$; kg) is calculated based on the annual mass of cement used for mortar (W_m; kg), loss rate of cement in the construction stage (l; kg/kg), annual carbonated fraction of construction waste from mortar (F_m; kg/kg; 1), clinker-to-cement ratio ($C_{clinker}$; kg/kg), average CaO content of clinker (f_{CaO}; kg/kg), proportion of CaO within fully carbonated cement that converts to $CaCO_3$ for mortar cement (γ_1; kg/kg), and mole ratio of CO_2 to CaO (M_r; kg/kg; 0.785).

$$U_{w,m} = W_m \times l \times F_m \times C_{clinker} \times f_{CaO} \times \gamma_1 \times M_r \quad (11)$$

Supplementary Note 4.3. Uptake by concrete

Supplementary Note 4.3.1. Concrete in use stage

The effects of different concrete strength classes (i), exposure conditions ($\beta_{i,ec}$), cement additives (β_{ad}), CO_2 concentration (β_{CO_2}), coating and cover (β_{cc}) were explicitly modeled[21], because these parameters significantly affect the carbonation rate of concrete ($k_{c,i}$; $mm/\sqrt{yr}$).

$$k_{c,i} = \beta_{i,ec} \times \beta_{ad} \times \beta_{CO_2} \times \beta_{cc} \quad (12)$$

The carbonated depth of concrete ($d_{c,i}$; mm) over a certain period of time (t_{use}; yr) is calculated using Fick's diffusion law[25] (see SUPPLEMENTARY FIGURE 103A).

$$d_{c,i} = k_{c,i} \times \sqrt{t_{use}} \quad (13)$$

The volume of carbonated concrete during the use stage ($V_{c,i}$; m^3) is calculated based on the carbonated depth ($d_{c,i}$; mm), mass of cement used in different concrete classes ($W_{c,i}$; kg), concrete cement content ($C_{c,i}$; kg/m^3), and average thickness of concrete structures (T_w; mm).

$$V_{c,i} = d_{c,i} \times \frac{W_{c,i}}{C_{c,i} \times T_w} \quad (14)$$

The mass of carbonated cement used in concrete over a certain period of time during the use stage ($W_{c,use}$; kg) can be calculated based on the volume of carbonated concrete ($V_{c,i}$; m^3) and concrete cement content ($C_{c,i}$; kg/m^3).

$$W_{c,use} = \sum_{i=1}^{n} V_{c,i} \times C_{c,i} = \sum_{i=1}^{n} d_{c,i} \times \frac{W_{c,i}}{T_w} \quad (15)$$

The uptake of CO_2 by concrete during the use stage ($U_{c,use}$; kg) can be calculated based on the mass of carbonated cement ($W_{c,use}$; kg), clinker-to-cement ratio ($C_{clinker}$; kg/kg), average CaO content of clinker (f_{CaO}; kg/kg), proportion of CaO within fully carbonated cement that converts to $CaCO_3$ for concrete cement (γ; kg/kg), and ratio of CO_2 to CaO (M_r; kg/kg; 0.785).

$$U_{c,use} = W_{c,use} \times C_{clinker} \times f_{CaO} \times \gamma \times M_r \quad \textbf{(16)}$$

Supplementary Note 4.3.2. Concrete in demolition and secondary use stages

Carbonation of concrete in demolition and secondary use stages was modeled assuming a spherical shape for particles in demolition waste (see SUPPLEMENTARY FIGURE 103B), because concrete structures are usually crushed into small pieces in order to recycle steel and facilitate subsequent transport of demolition waste.

The carbonated fraction of concrete in demolition and secondary use stages is affected by treatment methods of demolition waste, particle sizes of demolition waste, and changing exposure conditions during demolition and secondary use stages[30,31]. After demolition, globally, more than 90% of crushed demolition waste will be buried in landfills or as part of road base and backfill aggregates (see SUPPLEMENTARY TABLE 1).

During the demolition stage, demolished concrete particles are exposed to open air and the average exposure time during the demolition stage (t_d; yr) is about 0.4 years[21,22,31]. Therefore, the carbonated fraction of demolished concrete during the demolition stage ($F_{d,i}$; kg/kg) are estimated by the range of particle size (radius (R; mm), minimum diameter (a; mm), and maximum diameter (b; mm)), carbonated depths during the demolition stage ($d_{d,c,i}$; mm), and carbonation coefficient in open air exposure conditions ($k_{d,c,i}$; $mm/\sqrt{yr}$), using Fick's diffusion law.

$$d_{d,c,i} = k_{d,c,i} \times \sqrt{t_d} \quad \textbf{(17)}$$

$$F_{d,i} = \begin{cases} 100\% - \dfrac{\int_a^b \frac{4}{3}\pi\left(R - d_{d,c,i}\right)^3}{\int_a^b \frac{4}{3}\pi R^3} \times 100\% & (a \geq 2d_{d,c,i}) \\ 100\% - \dfrac{\int_{2d_{d,c,i}}^b \frac{4}{3}\pi\left(R - d_{d,c,i}\right)^3}{\int_a^b \frac{4}{3}\pi R^3} \times 100\% & (a < 2d_{d,c,i} < b) \\ 100\% & (b \leq 2d_{d,c,i}) \end{cases} \quad \textbf{(18)}$$

The mass of carbonated cement used in concrete during the demolition stage ($W_{c,d}$ and $W_{c,i,d}$; kg) can be calculated based on the carbonated fraction of demolished concrete during the demolition stage ($F_{d,i}$; kg/kg) and mass of carbonated cement used in different concrete classes during the use stage ($W_{c,i,use}$; kg).

$$W_{c,i,d} = \left(W_{c,i} - W_{c,i,use}\right) \times F_{d,i} \quad \textbf{(19)}$$

$$W_{c,d} = \sum_{i=1}^{n} W_{c,i,d} \quad \textbf{(20)}$$

The uptake of CO_2 by concrete during the demolition stage ($U_{c,d}$; kg) can be estimated based on the mass of carbonated cement ($W_{c,d}$; kg), clinker-to-cement ratio ($C_{clinker}$; kg/kg), average CaO content of clinker (f_{CaO}; kg/kg), proportion of CaO within fully carbonated cement that converts to $CaCO_3$ (γ; kg/kg), and mole ratio of CO_2 to CaO (M_r; kg/kg; 0.785).

$$U_{c,d} = W_{c,d} \times C_{clinker} \times f_{CaO} \times \gamma \times M_r \quad \textbf{(21)}$$

During the secondary use stage, carbonation will continue but slow down, due to superficial carbonation during the demolition stage and the fact that most of the demolished concrete particles will be buried, either in landfills, backfills, or road base aggregates, and thus they are not exposed to air. Therefore, the carbonated fraction of demolished concrete during the secondary stage ($F_{s,i}$; kg/kg) over a certain period of time (t_s; yr) can be estimated by particle sizes (radius (R; mm), minimum diameter (a; mm), and maximum diameter (b; mm)), carbonated depths during the demolition stage ($d_{d,c,i}$; mm), carbonated depths during the secondary use stage ($d_{s,c,i}$; mm), total carbonated depths during demolition and secondary use stages ($d_{t,c,i}$; mm), and carbonation coefficient in buried conditions ($k_{s,c,i}$; $mm/\sqrt{yr}$), using Fick's diffusion law.

$$d_{s,c,i} = k_{s,c,i} \times \left(\sqrt{t_d + t_s} - \sqrt{t_d}\right) \quad \textbf{(22)}$$

$$d_{t,c,i} = d_{d,c,i} + d_{s,c,i} \quad \textbf{(23)}$$

$$F_{s,i} = \begin{cases} 100\% - \dfrac{\int_a^b \frac{4}{3}\pi\left(R - d_{s,c,i}\right)^3}{\int_a^b \frac{4}{3}\pi R^3} \times 100\% - F_{d,i} \quad (a \geq 2d_{t,c,i}) \\ 100\% - \dfrac{\int_{2d_{t,c,i}}^b \frac{4}{3}\pi\left(R - d_{s,c,i}\right)^3}{\int_a^b \frac{4}{3}\pi R^3} \times 100\% - F_{d,i} \quad (a < 2d_{t,c,i} < b) \\ 100\% - F_{d,i} \quad (b \leq 2d_{t,c,i}) \end{cases} \quad \textbf{(24)}$$

The mass of carbonated cement used in concrete during the secondary use stage ($W_{c,s}$ and $W_{c,i,s}$; kg) can be calculated based on the carbonated fraction of demolished concrete during the secondary use stage ($F_{s,i}$; kg/kg), mass of carbonated cement used in concrete during the use stage ($W_{c,i,use}$; kg), and mass of carbonated cement used in concrete during the demolition stage ($W_{c,i,d}$; kg).

$$W_{c,i,s} = (W_{c,i} - W_{c,i,use} - W_{c,i,d}) \times F_{s,i} \quad \textbf{(25)}$$

$$W_{c,s} = \sum_{i=1}^{n} W_{c,i,s} \quad \textbf{(26)}$$

The uptake of CO_2 by concrete during the secondary use stage ($U_{c,s}$; kg) can be estimated based on the mass of carbonated cement ($W_{c,s}$; kg), clinker-to-cement ratio ($C_{clinker}$; kg/kg), average CaO content of clinker (f_{CaO}; kg/kg), proportion of CaO within fully carbonated cement that converts to $CaCO_3$ (γ; kg/kg), and ratio of CO_2 to CaO (M_r; kg/kg; 0.785).

$$U_{c,s} = W_{c,s} \times C_{clinker} \times f_{CaO} \times \gamma \times M_r \quad \textbf{(27)}$$

Supplementary Note 4.4. Uptake by mortar

Supplementary Note 4.4.1. Mortar for rendering and plastering in use stage

Cement mortar is used for rendering and plastering, masonry, and repairing and maintenance of concrete structures.

The depth of carbonated mortar used for rendering and plastering (d_{rp}; mm) over a certain period of time (t_{use}; yr) can be calculated based on the carbonation rate of mortar (k_m; $mm/\sqrt{yr}$) using Fick's diffusion law (see SUPPLEMENTARY FIGURE 103A).

$$d_{rp} = k_m \times \sqrt{t_{use}} \quad \textbf{(28)}$$

The carbonated fraction of cement used for rendering and plastering (F_{rp}; kg/kg) can be calculated based on the depth of carbonated cement (d_{rp}; mm) and thickness of rendering and plastering (T_{rp}; mm).

$$F_{rp} = \frac{d_{rp}}{T_{rp}} \quad \textbf{(29)}$$

The uptake of CO_2 by mortar used for rendering and plastering ($U_{m,rp}$; kg) can be estimated based on the mass of cement used in mortar (W_m; kg), percentage of mortar used for rendering and plastering (r_{rp}; kg/kg), carbonated fraction of cement used for rendering and plastering (F_{rp}; kg/kg), clinker-to-cement ratio ($C_{clinker}$; kg/kg), average CaO content of clinker (f_{CaO}; kg/kg), proportion of CaO within fully carbonated cement that converts to $CaCO_3$ for mortar cement (γ_1; kg/kg), and ratio of CO_2 to CaO (M_r; kg/kg; 0.785).

$$U_{m,rp} = W_m \times r_{rp} \times F_{rp} \times C_{clinker} \times f_{CaO} \times \gamma_1 \times M_r \quad \textbf{(30)}$$

Supplementary Note 4.4.2. Mortar for masonry with rendering on both sides in use stage

Mortar is a workable paste used to bind masonry units together and fill and seal the irregular gaps between them. Masonry walls are covered by rendering mortar on both sides or only inside, or not rendered at all.

The depth of carbonated mortar for masonry rendered on both sides ($d_{ma,b}$; mm) over a certain period of time (t_{use}; yr) can be calculated based on the carbonation rate of mortar (k_m; $mm/\sqrt{yr}$), thickness of rendering and plastering (T_{rp}; mm), and time that mortar for rendering is fully carbonated (t_r; yr), using Fick's diffusion law (see SUPPLEMENTARY FIGURE 103A).

$$d_{ma,b} = \begin{cases} 0 & (t_{use} \leq t_r) \\ 2\left(k_m \times \sqrt{t_{use}} - T_{rp}\right) & (t_{use} > t_r) \end{cases} \quad \textbf{(31)}$$

The carbonated fraction of mortar used for masonry rendered on both sides ($F_{ma,b}$; kg/kg) can be calculated based on the depth of carbonated mortar ($d_{ma,b}$; mm) and thickness of masonry walls (T_w; mm).

$$F_{ma,b} = \frac{d_{ma,b}}{T_w} \times 100\% \quad (32)$$

The uptake of CO_2 by mortar used for masonry rendered on both sides ($U_{m,ma,b}$; kg) can be calculated based on the mass of cement used in mortar (W_m; kg), percentage of mortar used for masonry (r_{ma}), percentage of masonry walls with rendering on both sides (r_b), carbonated fraction of cement used for masonry rendered on both sides ($F_{ma,b}$; kg/kg), clinker-to-cement ratio ($C_{clinker}$; kg/kg), average CaO content of clinker (f_{CaO}; kg/kg), proportion of CaO within fully carbonated cement that converts to $CaCO_3$ for mortar cement (γ_1; kg/kg), and ratio of CO_2 to CaO (M_r; kg/kg; 0.785).

$$U_{m,ma,b} = W_m \times r_{ma} \times r_b \times F_{ma,b} \times C_{clinker} \times f_{CaO} \times \gamma_1 \times M_r \quad (33)$$

Supplementary Note 4.4.3. Mortar for masonry with rendering on only one side in use stage

The depth of carbonated mortar for masonry rendered on only one side ($d_{ma,o}$; mm) over a certain period of time (t_{use}; yr) can be calculated based on the carbonation rate of mortar (k_m; $mm/\sqrt{yr}$), thickness of rendering and plastering mortar (T_{rp}; mm), and time that mortar for rendering is fully carbonated (t_r; yr), using Fick's diffusion law (see SUPPLEMENTARY FIGURE 103A).

$$d_{ma,o} = \begin{cases} k_m \times \sqrt{t_{use}} & (t_{use} \le t_r) \\ k_m \times \sqrt{t} + \left(k_m \times \sqrt{t_{use}} - T_{rp}\right) & (t_{use} > t_r) \end{cases} \quad (34)$$

The carbonated fraction of mortar used for masonry rendered on only one side ($F_{ma,o}$; kg/kg) can be calculated based on the depth of carbonated mortar ($d_{ma,o}$; mm) and thickness of masonry walls (T_w; mm).

$$F_{ma,o} = \frac{d_{ma,o}}{T_w} \times 100\% \quad (35)$$

The uptake of CO_2 by mortar used for masonry rendered on only one side ($U_{m,ma,o}$; kg) can be calculated based on the mass of cement used in mortar (W_m; kg), percentage of mortar used for masonry (r_{ma}), percentage of masonry walls with rendering on only one side (r_o), carbonated fraction of cement used for masonry rendered on only one side ($F_{ma,o}$; kg/kg), clinker-to-cement ratio ($C_{clinker}$; kg/kg), average CaO content of clinker (f_{CaO}; kg/kg), proportion of CaO within fully carbonated cement that converts to $CaCO_3$ for mortar cement (γ_1; kg/kg), and ratio of CO_2 to CaO (M_r; kg/kg; 0.785).

$$U_{m,ma,o} = W_m \times r_{ma} \times r_o \times F_{ma,b} \times C_{clinker} \times f_{CaO} \times \gamma_1 \times M_r \quad (36)$$

Supplementary Note 4.4.4. Mortar for masonry without rendering in use stage

The depth of carbonated mortar for masonry without rendering ($d_{ma,n}$; mm) over a certain period of time (t_{use}; yr) can be calculated based on the carbonated rate of mortar (k_m; $mm/\sqrt{yr}$) using Fick's diffusion law (see SUPPLEMENTARY FIGURE 103A).

$$d_{ma,n} = 2k_m \times \sqrt{t_{use}} \quad (37)$$

The carbonated fraction of mortar used for masonry without rendering ($F_{ma,n}$; kg/kg) can be calculated based on the depth of carbonated mortar ($d_{ma,n}$; mm) and thickness of masonry walls (T_w; mm).

$$F_{ma,n} = \frac{d_{ma,n}}{T_w} \times 100\% \quad (38)$$

The uptake of CO_2 by mortar used for masonry without rendering ($U_{m,ma,n}$; kg) can be calculated based on the mass of cement used in mortar (W_m; kg), percentage of mortar used for masonry (r_{ma}), percentage of masonry walls without rendering (r_n), carbonation fraction of cement used for masonry without rendering ($F_{ma,n}$; kg/kg), clinker-to-cement ratio ($C_{clinker}$; kg/kg), average CaO content of clinker (f_{CaO}; kg/kg), proportion of CaO within fully carbonated cement that converts to $CaCO_3$ for mortar cement (γ_1; kg/kg), and ratio of CO_2 to CaO (M_r; kg/kg; 0.785).

$$U_{m,ma,n} = W_m \times r_{ma} \times r_n \times F_{ma,n} \times C_{clinker} \times f_{CaO} \times \gamma_1 \times M_r \quad (39)$$

Supplementary Note 4.4.5. Mortar for repairing and maintenance in use stage

The depth of carbonated mortar used for repairing and maintaining (d_{rm}; mm) over a certain period of time (t_{use}; yr) can be calculated based on carbonation rate of mortar (k_m; $mm/\sqrt{yr}$) using Fick's diffusion law (see SUPPLEMENTARY FIGURE 103A).

$$d_{rm} = k_m \times \sqrt{t_{use}} \quad (40)$$

The carbonated fraction of cement used for repairing and maintenance (F_{rm}; kg/kg) can be calculated based on the depth of carbonated cement (d_{rm}; mm) and thickness of repairing and maintenance (T_{rm}; mm).

$$F_{rm} = \frac{d_{rm}}{T_{rm}} \quad (41)$$

The uptake of CO_2 by mortar used for repairing and maintenance ($U_{m,rm}$; kg) can be estimated based on the mass of cement used in mortar (W_m; kg), percentage of mortar used for repairing and maintenance (r_{rm}), carbonated fraction of cement used for repairing and maintenance (F_{rm}; kg), clinker-to-cement ratio ($C_{clinker}$; kg/kg), average CaO content of clinker (f_{CaO}; kg/kg), proportion of

CaO within fully carbonated cement that converts to $CaCO_3$ for mortar cement (γ_1; kg/kg), and ratio of CO_2 to CaO (M_r; kg/kg; 0.785).

$$U_{m,rm} = W_m \times r_{rm} \times F_{rm} \times C_{clinker} \times f_{CaO} \times \gamma_1 \times M_r \quad \textbf{(42)}$$

Supplementary Note 4.4.6. Mortar in demolition and secondary use stages

Mortar in the demolition and secondary use stages binds much less CO_2 than in the use stage, because the thin layer and large exposure area of mortar translate into rapid carbonation before its end-of-life. Carbonation of mortar in the demolition and secondary use stages was modeled using the same assumptions on particle sizes and modeling procedures as carbonation of concrete.

Supplementary Note 4.5. Data compilation and uncertainties

According to the global cement carbonation model[24], critical causes of uncertainties associated with carbonation were identified and their impacts on simulation results were evaluated by the Monte Carlo method recommended by the 2006 IPCC guidelines for National Greenhouse Gas Inventories[32].

Supplementary Note 4.5.1. CaO content in clinker

Values for the CaO content in clinker were randomly sampled from a Triangular distribution with a mode of 65%, a maximum of 67%, and a minimum of 60%, as recommended by the 2006 IPCC guidelines for National Greenhouse Gas Inventories in Volume III[32].

Supplementary Note 4.5.2. Proportion of CaO converted to $CaCO_3$

Values for the proportion of CaO converted to $CaCO_3$ in concrete were randomly sampled from a truncated Weibull distribution with a scale of 86%, a shape of 25, a maximum of 90%, and a minimum of 50%, according to the previous studies[21,22,33,34].

Values for the proportion of CaO converted to $CaCO_3$ in mortar were randomly sampled from a truncated Weibull distribution with a scale of 92%, a shape of 20, a maximum of 100%, and a minimum of 50%, according to results from experimental tests[24].

Supplementary Note 4.5.3. Market shares of cement used for concrete

Values for market shares of concrete cement were sampled from truncated Weibull distributions (see SUPPLEMENTARY TABLE 7).

Supplementary Note 4.5.4. Market shares of concrete strength classes

Values for the market shares of different concrete strength classes were randomly sampled from truncated Weibull distributions (see SUPPLEMENTARY TABLE 8).

Supplementary Note 4.5.5. Cement content of concrete

Values for the cement content of concrete (kg/m^3) were randomly sampled from Uniform distributions (see SUPPLEMENTARY TABLE 9).

Supplementary Note 4.5.6. Carbonation rates of different strength classes

Values for the carbonation rates ($mm/\sqrt{yr}$) of different strength classes were randomly sampled from Uniform distributions (see SUPPLEMENTARY TABLE 10).

Supplementary Note 4.5.7. Correction factor of cement additives

Values for the correction factor of cement additives were sampled from a truncated Weibull distribution with a scale of 1.16, a shape of 20, a maximum of 1.3, and a minimum of 1[21]. Cement additives may increase the carbonation rate of cement materials.

Supplementary Note 4.5.8. Correction factor of CO_2 concentration

Values for the correction factor of CO_2 concentration were sampled from a truncated Weibull distribution with a scale of 1.18, a shape of 25, a maximum of 1.41, and a minimum of 0.93[40,41].

Supplementary Note 4.5.9. Correction factor of coating and cover

Values for the correction factor of coating and cover were sampled from a truncated Weibull distribution with a scale of 1.0, a shape of 6.0, a maximum of 1.0, and a minimum of 0.5[22,42–47].

Supplementary Note 4.5.10. Breakdown of demolition waste by particle sizes

Values for the percentages of different particle sizes were sampled from Uniform distributions (see SUPPLEMENTARY TABLE 11).

Supplementary Note 4.5.11. Exposure time during the demolition stage

Values for the exposure time (yr) during the demolition stage were sampled from truncated Weibull distributions (see SUPPLEMENTARY TABLE 12).

Supplementary Note 4.5.12. Breakdown of secondary uses of demolition wastes

The fate of demolition waste varies from region to region, of which data were taken from different sources (China[48], Europe[31], US[30], and rest of world[31,49]). Values of the percentages of secondary uses of demolition wastes were sampled from Triangular distributions (see SUPPLEMENTARY TABLE 1).

Supplementary Note 4.5.13. Market shares of cement used for mortar

Values for market shares of mortar cement were sampled from truncated Weibull distributions (see SUPPLEMENTARY TABLE 13).

Supplementary Note 4.5.14. Breakdown of mortar uses

Values for the percentages of mortar uses were sampled from truncated Weibull distributions (see SUPPLEMENTARY TABLE 14).

Supplementary Note 4.5.15. Thickness of different mortar uses

Values for the thickness (mm) of different mortar uses were sampled from truncated Weibull distributions (see SUPPLEMENTARY TABLE 15).

Supplementary Note 4.5.16. Proportions of masonry walls with rendering

Values for the proportions of masonry walls with rendering were sampled from Triangular distributions (see SUPPLEMENTARY TABLE 16), according to results from experimental tests[24].

Supplementary Note 4.5.17. Thickness of concrete structures

Values for the thickness (mm) of concrete structures were sampled from a Uniform distribution with a maximum of 610 and a minimum of 60[24].

Supplementary Note 4.5.18. Carbonation rate of mortar

Values for the carbonation rate ($mm/\sqrt{yr}$) of mortar were sampled from a Triangular distribution with a mode of 19.6, a maximum of 36.8, and a minimum of 6.1[24].

Supplementary Note 4.5.19. Loss rates of cement in the construction stage

Values for the loss rates of cement from concrete and mortar in the construction stage were sampled from a Triangular distribution with a mode of 1.5%, a maximum of 3.0%, and a minimum of 1.0%[50,51].

Supplementary Note 4.5.20. Carbonation time of construction waste from concrete

Values for the carbonation time (yr) of construction waste from concrete were sampled from a Triangular distribution with a mode of 5, a maximum of 10, and a minimum of 1[52,53].

Supplementary Note 4.5.21. CKD generation rate based on clinker

Values for the CKD generation rate based on clinker were sampled from a Triangular distribution with a mode of 6.0%, a maximum of 11.5%, and a minimum of 4.1%[54].

Supplementary Note 4.5.22. Proportion of landfilled CKD

Values for the proportion of landfilled CKD were sampled from a Triangular distribution with a mode of 80.0%, a maximum of 90.0%, and a minimum of 52.0%[55,56].

Supplementary Note 4.5.23. CaO content of CKD

Values for the CaO content of CKD were sampled from a truncated Normal distribution with a mean of 44.0%, a standard deviation of 8.01%, a maximum of 61.23%, and a minimum of 19.40%[56,57].

Supplementary Note 5. Uncertainties and sensitivities

Supplementary Note 5.1. Sensitivity to lifetime

We conducted comprehensive sensitivity analyses on three representative regions: North America, Europe, and China. We increased or decreased the lifetime by 20% and reran the model.

Abbreviations for scenarios are as follows:

- S1-3: Low-Fast; Low-Moderate; Low-Slow
- S4-6: Medium-Fast; Medium-Moderate; Medium-Slow
- S7-9: High-Fast; High-Moderate; High-Slow

Supplementary Note 5.2. Sensitivity to population

We conducted comprehensive sensitivity analyses on three representative regions: North America, Europe, and China. We changed the population parameter and reran the model. We took three variants of population data from the United Nations population forecast.

Abbreviations for scenarios are as follows:

- S1-3: Low-Fast; Low-Moderate; Low-Slow
- S4-6: Medium-Fast; Medium-Moderate; Medium-Slow
- S7-9: High-Fast; High-Moderate; High-Slow

Supplementary Note 5.3. Lifetime extension

To examine the effect of lifetime extension, we performed an additional sensitivity analysis on China's cement cycle. We changed the lifetime parameter of the stock-driven model, assuming the average lifetime of the end-use products (e.g., buildings and structures) will gradually increase to 70 years. The justification of this assumption is: the duration of land use right in China is 70 years, and since steel-concrete structures are becoming prevalent and replacing low-quality building materials, building quality in China will progressively improve in the coming decades. Therefore, it is reasonable to assume that the maximum lifetime of buildings and structures in China would increase to 70 years.

SUPPLEMENTARY FIGURE 75 demonstrates that the extension of lifetime will decrease both CO_2 uptake and emissions in all scenarios due to its effects in reducing cement inflows and outflows, eventually resulting in diminished CO_2 balance.

Supplementary References

1. Cao, Z., Shen, L., Løvik, A. N., Müller, D. B. & Liu, G. Elaborating the History of Our Cementing Societies: An in-Use Stock Perspective. *Environ. Sci. Technol.* **51**, 11468–11475 (2017).
2. Pauliuk, S., Milford, R. L., Müller, D. B. & Allwood, J. M. The Steel Scrap Age. *Environmental Science & Technology* **47**, 3448–3454 (2013).
3. Liu, G., Bangs, C. E. & Müller, D. B. Stock dynamics and emission pathways of the global aluminium cycle. *Nature Climate Change* **3**, 338–342 (2012).
4. Müller, E., Hilty, L. M., Widmer, R., Schluep, M. & Faulstich, M. Modeling Metal Stocks and Flows: A Review of Dynamic Material Flow Analysis Methods. *Environmental Science & Technology* **48**, 2102–2113 (2014).
5. Müller, D. B. Stock dynamics for forecasting material flows—Case study for housing in The Netherlands. *Ecological Economics* **59**, 142–156 (2006).
6. Hatayama, H., Daigo, I., Matsuno, Y. & Adachi, Y. Outlook of the World Steel Cycle Based on the Stock and Flow Dynamics. *Environmental Science & Technology* **44**, 6457–6463 (2010).
7. Pauliuk, S., Wang, T. & Müller, D. B. Moving Toward the Circular Economy: The Role of Stocks in the Chinese Steel Cycle. *Environmental Science & Technology* **46**, 148–154 (2012).
8. Bader, H.-P., Scheidegger, R., Wittmer, D. & Lichtensteiger, T. Copper flows in buildings, infrastructure and mobiles: a dynamic model and its application to Switzerland. *Clean Technologies and Environmental Policy* **13**, 87–101 (2011).
9. Müller, D. B., Wang, T., Duval, B. & Graedel, T. E. Exploring the engine of anthropogenic iron cycles. *Proceedings of the National Academy of Sciences* **103**, 16111–16116 (2006).
10. United Nations, Department of Economic and Social Affairs, Population Division. World Population Prospects: The 2017 Revision, DVD Edition. (2017).
11. IEA, International Energy Agency, WBCSD, World Business Council on Sustainable Development & CSI, Cement Sustainability Initiative. *Technology Roadmap: Low-Carbon Transition*

in the Cement Industry.
http://www.iea.org/publications/freepublications/publication/TechnologyRoadmapLowCarbonTransitionintheCementIndustry.pdf (2018).

12. International Energy Agency. *Cement Technology Roadmap: Carbon Emissions Reductions up to 2050*. (OECD, 2009). doi:10.1787/9789264088061-en.

13. Pauliuk, S., Arvesen, A., Stadler, K. & Hertwich, E. G. Industrial ecology in integrated assessment models. *Nature Clim. Change* **7**, 13–20 (2017).

14. Intergovernmental Panel on Climate Change (IPCC). *IPCC Guidelines for National Greenhouse Gas Inventories*. (2006).

15. International Energy Agency. *Energy Technology Perspectives: Scenarios & Strategies to 2050*. (OECD, 2008).

16. Cement Sustainability Initiative. Getting Number Right, GNR Project. https://www.wbcsdcement.org/GNR-2016/index.html.

17. Cement Sustainability Initiative. *CO2 and Energy Accounting and Reporting Standard for the Cement Industry*. (2011).

18. Liu, Z. *et al.* Reduced carbon emission estimates from fossil fuel combustion and cement production in China. *Nature* **524**, 335–338 (2015).

19. CSI, Cement Sustainability Initiative & European Cement Research Academy, ECRA. *Development of State of the Art Techniques in Cement Manufacturing: trying to look ahead*. (2009).

20. CSI, Cement Sustainability Initiative & European Cement Research Academy, ECRA. *Development of State of the Art Techniques in Cement Manufacturing: trying to look ahead, Revision 2017*. http://www.wbcsdcement.org/pdf/technology/CSI_ECRA_Technology_Papers_2017.pdf (2017).

21. Pade, C. & Guimaraes, M. The CO2 uptake of concrete in a 100 year perspective. *Cement and Concrete Research* **37**, 1348–1356 (2007).

22. Andersson, R., Fridh, K., Stripple, H. & Häglund, M. Calculating CO2 Uptake for Existing Concrete Structures during and after Service Life. *Environ. Sci. Technol.* **47**, 11625–11633 (2013).

23. Dodoo, A., Gustavsson, L. & Sathre, R. Carbon implications of end-of-life management of building materials. *Resources, Conservation and Recycling* **53**, 276–286 (2009).

24. Xi, F. *et al.* Substantial global carbon uptake by cement carbonation. *Nature Geoscience* **9**, 880–883 (2016).

25. Monteiro, I., Branco, F. A., Brito, J. de & Neves, R. Statistical analysis of the carbonation coefficient in open air concrete structures. *Construction and Building Materials* **29**, 263–269 (2012).

26. Müller, D. B. *et al. Service lifetimes of mineral end uses*. (2007).

27. Cai, W., Wan, L., Jiang, Y., Wang, C. & Lin, L. Short-Lived Buildings in China: Impacts on Water, Energy, and Carbon Emissions. *Environ. Sci. Technol.* **49**, 13921–13928 (2015).

28. Huntzinger, D. N., Gierke, J. S., Sutter, L. L., Kawatra, S. K. & Eisele, T. C. Mineral carbonation for carbon sequestration in cement kiln dust from waste piles. *Journal of Hazardous Materials* **168**, 31–37 (2009).

29. Huntzinger, D. N., Gierke, J. S., Kawatra, S. K., Eisele, T. C. & Sutter, L. L. Carbon Dioxide Sequestration in Cement Kiln Dust through Mineral Carbonation. *Environ. Sci. Technol.* **43**, 1986–1992 (2009).

30. Kelly, T. D. *Crushed cement concrete substitution for construction aggregates, a materials flow analysis*. (US Department of the Interior, US Geological Survey, 1998).

31. Engelsen, C. J., Mehus, J., Pade, C. & Sæther, D. H. *Carbon dioxide uptake in demolished and crushed concrete*. (2005).

32. Eggleston, S., Buendia, L., Miwa, K., Ngara, T. & Tanabe, K. *2006 IPCC guidelines for national greenhouse gas inventories*. vol. 5 (Institute for Global Environmental Strategies Hayama, Japan, 2006).

33. Gajda, J. Absorption of atmospheric carbon dioxide by portland cement concrete. *Portland Cement Association* (2001).

34. Chang, C.-F. & Chen, J.-W. The experimental investigation of concrete carbonation depth. *Cement and Concrete Research* **36**, 1760–1767 (2006).

35. ERMCO, European Ready Mixed Concrete Organization. *Ready-Mixed Concrete Industry Statistics 2001-2013*. (2014).

36. Jónsson, G. & Wallevik, O. Information on the use of concrete in Denmark, Sweden, Norway and Iceland. *Icelandic Build. Res. Inst* (2005).

37. Kelly Thomas, D. & Matos Grecia, R. *Historical Statistics for Mineral and Material Commodities in the United States*. (2016).

38. Low, M.-S. Material flow analysis of concrete in the United States. (Massachusetts Institute of Technology, 2005).

39. Nisbet, M. A., VanGeem, M. G., Gajda, J. & Marceau, M. Environmental life cycle inventory of portland cement concrete. *PCA R&D Serial* (2000).

40. Papadakis, V. G., Vayenas, C. G. & Fardis, M. N. Experimental investigation and mathematical modeling of the concrete carbonation problem. *Chemical Engineering Science* **46**, 1333–1338 (1991).

41. Yoon, I.-S., Çopuroğlu, O. & Park, K.-B. Effect of global climatic change on carbonation progress of concrete. *Atmospheric Environment* **41**, 7274–7285 (2007).

42. Lagerblad, B. Carbon dioxide uptake during concrete life cycle–state of the art. *Swedish Cement and Concrete Research Institute CBI, Stockholm* (2005).

43. Pommer, K. & Pade, C. *Guidelines: uptake of carbon dioxide in the life cycle inventory of concrete*. (Nordic Innovation Centre, 2006).

44. Klopfer, H. The carbonation of external concrete and how to combat it. *Bautenschutz Bausanieruniz* **3**, 86–97 (1978).

45. Moon, H. Y., Shin, D. G. & Choi, D. S. Evaluation of the durability of mortar and concrete applied with inorganic coating material and surface treatment system. *Construction and Building Materials* **21**, 362–369 (2007).

46. Seneviratne, A. M. G., Sergi, G. & Page, C. L. Performance characteristics of surface coatings applied to concrete for control of reinforcement corrosion. *Construction and Building Materials* **14**, 55–59 (2000).

47. Lo, T. Y., Liao, W., K. Wong, C. & Tang, W. Evaluation of carbonation resistance of paint coated concrete for buildings. *Construction and Building Materials* **107**, 299–306 (2016).

48. Lu, W. Waste recycling system material metabolism analysis model and its application. *Department of Environmental Science and Engineering, Tsinghua University, Beijing (in Chinese)* (2010).

49. Yang, K.-H., Seo, E.-A. & Tae, S.-H. Carbonation and CO2 uptake of concrete. *Environmental Impact Assessment Review* **46**, 43–52 (2014).

50. Zhou, H. *Construction and installation engineering budget manual*. (China Machine Press, 2003).

51. Lu, W. *et al.* An empirical investigation of construction and demolition waste generation rates in Shenzhen city, South China. *Waste Management* **31**, 680–687 (2011).

52. Huang, T., Shi, F., Tanikawa, H., Fei, J. & Han, J. Materials demand and environmental impact of buildings construction and demolition in China based on dynamic material flow analysis. *Resources, Conservation and Recycling* **72**, 91–101 (2013).

53. Bossink, B. A. G. & Brouwers, H. J. H. Construction Waste: Quantification and Source Evaluation. *Journal of Construction Engineering and Management* **122**, 55–60 (1996).

54. EPA, U. *Environmental Protection Agency. Report to congress on cement kiln dust*. (1993).

55. Bhatty, J. I., Miller, F. M. & Kosmatka, S. H. *Innovations in Portland cement manufacturing*. vol. 2004 (Portland Cement Association Washington^ eDC DC, 2004).

56. Khanna, O. S. Characterization and utilization of cement kiln dusts (CKDs) as partial replacements of Portland cement. (2009).

57. Sreekrishnavilasam, A., King, S. & Santagata, M. Characterization of fresh and landfilled cement kiln dust for reuse in construction applications. *Engineering Geology* **85**, 165–173 (2006).
