# Supplementary material for: The sponge effect and carbon emission mitigation potentials of the global cement cycle
Source: Nat Commun. 2020 Jul 29;11:3777. doi: 10.1038/s41467-020-17583-w (PMC7392754; doi:10.1038/s41467-020-17583-w)
Supplement: Supplementary file 3 — Reporting Summary [file 41467_2020_17583_MOESM3_ESM.pdf]

nature research

Corresponding author(s): Gang Liu

Last updated by author(s): Jun 19, 2020

Reporting Summary

Nature Research wishes to improve the reproducibility of the work that we publish. This form provides structure for consistency and transparency in reporting. For further information on Nature Research policies, see Authors & Referees and the Editorial Policy Checklist .

Statistics

For all statistical analyses, confirm that the following items are present in the figure legend, table legend, main text, or Methods section.

n/a	Confirmed	
☐	☒	The exact sample size (*n*) for each experimental group/condition, given as a discrete number and unit of measurement
☒	☐	A statement on whether measurements were taken from distinct samples or whether the same sample was measured repeatedly
☒	☐	The statistical test(s) used AND whether they are one- or two-sided<br>*Only common tests should be described solely by name; describe more complex techniques in the Methods section.*
☒	☐	A description of all covariates tested
☒	☐	A description of any assumptions or corrections, such as tests of normality and adjustment for multiple comparisons
☐	☒	A full description of the statistical parameters including central tendency (e.g. means) or other basic estimates (e.g. regression coefficient) AND variation (e.g. standard deviation) or associated estimates of uncertainty (e.g. confidence intervals)
☒	☐	For null hypothesis testing, the test statistic (e.g. *F*, *t*, *r*) with confidence intervals, effect sizes, degrees of freedom and *P* value noted<br>*Give P values as exact values whenever suitable.*
☒	☐	For Bayesian analysis, information on the choice of priors and Markov chain Monte Carlo settings
☒	☐	For hierarchical and complex designs, identification of the appropriate level for tests and full reporting of outcomes
☒	☐	Estimates of effect sizes (e.g. Cohen's *d*, Pearson's *r*), indicating how they were calculated

Our web collection on statistics for biologists contains articles on many of the points above.

Software and code

Policy information about availability of computer code

Data collection	Data used in this study are from public sources: international databases, national databases, industry reports, and peer-reviewed journals. No software was used for data collection.
Data analysis	Data analyses are conducted in R (version 3.5.2) and Excel (version 2016). The Sankey diagram in Figure 1 was generated by Circular Sankey developed by Industrial Ecology Freiburg (http://www.visualisation.industrialecology.uni-freiburg.de/). Codes regarding cement carbonation modeling are deposited in zenodo (https://doi.org/10.5281/zenodo.3384828).

For manuscripts utilizing custom algorithms or software that are central to the research but not yet described in published literature, software must be made available to editors/reviewers. We strongly encourage code deposition in a community repository (e.g. GitHub). See the Nature Research guidelines for submitting code & software for further information.

Data

Policy information about availability of data

All manuscripts must include a data availability statement. This statement should provide the following information, where applicable:

- Accession codes, unique identifiers, or web links for publicly available datasets
- A list of figures that have associated raw data
- A description of any restrictions on data availability

The data that support the findings of this study are available from the Supplementary Information and Supplementary Source Data. CO2 emission intensities of cement production are available from Getting the Numbers Right (GNR) database (https://www.wbcsdcement.org/GNR-2016/index.html). Data and parameter uncertainties for the physicochemical carbonation model are available from the global cement carbonation model (ref. 5 in main text).

October 2018

Field-specific reporting

Please select the one below that is the best fit for your research. If you are not sure, read the appropriate sections before making your selection.

- [ ] Life sciences
- [ ] Behavioural & social sciences
- [x] Ecological, evolutionary & environmental sciences

For a reference copy of the document with all sections, see nature.com/documents/nr-reporting-summary-flat.pdf

Ecological, evolutionary & environmental sciences study design

All studies must disclose on these points even when the disclosure is negative.

Study description	Quantitative models were developed to analyze the past, present, and future stocks and flows of cement-related materials, the effects of CO2 emissions mitigation measures in cement production, and the atmospheric CO2 absorption by cement-related materials over time. Monte Carlo simulations were run to estimate uncertainties in CO2 emissions and CO2 uptake.
Research sample	No first-hand samples were produced in this study. CO2 emission intensities of cement production were collected from Getting the Numbers Right (GNR) database (https://www.wbcsdcement.org/GNR-2016/index.html). Data and parameter uncertainties for the physicochemical carbonation model were derived from the global cement carbonation model (ref. 5 in main text).
Sampling strategy	This study covers 10 geographical regions. The datasets collected are representative for the studied regions.
Data collection	Data used in this study were collected from public sources: international databases, national databases, industry reports, and peer-reviewed journals. Data were collected by the authors manually. No instruments were used for data collection.
Timing and spatial scale	Data collection started from November, 2016 and finished in June, 2017. The historical data were available from 1931 to 2014. Following data collection, the modeling and simulations were performed since June, 2017. The study includes all countries/regions for which data were available.
Data exclusions	No data were excluded from the analysis. If data were not available for specific countries/regions, or years, data points were interpolated or extrapolated.
Reproducibility	This study is not based on experimental assessment; therefore, there are no experimental findings to replicate.
Randomization	There were no experimental groups; therefore, there was no assignment to experimental groups
Blinding	There were no experimental groups; therefore, there was no blinding to group allocation.

Did the study involve field work? [ ] Yes [x] No

Reporting for specific materials, systems and methods

We require information from authors about some types of materials, experimental systems and methods used in many studies. Here, indicate whether each material, system or method listed is relevant to your study. If you are not sure if a list item applies to your research, read the appropriate section before selecting a response.

Materials & experimental systems

n/a	Involved in the study
[x]	[ ] Antibodies
[x]	[ ] Eukaryotic cell lines
[x]	[ ] Palaeontology
[x]	[ ] Animals and other organisms
[x]	[ ] Human research participants
[x]	[ ] Clinical data

Methods

n/a	Involved in the study
[x]	[ ] ChIP-seq
[x]	[ ] Flow cytometry
[x]	[ ] MRI-based neuroimaging
